# Expression, Purification, and Comparative Inhibition of *Helicobacter pylori* Urease by Regio-Selectively Alkylated Benzimidazole 2-Thione Derivatives

**DOI:** 10.3390/molecules27030865

**Published:** 2022-01-27

**Authors:** Salih Osman Mohammed, El Sayed H. El Ashry, Asaad Khalid, Mohamed R. Amer, Ahmed M. Metwaly, Ibrahim H. Eissa, Eslam B. Elkaeed, Ahmed Elshobaky, Elsayed E. Hafez

**Affiliations:** 1 Medicinal and Aromatic Plants and Traditional Medicine Research Institute, National Center for Research, Khartoum 11111, Sudan; drasaad@gmail.com; 2 Chemistry Department, Faculty of Science, Alexandria University, Alexandria 21568, Egypt; Elasherysayed@yahoo.com (E.S.H.E.A.); RamdanM67@yahoo.com (M.R.A.); 3 Substance Abuse and Toxicology Research Center, Jazan University, Jazan 45142, Saudi Arabia; 4 Pharmacognosy and Medicinal Plants Department, Faculty of Pharmacy (Boys), Al-Azhar University, Cairo 11884, Egypt; ametwaly@azhar.edu.eg; 5 Biopharmaceutical Products Research Department, Genetic Engineering and Biotechnology Research Institute, City of Scientific Research and Technological Applications (SRTA-City), Alexandria 21934, Egypt; 6 Pharmaceutical Medicinal Chemistry & Drug Design Department, Faculty of Pharmacy (Boys), Al-Azhar University, Cairo 11884, Egypt; Ibrahimeissa@azhar.edu.eg; 7 Department of Pharmaceutical Sciences, College of Pharmacy, AlMaarefa University, Ad Diriyah, Riyadh 13713, Saudi Arabia; ikaeed@mcst.edu.sa; 8 Botany Department, Faculty of Science, Mansoura University, Mansoura 35516, Egypt; dshobaky84@yahoo.com; 9 Plant Protection and Biomolecular Diagnosis Department, City of Scientific Research and Technological Applications, ARADI, Alexandria 21934, Egypt

**Keywords:** *o*-phenylenediamine, regioselectivity, benzimidazole 2-thione, urease inhibition, molecular docking, recombinant urease

## Abstract

The urease enzyme has been an important target for the discovery of effective pharmacological and agricultural products. Thirteen regio-selectively alkylated benzimidazole-2-thione derivatives have been designed to carry the essential features of urease inhibitors. The urease enzyme was isolated from *Helicobacter pylori* as a recombinant urease utilizing the His-tag method. The isolated enzyme was purified and characterized using chromatographic and FPLC techniques showing a maximal activity of 200 mg/mL. Additionally, the commercial *Jack bean* urease was purchased and included in this study for comparative and mechanistic investigations. The designed compounds were synthesized and screened for their inhibitory activity against the two ureases. Compound **2** inhibited *H. pylori* and *Jack bean* ureases with IC_50_ values of 0.11; and 0.26 mM; respectively. While compound **5** showed IC_50_ values of 0.01; and 0.29 mM; respectively. Compounds **2** and **5** were docked against *Helicobacter pylori* urease (PDB ID: 1E9Y; resolution: 3.00 Å) and exhibited correct binding modes with free energy (ΔG) values of −9.74 and −13.82 kcal mol^−1^; respectively. Further; the in silico ADMET and toxicity properties of **2** and **5** indicated their general safeties and likeness to be used as drugs. Finally, the compounds’ safety was authenticated by an in vitro cytotoxicity assay against fibroblast cells.

## 1. Introduction

Urease is a dinickel enzyme that is found in several microbes, such as bacteria and fungi, in addition to plants and invertebrates. Urease was discovered in 1975 and was the first enzyme that nickel was vital for its action [1]. The function of urease is to catalyze the urea hydrolysis process to ammonia and carbonic acid [2].

Urease is an essential factor in the pathogenesis of various dangerous microbes. The pathogenic bacterium *Helicobacter pylori* can survive the acidic gastric through the utilization of urease to produce large amounts of ammonia and increase the pH [3].

*Streptococcus salivarius* depends on urease to produce ammonia, causing dental plaque and calculus [4]. *Klebsiella pneumoniae* and *Proteus mirabilis* rely on the urease to cause pneumonia, kidney stones, and urinary tract infections [5,6]. Additionally, the roles of urease in infection-induced reactive arthritis and acute pyelonephritis were reported [7,8].

In agriculture, the ureolytic bacteria increase ammonia amounts in the soil through fast urea degradation. This increase causes plant toxicity because of the high concentrations of ammonia and carbon dioxide [9].

Accordingly, the discovery of potent and safe urease inhibitors is an essential target in the fields of pharmaceutical and agricultural research. The urease inhibition effects of several synthetic compounds such as quinones [10], coumarins [11], triazoles [12], thiazoles [13], hydroxyurea [14], formamide [15], β-mercaptoethanol [16], acetohydroxamic acid [17], phosphoric triamide [18], boric acid [19], bismuth derivatives [20], and thiobarbituric acids [21] have been reported.

The benzimidazole derivatives drew attention to interesting pharmacophores in drug discovery. Benzimidazole derivatives have been utilized in medicinal chemistry for the development of antihistaminics [22], antiulceratives [23], anthelmintics [24], and antipsychotics [25]. Furthermore, several benzimidazoles have been reported as antiviral [26], anticoagulant [27], anti-inflammatory [28], antibacterial [29], and anticancer [30].

In the present study, we have expressed and purified the urease enzyme from *H. pylori*. Furthermore, the urease inhibitory effects of region selectively alkylated benzimidazole-2-thione derivatives have been investigated. Experimental enzyme kinetic and molecular docking studies were utilized to understand the mechanism of urease inhibition that was exhibited by the active compounds. The cytotoxicity activities of the active urease inhibitors were investigated as well.

### Rationale of the Design and Synthesis

**Figure 1:**
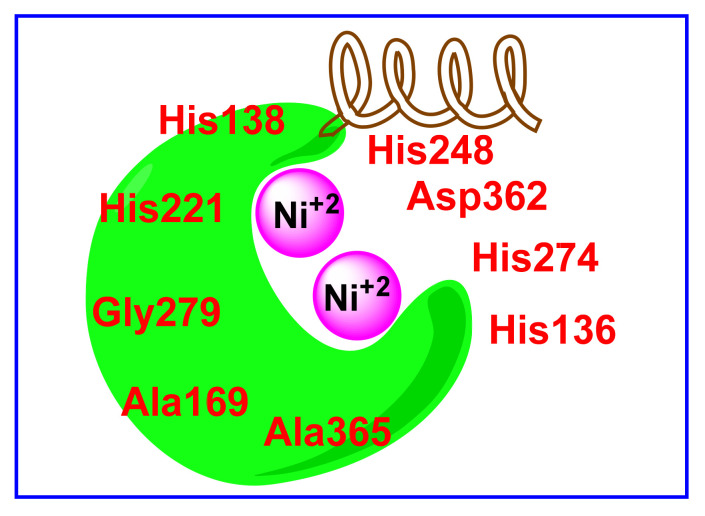
The active binding site of *Helicobacter pylori* hydrolase shows two Ni^+2^, important amino acids, and covering flap.

*H. pylori* urease has 12 active sites containing two Ni^2+^ ions each. The enzyme has a tetrahedral form consisting of four triangle shapes [31]. The active site of *H. pylori* urease is covered by a flap of a barrel-like shape. At the bottom of the barrel, the Ni^2+^ coordination site exists. This Ni^2+^ site contains both a penta- and hexacoordinate nickel, with coordinating ligands (Asp362, His274, His248, His136, and His138). The binding pocket is mostly lined with hydrophobic amino acids, such as Gly279, His221, Ala169, and Ala365. Crystal structures of the ureases show that, in addition to the conserved residues in the active site, the ureases share conserved residues that make up the mobile flap that covers the active site [32,33] (Figure 1).

Hydroxamic acid derivatives have emerged as the most promising candidates from among the different classes of inhibitors of urease. It was reported that hydroxamic acids with hydrophobic groups attached to them are more potent inhibitors of urease because they can easily penetrate the hydrophobic environment surrounding the active site. The -CONHO- moiety of the hydroxamic acid is also found to be necessary for chelation and inhibition of urease [34].

**Figure 2:**
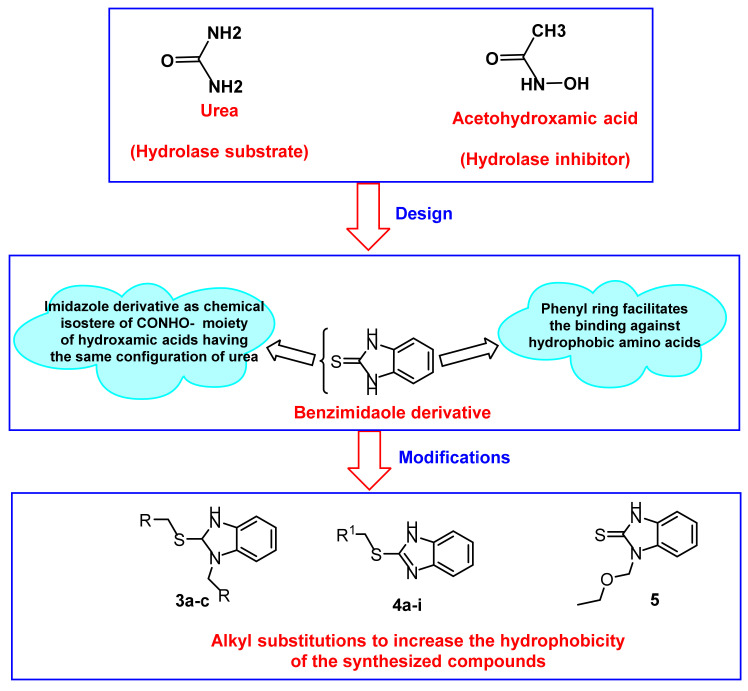
Molecular design rationale of the new proposed *Helicobacter pylori* hydrolase inhibitors.

In this work, a series of benzimidazole derivatives having the function group (-NHCSNH-) is considered as a chemical isostere for -CONHO- moiety of hydroxamic acids. In addition, the -NHCSNH- moiety has the same configuration as urea (the classical substrate of hydrolase). Moreover, the benzene ring of the designed benzimidazole derivatives may facilitate the binding against the hydrophobic amino acid residues in the active site. Furthermore, different alkyl substitutions were carried out to increase the hydrophobicity of the synthesized compounds, hoping to reach a more efficient hydrolase antagonist (Figure 2).

## 2. Results

### 2.1. Synthesis of Benzimidazole-2-Thione Derivatives

**Scheme 1:**
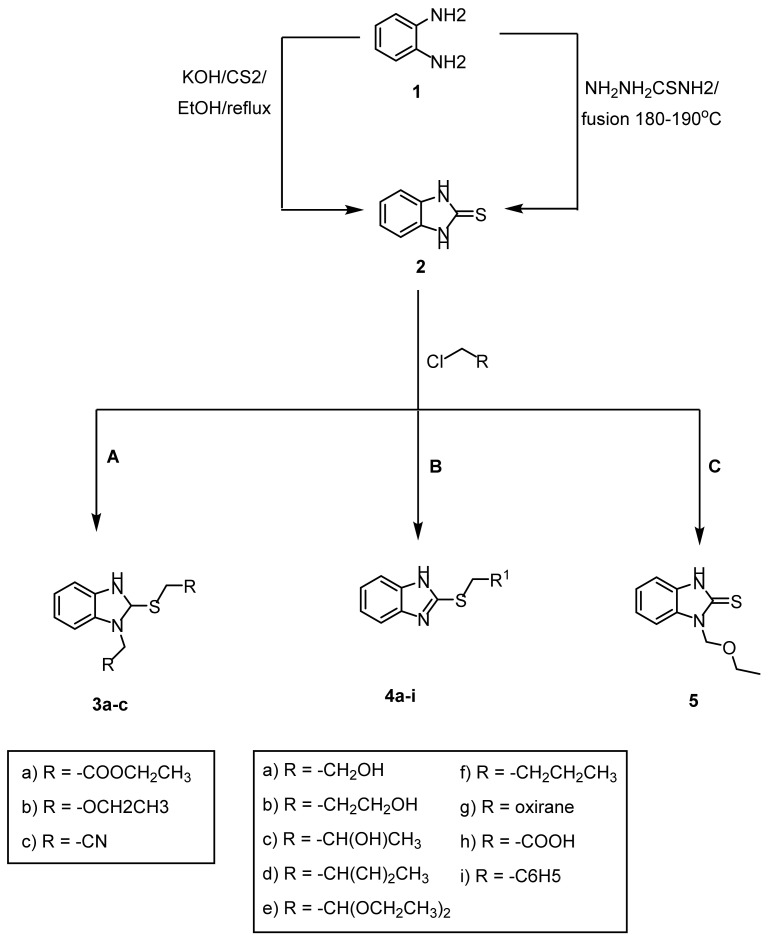
Alkylation of benzimidazole 2-thione; reagents and conditions: (**A**) K_2_CO_3_/DHF/acetone; (**B**) Triethylamine/DHF/acetone; (**C**) KOH/H_2_O/acetone.

In the present study, benzimidaole-thione (**2**) was utilized to prepare different derivatives through a regioselective alkylation (Scheme 1). The obtained compounds are the *S, N*-bis (alkylated) analogs **3a**, **3b** and **3c** as well as the *S*-alkylated analogs **4a**–**i** and *N*-alkylated analog **5**. The strategy for synthesizing the desired alkylated analoges was based on the possible regioselective alkylation of **2** with different alkylating agents under different conditions. The benzimidazole-2-thione **2** was prepared in a good yield using two different methods according to the literature [35,36] (Scheme 1).

Then, the reaction of **2** with equivalent amounts of the alkylating agents in the presence of K_2_CO_3_ in dry acetone/DMF mixture with stirring overnight gave *S,N*-bis(alkylated) analoges; ethyl-2-(1-(2-ethoxy-2-oxoethyl)-1*H*-benzo[*d*]imidazol-2-ylthio) acetate (**3a**), 1-(ethoxymethyl)-2-(ethoxymethylthio)-1*H*-benzo[*d*]imidazole (**3b**) and 2-(1-(cyanomethyl)-1H-benzo[*d*]imidazol-2-ylthio)acetonitrile (**3c**) [36], respectively, in (35–45%) yield. The structure of products was assigned based on the spectral data as well as the elemental analysis.

The ^1^H-NMR spectra of compounds **3a**, **3b** and **3c** indicated bis alkylation of **2** at the sulphur and nitrogen atoms by showing the presence of singlet signals at δ = 4.20 ppm, δ = 5.80 ppm and δ = 4.46 ppm that was assigned to SCH_2,_ and the presence of singlet signals at δ = 5.13 ppm, δ = 5.80 ppm and δ = 5.61 that was assigned to NCH_2,_ respectively. The ^13^C-NMR spectra of compound **3a** showed the presence of 2CH_2_ signals, one of them at δ = 34.2 ppm assigned to SCH_2_ and δ = 44.7 ppm assigned to NCH_2_. Similarly, compound **3c** showed SCH_2_ at δ = 17.8 ppm and NCH_2_ at δ = 31.8 ppm beside the remaining analysis that completely fit the proposed structures.

Also, the alkylation of **2** with a variety of alkyl halides (bromoethanol, 3-chloropropanol, chloropropan-2-ol,1-bromo-undecane, 2-bromo-1,1-diethoxyethane, 1-bromobutane, epichlorohydrin, 2-chloro acetic acid, and benzylbromide) could proceed through the formation of the corresponding *S*-alkylated analogs **4a**–**i**, presumably due to the higher nucleophilicity of the sulphur atom. Consequently, the salts of the thiolate anions generated by proton-abstraction from the thiol groups under the basic catalyst (K_2_CO_3_, Et_3_N and KOH) were initially formed and hence behaved as an ambient nucleophile in the nucleophilic substitution reactions. Direct coupling of the alkylating agents with **2** in K_2_CO_3_ in dry acetone gave the corresponding *S*-alkylated derivatives **4a**–**i** in 49–71% yields.

The ^1^H-NMR spectra of compounds **4a**–**i** showed the characteristic signal of SCH_2_ in the range of δ =3.11 ppm to δ =4.79 ppm besides the NH group. The ^13^C-NMR spectra for these derivatives showed SCH_2_ signals ranging from δ = 31.3 ppm to δ = 39.7 ppm and C-S signals from δ = 146.2 ppm to δ = 153.1 ppm.

**Figure 3:**
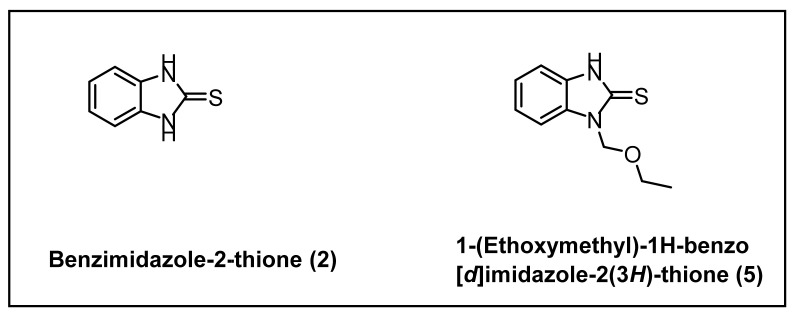
Structures of the bioactive urease inhibitors identified in this study.

Compound **5** (Figure 3) was synthesized by the reaction of benzimdazole-2-thione **2** with chloroethoxymethane, in water/acetone containing potassium hydroxide base medium. The reaction afforded the corresponding 1-(ethoxymethyl)-1*H*-benzo[d]imidazole-2(3*H*)-thione **5** in 65% yield, and the structure of **5** was established on analytical and spectral data. The IR spectrum showed bands due to NH and C=S at 3450 cm^−1^ and 1458 cm^−1^ and the mass spectrum of **5** showed a molecular ion peak at *m*/*z* 208, which compatible with molecular formula C_10_H_13_N_2_OS, ^1^H-NMR spectrum showed the characteristic signal due to SCH_2_ at δ = 5.66 ppm and NH at δ = 12.55 ppm. Both singlets, as well as ^13^C-NMR, showed the presence of C=S at δ = 169.6 ppm and SCH_2_ at δ = 27.1 ppm that completely fits the proposed structure **5**.

### 2.2. Preparation of Urease Enzyme

#### 2.2.1. Expression, Purification from *H. pylori*

**Figure 4:**
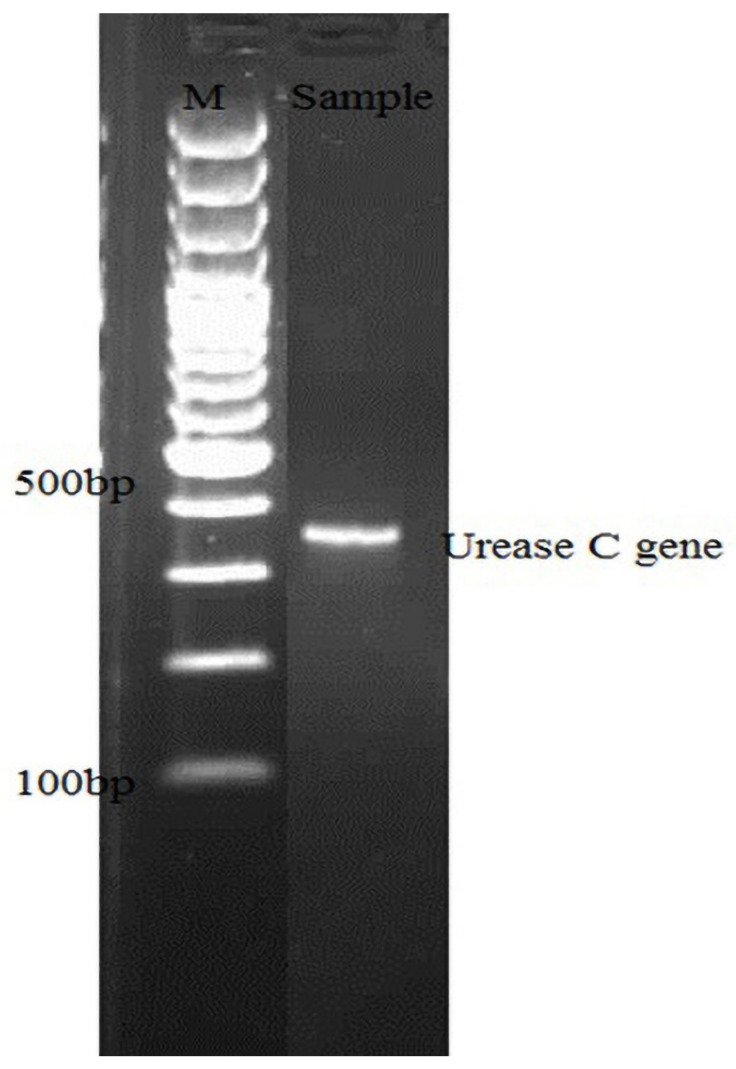
PCR product of the urease C gene isolated from *H. pylori*. Lane M: 100 bp DNA ladder. Lane 1: The urease gene with molecular size 360 bp.

The *H. pylori* DNA was extracted using a DNA extraction kit (Qiagene, Hilden, Germany). The Urease C gene was amplified by PCR using the purified genomic DNA as a template. Specific primers were synthesized to amplify the intact region of the genes according to Bickley [37]. The PCR product was then qualitatively analyzed on 1% agarose gel to give an expected size of 360 bp and deposited in the gene bank under the accession number of KF233428 (Figure 4). The PCR product was recovered using the Thermo Scientific gel extraction kit, and the amplified product was then purified and used for cloning and expression purposes.

#### 2.2.2. Cloning and Transmission of Purified PCR Product

**Figure 5:**
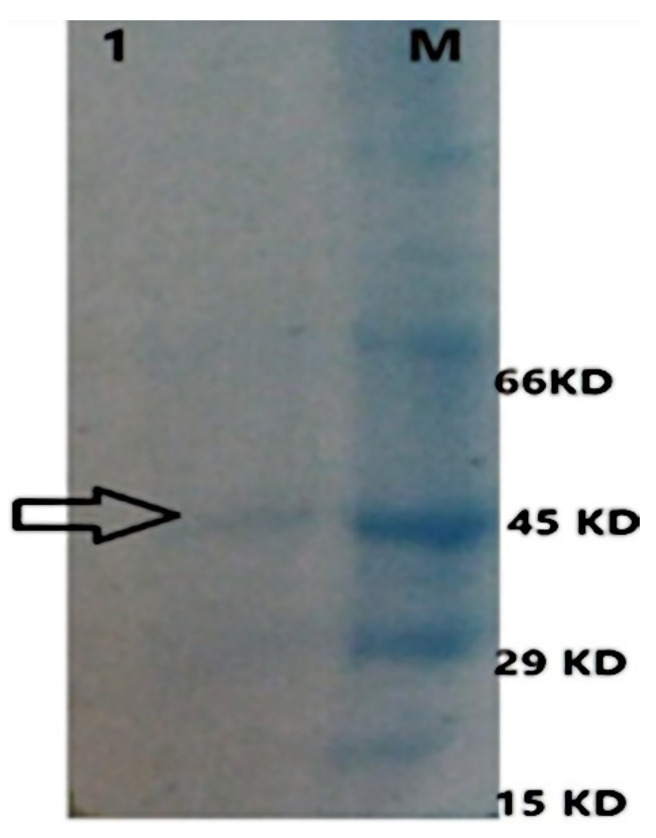
SDS-PAGE for the urease enzyme purified from the recombinant *E. coli*. Lane M: Unstained protein marker midrange. Lane 1: The purified urease band at a molecular size of approximately 45 kDa.

The recombinant gene of the pH6HTN His6HaloTag T7–urease C was transformed into a BL21 *E. coli* strain, and the fusion protein was expressed. The recombinant urease enzyme was purified from the recombinant *E. coli.* The obtained urease enzyme was determined using the Bradford method. It was observed that 200 mg/mL activity was determined. Moreover, the minimum activity of the enzyme after the evaluation was 20 mg/mL with a fold of 0.40. After that, the recombinant proteins from *E. coli* after being concentrated were injected on FPLC lop and detected by UV at the absorbance of 280 nm and the urease activity was observed in the first peak. The purified urease enzyme was analyzed by SDS-page for the fusion protein with predicted molecular masses of 45 KD (Figure 5).

**Figure 6:**
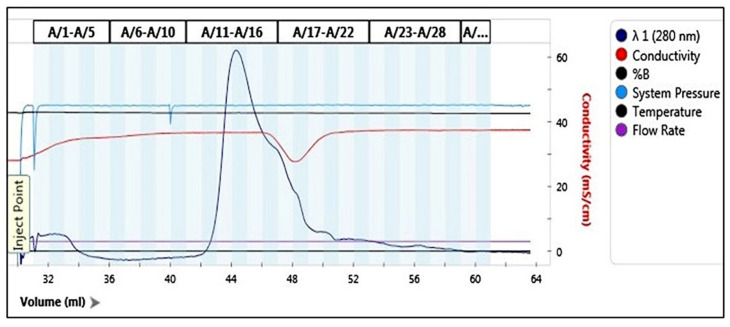
Gel filtration-chromatography (FPLC) of *H. pylori* urease C gene equilibrated with gel-permeation buffer at 0.5 mL/min, where the 4-mL volume of recommended proteins was applied to the column after adjusting the salt concentration to running buffer. Proteins were detected at 280 nm.

Different bacterial isolates were obtained, characterized, and identified as *H*. *pylori* using biochemical and molecular techniques. The molecular identification revealed that the selected isolates are *H. pylori* with identical 95 and 99%. Urease genes were amplified using specific primers and amplicons with molecular size 360 bp (*H. pylori* urease C gene) were observed (Figure 6). The obtained results were similar to those reported by Agent’s [38] and not agreed with by those obtained by Hossein [39]. The purified PCR products were quantified and then cloned into pGEM-T easy cloning vector, sub-cloned using *Eco*R1 into, pH6HTN His6HaloTag, prokaryotic expression vector, and the result revealed that white colons after transmission were observed and transferred into L.B medium containing IPTG. The overnight cells were subjected to lysis and protein purification. The protein purification by FPLC column chromatography showed single peaks (Figure 6). The activities enzyme purified was 200 mg/mL with fold 0.40 with the recovery of 98%, these results were similar to 190,230 mg/mL that was reported by Mohamed [40] and Pınar [41] and different to 200 mg/mL. When comparing the activity of the recombinant urease with the standard urease enzyme (*J. bean*), the *H. pylori* purified urease gene enzyme showed a similar ammonia production to the standard urease enzyme. When the fractions were separated on SDS-PAGE gel faint band at molecular weight 45 kDa (Figure 6) with the pooled fractions from the column chromatography, very distinct bands were observed in the FPLC fraction. The results are similar to those obtained by Hossein [39] and Leila [42].

### 2.3. Urease Inhibition Assay

The alkylated benzimidazole 2-thione derivatives were screened for urease inhibition. Only two compounds, **2** and **5** exhibited interesting inhibition activities against urease enzymes. Other compounds showed weak inhibition with percentage values less than 40% at 1 mM concentrations. The structures of the active compounds are shown in Figure 3. The results in Table 1 shows that compound **2** was found more specific to *H. pylori* than the *J. bean* urease enzyme. Compound **5** was found more specific to *H. pylori* urease than the *J. bean* urease enzyme. It was observed that compound **2** is one of the non-competitive inhibitors against *H. pylori,* and *J. bean* ureases. This compound was able to decrease the V*_max_* value without affecting the affinity of the enzyme-substrate (K_m_ value). On the other hand, compound **5** founded to be an uncompetitive inhibitor against *H. pylori* urease enzymes as it decreased both the V_max_ and K_m_ values. Controversially, the compound appeared to be a non-competitive inhibitor of *J. bean* urease as it only decreased the V*_max_* value without affecting the affinity of the enzyme-substrate (K_m_ value). Other compounds from the alkylation of the benzimidazole 2-thione group, showed weak activity against all the urease enzymes.

### 2.4. Molecular Docking

Depending on Figure 1, which clarified the essential amino acid residues in the catalytic active site of *H pylori* hydrolase, we carried out the docking studies for the most active compounds **2** and **5** against *Helicobacter pylori* urease (PDB ID: 1E9Y, resolution: 3.00 Å). The docking studies were carried out to investigate the possible binding interaction of the synthesized compounds against the prospective biological target (*Helicobacter pylori* urease).

**Figure 7:**
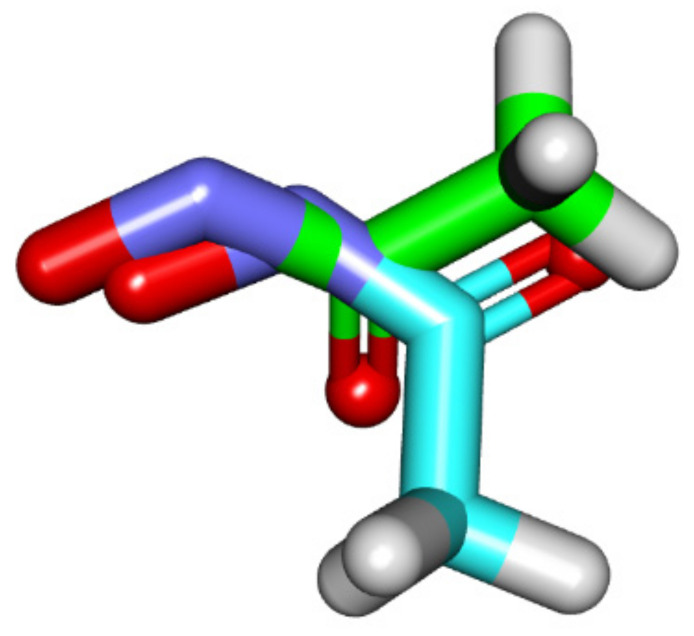
Superimposition of the co-crystallized molecule (green) and the docking pose (turquoise) of the same molecule.

Validation of the docking process was achieved through the running of the docking procedure for only the co-crystallized ligand ((acetohydroxamic acid, **HAE**) against the active pocket. It was found that the produced RMSD value between the generated pose of the docked molecule and the original one was equal to 1.63 Å. This indicates the validity of the docking process (Figure 7).

At first, the binding mode of the classical urease inhibitor (acetohydroxamic acid, HAE) was investigated as a reference molecule. Additionally, the binding role of the two Ni^+2^ at the active site was clarified. Then, the binding pattern of the synthesized compounds (**2** and **5**) was examined to compare their binding mode with that of the reference molecule (HAE).

In these studies, we focused on the essential amino acids (Asp362, His274, His248, His136, His13, Gly279, His221, Ala169, and Ala365) in the catalytic active site of hydrolase.

Comprehensive docking studies were carried out using MOE14.0 software. These studies resulted in free energy (ΔG) values which indicate the binding interaction of the tested molecules with the target protein, as shown in Table 2.

**Figure 8:**
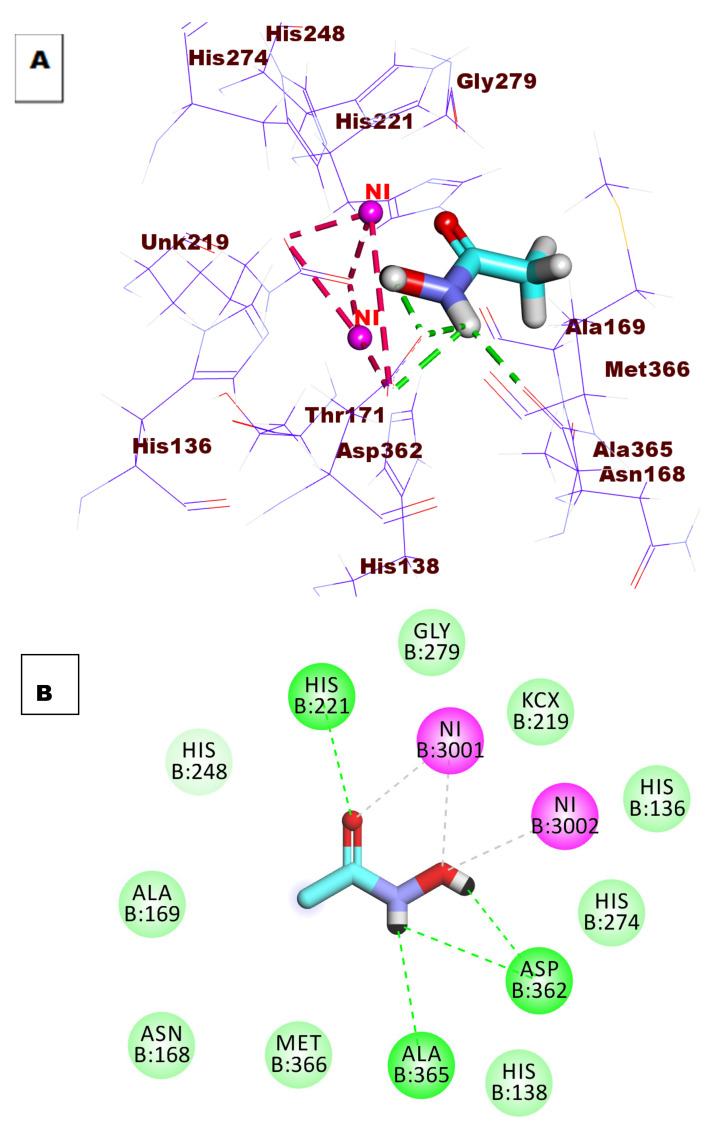
(**A**) 3D of co-crystallized ligand (**HAE**) docked into the active site of *Helicobacter pylori* urease. (**B**) 2D of co-crystallized ligand (**HAE**) docked into the active site of *Helicobacter pylori* urease.

The binding mode of the co-crystallized ligand (acetohydroxamic acid, **HAE**) against *Helicobacter pylori* urease showed binding energy of −7.78 Kcal. Mol^−1^). It exhibited four hydrogen bonds with the essential amino acid residues in the active site. In addition, such compounds exhibited three electrostatic interactions with Ni^+2^ which facilitate the interaction with the receptor. The C=O group was incorporated in the hydrogen bonding interaction with His221. Additionally, The OH group formed a hydrogen bond with Asp362. Furthermore, the NH group exhibited two hydrogen bonds with Ala365 and Asp362 (Figure 8).

**Figure 9:**
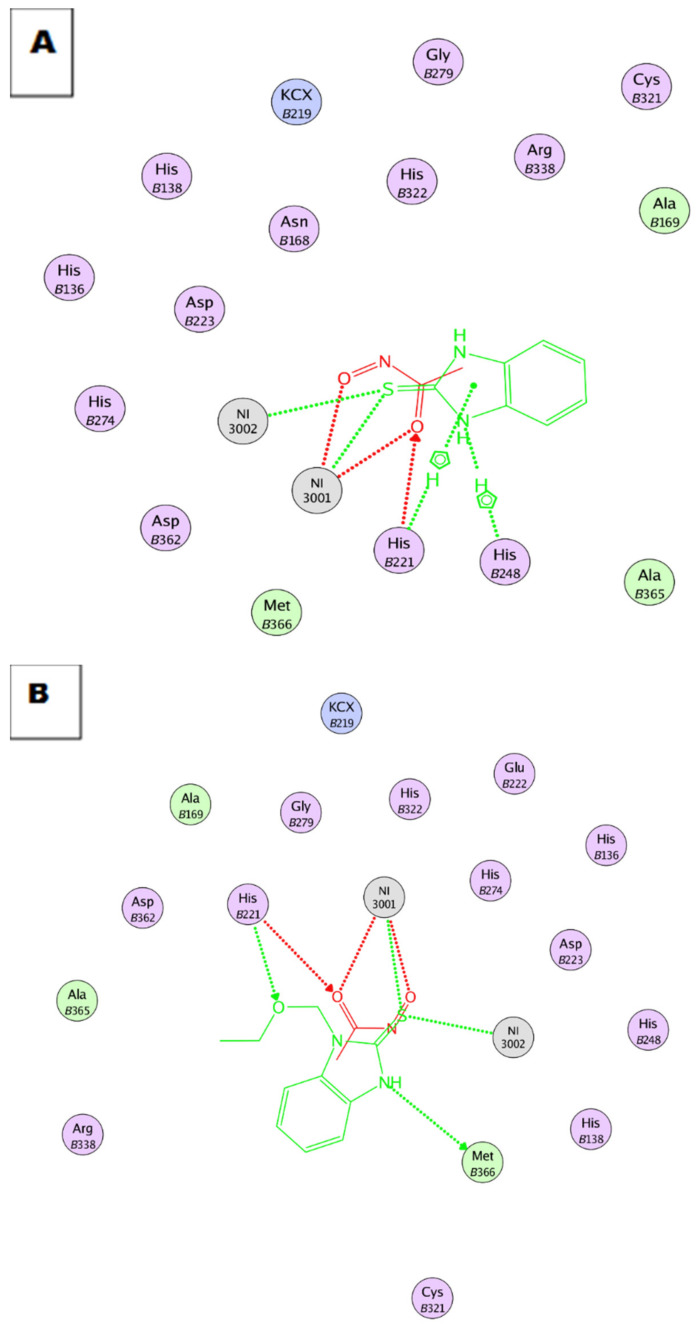
Overlay of compounds **2** (**A**) and **5** (**B**) against the co-crystallized ligand (HAE) in the active site showing the same binding mode.

The results of the docking studies revealed that compounds **2** and **5** had occupied the same pocket and showed the like binding mode of the co-crystallized ligands. Interestingly, the hydrophobic moieties of the synthesized compounds were incorporated in many hydrophobic interactions in the active and resulted in higher binding energy than the co-crystallized ligand (Figure 9).

**Figure 10:**
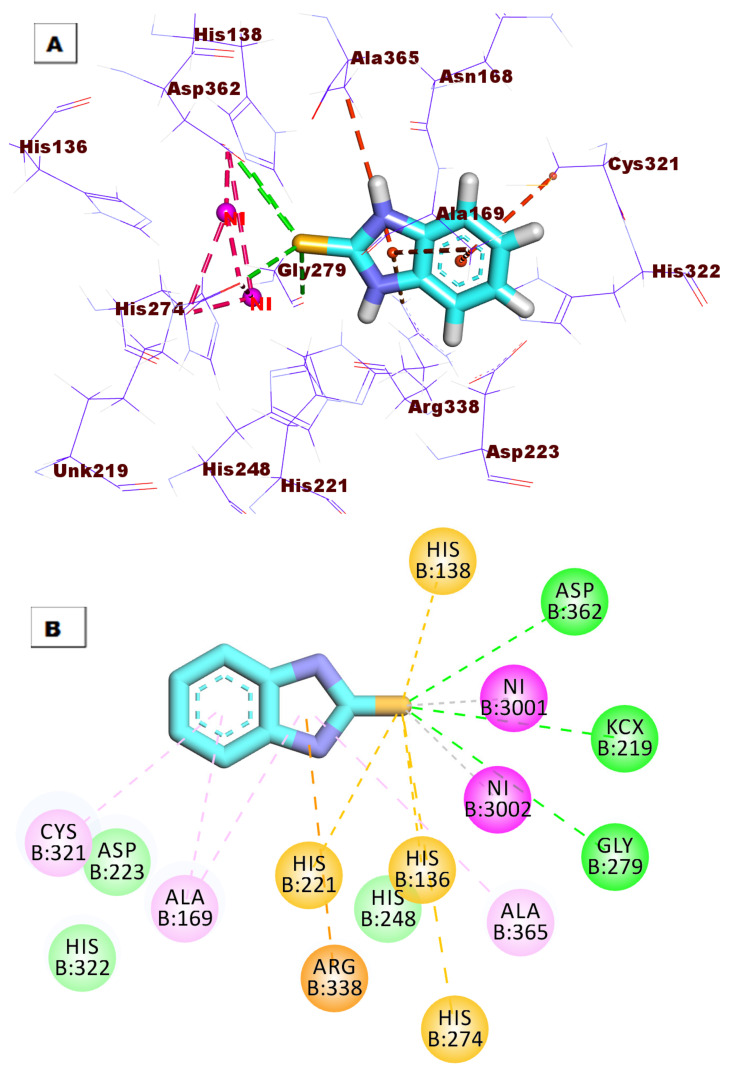
(**A**) 3D and (**B**) 2D of compound **2** docked into the active site of Helicobacter pylori urease.

The binding mode of compound **2** against *Helicobacter pylori* urease showed binding energy of −13.89 kcal/mol. It exhibited three hydrogen bonds, four hydrophobic interactions, and seven electrostatic attractions. In detail, the C=S group formed three hydrogen bonds with Gly279, Asp362, inside the urease subunit KCX219. In addition, The C=S group formed four electrostatic interactions with His221, His136, His274, and His138. Moreover, the C=S moiety formed two co-ordinate interactions with the Ni^+2^. Additionally, the aromatic system formed four hydrophobic interactions with Ala365, Ala169, Cys321, and Ala365. Additionally, the imidazole ring formed one electrostatic attraction with Arg338 (Figure 10).

**Figure 11:**
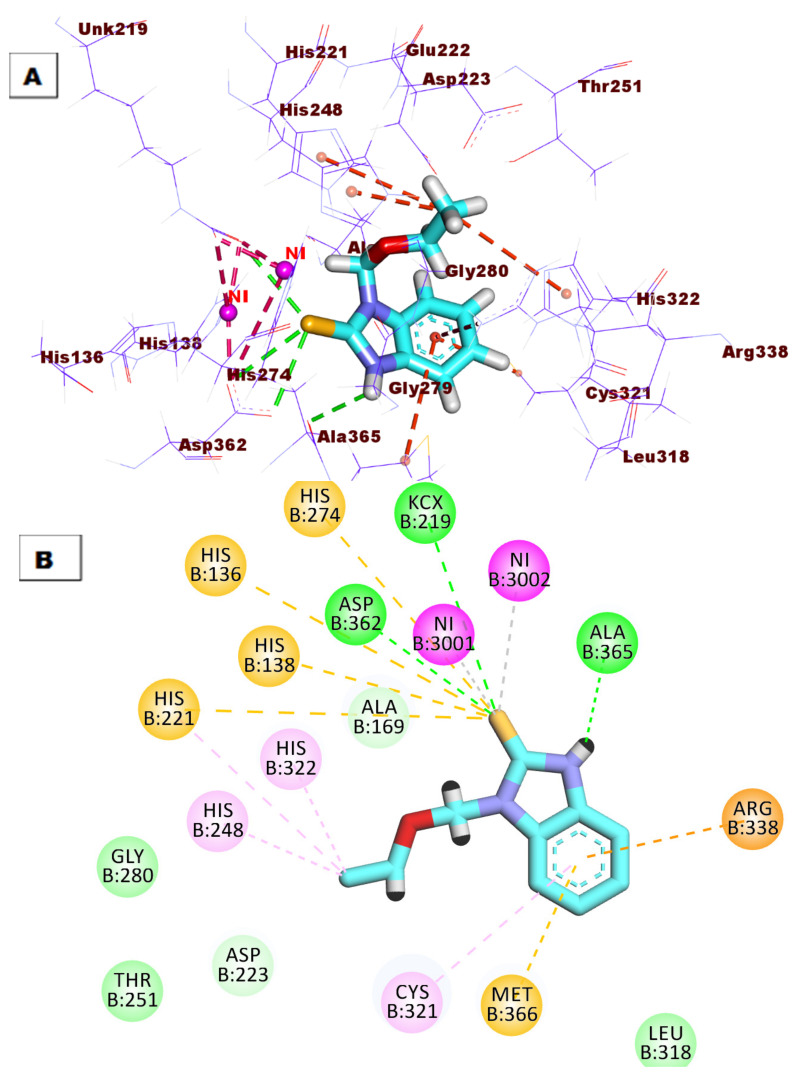
(**A**) 3D and (**B**) 2D of compound **5** docked into the active site of Helicobacter pylori urease.

Compound **5** showed good binding mode against *Helicobacter pylori* urease with a high binding energy of −17.20 kcal/mol). The NH group formed one hydrogen bond with Ala365. The C=S group formed two hydrogen bonds with Asp362 inside the urease subunit KCX219. Additionally, it formed four electrostatic interactions with His274, His136, His138, His221. Additionally, it formed two co-ordinate interactions with the two nickel atoms in the active site. The ethoxymethyl group formed three hydrophobic interactions with His322, His248, and His221. The aromatic system formed one hydrophobic interaction with Cys321, and two electrostatic interactions with Arg338 and Met366 (Figure 11).

### 2.5. ADMET Studies

In silico ADMET parameters were investigated for the most active candidates **2** and **5**. The co-crystallized ligand (acetohydroxamic acid, **HAE**) was used as a reference drug. Discovery Studio 4.0 was used to predict ADMET descriptors. The predicted descriptors are listed in Table 3.

The results revealed that compound **2** has a medium BBB penetration level, whereas compound **5** showed high BBB penetration power.

**Figure 12:**
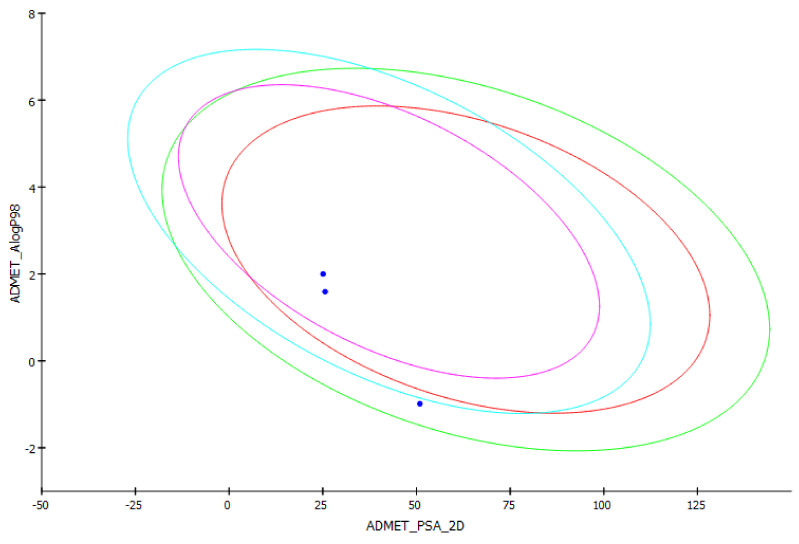
The expected ADMET study.

For aqueous solubility and intestinal absorption levels, the tested compounds exhibited a good level. Both **2** and **5** were also predicted to be a non-inhibitor of CYP2D6. The plasma protein binding revealed that compound **2** exhibited plasma protein binding power ˂ 90%. In contrast, compound **5** showed plasma protein binding power ˂ 90% (Figure 12).

### 2.6. Toxicity Studies

Toxicity prediction was carried out for compounds **2** and **5** based on the validated and constructed Discovery Studio software [43,44].

Table 4 showed that the tested compounds have low toxicity. For the carcinogenic potency TD_50_ rat model, compound **2** showed a TD_50_ value of 74.796 mg/kg body weight/day, less than that of the reference drug HAE (82.223 mg/kg body weight/day). On the other hand, compound **5** showed a higher TD_50_ value (127.982 mg/kg body weight/day) than HAE.

Regarding the rat maximum tolerated dose model, the tested compounds **2** and **5** showed a maximum tolerated dose of 0.115 and 0.072 g/kg body weight, respectively. These values were less than the reference compound (0.135 g/kg body weight).

For the rat oral LD_50_ model, the tested compounds showed oral LD_50_ values of 0.271 and 0.314 mg/kg body weight/day, respectively. These values were less than that of HAE (1.090 mg/kg body weight/day). For the rat chronic LOAEL model, the tested compounds showed LOAEL values of 0.069 and 0.034 g/kg body weight, respectively, which were less than HAE (0.423 g/kg body weight). The tested compounds were predicted to be an irritant against the skin and ocular irritancy models.

### 2.7. In Vitro Cytotoxicity

The two active urease inhibiting compounds were subjected to a cytotoxicity test. The obtained data clarified that both compounds **2** and **5b** showed cellular viability IC_50_ at 0.031 and 0.062 µM, respectively. The result indicated that compounds **2** and 5 are not cytotoxic in high concentrations (Table 5).

## 3. Experimental

### 3.1. Compound Synthesis

#### 3.1.1. Preparation of 1H-Benzimidazole-2(3H)-thione (2)

Melting points were determined with a Mel-Temp apparatus (SMP10) in open capillaries and are uncorrected. TLC was performed on E. Merck Silica Gel 60 F_254_ with detection by UV light absorption. ^1^H NMR spectra were recorded on a Bruker Avance AV NMR spectrometer at 300 or 400 MHz, whereas the ^13^C NMR was recorded on the same instrument at 75 or 100 MHz, respectively, with TMS as the internal standard. Mass spectra were recorded on a Finnigan (MAT312) and Jeol (JMS.600H) instrument; HRMS was recorded with Thermo Finnegan (MAT 95XP). Solvents used were purified by simple distillation.

Method (i): A mixture *o*-phenylene diamine **1** (5.4 g, 0.05 mol), and thiosemicarbazide (4.6 g, 0.05 mol) was heated in an oil bath at 180–190 °C, where the mixture was melted and then solidified after 1 h. The reaction mixture was cooled and recrystallized from ethanol to give a brown solid.

Method (ii): *o*-Phenylene diamine **1** (17.7 g, 0.1 mol) was suspended in a mixture of ethanol 100 mL, and water 15 mL. Carbon disulfide (9 g, 0.11 mol), and potassium hydroxide (6.3 g, 0.11 mol) was added, and the mixture was heated at reflux for 6 h. A charcoal 4 gm was added and refluxed for 10 min. The hot solution was filtration and diluted with water 100 mL, and 8 mL of glacial acetic acid. The precipitate was collected by filtration, and recrystallization from ethanol the glistening white crystals was obtained. Yield (method (i): 69%, (ii): 86% mp 299 °C (lit. mp 303–304 °C); TLC, R_f_ 0.57, (4:6 EtOAc-n-hexane); ^1^H NMR (DMSO-d_6_, 300 MHz): δ 7.07–7.14 (m, 4H, ArH), 12.50 (s, 2H, 2NH); ^13^C NMR (DMSO-d_6,_ 75 MHz): δ 109.3 (C-7, C-4), 122.2 (C-5, C-6), 132.2 (C-8, C-9), 168.1 (C=S); EIMS: *m*/*z* (%) = 150 (100), 118 (28), 106 (14), 91 (11), 75 (12); HREIMS (M^+^): Calcd for C_7_H_6_N_2_S: *m*/*z* 150.0252, Found 150.0236.

#### 3.1.2. General Procedure for the Synthesis of Compounds **3a**–**c**

A mixture of compound **2** (0.15 g, 1.0 mmol), and potassium carbonate (0.2 g, 1.5 mmol) were stirred for 1 h in dry acetone (25 mL) containing DMF (2 mL), and then the appropriate alkyl halides (1.2 mmol) were added. Stirring was continued overnight and the mixture was filtered and washed with acetone. The solvent was evaporated under reduced pressure and the product was subjected to column chromatography to afford compounds **3a**–**c**.

*Ethyl-2-(1-(2-ethoxy-2-oxoethyl)-1H-benzo[d]imidazol-2-ylthio)acetate* (**3a**) Yield (method A: 41%, B: 38 %), yellow gum, TLC, R_f_ 0.71 (1:1 EtOAc-n-hexane); ^1^H NMR (DMSO-d_6_, 300 MHz): δ 1.14–1.24 (m, 6H, 2CH_3_), 4.07–4.18 (m, 4H, 2CH_2_), 4.20 (s, 2H, SCH_2_), 5.13 (s, 2H, NCH_2_), 7.14–7.21 (m, 2H, ArH), 7.48–7.54 (m, 2H, ArH); ^13^C NMR (DMSO-d_6,_ 125 MHz): δ 13.8, 13.9 (2CH_3_), 34.2 (SCH_2_), 44.7 (NCH_2_), 61.2, 61.4 (2OCH_2_), 109.7 (C-7), 117.8 (C-4), 121.8, 121.9 (C-5, C-6), 136.4 (C-9), 142.5 (C-8), 150.6 (C-S), 167.4, 168.2 (2C=O); EIMS: *m*/*z* (%) = 323 (89), 277 (17), 249 (100), 236 (8), 221 (32), 175 (60), 163 (21), 118 (29), 77 (10); HREIMS (M^+^): Calcd for C_15_H_18_N_2_O_4_S: *m*/*z* 322.0987, Found 322.0965.

*1-(Ethoxymethyl)-2-(ethoxymethylthio)-1H-benzo[d]imidazole* (**3b**) Yield (method A: 35%), colorless crystals, mp 108 °C; TLC, R_f_ 0.70 (4:6 EtOAc-n-hexane); ^1^H NMR (CDCl_3_, 300 MHz): δ 1.16 (t, 6H, J = 7.0 Hz, 2CH_3_), 3.63 (q, 4H, 2OCH_2_), 5.80 (s, 4H, SCH_2_, NCH_2_), 7.24–7.26 (m, 2H, ArH), 7.36–7.39 (m, 2H, ArH); EIMS: *m*/*z* (%) = 266 (26), 237 (5), 233 (5), 191 (35), 163 (52), 118 (20), 90 (8); Anal. Calcd for C_13_H_18_N_2_O_2_S: C, 58.62; H, 6.81; N, 10.52. Found: C, 58.92; H, 6.92; N, 10.58.

*2-(1-(Cyanomethyl)-1H-benzo[d]imidazol-2-ylthio)acetonitrile* (**3c**) Yield (method A: 45%, B: 55%), White powder, mp 161–163 °C; TLC, R_f_ 0.67 (4:6 EtOAc-n-hexane); ^1^H NMR (DMSO-d_6_, 300 MHz): δ 4.46 (s, 2H, SCH_2_), 5.61 (s, 2H, NCH_2_), 7.26–7.36 (m, 2H, ArH), 7.66–7.71 (m, 2H, ArH); ^13^C NMR (DMSO-d_6_, 75 MHz): δ 17.8 (SCH_2_), 31.8 (NCH_2_), 109.9 (C-7), 115.1, 117.5 (2 CN), 118.5 (C-4), 122.9 (C-5),123.1 (C-6), 135.5 (C-9) 142.5 (C-8), 148.5 (C-S); EIMS: *m*/*z* (%) = 228 (55), 200 (2), 188 (47), 161 (100), 134 (39), 149 (3), 90 (27); HREIMS (M^+^): Calcd for C_11_H_8_N_4_S: *m*/*z* 228.0470. Found: 228.0498.

#### 3.1.3. General Procedure for the Synthesis of Compounds **4a**–**i**

A mixture of compound **2** (0.15 g, 1.0 mmol), and triethylamine (0.14 mL, 1.0 mmol) in dry acetone (25 mL) containing DMF (2 mL). Then the appropriate alkyl halides (1.2 mmol) were added. Stirring was continued overnight and the mixture was filtered and washed with acetone. The solvent was evaporated under reduced pressure and the product was subjected to column chromatography to give titled compounds **4a**–**i**.

*2.-(1. H-Benzo[d]imidazol-2-ylthio)ethanol***(4a)** Yield (method A: 71%, B: 64%, C: 54%), yellow crystals, mp 130 °C; TLC, R_f_ 0.14 (4:6 EtOAc-n-hexane); ^1^H NMR (DMSO-d_6,_ 300 MHz): δ 3.34 (t, 2H, J = 6.5 Hz, CH_2_O), 3.69 (t, 2H, J = 6.4 Hz, SCH_2_), 6.90 (s, 1H, ArH), 7.07–7.10 (m, 2H, ArH), 7.41 (s, 1H, ArH), 10.55 (s, 1H, OH), 12.53 (s, 1H, NH); ^13^C NMR (DMSO-d_6_, 75 MHz): δ 33.8 (SCH_2_), 60.3 (CH_2_O), 108.4 (C-7, C-4), 120.3 (C-6, C-5), 129.6 (C-8, C-9), 150.3 (C-S); EIMS: *m*/*z* (%) = 194 (73), 150 (100), 118 (23), 79 (6); ESIMS: Calcd for C_9_H_11_N_2_OS (M^+^+H), *m*/*z* 195.0592. Found: 195.0586.

*3-(1H-Benzo[d]imidazol-2-ylthio)propan-1-ol* (**4b**) Yield (method A: 61 %, B: 67%, C: 54%), white crystals, mp 119 °C, TLC, R_f_ 0.31 (4:6 EtOAc-n-hexane); ^1^H NMR (DMSO-d_6,_ 300 MHz): δ 1.76–1.88 (m, 2H, CH_2_-2′), 3.29 (t, 2H, J = 7.1 Hz, SCH_2_), 3.53 (t, 2H, J = 6.1 Hz, CH_2_O), 7.09–7.12 (m, 2H, ArH), 7.40 (s, 2H, ArH), 12.53 (s, 1H, NH); ^13^C NMR (DMSO-d_6,_ 75 MHz): δ 28.1 (C-2′), 32.5 (SCH_2_), 59.0 (OCH_2_), 121.3 (ArH), 150.4 (C-S); EIMS: *m*/*z* (%) = 208 (7), 177 (13), 150 (100), 118 (17), 78 (6); HREIMS (M^+^): Calcd for C_10_H_12_N_2_OS: *m*/*z* 208.0670. Found: 208.0679.

*1-(1H-Benzo[d]imidazol-2-ylthio)propan-2-ol* (**4c**) Yield (method A: 51%, B: 53%, C: 44%), colorless crystals, mp 250 °C; TLC, R_f_ 0.26 (4:6 EtOAc-n-hexane); ^1^H NMR (CDCl_3_, 300 MHz): δ 1.17 (d, 3H, J = 6.3 Hz, CH_3_), 3.11 (d, 2H, J = 5.4 Hz, SCH_2_), 3.93–4.03 (m, 1H, CH), 7.01–7.06 (m, 2H, ArH), 7.30–7.33 (m, 2H, ArH); ^13^C NMR (DMSO-d_6_, 75 MHz): δ 22.46 (CH_3_), 39.7 (SCH_2_), 65.4 (CH), 108.4 (C-7), 110.2 (C-4), 117.1 (C-6), 120.3 (C-5), 121.2 (C-8, C-9), 150.7 (C-S); EIMS: *m*/*z* (%) = 208 (12), 193 (14), 150 (100), 132 (2), 78 (8); FABMS: *m*/*z* 209 [M^+^+1]; HREIMS (M^+^): Calcd for C_10_H_12_N_2_OS: *m*/*z* 208.0670. Found: 208.0677.

*2-(n-Undecanylthio)-1H-benzo[d]imidazole* (**4d**) Yield (method A: 64%, B: 88%, C: 78%), white crystals, mp 104 °C; TLC, R_f_ 0.67 (4:6 EtOAc-n-hexane); ^1^H NMR (CDCl_3_, 300 MHz): δ 0.85 (t, 3H, J = 6.8 Hz, CH_3_), 1.23 (br s, 14H, 7CH_2_), 1.39–1.44 (m, 2H, CH_2_-3′), 1.69–1.77 (m, 2H, CH_2_-2′), 3.31 (t, 3H, J = 7.3 Hz, SCH_2_), 7.15–7.19 (m, 2H, ArH), 7.29–7.32 (m, 1H, ArH), 7.64–7.67 (m, 1H, ArH), 8.95 (s, 1H, NH); ^13^C NMR (CDCl_3,_ 100 MHz): δ 14.1 (CH_3_), 22.6 (CH_2_-10′), 28.7, 29.1, 29.3, 29.4, 29.5, 29.6 (7CH_2_) 31.8 (CH_2_-2′), 32.8 (SCH_2_), 122.2 (ArH), 150.9 (C-S); EIMS: *m*/*z* (%) = 304 (6), 257 (14), 205 (5), 177 (5), 150 (100), 122 (6); HREIMS (M^+^): Calcd for C_18_H_28_N_2_S: *m*/*z* 304.1973. Found: 304.1980.

*2-(2,2-Diethoxyethylthio)-1H-benzo[d]imidazole* (**4e**) Yield (method A: 49%, B: 51%, C: 60%), yellow powder, mp 80 °C, TLC, R_f_ 0.53 (4:6 EtOAc-n-hexane); ^1^H NMR (CDCl_3_, 300 MHz): δ 1.27 (t, 6H, J = 7.0 Hz, 2CH_3_), 3.32(d, 1H, J = 5.3 Hz, CH), 3.61–3.70 (m, 2H, OCH_2_), 3.76–3.84 (m, 2H, OCH_2_), 4.79 (t, 2H, J = 5.2 Hz, SCH_2_), 7.16–7.24 (m, 2H, ArH), 7.40 (s, 1H, ArH), 7.8 (s, 1H, ArH), 10.25 (s, 1H, NH); ^13^C NMR (CDCl_3_, 75 MHz): δ 15.1 (2CH_3_), 35.9 (SCH_2_), 63.3 (2CH_2_), 102.8 (CH), 122.1 (ArH), 150.6 (C-S); EIMS: *m*/*z* (%) = 266 (10), 221 (25), 193 (4), 175 (18), 150 (60), 117 (24), 103 (100); HREIMS (M^+^): Calcd for C_13_H_18_N_2_O_2_S: *m*/*z* 266.1089. Found: 266.1103.

*2-(Butylthio)-1H-benzo[d]imidazole* (**4f**) Yield (method A: 61%), white crystals, mp 145 °C, TLC, R_f_ 0.91 (4:6 EtOAc-n-hexane); ^1^H NMR (DMSO-d_6_, 300 MHz): δ 0.89 (t, 3H, J = 7.3 Hz, CH_3_), 1.41 (q, 2H, CH_2_-3′), 1.67 (q, 2H, CH_2_-2′), 3.26 (t, 2H, SCH_2_), 7.07–7.11 (m, 2H, ArH), 7.42 (d, 2H, ArH), 12.54 (s, 1H, NH); ^13^C NMR (DMSO-d_6_, 75 MHz): δ 13.4 (CH_3_), 21.2 (CH_2_-3′), 30.8 (CH_2_-2′), 31.3 (SCH_2_), 110.2 (C-7), 117.2 (C-4), 120.9 (C-5), 121.4 (C-6), 135.4 (C-8), 143.7 (C-9), 150.2 (C-S); EIMS: *m*/*z* (%) = 206 (17), 177 (10), 150 (100), 122 (21), 90 (8), 78 (3); HREIMS (M^+^): Calcd for C_11_H_14_N_2_S: *m*/*z* 206.0878. Found: 206.0864.

*2-(Oxiran-2-ylmethylthio)-1H-benzo[d]imidazole* (**4g**) Yield (method A: 55%, B: 50%, C: 48%), yellow crystals, mp 211 °C; TLC, R_f_ 0.22 (4:6 EtOAc-n-hexane); ^1^H NMR (DMSO-d_6_, 300 MHz): δ 2.48–3.41 (m, 2H, CH_2_), 4.02–4.46 (m, 1H, CH), 5.70 (d, 2H, J = 3.9 Hz, SCH_2_), 7.11–7.14 (m, 2H, ArH), 7.39–7.44 (m, 2H, ArH); ^13^C NMR (DMSO-d_6_, 75 MHz): δ 31.3 (SCH_2_), 48.2(CH_2_), 60.6 (CH), 108.6 (C-7), 116.9 (C-4), 120.7 (C-5), 121.7 (C-6), 135.8 (C-8), 142.8 (C-9), 146.2 (C-S); EIMS: *m*/*z* (%) = 206 (100), 162 (78),148 (13), 118 (99), 77 (8); HREIMS (M^+^): Calcd for C_10_H_10_N_2_OS: *m*/*z* 206.0514. Found: 206.0527.

*2-(1H-Benzo[d]imidazol-2-ylthio)acetic acid* (**4h**) Yield (method A: 50%), yellow powder, mp 250 °C; TLC, R_f_ 0.48 (2:8 MeOH-CH_2_Cl_2_); ^1^H NMR (DMSO-d_6_, 300 MHz): δ 3.65 (s, 2H, SCH_2_), 4.22 (s, 1H, OH), 7.08–7.11 (m, 2H, ArH), 7.41 (m, 2H, ArH), 12.59 (s, 1H, NH); ^13^C NMR (DMSO-d_6_, 75 MHz): δ 37.8 (SCH_2_), 120.8 (ArH), 153.1 (C-S), 170.4 (C=O); FABMS: *m*/*z* 209 [M^+^+1]; HREIMS (M^+^): Calcd for C_9_H_8_N_2_O_2_S: *m*/*z* 208.0306. Found: 208.0293.

*2-(Benzylthio)-1H-benzo[d]imidazole* (**4i**) Yield (method A: 55%, B: 67%, C: 49%), yellow crystals, mp 185 °C; TLC, R_f_ 0.6 (4:6 EtOAc-n-hexane); ^1^H NMR (MeOD, 300 MHz): δ 4.55 (s, 2H, SCH_2_), 7.08–7.13 (m, 2H, ArH), 7.20–7.32 (m, 5H, ArH), 7.14–7.44 (m, 2H, ArH), 12.53 (s, 1H, NH). ^13^C NMR (DMSO-d_6_, 75 MHz): δ 35.1 (SCH_2_), 121.4, 127.3, 128.4, 128.8, 137.6 (ArH), 149.6 (C-S); EIMS: *m*/*z* (%) = 240 (36), 207 (21), 163 (5), 149 (8), 122 (11), 91 (100); ESIMS: Calcd for C_14_H_13_N_2_S (M^+^+H), *m*/*z* 241.0793. Found: 241.0790.

#### 3.1.4. General Procedure for the Synthesis of Compound **5**

A mixture of compound **2** (1.5 g, 0.01 mol) in aqueous potassium hydroxide (0.6 g, 0.01 mol) in water (15 mL) was treated with the appropriate alkyl halide (0.012 mol) in dry acetone (30 mL), and stirred at room temperature until the reaction was judged complete by TLC using EtOAc-n-hexane (4:6). After completion of the reaction, the solvent was evaporated under reduced pressure and the product was subjected to column chromatography to give titled compound **5**.

*1-(Ethoxymethyl)-1H-benzo[d]imidazole-2(3H)-thione***(5)** Yield (method A: 65%), white crystals, mp 165 °C, TLC, R_f_ 0.88 (1:1 EtOAc-n-hexane); IR (KBr, cm^−1^): 3450 (NH), 1458 (C=S); ^1^H NMR (DMSO-d_6_, 300 MHz): δ 1.06 (t, 3H, J = 6.9 Hz, CH_3_), 3.38–3.57 (m, 2H, OCH_2_), 5.66 (s, 2H, SCH_2_), 7.20 (d, 3H, ArH), 7.37 (m, 1H, ArH), 12.88 (s, 1H, NH); ^13^C NMR (DMSO-d_6_, 125 MHz): δ 14.8 (CH_3_), 63.9 (OCH_2_), 27.1 (SCH_2_), 109.9 (C-4), 109.7 (C-7), 122.5 (C-6), 123.3 (C-5), 130.7 (C-8), 132.2 (C-9), 169.6 (C=S); EIMS: *m*/*z* (%) = 208 (100), 179 (41), 150 (25), 118 (11), 78 (58); ESIMS: Calcd for C_10_H_13_N_2_OS (M^+^+H), *m*/*z* 209.0748. Found: 209.0743.

### 3.2. Urease Expression and Purification

The bacterial DNA was extracted using a DNA extraction kit (Qiagene, Hilden, Germany). The Urease C gene was amplified by PCR using specific primers to amplify the gene according to Bickley [37] (5′TGGTAGAAAACGCTTTAGTA-3′) as the reverse primer with a restriction site of *Eco*R1 and (5′AAACGCCCACACCCACATCTATCA-3′) as the forward primer with a restriction site of *Eco*R I. The amplified PCR product was then qualitatively analyzed on 1% agarose gel. The PCR product was recovered using the Thermo Scientific gel extraction kit (Thermo Scientific, Waltham, MA, USA), and the purified PCR product was used for cloning and expression purposes. The expected size of the target fragment was 360 bp.

#### 3.2.1. Cloning and Transmission of Purified PCR Product

The purified PCR product was ligated into the pGEM–T Easy cloning vector (Promega, Madison, WI, USA) and the ligation reactions were transformed into the competent *E. coli* DH-5-α according to the kit procedures. Plasmid DNA was isolated from the white recombinant cells using a Miniprep plasmid extraction kit (Thermo Scientific, Waltham, MA, USA).

#### 3.2.2. Insert Released of Urease *C gene* from the Recombinant Plasmid

The purified DNA plasmid was subjected to restriction digestion using a fast digest *EcoR1* restriction enzyme, and the reaction was performed in 20 μL reaction volume with recombinant units of enzyme and appropriate buffers at 37 °C for 5 min [the digestion reaction consists of 2 µL plasmid DNA (100 ng), 2 µL of enzyme buffer, 1 µL of restriction enzyme *EcoR1* (50 U) and the volume was made up to 20 μL with nuclease-free water. The digested DNA was separated on 1% agarose gel to confirm the release of the insert, and the released gene insert was eluted using a gel extraction kit (Qiagene, Hilden, Germany).

#### 3.2.3. Sub-Cloning of the Urease Gene into Expression Vector

Eluted DNA insert 1 µL (3 ng) was ligated with the pH6HTN His6HaloTag T7 expression vector 1 µ (1 ng/μ) (Promega, Madison, WI, USA) after being digested by the same restriction enzyme. The ligation mixture was incubated at 4 °C overnight and the reaction was transformed into the *E. coli* BL21 and plated on the LB/ampicillin/IPTG/X-gal plate, according to Sambrook [45].

#### 3.2.4. Recombinant Urease C Enzyme Extraction and Purification

After 24 h of incubation, the cells were harvested by centrifuge at 12,000 rpm for 10 min at 4 °C; the pelleted cells were flooded with 2 mL of phosphate buffer (buffer Q, pH 7.5). The suspended cells were collected in a centrifuge tube and the previous step was repeated several times. The suspension was sonicated on ice for 5 min, and then centrifuged at 10,000 rpm for 30 min, and the supernatant was removed with a Pasteur pipette and subjected to purification by the FPLC system.

#### 3.2.5. Recombinant Urease Concentration and Specific Activity Determination

Determination of protein content in the fractions was carried out according to the Bradford [46] method with bovine serum albumin as a standard and the urease specific activities were estimated according to the Weather [47] method, while the purity of the obtained urease enzyme was performed by Sodium Dodecyl Sulfate–Polyacrylamide Gel Electrophoresis (SDS–PAGE) according to Sambrook [48].

#### 3.2.6. Urease Assay and Inhibition on the Recombinant Urease C

Reaction mixtures comprising 25 μL of urease enzymes solution and 55 μL of sodium phosphate buffers containing 100 mM urea were incubated with 5 μL of tested compounds above (0.5 mM concentration) at 30 °C for 15 min in 96-well plates. Urease activity was determined by measuring the ammonia production using the indophenol method as described by Weatherburn [47]. Briefly, 45 μL of each phenol reagent (1% *w*/*v* phenol and 0.005% *w*/*v* sodium nitroprussside) and 70 μL of alkali reagent (0.5% *w*/*v* NaOH and 0.1% active chloride NaOCl) were added to each well. The absorbance at 630 nm was measured after 50 min using a microplate reader (SPECTRO star Nano, Ortenberg, Germany). All reactions were performed in triplicate in a final volume of 200 μL. The results (change in absorbance per min) were processed by using MARS Data Analysis Version 2.41, software (SPECTRO star Nano, Ortenberg, Germany).

The assays were performed at pH 6.8. The percentage of inhibitions were calculated from the formula 100 − (OD_test well_/OD_control_) × 100. Thiourea was used as the standard inhibitor of Urease.

The concentrations of the test compounds that inhibited the hydrolysis of substrates by 50% (IC_50_) were determined by monitoring the effect of various concentrations of these compounds in the assays on the inhibition values. The IC_50_ values were then calculated using Graph Pad Prism 6 software.

### 3.3. Docking Studies

Docking studies were carried out for the antiviral compounds against *Helicobacter pylori* urease (PDB ID: 1E9Y, resolution: 3.00 Å) using MOE14.0 software, comparing the co-crystallized ligand [49,50,51,52,53,54] (acetohydroxamic acid, HAE), as shown in the Supplementary File.

### 3.4. Toxicity Studies

The in silico toxicity profiles were calculated for compounds **2** and **5** using Discovery studio 4.0 [55,56,57,58], as shown in the Supplementary File.

### 3.5. ADMET Analysis

Discovery studio 4.0 was operated [59,60,61] (method part in the Supplementary File).

### 3.6. In Vitro Cytotoxicity Assay

The active compounds were selected and subjected to cytotoxicity evaluation using the neutral red assay. Cytotoxic assays were performed according to Borenfreund and Puerner [62].

## 4. Conclusions

In conclusion, following the general features of urease inhibitors, compounds **2** and **5** were designed and synthesized through the alkylation of benzimidazole 2-thione derivatives. Both in vitro inhibition kinetics against *H. pylori* ureases and in silico molecular docking studies have been shown by both compounds. In silico ADMET and toxicity investigations indicated the general safety and drug-likeness of both compounds. The safety of the compounds was confirmed by an in vitro cytotoxicity assay against fibroblast cells. Compounds **2** and **5** could be effective agents against several serious pathogenic bacteria such as *Helicobacter pylori*, *Streptococcus salivarius*, and *Klebsiella pneumoniae* as well as several bacteria that infect plants. Further in vivo and preclinical studies would help in providing further insights into the pharmacological properties of these leading compounds.

## Figures and Tables

**Table 1:** Inhibition of *H. Pylori*, and *J. Bean* Ureases by compounds **2** and **5**.

Compound	*H. pylori* Urease				*J. bean* Urease			
	IC_50_ mM	Vmax µmol/min	Km (mM)	Type of Inhibition	IC_50_ mM	Vmax µmol/min	Km (mM)	Type of Inhibition
**2**	0.11 ± 0.048	1.54 ± 0.057	0.04 ± 0.064	NC	0.26 ± 0.008	1.53 ± 0.005	0.01 ± 0.008	NC
**5**	0.01 ± 0.004	1.52 ± 0.024	0.04 ± 0.016	UC	0.29 ± 0.018	1.60 ± 0.04	0.01 ± 0.003	NC

NC = non-competitive UC = uncompetitive = V_max_ = maximal velocity K_m =_ Michalis -Menten consta.

**Table 2:** The computed values of ∆G of the synthesized compounds and the co-crystallized ligand (acetohydroxamic acid, **HAE**) against *Helicobacter pylori* urease.

Compound	∆G (Kcal. Mol^−1^)
**2**	−13.89
**5**	−17.20
**HAE**	−7.78

**Table 3:** Predicted ADMET indices for **2**, **5** and **HAE**.

Comp.	BBB Level ^a^	Solubility Level ^b^	Absorption Level ^c^	CYP2D6 Prediction ^d^	PPB Prediction ^e^
**2**	2	3	0	false	false
**5**	1	3	0	false	true
**HAE**	4	5	1	false	false

^a^ BBB level, blood-brain barrier level, 0 = very high, 1 = high, 2 = medium, 3 = low, 4 = very low. ^b^ Solubility level, 1 = very low, 2 = low, 3 = good, 4 = optimal. ^c^ Absorption level, 0 = good, 1 = moderate, 2 = poor, 3 = very poor. ^d^ CYP2D6, cytochrome P2D6, TRUE = inhibitor, FALSE = non-inhibitor. ^e^ PBB, plasma protein binding, FALSE means less than 90%, TRUE means more than 90%.

**Table 4:** In silico toxicity properties of compounds **2**, **5**, and **HAE**.

Comp.	Carcinogenic Potency TD50 Rat ^a^	Rat Maximum Tolerated Dose (Feed) ^b^	Rat Oral LD_50_^b^	Rat Chronic LOAEL ^b^	Skin Irritancy	Ocular Irritancy
**2**	74.796	0.115	0.271	0.069	Irritant	Irritant
**5**	127.982	0.072	0.314	0.034	Irritant	Irritant
**HAE**	82.223	0.135	1.090	0.423	Non-Irritant	Irritant

^a^ mg/kg body weight/day, ^b^ Unit: g/kg body weight.

**Table 5:** Cytotoxicity of various concentrations of compounds **2** and **5** on the fibroblast cells.

Conc. (µM)	% Inhibition	
	Compd 2	Compd 5
0.123	82.5	53.55
0.064	80.6	52.07
0.031	49.5	26.22
0.015	49.35	4.6
0.007	44.03	−15.9
0.003	40.42	−28.81
0.009	20.01	−61.25

## Data Availability

Data is contained within the article.

## Supplementary Materials

The following are available online, NMR and Ms spectra of the synthesized compounds, Detailed toxicity report, in addition to the detailed in silico method.
