# Supplementary material for: Expression, Purification, and Comparative Inhibition of Helicobacter pylori Urease by Regio-Selectively Alkylated Benzimidazole 2-Thione Derivatives
Source: Molecules. 2022 Jan 27;27(3):865. doi: 10.3390/molecules27030865 (PMC8838460; doi:10.3390/molecules27030865)
Supplement: Supplementary file 1 [file molecules-27-00865-s001.zip › molecules-1522720-supplementary.pdf]

Supporting data

Expression, purification, and comparative inhibition of *Helicobacter pylori* urease by regio-selectively alkylated benzimidazole 2-thione derivatives

Salih Osman Mohammed[1], El Sayed H. El Ashry[2], Asaad Khalid[1,3], Mohamed R. Amer[2], Ahmed M. Metawely[4,5], Ibrahim H. Eissa[6], Eslam B. Elkaeed[7], El Shobaky Ahmed[8], Elsayed Elsayed Hafez[9]

[1]Medicinal and Aromatic Plants and Traditional Medicine Research Institute, National Center for Research, Khartoum-11111, Sudan.

[2]Chemistry Department, Faculty of Science, Alexandria University, Alexandria, Egypt.

[3] Substance Abuse and Toxicology Research Center, Jazan University, Jazan, Saudi Arabia.

[4]Pharmacognosy and Medicinal Plants Department, Faculty of Pharmacy (Boys), Al-Azhar University, Cairo, Egypt

[5]Biopharmaceutical Products Research Department, Genetic Engineering and Biotechnology Research Institute, City of Scientific Research and Technological Applications (SRTA-City), Alexandria, Egypt

[6]Pharmaceutical Medicinal Chemistry & Drug Design Department, Faculty of Pharmacy (Boys), Al-Azhar University, Cairo11884, Egypt

[7]Department of Pharmaceutical Sciences, College of Pharmacy, AlMaarefa University, Ad Diriyah 13713, Riyadh, Saudi Arabia

[8]Botany Department, Faculty of Science, Mansoura University, Egypt.

[9] City of Scientific Research and Technological Applications, ARADI, 21934, Alexandria, Egypt

Content

**Detailed Method of *in silico* studies**	**1**
**NMR Data**	**2**
**Toxicity report of compounds**	**3**

Detailed Method of *in silico* studies

Docking studies

The crystal structure of the target protein was obtained from Protein Data Bank. The docking investigation was accomplished using MOE2014 software. At first, the crystal structure of the protein was prepared by removing water molecules. Only one chain was retained besides the co-crystallized ligand (GWS). Then, the selected chain was protonated and subjected to minimization of energy process. Next, the active site of the target protein was defined.

Structures of the tested compounds and the co-crystallized ligand were drawn using ChemBioDraw Ultra 14.0 and saved as MDL-SD format. Such file was opened using MOE to display the 3D structures which were protonated and subjected to energy minimization. Formerly, validation of the docking process was performed by docking the co-crystallized ligand against the isolated pocket of active site. The produced RMSD value indicated the validity of process. Finally, docking of the tested compounds was done through the dock option inserted in compute window. For each docked molecule, 30 docked poses were produced using ASE for scoring function and force field for refinement. The results of the docking process were then visualized using Discovery Studio 4.0 software.

ADMET studies

ADMET descriptors (absorption, distribution, metabolism, excretion and toxicity) of the compounds were determined using Discovery studio 4.0. At first, the CHARMM force field was applied then the tested compounds were prepared and minimized according to the preparation of small molecule protocol. Then ADMET descriptors protocol was applied to carry out these studies.

Toxicity studies

The toxicity parameters of the tested compounds were calculated using Discovery studio 4.0. Indinavir was used as a reference drug. At first, the CHARMM force field was applied then the compounds were prepared and minimized according to the preparation of small molecule protocol. Then different parameters were calculated from the toxicity prediction (extensible) protocol.

File Name : d:\mswin\data\mra-31.mss
Creation Date/Time : 23/07/07 at 14:41:58
File Type : Lo-Res Mass Data (Centroid)
File Source : Acquired on MASPEC system [msw/A091]
File Title : EI
Operator : Barkat Ali
Instrument : MAT312

SCAN GRAPH. Flagging=M/z. Filter=[Int:1%.].
Scan 6-4:07. Entries=27. 100% Int.=72754.

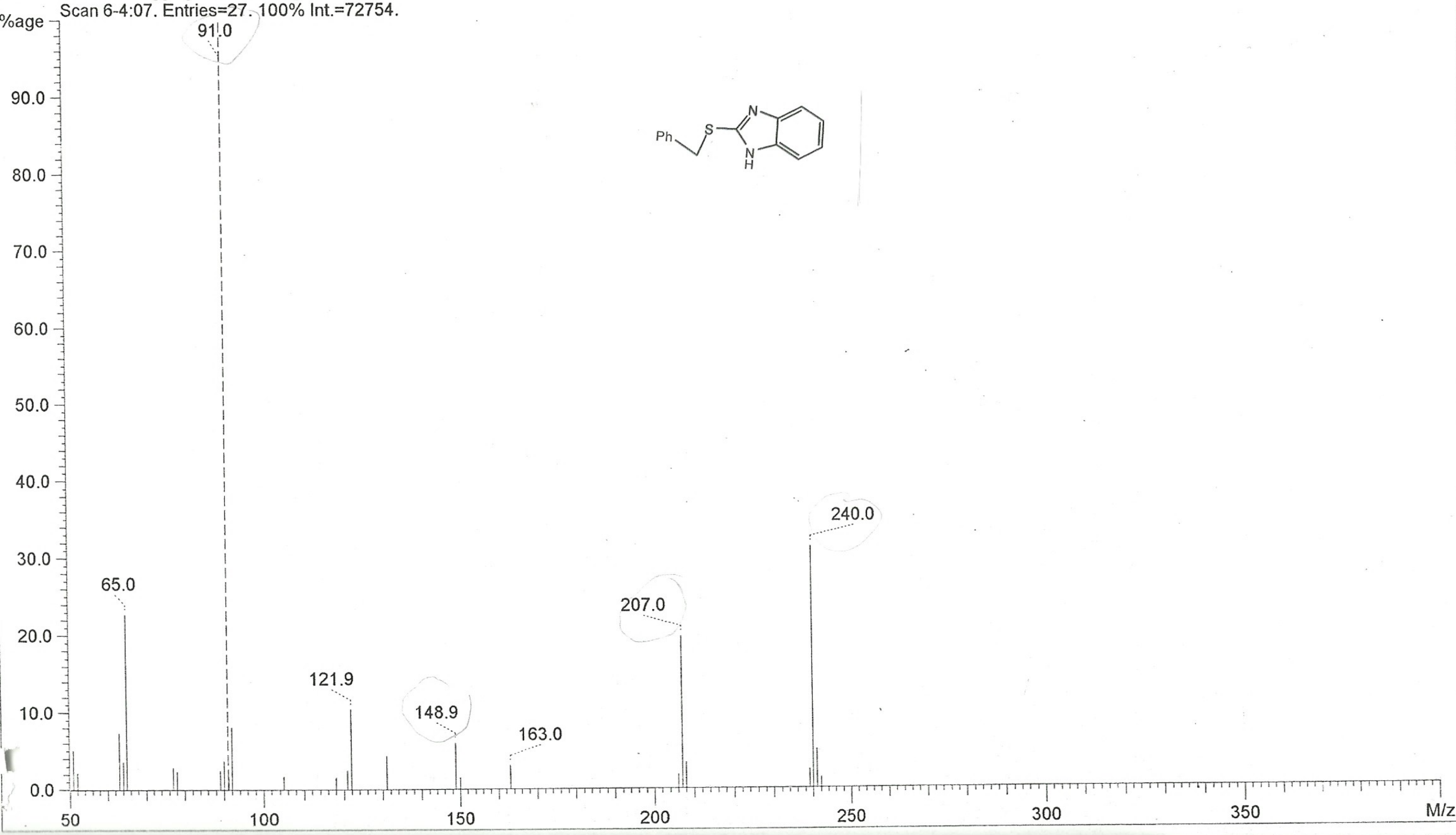

M.Amer/Dr.EL Ashry
Sample: MRA-45

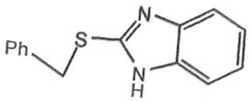

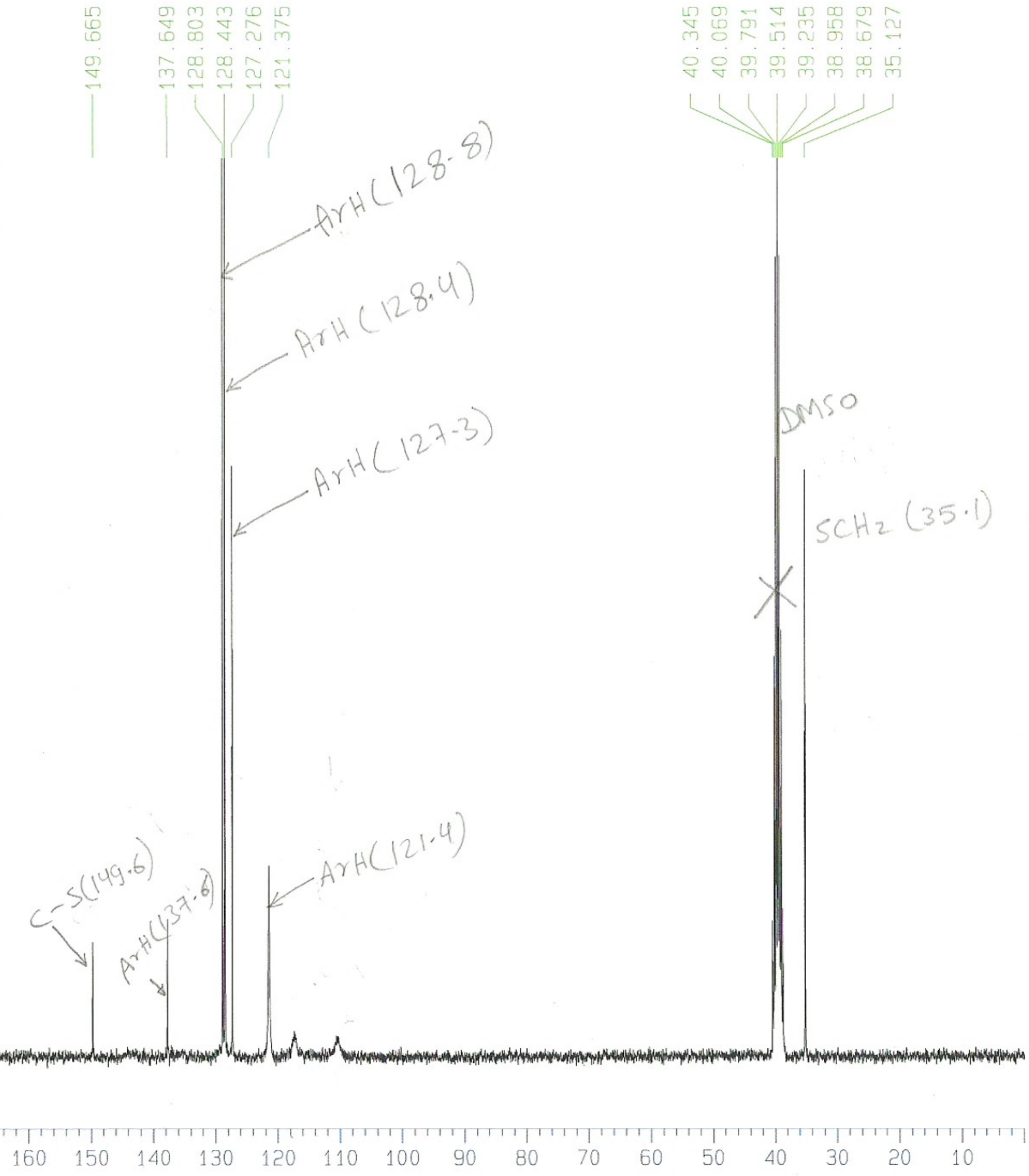

AVANCE [illegible] LAB. No. [illegible]

Current Data Parameters
NAME jan17
EXPNO 4
PROCNO 1

F2 - Acquisition Parameters
Date_ 20080117
Time 7.15
INSTRUM spect
PROBHD 5 mm DUL 13C-1
PULPROG zgpg
TD 66560
SOLVENT DMSO
NS 2476
DS 4
SWH 17985.611 Hz
FIDRES 0.270217 Hz
AQ 1.8504180 sec
RG 32768
DW 27.800 usec
DE 6.00 usec
TE 300.1 K
D1 1.50000000 sec
d11 0.03000000 sec
DELTA 1.39999998 sec
MCREST 0.00000000 sec
MCWRK 0.01500000 sec

======== CHANNEL f1 ========
NUC1 13C
P1 6.50 usec
PL1 0.00 dB
SFO1 75.4764278 MHz

======== CHANNEL f2 ========
CPDPRG2 waltz16
NUC2 1H
PCPD2 85.00 usec
PL2 0.00 dB
PL12 19.00 dB
PL13 19.00 dB
SFO2 300.1315007 MHz

F2 - Processing parameters
SI 32768
SF 75.4677867 MHz
WDW EM
SSB 0
LB 1.50 Hz
GB 0
PC 1.20

1D NMR plot parameters
CX 20.00 cm
CY 15.00 cm
F1P 231.109 ppm
F1 17441.30 Hz
F2P -0.068 ppm
F2 -5.13 Hz
PPMCM 11.55886 ppm/cm
HZCM 872.32141 Hz/cm

M.RAMDAN/DR.EL ASHRY/MRA-31/DMSO
1H NMR

AVANCE AV 300
LAB. No. 115

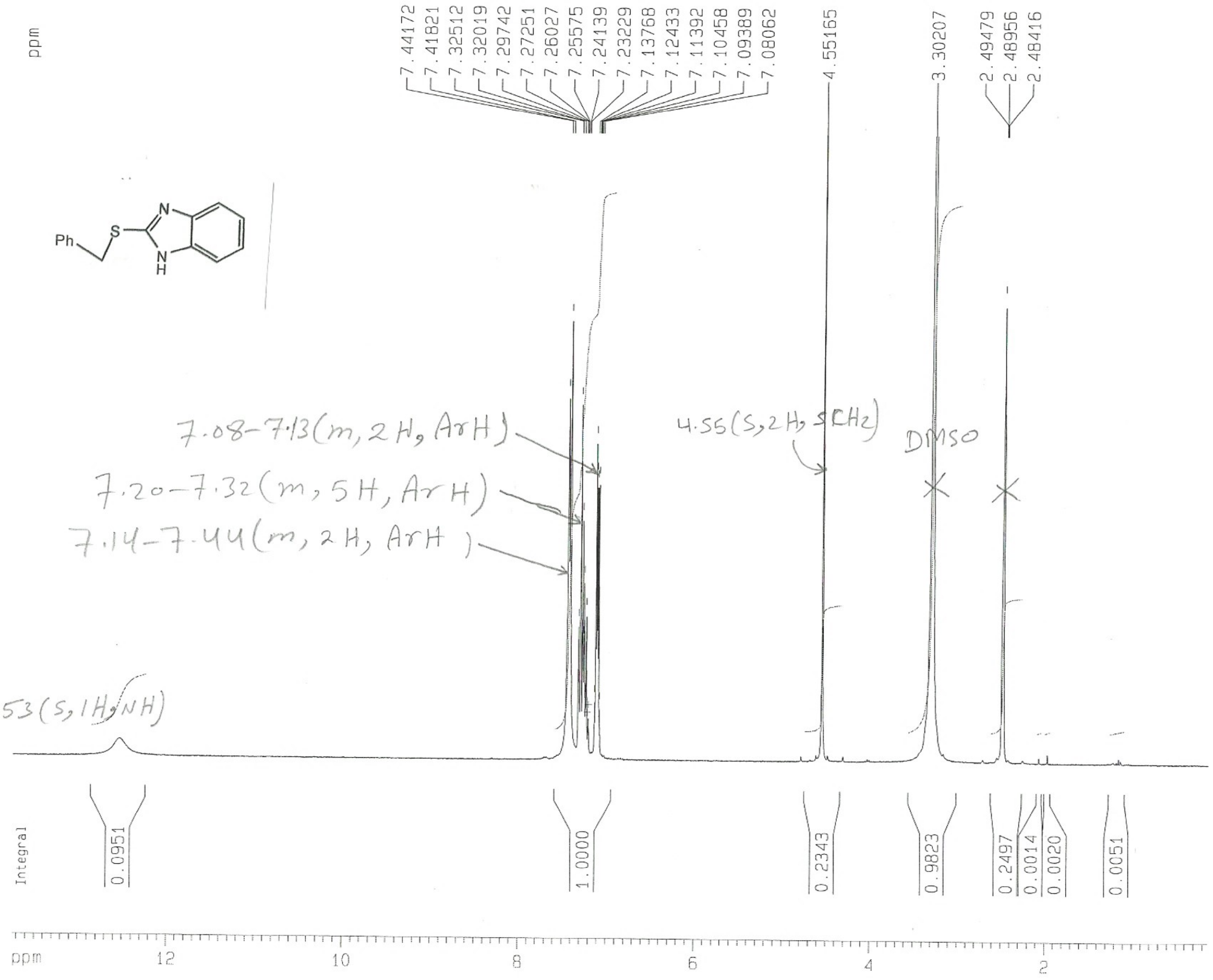

Current Data Parameters
NAME july25
EXPNO 8
PROCNO 1

F2 - Acquisition Parameters
Date_ 20070725
Time 14.38
INSTRUM spect
PROBHD 5 mm DUL 13C-1
PULPROG zg30
TD 32768
SOLVENT MeOD
NS 128
DS 0
SWH 5995.204 Hz
FIDRES 0.182959 Hz
AQ 2.7329011 sec
RG 1024
DW 83.400 usec
DE 10.00 usec
TE 0.0 K
D1 1.00000000 sec
MCREST 0.00000000 sec
MCWRK 0.01500000 sec

======== CHANNEL f1 ========
NUC1 1H
P1 10.20 usec
PL1 -3.00 dB
SFO1 300.1324010 MHz

F2 - Processing parameters
SI 16384
SF 300.1300045 MHz
WDW EM
SSB 0
LB 0.30 Hz
GB 0
PC 1.00

1D NMR plot parameters
CX 20.00 cm
CY 20.00 cm
F1P 13.749 ppm
F1 4126.60 Hz
F2P 0.139 ppm
F2 41.69 Hz
PPMCM 0.68052 ppm/cm
HZCM 204.24544 Hz/cm

OK

RAMADAN/DR.EL ASHRY/MRA.81B/DMSO

AVANCE AV 300
LAB. No. 115

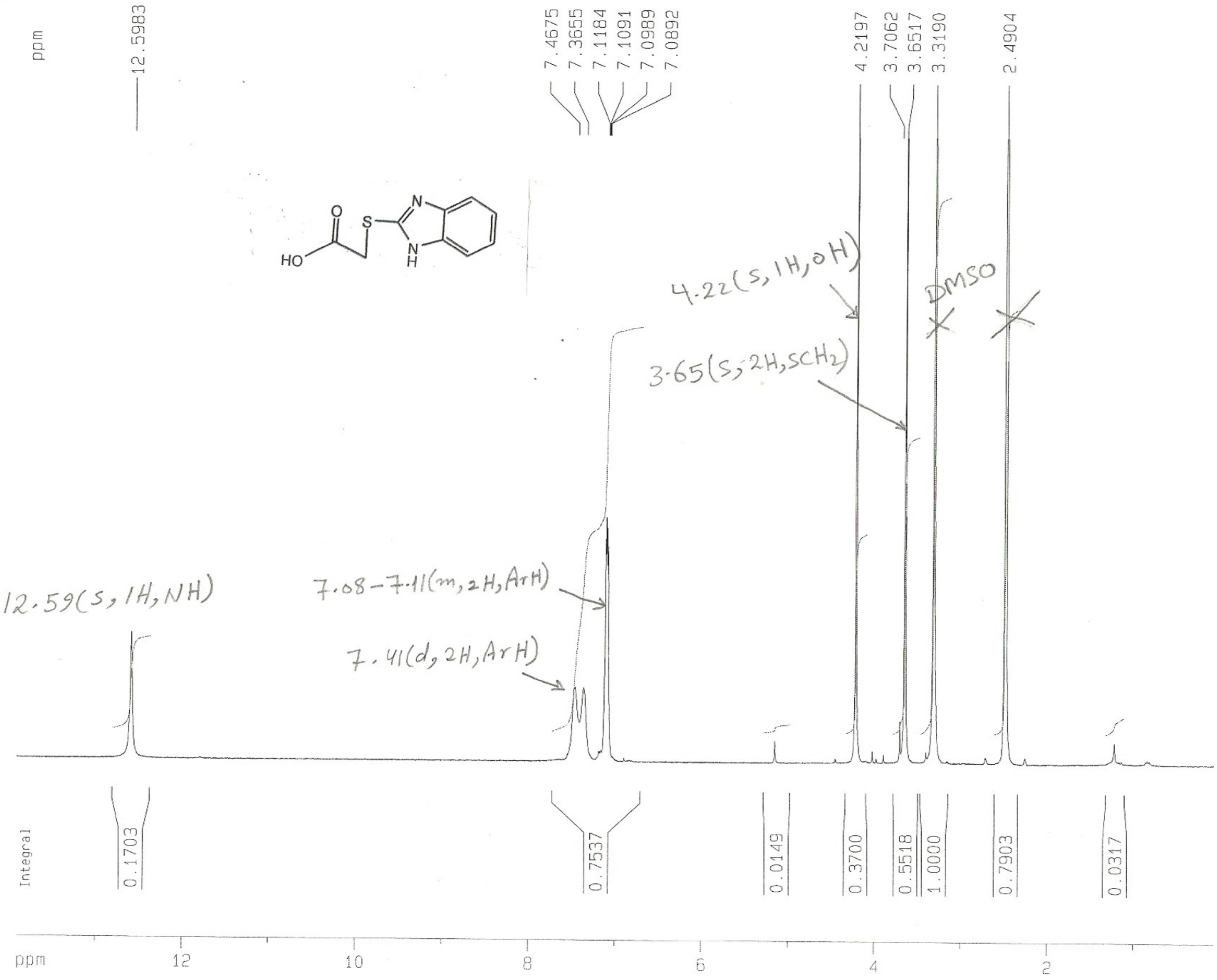

Current Data Parameters

NAME	nov10
EXPNO	12
PROCNO	1

F2 - Acquisition Parameters

Date_	20071110
Time	12.21
INSTRUM	spect
PROBHD	5 mm DUL 13C-1
PULPROG	zg30
TD	32768
SOLVENT	DMSO
NS	128
DS	0
SWH	5995.204 Hz
FIDRES	0.182959 Hz
AQ	2.7329011 sec
RG	1290.2
DW	83.400 usec
DE	10.00 usec
TE	0.0 K
D1	1.00000000 sec
MCREST	0.00000000 sec
MCWRK	0.01500000 sec

======== CHANNEL f1 ========

NUC1	1H
P1	9.80 usec
PL1	-3.00 dB
SFO1	300.1324010 MHz

F2 - Processing parameters

SI	16384
SF	300.1300042 MHz
WDW	EM
SSB	0
LB	0.30 Hz
GB	0
PC	1.00

1D NMR plot parameters

CX	20.00 cm
CY	45.00 cm
F1P	13.915 ppm
F1	4176.39 Hz
F2P	0.068 ppm
F2	20.28 Hz
PPMCM	0.69238 ppm/cm
HZCM	207.80550 Hz/cm

(2)

AAMER/DR.EL ASHRY/MRA.81
CARBON13C

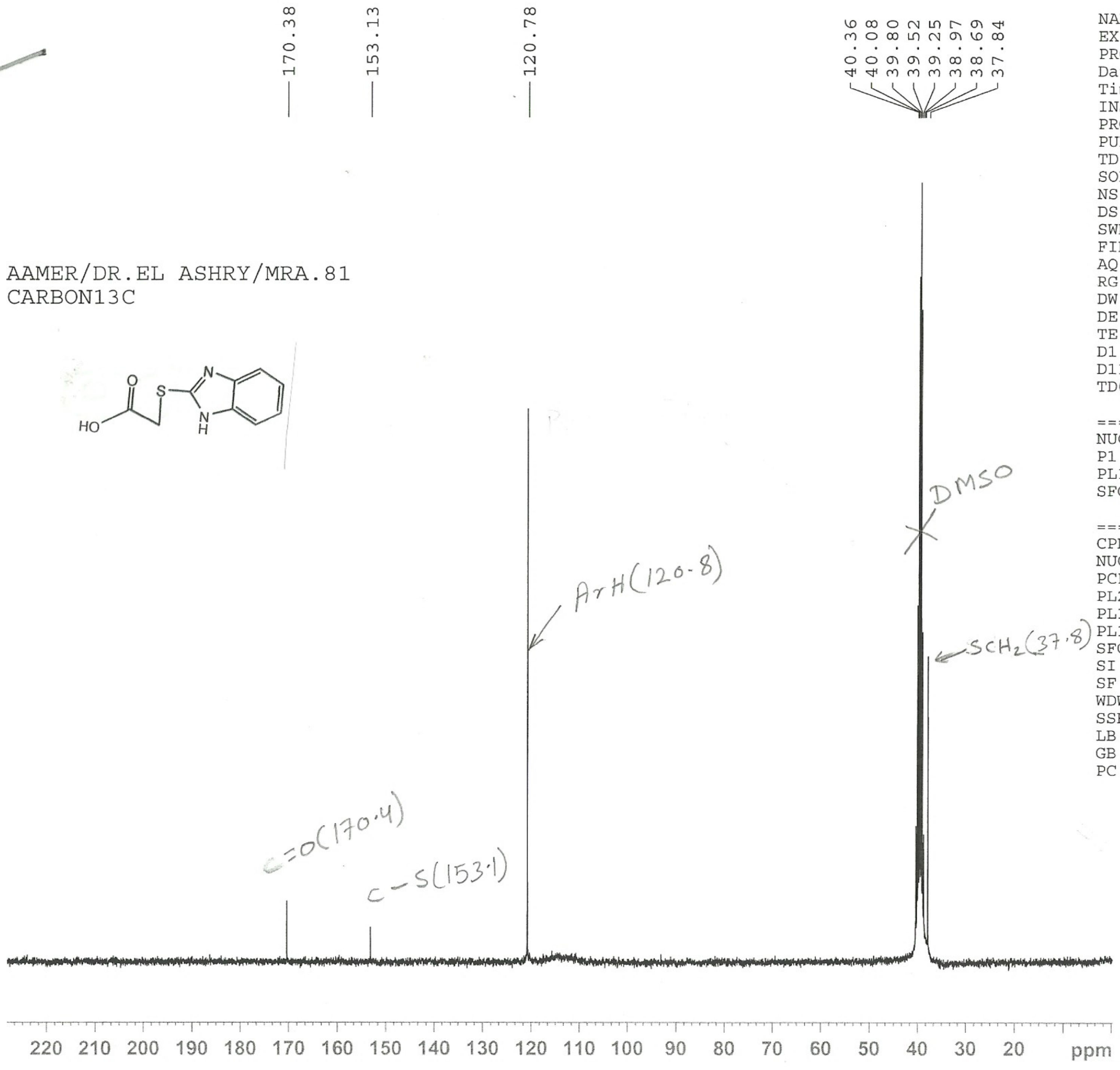

NAME	jan19
EXPNO	15
PROCNO	1
Date_	20090119
Time	15.42
INSTRUM	spect
PROBHD	5 mm DUL 13C-1
PULPROG	zgpg
TD	32768
SOLVENT	DMSO
NS	16384
DS	2
SWH	17985.611 Hz
FIDRES	0.548877 Hz
AQ	0.9110004 sec
RG	32768
DW	27.800 usec
DE	6.50 usec
TE	300.0 K
D1	1.50000000 sec
D11	0.03000000 sec
TD0	16

======== CHANNEL f1 ========

NUC1	13C
P1	11.50 usec
PL1	-2.00 dB
SFO1	75.4765033 MHz

======== CHANNEL f2 ========

CPDPRG2	waltz16
NUC2	1H
PCPD2	100.00 usec
PL2	-3.00 dB
PL12	17.00 dB
PL13	20.00 dB
SFO2	300.1315007 MHz
SI	16384
SF	75.4677838 MHz
WDW	EM
SSB	0
LB	1.00 Hz
GB	0
PC	1.40

[illegible] AV 300
LAB. No. 115

MASS SPECTRUM Data File: MRA81 13-FEB- 8 10:0
Sample: HEJ
RT 0'09" FAB(Pos.) GC 1.4c BP: m/z 93.0000 Int. 69.5540 Lu 1.00
Scan# (2)

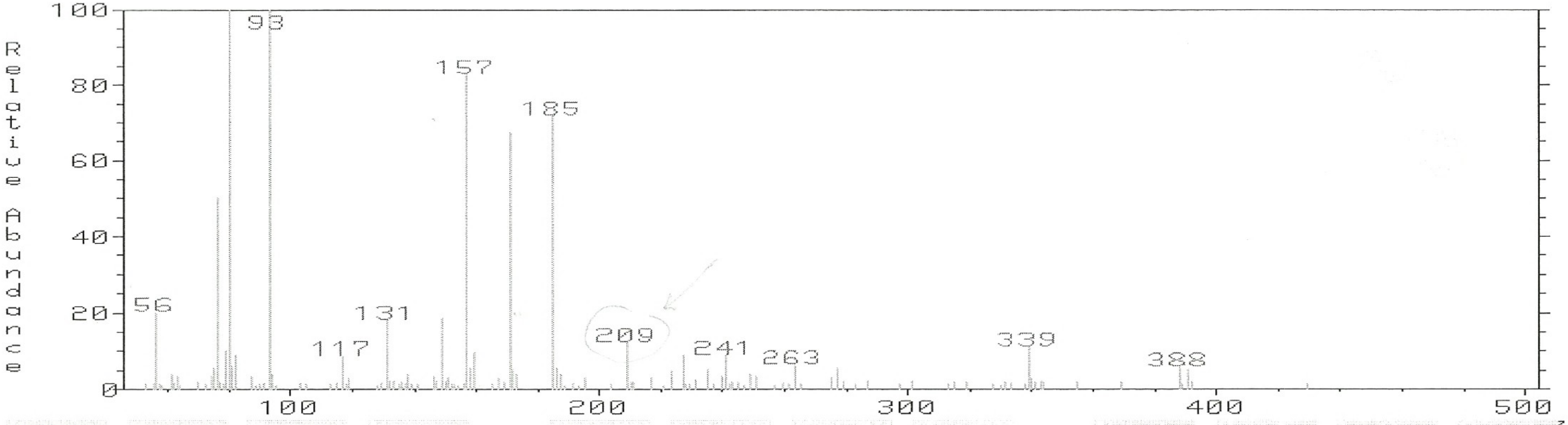

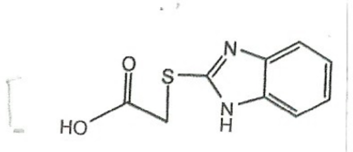

Mwt = 208 + H^+ = 209

1 → 10 acceptable.

Calc

Mass	Relative Intensity	Theoretical Mass	Delta [ppm]	Delta [mmu]	RDB	Composition
		192.0205	-9.2	-1.8	3.0	$C_5 H_8 O_4 N_2 S_1$
		192.0211	-12.6	-2.4	12.0	$C_{13} H_4 O_2$
192.9883	2.9933	192.9885	-1.4	-0.3	8.5	$C_7 H_1 O_5 N_2$
		192.9860	11.6	2.2	12.5	$C_{11} H_1 N_2 S_1$
		192.9859	12.5	2.4	4.0	$C_4 H_3 O_8 N_1$
204.9883	1.9057	204.9885	-1.3	-0.3	9.5	$C_8 H_1 O_5 N_2$
		204.9892	-4.7	-1.0	0.0	$C_2 H_7 O_8 N_1 S_1$
		204.9860	10.9	2.2	13.5	$C_{12} H_1 N_2 S_1$
205.0493	1.4363	205.0501	-4.1	-0.8	7.5	$C_{11} H_9 O_4$
		205.0461	15.6	3.2	3.5	$C_6 H_9 O_6 N_2$
		205.0528	-17.1	-3.5	12.0	$C_{14} H_7 O_1 N_1$
206.0579	1.5262	206.0579	0.2	0.0	7.0	$C_{11} H_{10} O_4$
		206.0606	-12.8	-2.6	11.5	$C_{14} H_8 O_1 N_1$
		206.0613	-16.2	-3.3	2.0	$C_8 H_{14} O_4 S_1$
208.0293	4.6620	208.0306	-6.3	-1.3	7.0	$C_9 H_8 O_2 N_2 S_1$
		208.0280	6.6	1.4	2.5	$C_6 H_{10} O_5 N_1 S_1$
		208.0313	-9.4	-2.0	16.0	$C_{17} H_4$
218.9851	3.4103	218.9863	-5.4	-1.2	1.0	$C_2 H_5 O_{11} N_1$
		218.9838	6.0	1.3	5.0	$C_6 H_5 O_6 N_1 S_1$
		218.9864	-6.2	-1.4	9.5	$C_9 H_3 O_3 N_2 S_1$
230.9851	4.2577	230.9863	-5.1	-1.2	2.0	$C_3 H_5 O_{11} N_1$
		230.9838	5.7	1.3	6.0	$C_7 H_5 O_6 N_1 S_1$
		230.9864	-5.9	-1.4	10.5	$C_{10} H_3 O_3 N_2 S_1$
242.9851	6.2305	242.9863	-4.9	-1.2	3.0	$C_4 H_5 O_{11} N_1$
		242.9838	5.4	1.3	7.0	$C_8 H_5 O_6 N_1 S_1$
		242.9864	-5.6	-1.4	11.5	$C_{11} H_3 O_3 N_2 S_1$
254.9851	2.1774	254.9863	-4.6	-1.2	4.0	$C_5 H_5 O_{11} N_1$
		254.9838	5.2	1.3	8.0	$C_9 H_5 O_6 N_1 S_1$
		254.9864	-5.3	-1.4	12.5	$C_{12} H_3 O_3 N_2 S_1$
266.9881	1.2316	266.9889	-3.0	-0.8	9.5	$C_9 H_3 O_8 N_2$
		266.9896	-5.6	-1.5	0.0	$C_3 H_9 O_{11} N_1 S_1$
		266.9864	6.4	1.7	13.5	$C_{13} H_3 O_3 N_2 S_1$
268.9819	1.3998	268.9810	3.5	0.9	17.5	$C_{16} H_1 O_1 N_2 S_1$
		268.9808	4.1	1.1	9.0	$C_9 H_3 O_9 N_1$
		268.9835	-5.9	-1.6	13.5	$C_{12} H_1 O_6 N_2$
280.9819	3.6877	280.9815	1.5	0.4	0.5	$C_4 H_9 O_{12} S_1$
		280.9810	3.3	0.9	18.5	$C_{17} H_1 O_1 N_2 S_1$
		280.9808	3.9	1.1	10.0	$C_{10} H_3 O_9 N_1$
292.9819	6.5281	292.9815	1.4	0.4	1.5	$C_5 H_9 O_{12} S_1$
		292.9810	3.2	0.9	19.5	$C_{18} H_1 O_1 N_2 S_1$
		292.9808	3.8	1.1	11.0	$C_{11} H_3 O_9 N_1$
304.9819	1.0672	304.9815	1.4	0.4	2.5	$C_6 H_9 O_{12} S_1$
		304.9810	3.0	0.9	20.5	$C_{19} H_1 O_1 N_2 S_1$
		304.9808	3.6	1.1	12.0	$C_{12} H_3 O_9 N_1$

Expected = 208.0306
obtained = 208.0293

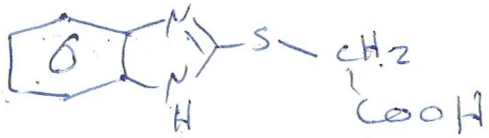

$C_9 H_8 N_2 O_2 S_1$ =

208.0306 = Calc.

208.0293 = found

~~0.0013~~

~~error = 0.0013 / 208.0306 × 10^6 = 6.2~~

RAMADAN/DR.EL ASHRY/MRA.38/DMSO

c13 {BB}

AVANCE AV 300
LAB. No. 115

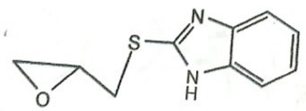

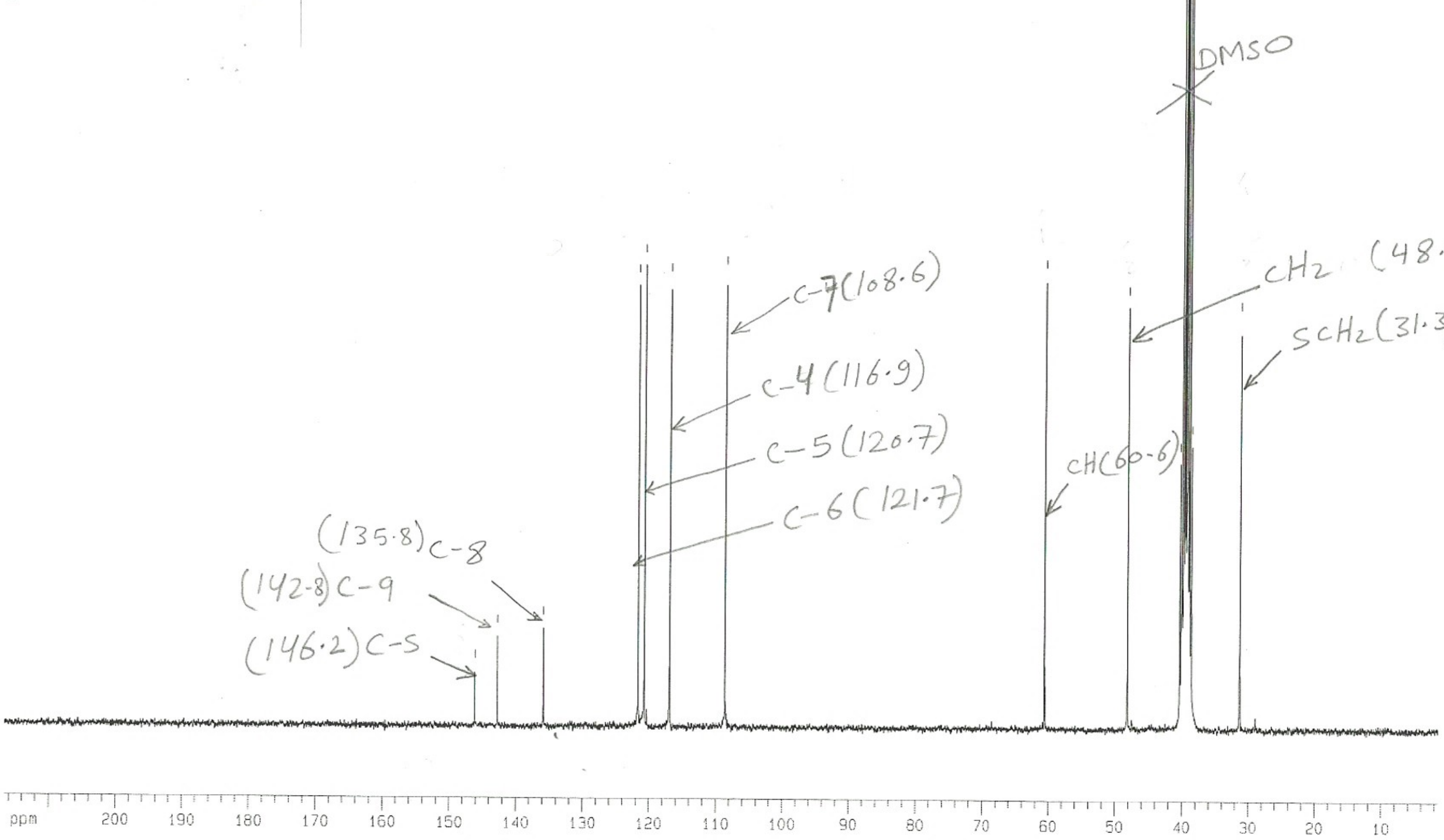

Current Data Parameters

NAME	oct08
EXPNO	10
PROCNO	1

F2 - Acquisition Parameters

Date_	20071008
Time	12.36
INSTRUM	spect
PROBHD	5 mm DUL 13C-1
PULPROG	zgpg30
TD	65536
SOLVENT	DMSO
NS	26820
DS	2
SWH	18518.518 Hz
FIDRES	0.282570 Hz
AQ	1.7695220 sec
RG	16384
DW	27.000 usec
DE	10.00 usec
TE	0.0 K
D1	1.00000000 sec
d11	0.03000000 sec
DELTA	0.89999998 sec
MCREST	0.00000000 sec
MCWRK	0.01500000 sec

======== CHANNEL f1 ========

NUC1	13C
P1	14.00 usec
PL1	-6.00 dB
SFO1	75.4764278 MHz

======== CHANNEL f2 ========

CPDPRG2	waltz16
NUC2	1H
PCPD2	100.00 usec
PL2	-3.00 dB
PL12	20.00 dB
PL13	20.00 dB
SFO2	300.1312005 MHz

F2 - Processing parameters

SI	32768
SF	75.4677864 MHz
WDW	EM
SSB	0
LB	1.50 Hz
GB	0
PC	1.40

1D NMR plot parameters

CX	20.00 cm
CY	26.00 cm
F1P	216.645 ppm
F1	16349.69 Hz
F2P	1.470 ppm
F2	110.94 Hz
PPMCM	10.75873 ppm/cm
HZCM	811.93719 Hz/cm

M.RAMADAN/DR.EL ASHRY/MRA.19'/DMSO

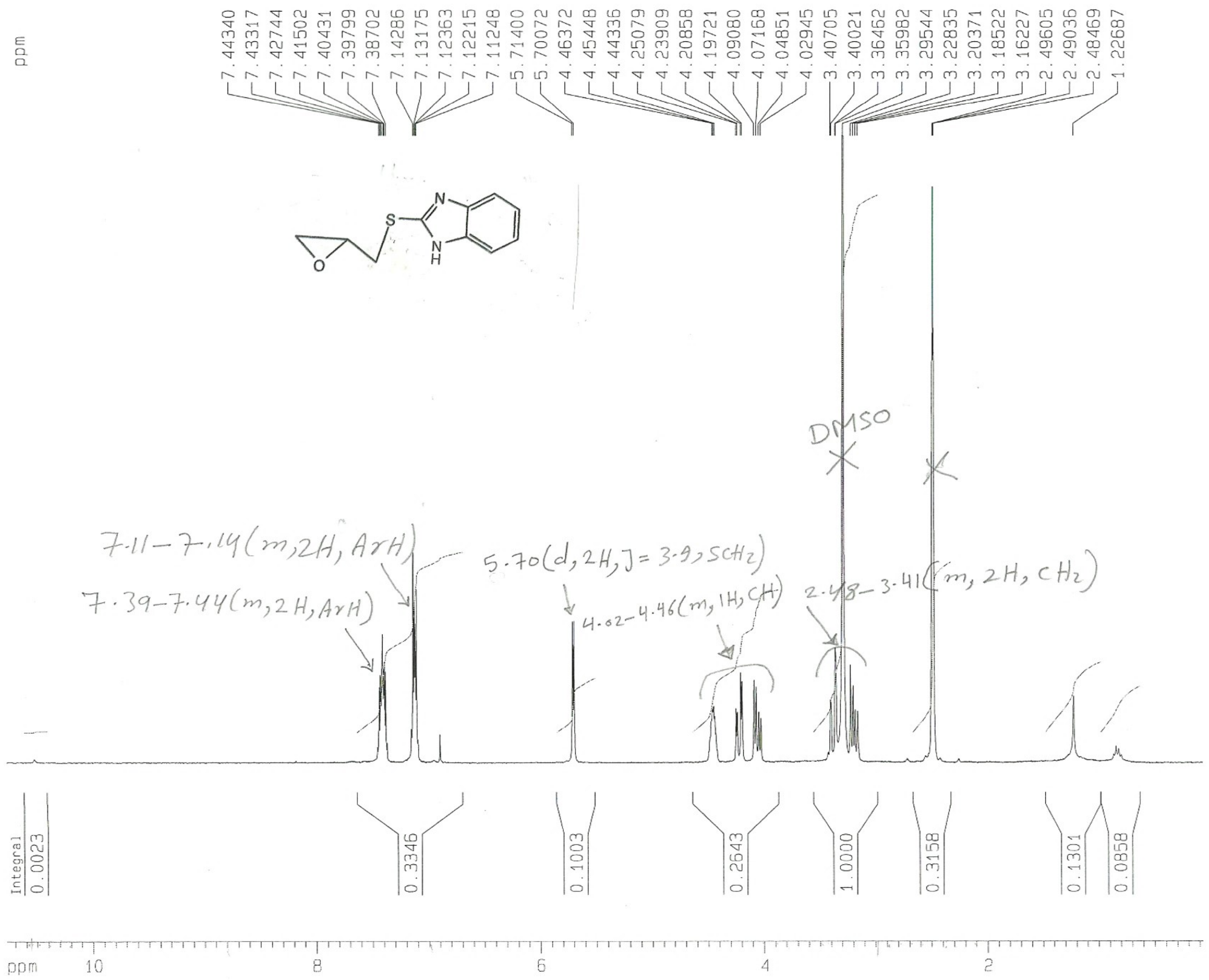

Current Data Parameters
NAME july26
EXPNO 1
PROCNO 1

F2 - Acquisition Parameters
Date_ 20070726
Time 9.24
INSTRUM spect
PROBHD 5 mm DUL 13C-1
PULPROG zg30
TD 32768
SOLVENT DMSO
NS 128
DS 0
SWH 5995.204 Hz
FIDRES 0.182959 Hz
AQ 2.7329011 sec
RG 1625.5
DW 83.400 usec
DE 10.00 usec
TE 0.0 K
D1 1.00000000 sec
MCREST 0.00000000 sec
MCWRK 0.01500000 sec

======== CHANNEL f1 ========
NUC1 1H
P1 10.20 usec
PL1 -3.00 dB
SFO1 300.1324010 MHz

F2 - Processing parameters
SI 16384
SF 300.1300042 MHz
WDW EM
SSB 0
LB 0.30 Hz
GB 0
PC 1.00

1D NMR plot parameters
CX 20.00 cm
CY 80.00 cm
F1P 10.779 ppm
F1 3235.22 Hz
F2P 0.071 ppm
F2 21.36 Hz
PPMCM 0.53541 ppm/cm
HZCM 160.69310 Hz/cm

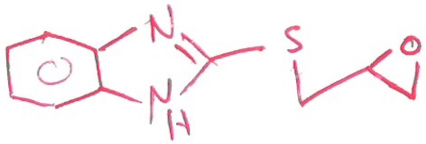

Mass	Relative Intensity	Theoretical Mass	Delta [ppm]	Delta [mmu]	Composition
		190.0590	11.5	2.2	$C_6 H_{10} O_5 N_2$
		190.0657	-23.8	-4.5	$C_{14} H_8 N_1$
192.9883	1.3773	192.9885	-1.4	-0.3	$C_7 H_1 O_5 N_2$
		192.9860	11.6	2.2	$C_{11} H_1 N_2 S_1$
		192.9859	12.5	2.4	$C_4 H_3 O_8 N_1$
204.9883	0.5411	204.9885	-1.3	-0.3	$C_8 H_1 O_5 N_2$
		204.9860	10.9	2.2	$C_{12} H_1 N_2 S_1$
		204.9859	11.7	2.4	$C_5 H_3 O_8 N_1$
205.0460	0.9081	205.0461	-0.4	-0.1	$C_6 H_9 O_6 N_2$
		205.0436	11.8	2.4	$C_{10} H_9 O_1 N_2 S_1$
		205.0501	-20.0	-4.1	$C_{11} H_9 O_4$
206.0527	100.0000	206.0539	-5.6	-1.1	$C_6 H_{10} O_6 N_2$
		206.0514	6.6	1.4	$C_{10} H_{10} O_1 N_2 S_1$
		206.0487	19.6	4.0	$C_7 H_{12} O_4 N_1 S_1$
207.0548	12.2911	207.0558	-5.0	-1.0	$C_{13} H_7 O_1 N_2$
		207.0532	7.9	1.6	$C_{10} H_9 O_4 N_1$
		207.0565	-8.3	-1.7	$C_7 H_{13} O_4 N_1 S_1$
208.0474	5.3630	208.0484	-5.0	-1.0	$C_9 H_8 O_4 N_2$
		208.0457	7.9	1.6	$C_6 H_{10} O_7 N_1$
		208.0432	19.9	4.1	$C_{10} H_{10} O_2 N_1 S_1$
209.0528	0.3492	209.0536	-3.6	-0.8	$C_6 H_{11} O_7 N_1$
		209.0510	8.3	1.7	$C_{10} H_{11} O_2 N_1 S_1$
		209.0562	-16.5	-3.4	$C_9 H_9 O_4 N_2$
218.9851	3.4234	218.9863	-5.4	-1.2	$C_2 H_5 O_{11} N_1$
		218.9838	6.0	1.3	$C_6 H_5 O_6 N_1 S_1$
		218.9864	-6.2	-1.4	$C_9 H_3 O_3 N_2 S_1$
230.9851	3.5522	230.9863	-5.1	-1.2	$C_3 H_5 O_{11} N_1$
		230.9838	5.7	1.3	$C_7 H_5 O_6 N_1 S_1$
		230.9864	-5.9	-1.4	$C_{10} H_3 O_3 N_2 S_1$
242.9851	1.4100	242.9863	-4.9	-1.2	$C_4 H_5 O_{11} N_1$
		242.9838	5.4	1.3	$C_8 H_5 O_6 N_1 S_1$
		242.9864	-5.6	-1.4	$C_{11} H_3 O_3 N_2 S_1$
254.9851	0.4070	254.9863	-4.6	-1.2	$C_5 H_5 O_{11} N_1$
		254.9838	5.2	1.3	$C_9 H_5 O_6 N_1 S_1$
		254.9864	-5.3	-1.4	$C_{12} H_3 O_3 N_2 S_1$
266.9859	0.3044	266.9863	-1.3	-0.4	$C_6 H_5 O_{11} N_1$
		266.9864	-2.0	-0.5	$C_{13} H_3 O_3 N_2 S_1$
		266.9838	8.0	2.1	$C_{10} H_5 O_6 N_1 S_1$
268.9819	1.3442	268.9810	3.5	0.9	$C_{16} H_1 O_1 N_2 S_1$
		268.9808	4.1	1.1	$C_9 H_3 O_9 N_1$
		268.9835	-5.9	-1.6	$C_{12} H_1 O_6 N_2$
280.9819	3.0609	280.9810	3.3	0.9	$C_{17} H_1 O_1 N_2 S_1$
		280.9808	3.9	1.1	$C_{10} H_3 O_9 N_1$
		280.9835	-5.6	-1.6	$C_{13} H_1 O_6 N_2$

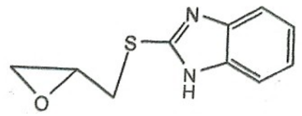

Found = 206.0527

Calc. = 206.0514

$C_{10}H_{10}O_1N_2S_1$

File Name : d:\mswin\data\mra-38.mss
Creation Date/Time : 30/07/07 at 10:16:44
File Type : Lo-Res Mass Data (Centroid)
File Source : Acquired on MASPEC system [msw/A091]
File Title : EI
Operator : Barkat Ali
Instrument : MAT312

SCAN GRAPH. Flagging=M/z. Filter=[Int:1%.].
Scan 14-9:39. Entries=50. 100% Int.=13418.

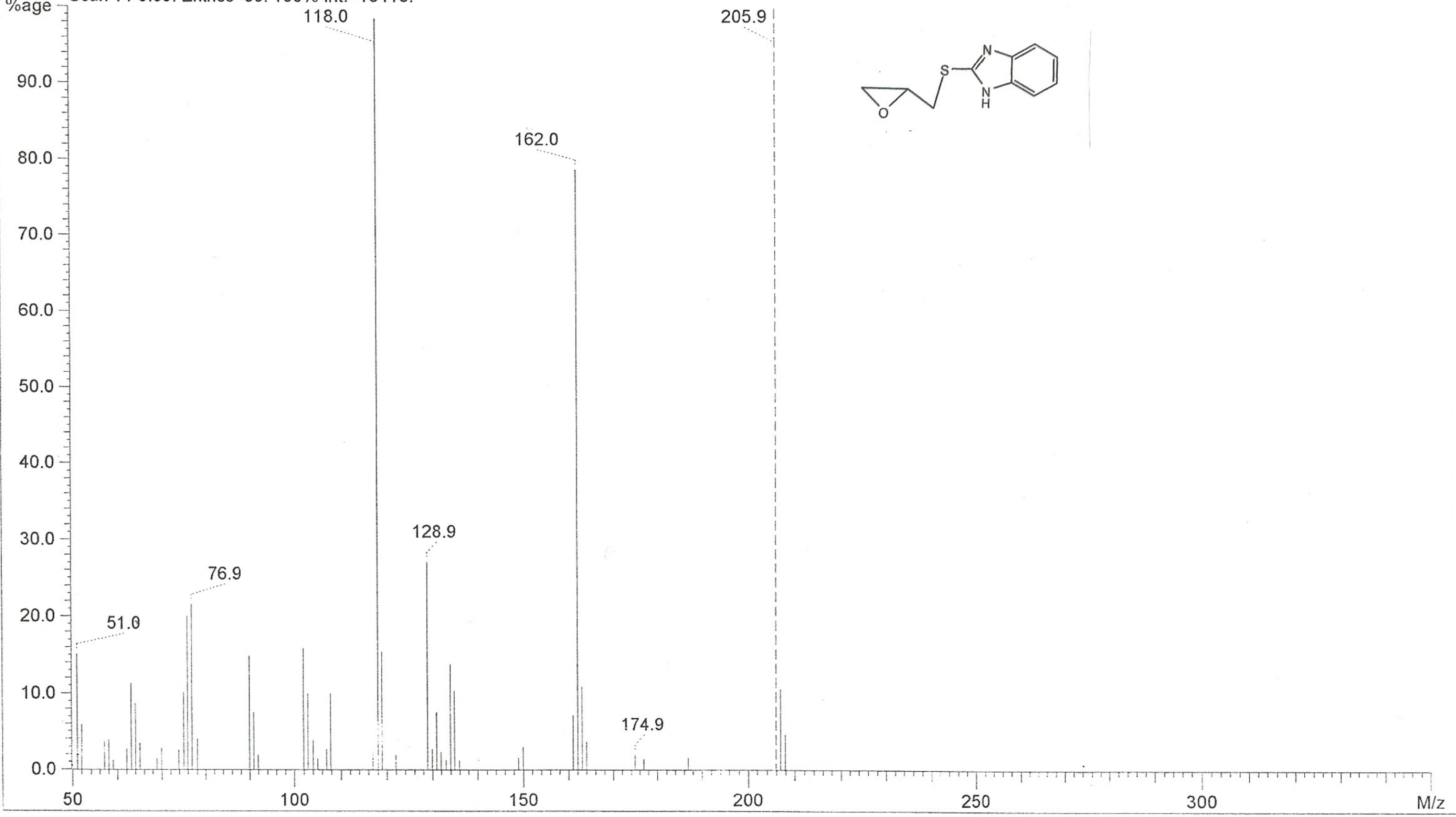

M.RAMADAN/DR.EL.ASHRY/MRA-14

C13 {BB}

AVANCE 400
LAB. No. 117

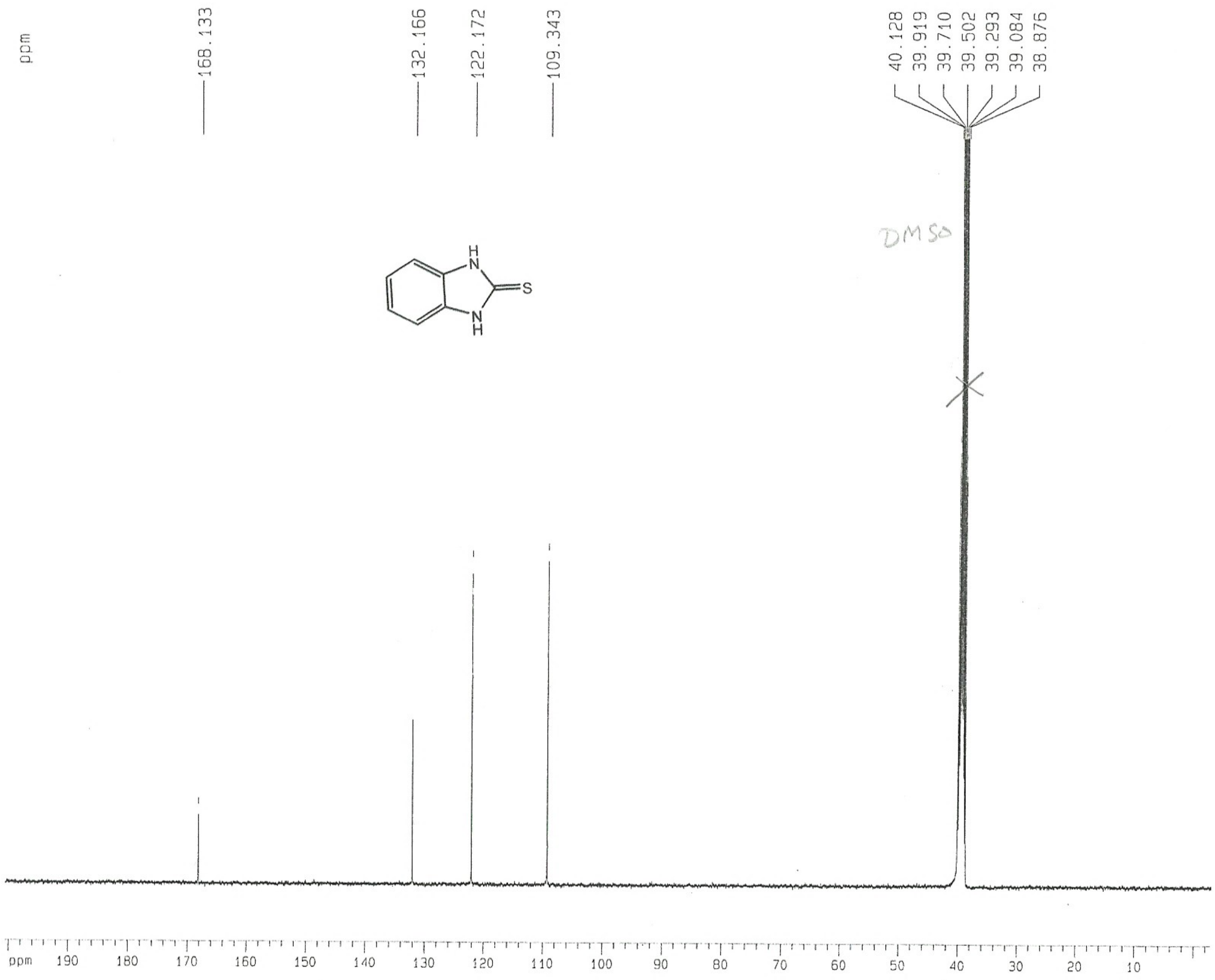

Current Data Parameters

NAME	july28
EXPNO	1
PROCNO	1

F2 - Acquisition Parameters

Date_	20070728
Time	14.00
INSTRUM	spect
PROBHD	5 mm DUL 13C-1
PULPROG	zgpg30
TD	32768
SOLVENT	DMSO
NS	25000
DS	2
SWH	26178.010 Hz
FIDRES	0.798889 Hz
AQ	0.6259188 sec
RG	16384
DW	19.100 usec
DE	20.00 usec
TE	300.0 K
D1	1.50000000 sec
d11	0.03000000 sec
d12	0.00002000 sec

======== CHANNEL f1 ========

NUC1	13C
P1	5.50 usec
PL1	0.00 dB
SFO1	100.6243395 MHz

======== CHANNEL f2 ========

CPDPRG2	waltz16
NUC2	1H
PCPD2	100.00 usec
PL2	-3.00 dB
PL12	16.50 dB
PL13	16.50 dB
SFO2	400.1320007 MHz

F2 - Processing parameters

SI	32768
SF	100.6128269 MHz
WDW	EM
SSB	0
LB	1.50 Hz
GB	0
PC	1.40

1D NMR plot parameters

CX	20.00 cm
CY	50.00 cm
F1P	200.653 ppm
F1	20188.24 Hz
F2P	-2.922 ppm
F2	-293.98 Hz
PPMCM	10.17873 ppm/cm
HZCM	1024.11084 Hz/cm

AVANCE 400
LAB. No. 117

M.RAMADAN/DR.EL.ASHRY/MRA-14
C13 {BB}

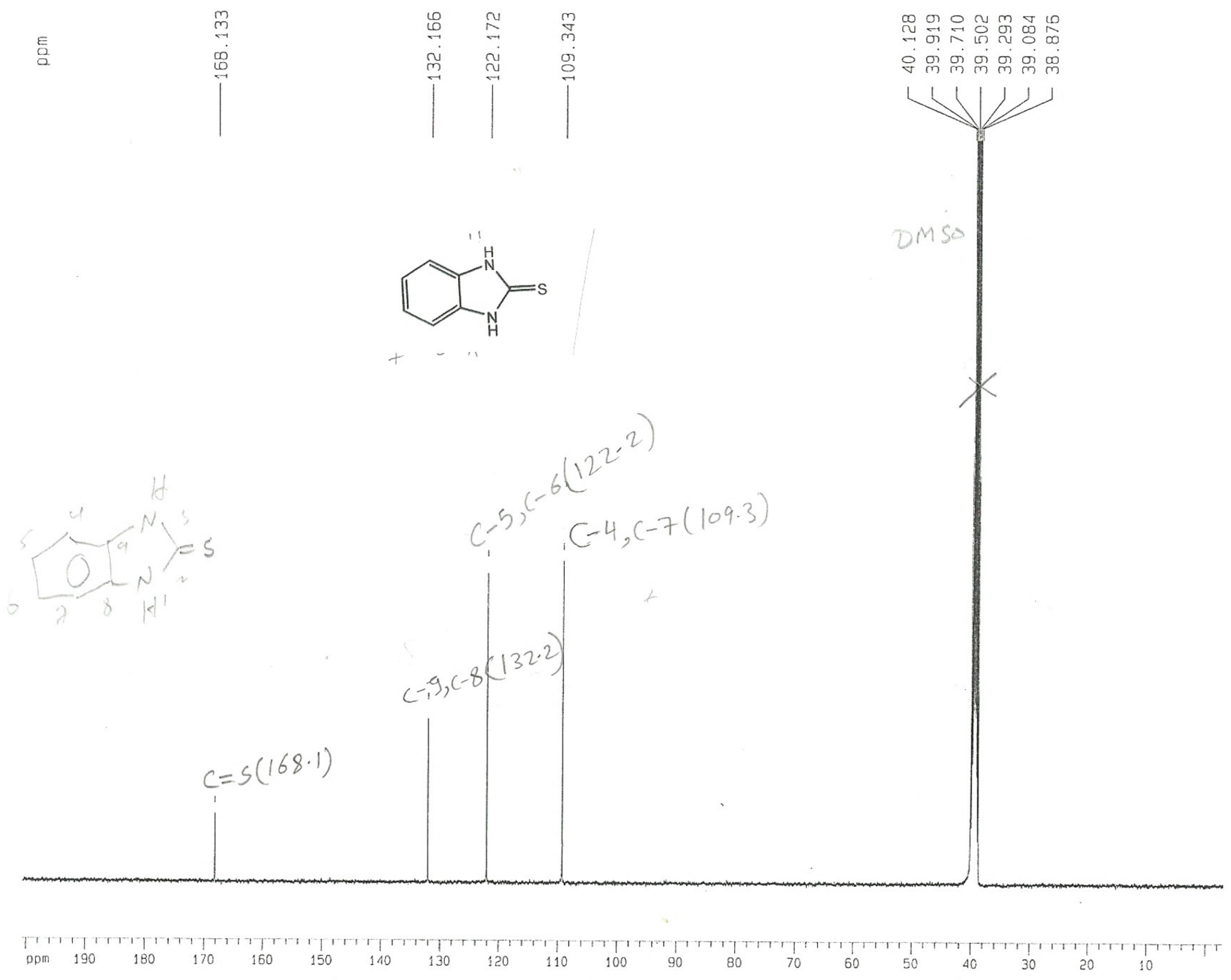

Current Data Parameters

NAME	july28
EXPNO	1
PROCNO	1

F2 - Acquisition Parameters

Date_	20070728
Time	14.00
INSTRUM	spect
PROBHD	5 mm DUL 13C-1
PULPROG	zgpg30
TD	32768
SOLVENT	DMSO
NS	25000
DS	2
SWH	26178.010 Hz
FIDRES	0.798889 Hz
AQ	0.6259188 sec
RG	16384
DW	19.100 usec
DE	20.00 usec
TE	300.0 K
D1	1.50000000 sec
d11	0.03000000 sec
d12	0.00002000 sec

======== CHANNEL f1 ========

NUC1	13C
P1	5.50 usec
PL1	0.00 dB
SFO1	100.6243395 MHz

======== CHANNEL f2 ========

CPDPRG2	waltz16
NUC2	1H
PCPD2	100.00 usec
PL2	-3.00 dB
PL12	16.50 dB
PL13	16.50 dB
SFO2	400.1320007 MHz

F2 - Processing parameters

SI	32768
SF	100.6128269 MHz
WDW	EM
SSB	0
LB	1.50 Hz
GB	0
PC	1.40

1D NMR plot parameters

CX	20.00 cm
CY	50.00 cm
F1P	200.653 ppm
F1	20188.24 Hz
F2P	-2.922 ppm
F2	-293.98 Hz
PPMCM	10.17873 ppm/cm
HZCM	1024.11084 Hz/cm

File: MRA10
Date Run: 10-04-2007 (Time Run: 09:20:54)
Sample: MOHAMED RAMADAN AMER
Instrument: JEOL MSRoute
Inlet: My Inlet
Ionization mode: EI-

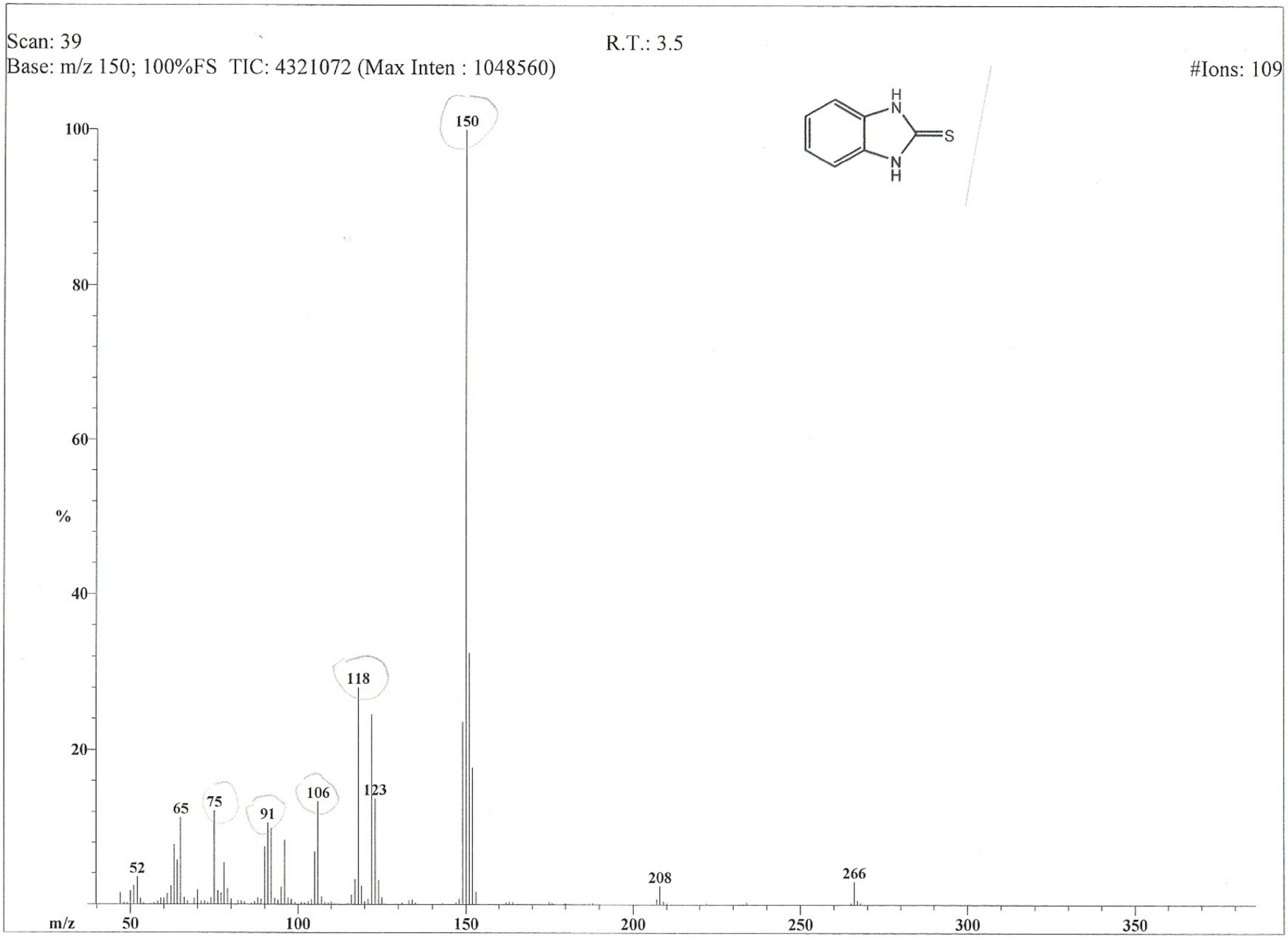

QURA TUL AIN/DR.EL ASHRY/QU.15B/DMSO

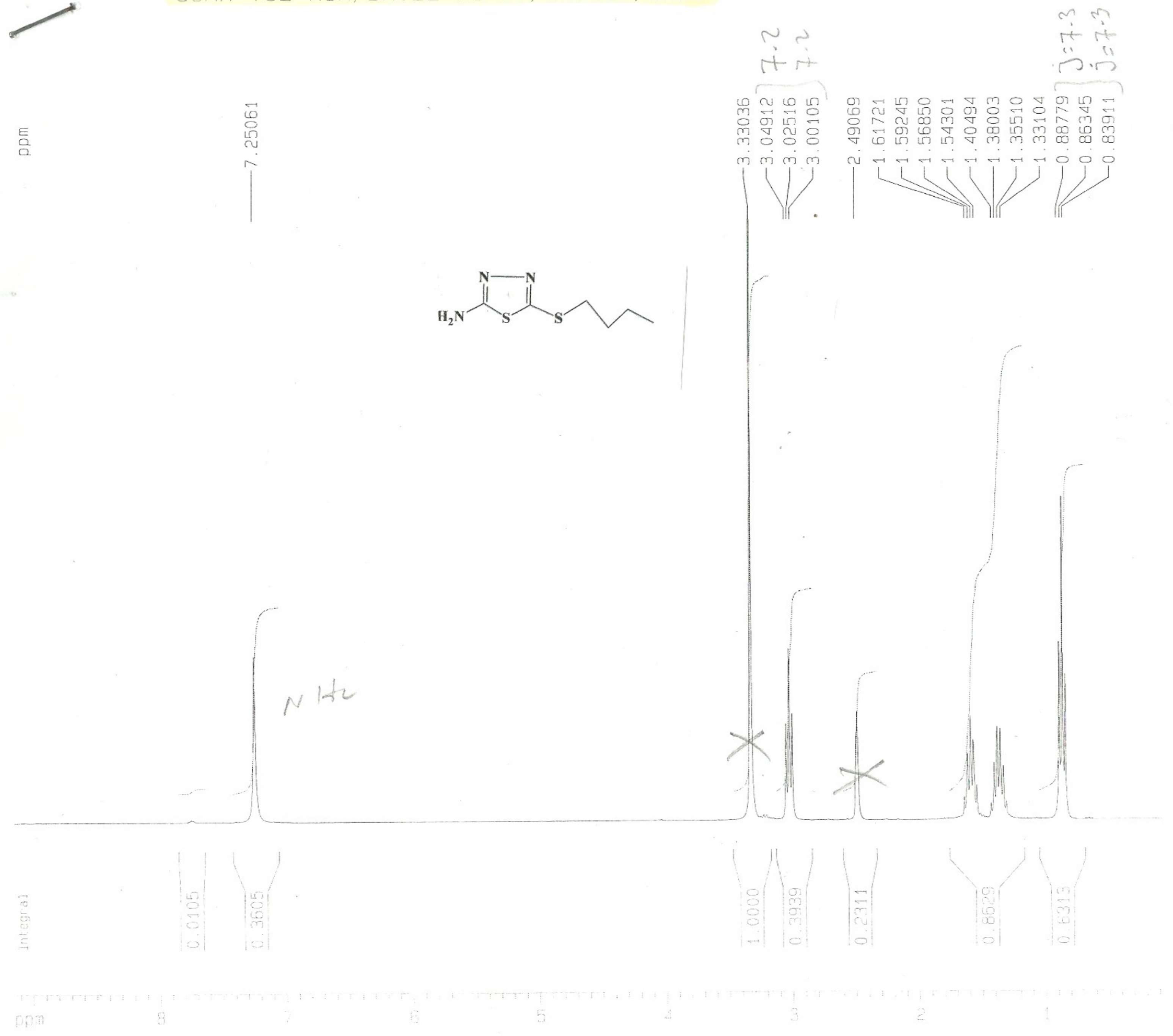

Current Data Parameters

NAME	may15
EXPNO	19
PROCNO	1

F2 - Acquisition Parameters

Date_	20080515
Time	14.33
INSTRUM	spect
PROBHD	5 mm DUL 13C-1
PULPROG	zg30
TD	32768
SOLVENT	DMSO
NS	128
DS	0
SWH	5995.204 Hz
FIDRES	0.182959 Hz
AQ	2.7329011 sec
RG	574.7
DW	83.400 usec
DE	10.00 usec
TE	0.0 K
D1	1.00000000 sec
MCREST	0.00000000 sec
MCWRK	0.01500000 sec

======== CHANNEL f1 ========

NUC1	1H
P1	10.20 usec
PL1	-3.00 dB
SFO1	300.1324010 MHz

F2 - Processing parameters

SI	16384
SF	300.1300042 MHz
WDW	EM
SSB	0
LB	0.30 Hz
GB	0
PC	1.00

1D NMR plot parameters

CX	20.00 cm
CY	20.00 cm
F1P	9.155 ppm
F1	2747.59 Hz
F2P	0.071 ppm
F2	21.30 Hz
PPMCM	0.45419 ppm/cm
HZCM	135.31480 Hz/cm

File: MRA-91 **Date Run: 03-26-2008 (Time Run: 11:16:08)**
Sample: MOHAMED RAMADAN AMER
Instrument: JEOL MSRoute
Inlet: My Inlet **Ionization mode: EI-**

Scan: 3 R.T.: .18
Base: m/z 190; 100%FS TIC: 5069312 (Max Inten : 1048560) #Ions: 82

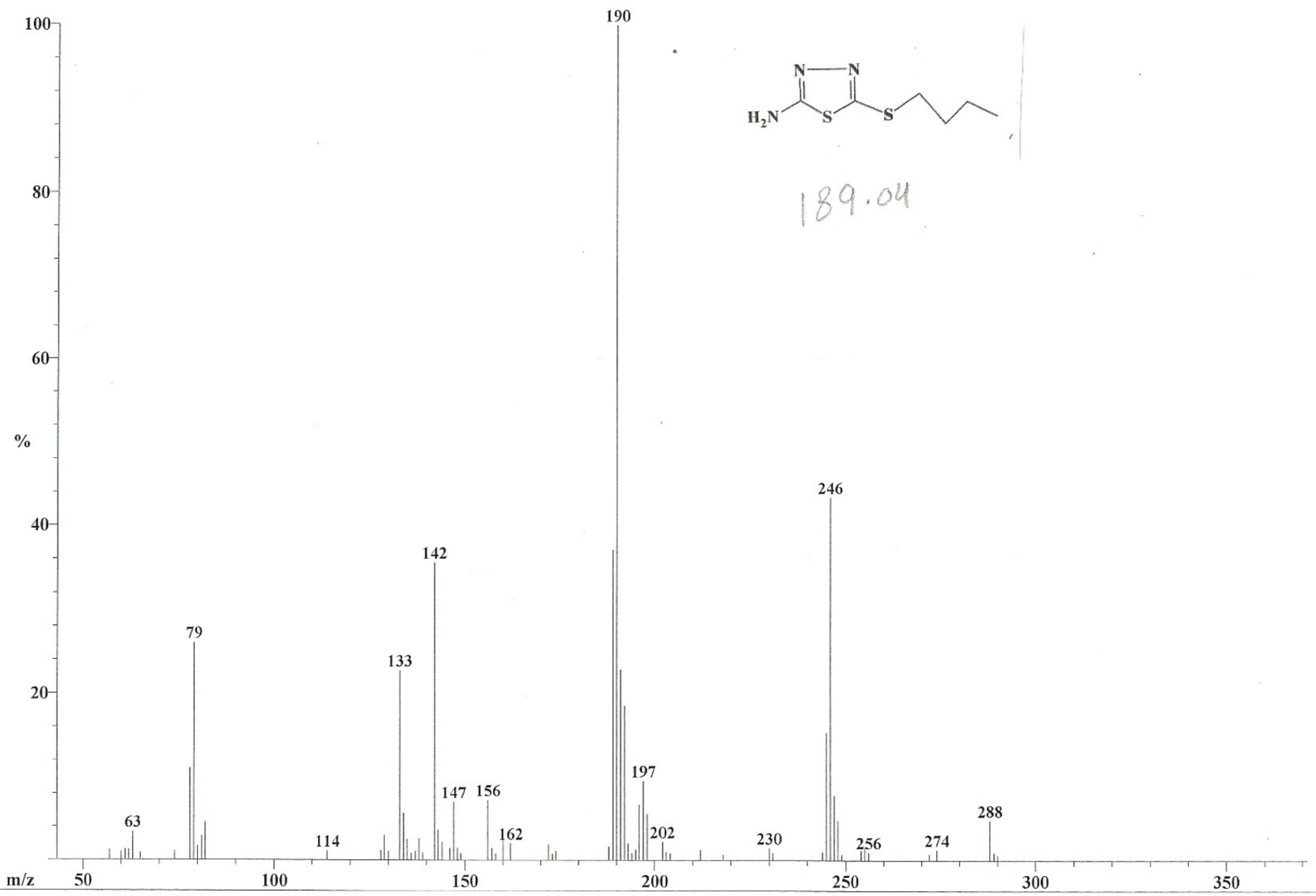

Mass	Relative Intensity	Theoretical Mass	Delta [ppm]	Delta [mmu]	RDB	Composition
188.0321	2.3877	188.0321	-0.1	-0.0	4.0	$C_7H_8O_6$
		188.0316	2.4	0.5	3.5	$C_6H_{10}N_3S_2$
		188.0330	-4.7	-0.9	3.0	$C_8H_{12}O_1S_2$
189.0050	1.5708	189.0056	-3.0	-0.6	0.0	$C_1H_7O_6N_3S_1$
		189.0044	3.2	0.6	3.5	$C_7H_9O_2S_2$
		189.0062	-6.4	-1.2	9.0	$C_9H_3O_4N_1$
189.0406	31.4038	189.0408	-1.2	-0.2	2.5	$C_8H_{13}O_1S_2$
		189.0399	3.4	0.6	3.5	$C_7H_9O_6$
		189.0394	5.9	1.1	3.0	$C_6H_{11}N_3S_2$
190.0448	4.3411	190.0452	-2.3	-0.4	7.0	$C_{11}H_{10}O_1S_1$
		190.0439	4.8	0.9	7.5	$C_9H_8N_3S_1$
		190.0464	-8.4	-1.6	3.5	$C_5H_8O_5N_3$
191.0384	2.7508	191.0378	3.1	0.6	2.5	$C_7H_{11}O_4S_1$
		191.0371	6.7	1.3	12.0	$C_{13}H_5O_1N_1$
		191.0365	10.1	1.9	3.0	$C_5H_9O_3N_3S_1$
192.9883	3.7881	192.9885	-1.4	-0.3	8.5	$C_7H_1O_5N_2$
		192.9894	-5.9	-1.1	7.5	$C_8H_5N_2S_2$
		192.9867	8.0	1.5	3.0	$C_5H_7O_3N_1S_2$
198.0699	16.7322	198.0701	-1.0	-0.2	4.5	$C_8H_{12}O_1N_3S_1$
		198.0715	-7.8	-1.5	4.0	$C_{10}H_{14}O_2S_1$
		198.0681	9.3	1.8	9.0	$C_{13}H_{10}O_2$
198.1055	14.8270	198.1045	5.1	1.0	8.0	$C_{14}H_{14}O_1$
		198.1065	-5.1	-1.0	3.5	$C_9H_{16}N_3S_1$
		198.1031	11.9	2.4	8.5	$C_{12}H_{12}N_3$
199.0736	2.4772	199.0746	-4.7	-0.9	9.0	$C_{11}H_9O_1N_3$
		199.0719	8.7	1.7	4.5	$C_8H_{11}O_4N_2$
		199.0759	-11.5	-2.3	8.5	$C_{13}H_{11}O_2$
199.1101	2.8232	199.1109	-4.5	-0.9	8.0	$C_{12}H_{13}N_3$
		199.1083	9.0	1.8	3.5	$C_9H_{15}O_3N_2$
		199.1123	-11.2	-2.2	7.5	$C_{14}H_{15}O_1$
200.0697	1.1723	200.0693	1.9	0.4	3.0	$C_{10}H_{16}S_2$
		200.0685	6.2	1.2	4.0	$C_9H_{12}O_5$
		200.0712	-7.2	-1.4	8.5	$C_{12}H_{10}O_2N_1$
200.1050	1.0602	200.1049	0.8	0.2	3.0	$C_{10}H_{16}O_4$
		200.1035	7.5	1.5	3.5	$C_8H_{14}O_3N_3$
		200.1075	-12.6	-2.5	7.5	$C_{13}H_{14}O_1N_1$
202.0501	4.3781	202.0504	-1.5	-0.3	8.5	$C_{11}H_8O_3N_1$
		202.0486	7.5	1.5	3.0	$C_9H_{14}O_1S_2$
		202.0477	11.8	2.4	4.0	$C_8H_{10}O_6$
203.0538	3.3306	203.0542	-1.9	-0.4	4.0	$C_6H_9O_5N_3$
		203.0531	3.8	0.8	7.5	$C_{12}H_{11}O_1S_1$
		203.0551	-6.2	-1.2	3.0	$C_7H_{13}N_3S_2$
204.9883	3.4626	204.9885	-1.3	-0.3	9.5	$C_8H_1O_5N_2$
		204.9892	-4.7	-1.0	0.0	$C_2H_7O_8N_1S_1$

Found

Calc:

Found: 189.0406

Calc. : 189.0394

$C_6H_{11}N_3S_2$

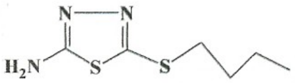

found = 189.0406

Calc. = 189.0394

Error = 6.3 ppm

AVANCE AV 300
LAB. No. 115

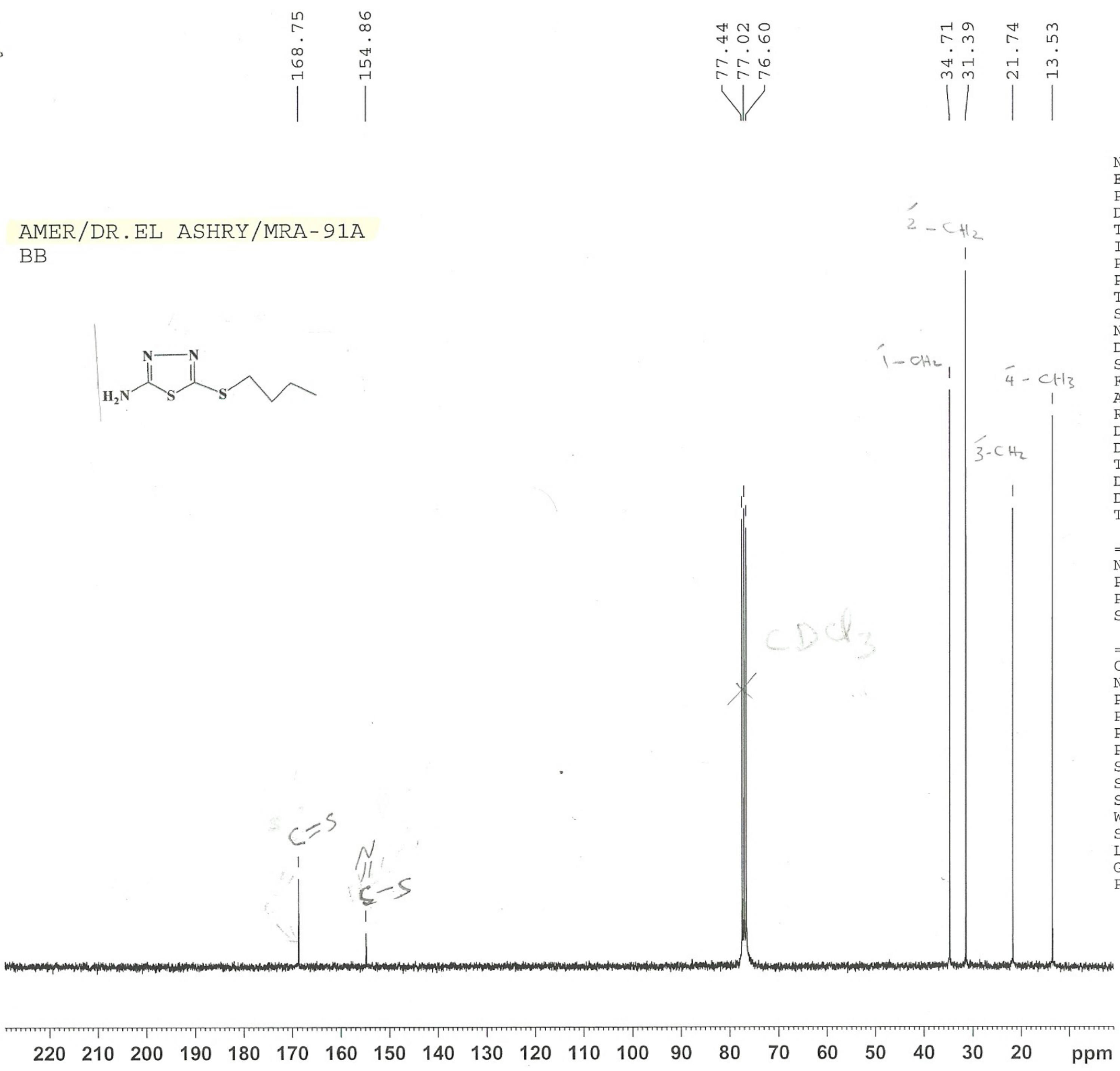

NAME	march15
EXPNO	1
PROCNO	1
Date_	20090315
Time	12.52
INSTRUM	spect
PROBHD	5 mm DUL 13C-1
PULPROG	zgpg
TD	32768
SOLVENT	CDCl3
NS	20480
DS	2
SWH	17985.611 Hz
FIDRES	0.548877 Hz
AQ	0.9110004 sec
RG	32768
DW	27.800 usec
DE	6.50 usec
TE	300.0 K
D1	1.50000000 sec
D11	0.03000000 sec
TD0	20

======== CHANNEL f1 ========

NUC1	13C
P1	11.50 usec
PL1	-2.00 dB
SFO1	75.4765033 MHz

======== CHANNEL f2 ========

CPDPRG2	waltz16
NUC2	1H
PCPD2	100.00 usec
PL2	-3.00 dB
PL12	17.00 dB
PL13	20.00 dB
SFO2	300.1315007 MHz
SI	16384
SF	75.4677492 MHz
WDW	EM
SSB	0
LB	1.00 Hz
GB	0
PC	1.40

AAMER/DR.EL ASHRY/MRA.207

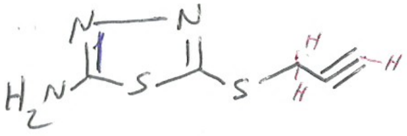

(NH2, s) 7.40
(SCH2, d) 5.24
(CH, s) 3.30

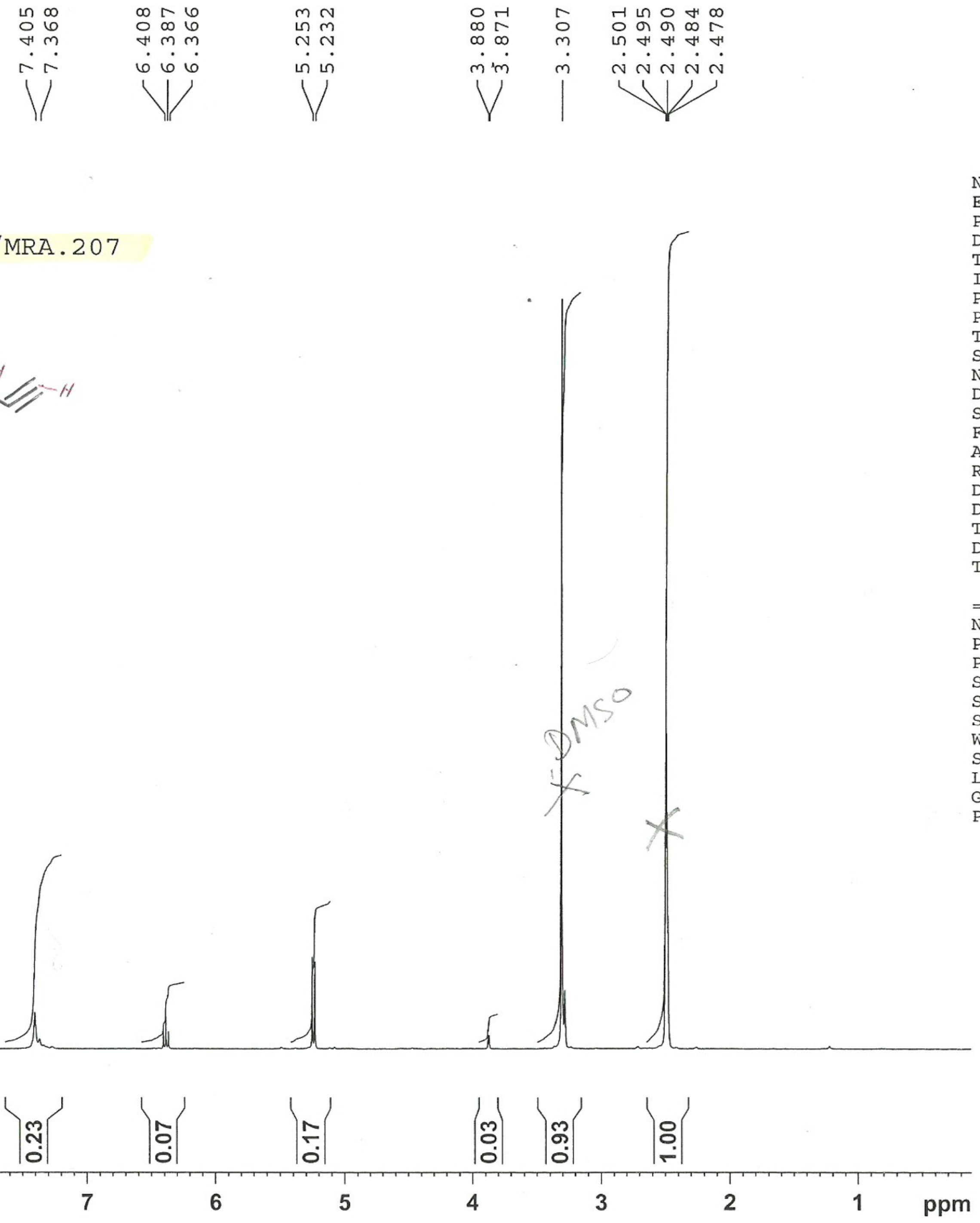

NAME	sep10
EXPNO	4
PROCNO	1
Date_	20090910
Time	10.38
INSTRUM	spect
PROBHD	5 mm DUL 13C-1
PULPROG	zg30
TD	32768
SOLVENT	DMSO
NS	128
DS	0
SWH	6172.839 Hz
FIDRES	0.188380 Hz
AQ	2.6542580 sec
RG	1625.5
DW	81.000 usec
DE	6.50 usec
TE	300.0 K
D1	1.50000000 sec
TD0	1

======== CHANNEL f1 ========

NUC1	1H
P1	10.00 usec
PL1	-3.00 dB
SFO1	300.1324010 MHz
SI	16384
SF	300.1300041 MHz
WDW	EM
SSB	0
LB	0.30 Hz
GB	0
PC	1.00

AVANCE AV 30[illegible] LAB No 11[illegible]

AVANCE AV 300
LAB. No. 115

QURAT-UL-AIN/DR.EL ASHRY/QU-15A

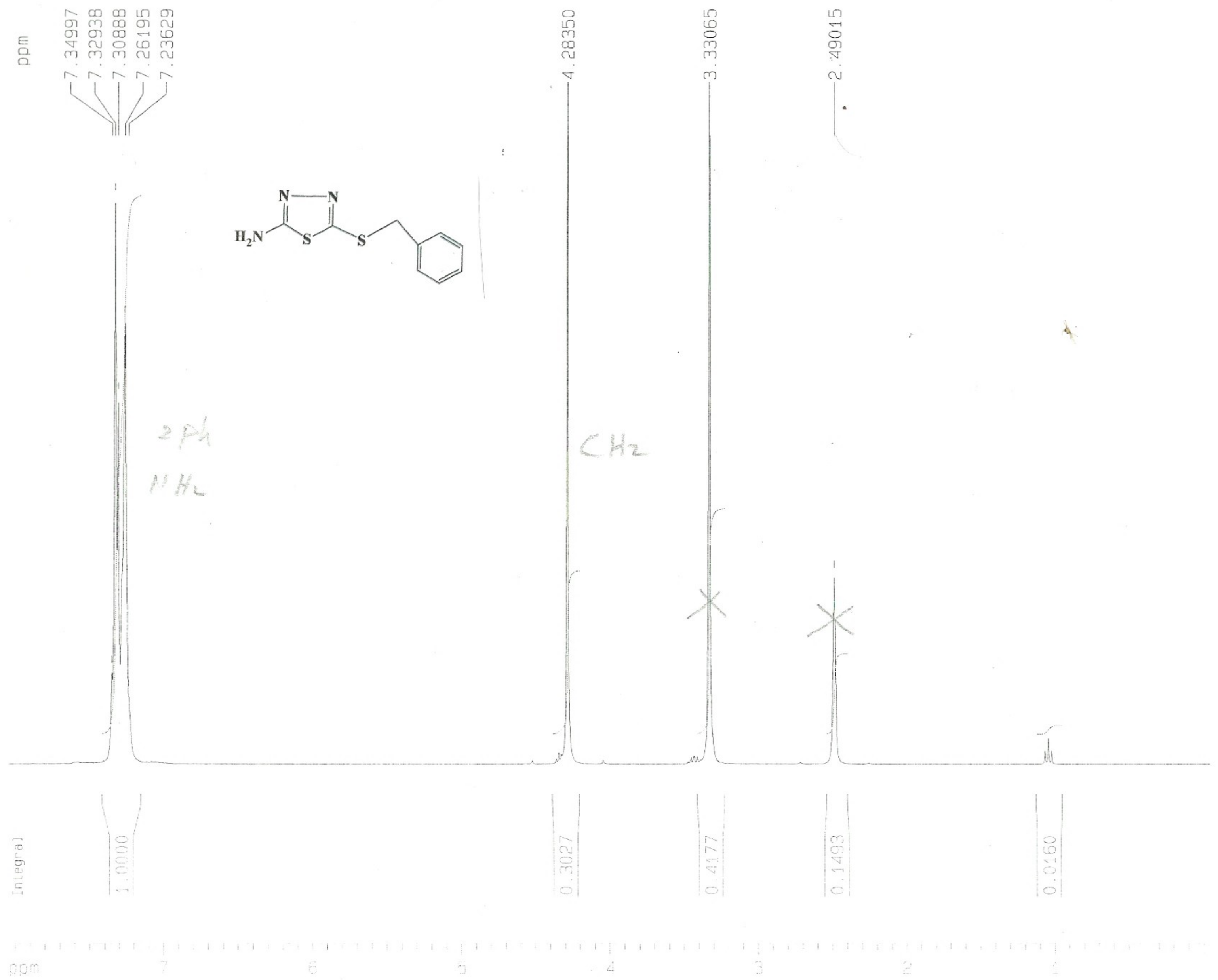

Current Data Parameters
NAME may09
EXPNO 7
PROCNO 1

F2 - Acquisition Parameters
Date_ 20080509
Time 11.02
INSTRUM spect
PROBHD 5 mm DUL 13C-1
PULPROG zg30
TD 32768
SOLVENT DMSO
NS 64
DS 0
SWH 5995.204 Hz
FIDRES 0.182959 Hz
AQ 2.7329011 sec
RG 512
DW 83.400 usec
DE 10.00 usec
TE 0.0 K
D1 1.00000000 sec
MCREST 0.00000000 sec
MCWRK 0.01500000 sec

======== CHANNEL f1 ========
NUC1 1H
P1 10.20 usec
PL1 -3.00 dB
SFO1 300.1324010 MHz

F2 - Processing parameters
SI 16384
SF 300.1300042 MHz
WDW EM
SSB 0
LB 0.30 Hz
GB 0
PC 1.00

1D NMR plot parameters
CX 20.00 cm
CY 0.00 cm
F1P 8.046 ppm
F1 2414.90 Hz
F2P -0.040 ppm
F2 -12.01 Hz
PPMCM 0.40431 ppm/cm
HZCM 121.34581 Hz/cm

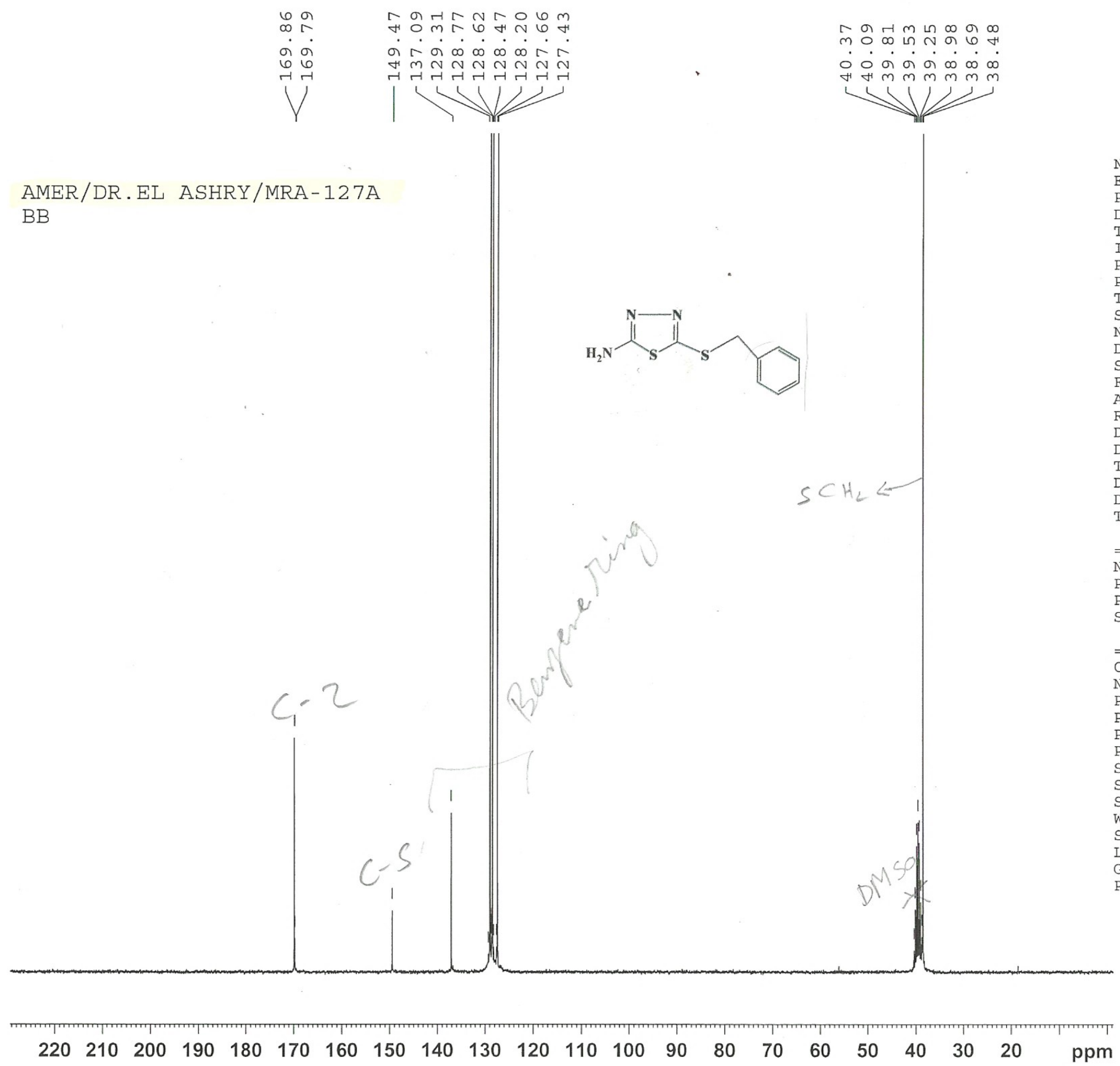

NAME	march16
EXPNO	11
PROCNO	1
Date_	20090316
Time	13.41
INSTRUM	spect
PROBHD	5 mm DUL 13C-1
PULPROG	zgpg
TD	32768
SOLVENT	DMSO
NS	20480
DS	2
SWH	17985.611 Hz
FIDRES	0.548877 Hz
AQ	0.9110004 sec
RG	32768
DW	27.800 usec
DE	6.50 usec
TE	300.0 K
D1	1.50000000 sec
D11	0.03000000 sec
TD0	20
======== CHANNEL f1 ========	
NUC1	13C
P1	11.50 usec
PL1	-2.00 dB
SFO1	75.4765033 MHz
======== CHANNEL f2 ========	
CPDPRG2	waltz16
NUC2	1H
PCPD2	100.00 usec
PL2	-3.00 dB
PL12	17.00 dB
PL13	20.00 dB
SFO2	300.1315007 MHz
SI	16384
SF	75.4677838 MHz
WDW	EM
SSB	0
LB	1.00 Hz
GB	0
PC	1.40

File Name	:	d:\mswin\data\mra-127.mss
Creation Date/Time	:	26/03/08 at 10:50:45
File Type	:	Lo-Res Mass Data (Centroid)
File Source	:	Acquired on MASPEC system [msw/A091]
File Title	:	EI
Operator	:	Barkat Ali
Instrument	:	MAT312

SCAN GRAPH. Flagging=M/z. Filter=[Int:1%.].
Scan 10-3:30. Entries=30. 100% Int.=109420.

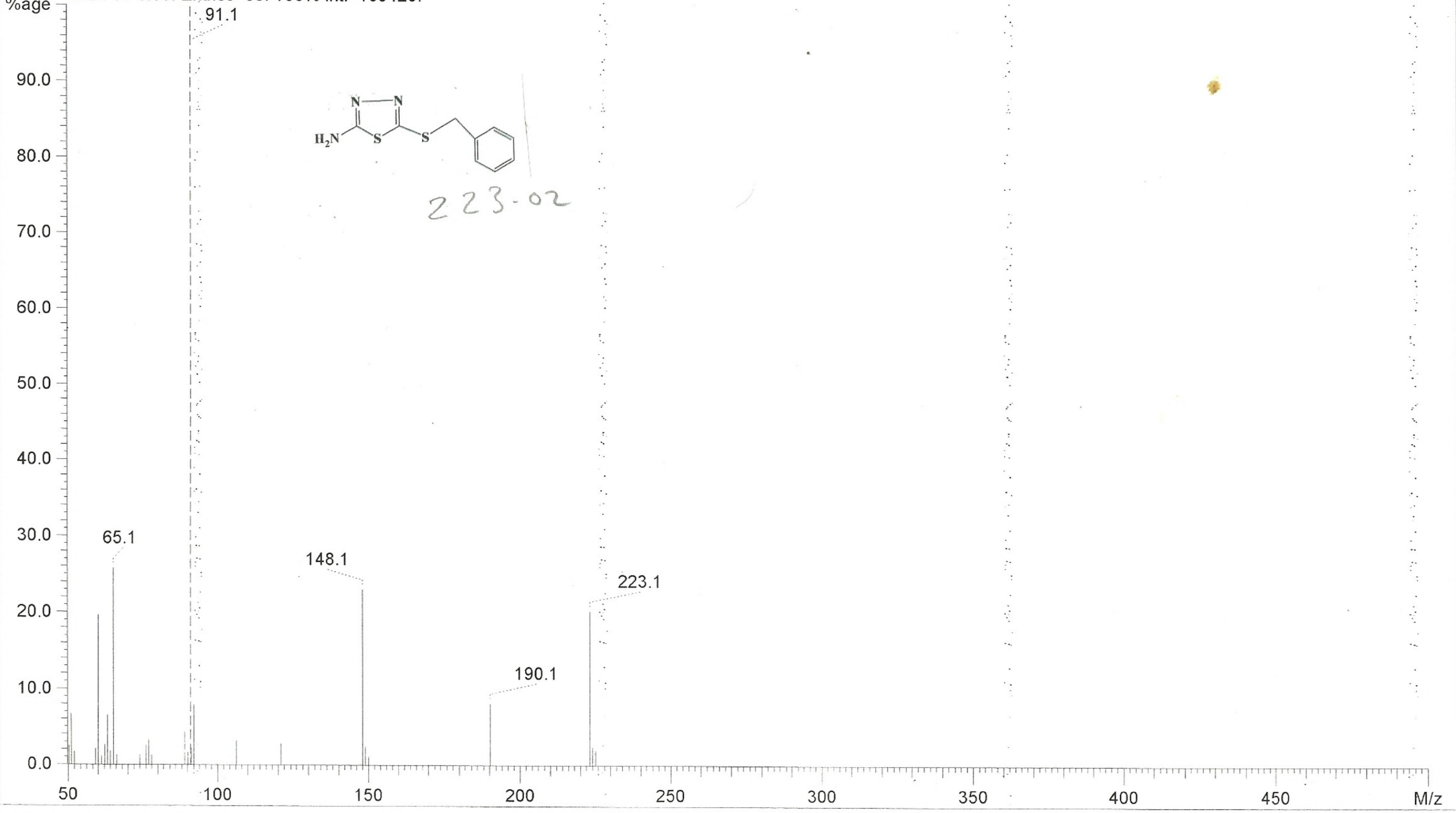

M.RAMADAN/DR.EL ASHRY/MRA.32/DMSO

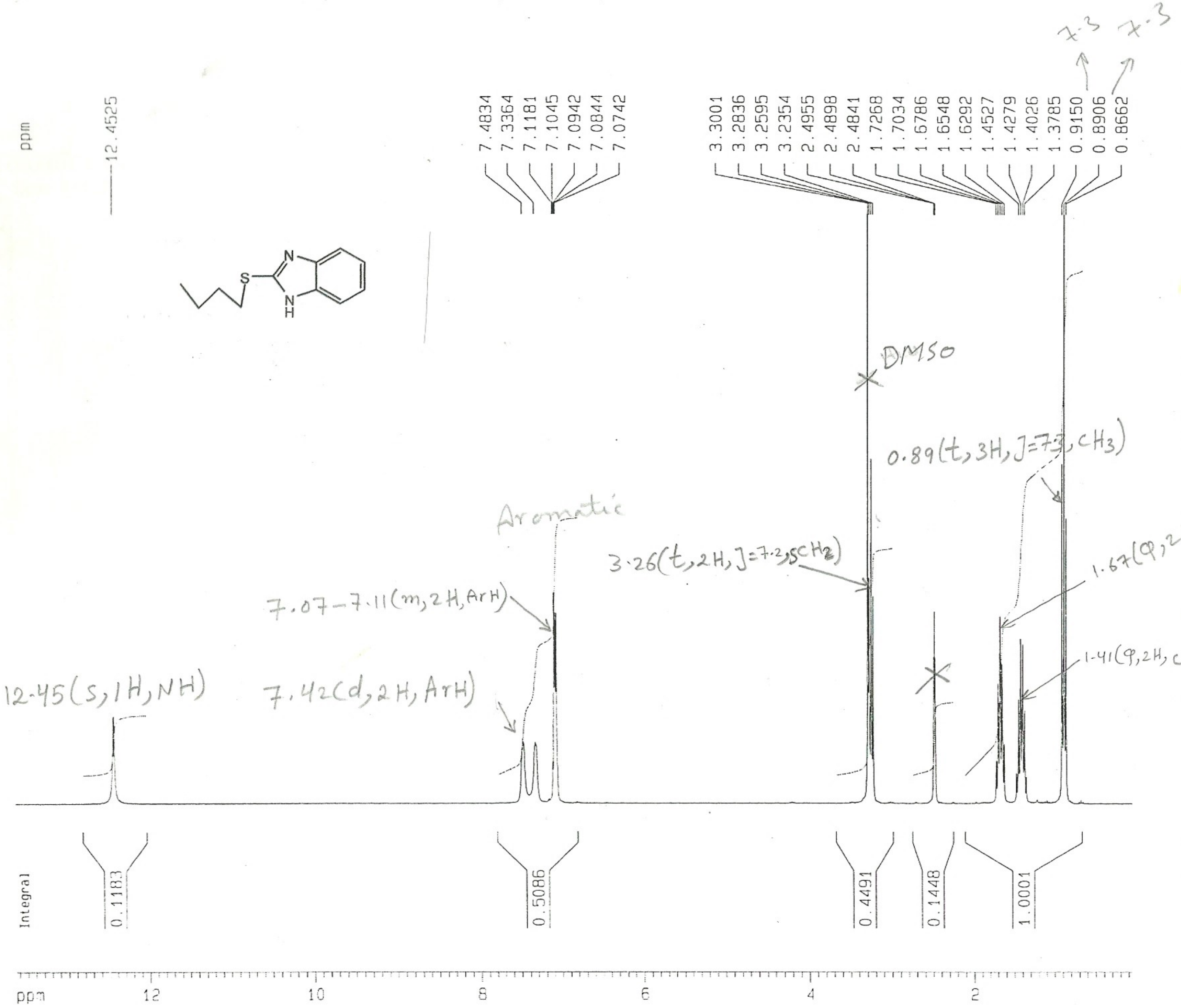

Current Data Parameters

NAME	july16
EXPNO	2
PROCNO	1

F2 - Acquisition Parameters

Date_	20070716
Time	11.17
INSTRUM	spect
PROBHD	5 mm DUL 13C-1
PULPROG	zg30
TD	32768
SOLVENT	DMSO
NS	128
DS	0
SWH	5995.204 Hz
FIDRES	0.182959 Hz
AQ	2.7329011 sec
RG	1290.2
DW	83.400 usec
DE	10.00 usec
TE	0.0 K
D1	1.00000000 sec
MCREST	0.00000000 sec
MCWRK	0.01500000 sec

======== CHANNEL f1 ========

NUC1	1H
P1	10.20 usec
PL1	-3.00 dB
SFO1	300.1324010 MHz

F2 - Processing parameters

SI	16384
SF	300.1300042 MHz
WDW	EM
SSB	0
LB	0.30 Hz
GB	0
PC	1.00

1D NMR plot parameters

CX	20.00 cm
CY	12.50 cm
F1P	13.650 ppm
F1	4096.92 Hz
F2P	0.060 ppm
F2	18.02 Hz
PPMCM	0.67952 ppm/cm
HZCM	203.94507 Hz/cm

M.Amer Ali/Dr.EL Ashry
Sample: MRA-32

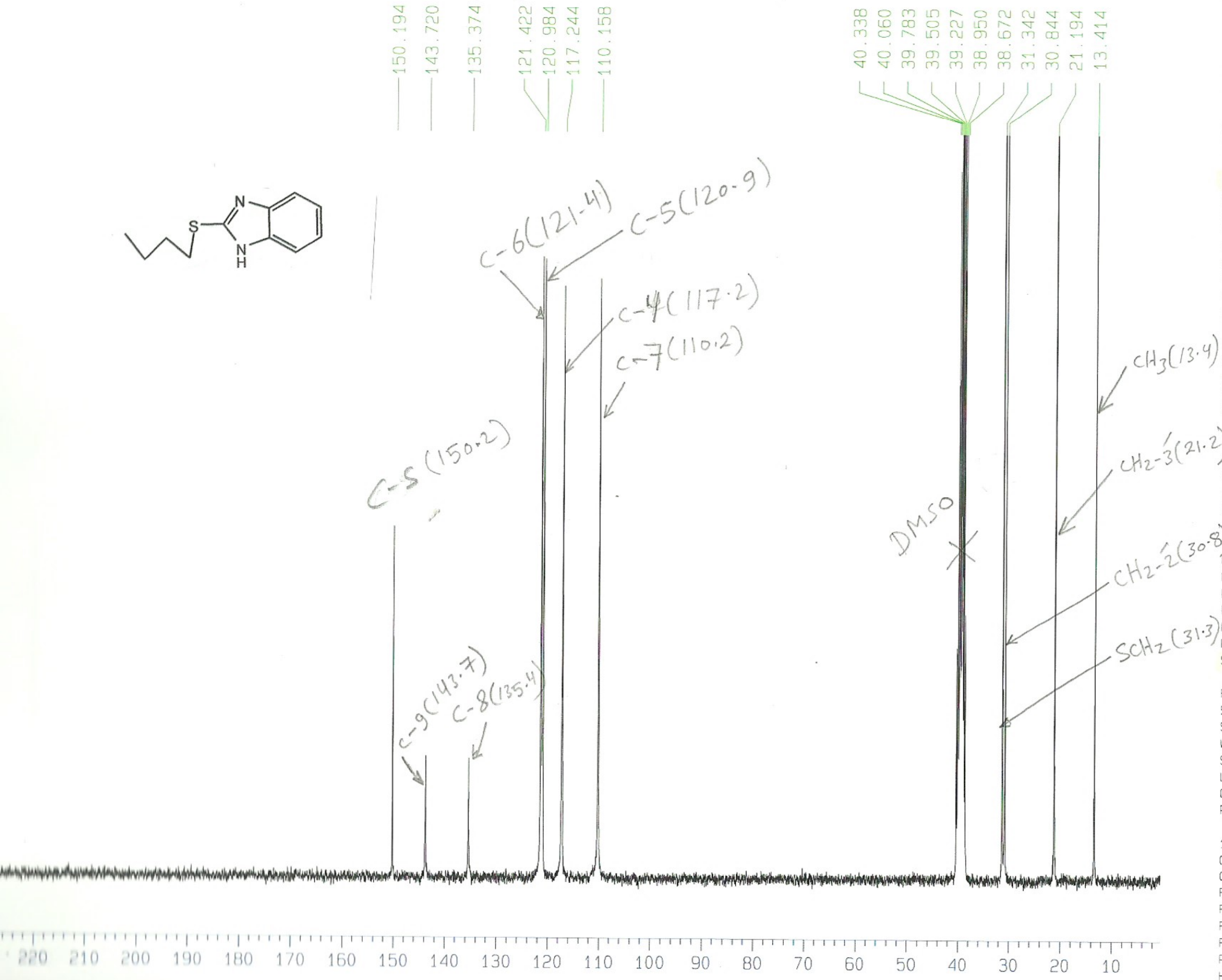

Current Data Parameters
NAME jan22
EXPNO 6
PROCNO 1

F2 - Acquisition Parameters
Date_ 20080122
Time 11.01
INSTRUM spect
PROBHD 5 mm DUL 13C-1
PULPROG zgpg
TD 66560
SOLVENT DMSO
NS 9216
DS 4
SWH 17985.611 Hz
FIDRES 0.270217 Hz
AQ 1.8504180 sec
RG 32768
DW 27.800 usec
DE 6.00 usec
TE 302.5 K
D1 1.50000000 sec
d11 0.03000000 sec
DELTA 1.39999998 sec
MCREST 0.00000000 sec
MCWRK 0.01500000 sec

======== CHANNEL f1 ========
NUC1 13C
P1 6.50 usec
PL1 0.00 dB
SFO1 75.4764278 MHz

======== CHANNEL f2 ========
CPDPRG2 waltz16
NUC2 1H
PCPD2 85.00 usec
PL2 0.00 dB
PL12 19.00 dB
PL13 19.00 dB
SFO2 300.1315007 MHz

F2 - Processing parameters
SI 32768
SF 75.4677867 MHz
WDW EM
SSB 0
LB 1.50 Hz
GB 0
PC 1.20

1D NMR plot parameters
CX 20.00 cm
CY 35.00 cm
F1P 233.150 ppm
F1 17595.35 Hz
F2P 0.442 ppm
F2 33.38 Hz
PPMCM 11.63541 ppm/cm
HZCM 878.09839 Hz/cm

File Name	:	d:\mswin\data\mra-32.mss
Creation Date/Time	:	16/07/07 at 11:58:14
File Type	:	Lo-Res Mass Data (Centroid)
File Source	:	Acquired on MASPEC system [msw/A091]
File Title	:	EI
Operator	:	Barkat Ali
Instrument	:	MAT312

SCAN GRAPH. Flagging=M/z. Filter=[Int:1%.].

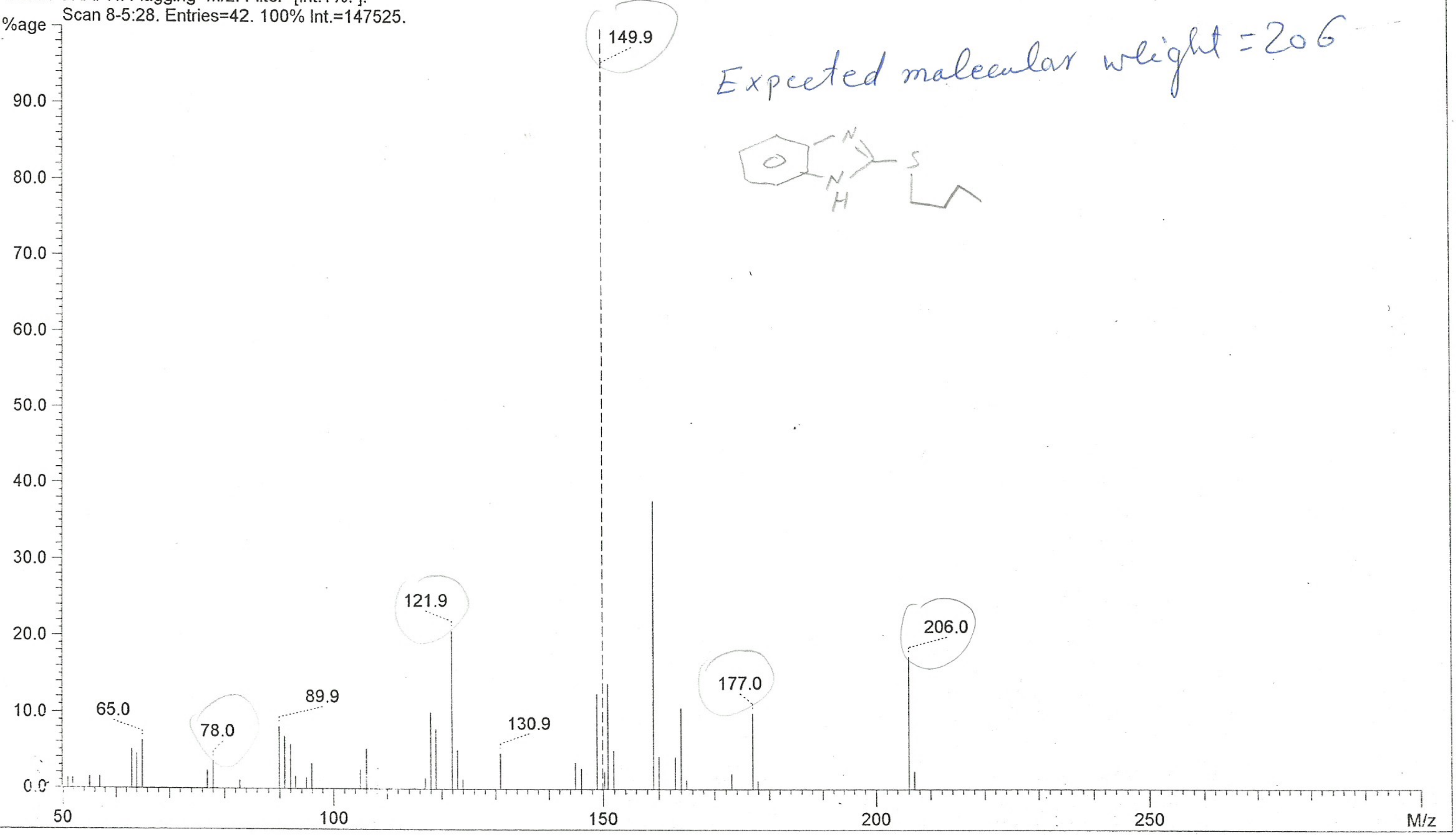

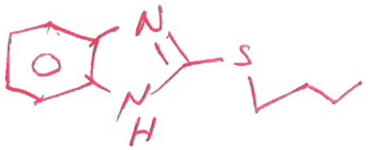

Mass	Relative Intensity	Theoretical Mass	Delta [ppm]	Delta [mmu]	Composition
180.9883	21.7709	180.9885	-1.5	-0.3	$C_6 H_1 O_5 N_2$
		180.9860	12.3	2.2	$C_{10} H_1 N_2 S_1$
		180.9859	13.3	2.4	$C_3 H_3 O_8 N_1$
181.9902	0.8905	181.9912	-5.2	-0.9	$C_7 H_4 O_3 N_1 S_1$
		181.9885	9.6	1.7	$C_4 H_6 O_6 S_1$
		181.9878	13.4	2.4	$C_{10} O_3 N_1$
192.9883	4.9658	192.9885	-1.4	-0.3	$C_7 H_1 O_5 N_2$
		192.9860	11.6	2.2	$C_{11} H_1 N_2 S_1$
		192.9859	12.5	2.4	$C_4 H_3 O_8 N_1$
204.9883	2.2157	204.9885	-1.3	-0.3	$C_8 H_1 O_5 N_2$
		204.9860	10.9	2.2	$C_{12} H_1 N_2 S_1$
		204.9859	11.7	2.4	$C_5 H_3 O_8 N_1$
206.0864	7.2280	206.0878	-6.5	-1.3	$C_{11} H_{14} N_2 S_1$
		206.0851	6.5	1.3	$C_8 H_{16} O_3 N_1 S_1$
		206.0844	9.9	2.0	$C_{14} H_{10} N_2$
207.0901	1.5364	207.0895	2.5	0.5	$C_{11} H_{13} O_3 N_1$
		207.0922	-10.4	-2.2	$C_{14} H_{11} N_2$
		207.0929	-13.8	-2.9	$C_8 H_{17} O_3 N_1 S_1$
211.9844	1.1711	211.9831	5.9	1.3	$C_7 H_2 O_7 N_1$
		211.9858	-6.7	-1.4	$C_{10} O_4 N_2$
		211.9865	-10.0	-2.1	$C_4 H_6 O_7 N_1 S_1$
216.9871	1.0198	216.9860	4.8	1.0	$C_{13} H_1 N_2 S_1$
		216.9859	5.6	1.2	$C_6 H_3 O_8 N_1$
		216.9885	-6.7	-1.5	$C_9 H_1 O_5 N_2$
218.9851	11.0914	218.9863	-5.4	-1.2	$C_2 H_5 O_{11} N_1$
		218.9838	6.0	1.3	$C_6 H_5 O_6 N_1 S_1$
		218.9864	-6.2	-1.4	$C_9 H_3 O_3 N_2 S_1$
230.9851	14.9390	230.9863	-5.1	-1.2	$C_3 H_5 O_{11} N_1$
		230.9838	5.7	1.3	$C_7 H_5 O_6 N_1 S_1$
		230.9864	-5.9	-1.4	$C_{10} H_3 O_3 N_2 S_1$
231.9907	0.8308	231.9909	-0.9	-0.2	$C_{13} O_3 N_2$
		231.9916	-3.8	-0.9	$C_7 H_6 O_6 N_1 S_1$
		231.9889	7.7	1.8	$C_4 H_8 O_9 S_1$
235.9849	1.7994	235.9858	-3.8	-0.9	$C_{12} O_4 N_2$
		235.9865	-6.8	-1.6	$C_6 H_6 O_7 N_1 S_1$
		235.9831	7.5	1.8	$C_9 H_2 O_7 N_1$
242.9851	7.5964	242.9863	-4.9	-1.2	$C_4 H_5 O_{11} N_1$
		242.9838	5.4	1.3	$C_8 H_5 O_6 N_1 S_1$
		242.9864	-5.6	-1.4	$C_{11} H_3 O_3 N_2 S_1$
254.9851	2.7585	254.9863	-4.6	-1.2	$C_5 H_5 O_{11} N_1$
		254.9838	5.2	1.3	$C_9 H_5 O_6 N_1 S_1$
		254.9864	-5.3	-1.4	$C_{12} H_3 O_3 N_2 S_1$
266.9833	1.4164	266.9838	-1.7	-0.5	$C_{10} H_5 O_6 N_1 S_1$
		266.9811	8.3	2.2	$C_7 H_7 O_9 S_1$

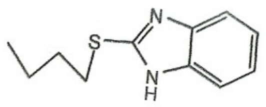

Found = 206.0864

Calc. = 206.0878

$C_{11}H_{14}N_2S$

inting Time: 10:36:02 AM
inting Date: Saturday, April 04, 2009

*Installation
*QStar XL Ser. no. Y3200502

	Formula	Calculated m/z (amu)	mDa Error	PPM Error	DBE
1	C14 H11 N2	207.0916	1.3251	6.3986	10.5
2	C11 H15 N2 S	207.0950	-2.0468	-9.8836	5.5

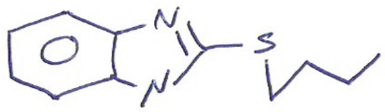

Found = 207.0950

Calc. = 207.0930

$C_{11}H_{15}N_2S$

AVANCE AV 300
LAB. No. 115

M.RAMADA/DR.EL ASHRY/MRA.28/CDCL3

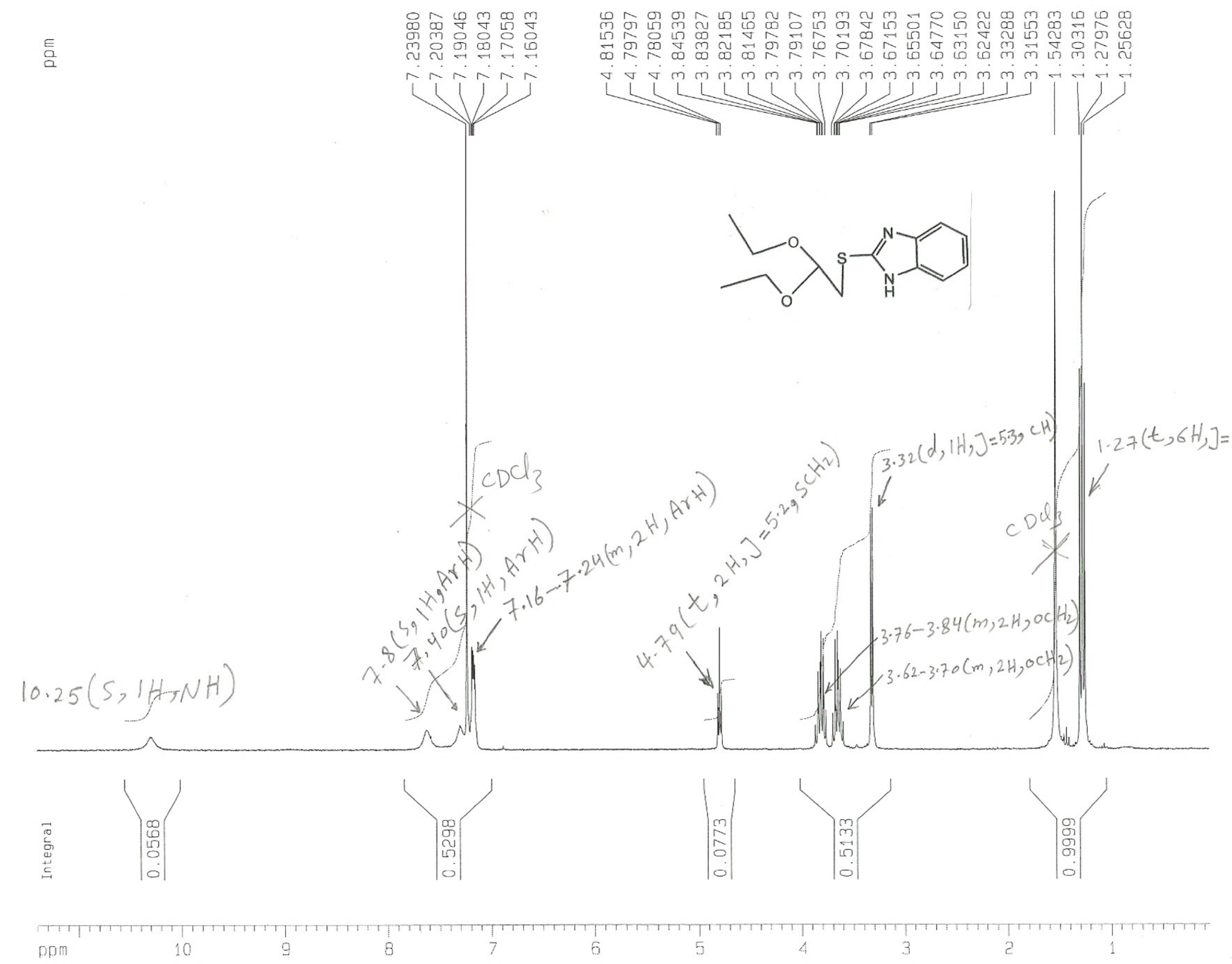

Current Data Parameters

NAME	july25
EXPNO	3
PROCNO	1

F2 - Acquisition Parameters

Date_	20070725
Time	11.39
INSTRUM	spect
PROBHD	5 mm DUL 13C-1
PULPROG	zg30
TD	32768
SOLVENT	CDCl3
NS	128
DS	0
SWH	5995.204 Hz
FIDRES	0.182959 Hz
AQ	2.7329011 sec
RG	2298.8
DW	83.400 usec
DE	10.00 usec
TE	0.0 K
D1	1.00000000 sec
MCREST	0.00000000 sec
MCWRK	0.01500000 sec

======== CHANNEL f1 ========

NUC1	1H
P1	10.20 usec
PL1	-3.00 dB
SFO1	300.1324010 MHz

F2 - Processing parameters

SI	16384
SF	300.1300123 MHz
WDW	EM
SSB	0
LB	0.30 Hz
GB	0
PC	1.00

1D NMR plot parameters

CX	20.00 cm
CY	13.00 cm
F1P	11.402 ppm
F1	3421.96 Hz
F2P	0.053 ppm
F2	15.87 Hz
PPMCM	0.56744 ppm/cm
HZCM	170.30464 Hz/cm

M.RAMDAN/DR.EL ASHRY/MRA-41/CDCL3

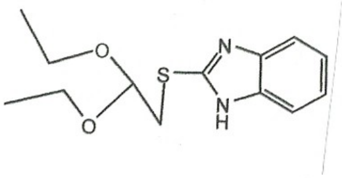

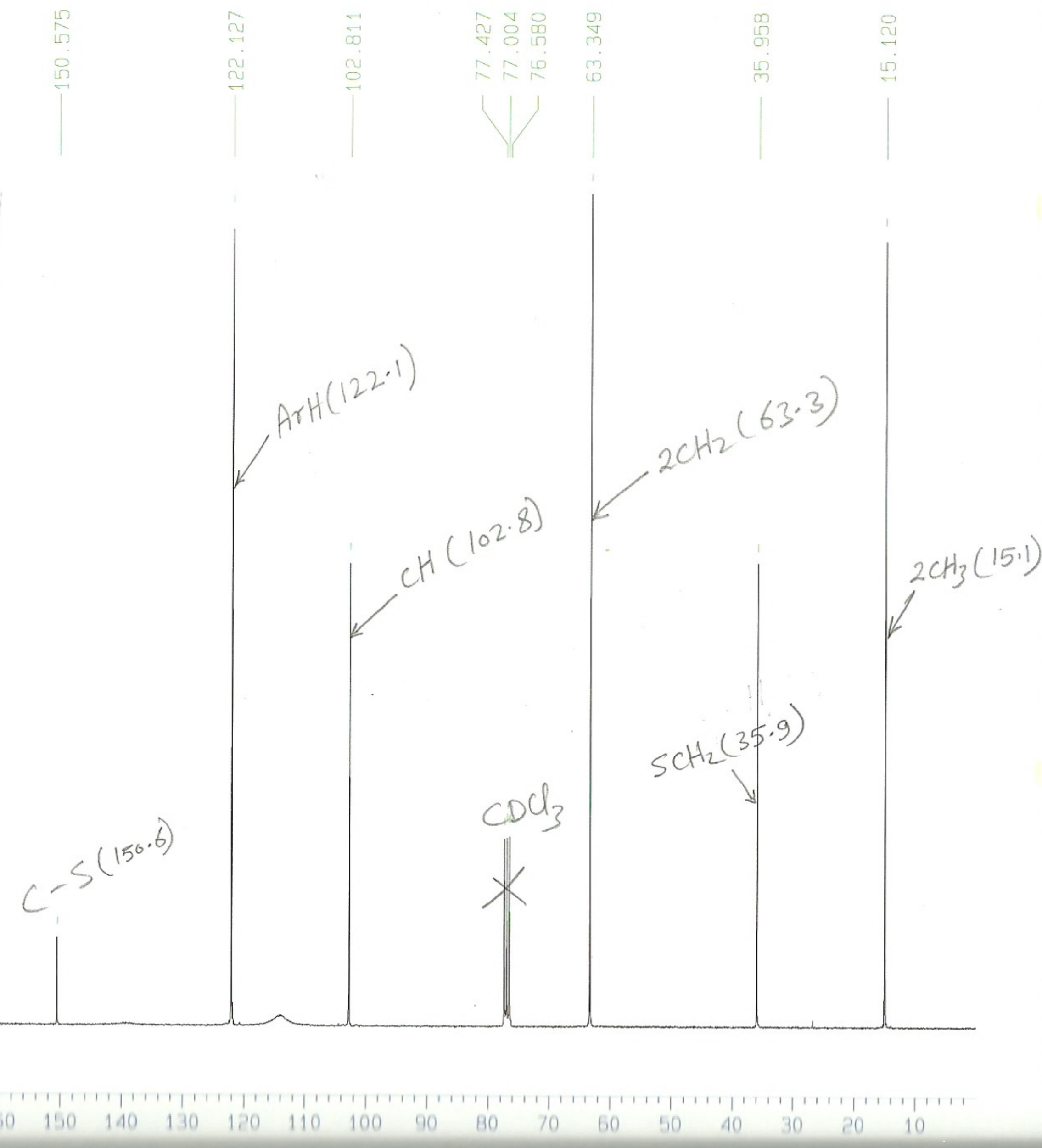

Current Data Parameters
NAME oct05
EXPNO 5
PROCNO 1

F2 - Acquisition Parameters
Date_ 20071005
Time 7.02
INSTRUM spect
PROBHD 5 mm DUL 13C-1
PULPROG zgpg
TD 66560
SOLVENT CDCl3
NS 20480
DS 4
SWH 17985.611 Hz
FIDRES 0.270217 Hz
AQ 1.8504180 sec
RG 32768
DW 27.800 usec
DE 6.00 usec
TE 300.0 K
D1 1.00000000 sec
d11 0.03000000 sec
DELTA 0.89999998 sec
MCREST 0.00000000 sec
MCWRK 0.01500000 sec

======== CHANNEL f1 ========
NUC1 13C
P1 6.50 usec
PL1 0.00 dB
SFO1 75.4764278 MHz

======== CHANNEL f2 ========
CPDPRG2 waltz16
NUC2 1H
PCPD2 85.00 usec
PL2 0.00 dB
PL12 19.00 dB
PL13 19.00 dB
SFO2 300.1315007 MHz

F2 - Processing parameters
SI 32768
SF 75.4677527 MHz
WDW EM
SSB 0
LB 1.00 Hz
GB 0
PC 1.20

1D NMR plot parameters
CX 20.00 cm
CY 12.00 cm
F1P 227.664 ppm
F1 17181.28 Hz
F2P 0.088 ppm
F2 6.64 Hz
PPMCM 11.37879 ppm/cm
HZCM 858.73181 Hz/cm

AVANCE AV-300
LAB No. 108

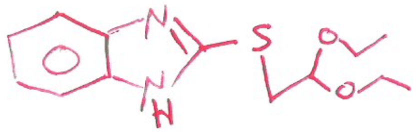

Mass	Relative Intensity	Theoretical Mass	Delta [ppm]	Delta [mmu]	Composition
		230.9811	17.3	4.0	$C_{4}H_{7}O_{9}S_{1}$
237.0676	2.3932	237.0671	2.3	0.5	$C_{8}H_{15}O_{5}N_{1}S_{1}$
		237.0664	5.2	1.2	$C_{14}H_{9}O_{2}N_{2}$
		237.0698	-9.0	-2.1	$C_{11}H_{13}O_{2}N_{2}S_{1}$
		237.0704	-11.8	-2.8	$C_{19}H_{9}$
		237.0637	16.5	3.9	$C_{11}H_{11}O_{5}N_{1}$
242.9851	0.8016	242.9863	-4.9	-1.2	$C_{4}H_{5}O_{11}N_{1}$
		242.9838	5.4	1.3	$C_{8}H_{5}O_{6}N_{1}S_{1}$
		242.9864	-5.6	-1.4	$C_{11}H_{3}O_{3}N_{2}S_{1}$
		242.9889	-15.9	-3.9	$C_{7}H_{3}O_{8}N_{2}$
		242.9811	16.5	4.0	$C_{5}H_{7}O_{9}S_{1}$
266.1103	5.2675	266.1096	2.8	0.8	$C_{21}H_{14}$
		266.1114	-4.1	-1.1	$C_{9}H_{18}O_{7}N_{2}$
		266.1089	5.3	1.4	$C_{13}H_{18}O_{2}N_{2}S_{1}$
		266.1129	-9.8	-2.6	$C_{18}H_{18}S_{1}$
		266.1062	15.3	4.1	$C_{10}H_{20}O_{5}N_{1}S_{1}$
280.9825	1.4720	280.9835	-3.5	-1.0	$C_{13}H_{1}O_{6}N_{2}$
		280.9810	5.4	1.5	$C_{17}H_{1}O_{1}N_{2}S_{1}$
		280.9808	6.0	1.7	$C_{10}H_{3}O_{9}N_{1}$
		280.9842	-6.0	-1.7	$C_{7}H_{7}O_{9}N_{1}S_{1}$
		280.9783	14.9	4.2	$C_{14}H_{3}O_{4}N_{1}S_{1}$
292.9827	1.1939	292.9835	-2.6	-0.8	$C_{14}H_{1}O_{6}N_{2}$
		292.9815	4.2	1.2	$C_{5}H_{9}O_{12}S_{1}$
		292.9842	-5.0	-1.5	$C_{8}H_{7}O_{9}N_{1}S_{1}$
		292.9810	5.9	1.7	$C_{18}H_{1}O_{1}N_{2}S_{1}$
		292.9808	6.5	1.9	$C_{11}H_{3}O_{9}N_{1}$
330.9787	1.1706	330.9787	0.1	0.0	$C_{14}H_{5}O_{7}N_{1}S_{1}$
		330.9785	0.6	0.2	$C_{7}H_{7}O_{15}$
		330.9812	-7.5	-2.5	$C_{10}H_{5}O_{12}N_{1}$
		330.9814	-8.0	-2.7	$C_{17}H_{3}O_{4}N_{2}S_{1}$
		330.9760	8.2	2.7	$C_{11}H_{7}O_{10}S_{1}$
380.9678	0.8041	380.9671	1.8	0.7	$C_{21}H_{1}O_{8}$
		380.9690	-3.1	-1.2	$C_{9}H_{5}O_{15}N_{2}$
		380.9665	3.5	1.3	$C_{13}H_{5}O_{10}N_{2}S_{1}$
		380.9663	4.0	1.5	$C_{6}H_{7}O_{18}N_{1}$
		380.9705	-7.1	-2.7	$C_{18}H_{5}O_{8}S_{1}$

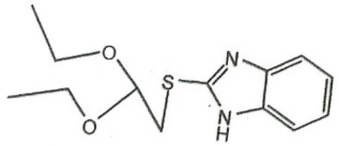

Found = 266.1103

Calc. = 266.1089

$C_{13}H_{18}O_{2}N_{2}S$

File Name : d:\mswin\data\mra-28.mss
Creation Date/Time : 23/07/07 at 15:48:00
File Type : Lo-Res Mass Data (Centroid)
File Source : Acquired on MASPEC system [msw/A091]
File Title : EI
Operator : Barkat Ali
Instrument : MAT312

SCAN GRAPH. Flagging=M/z. Filter=[Int:1%.].
Scan 5-3:26. Entries=52. 100% Int.=42986.

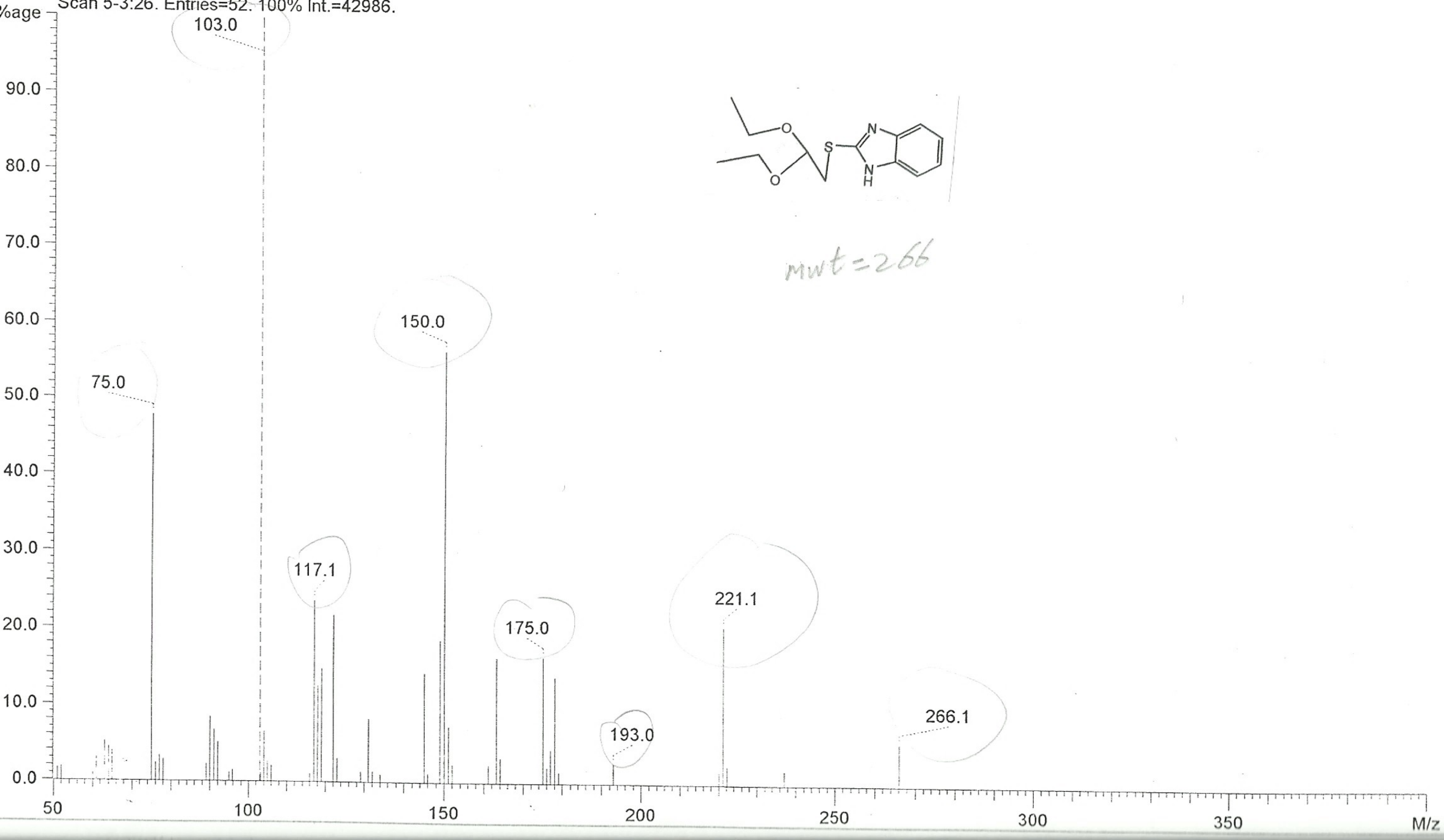

AVANCE AV 300
LAB. No. 115

RAMADAN/DR.EL ASHRY/MRA.29/CDCL3

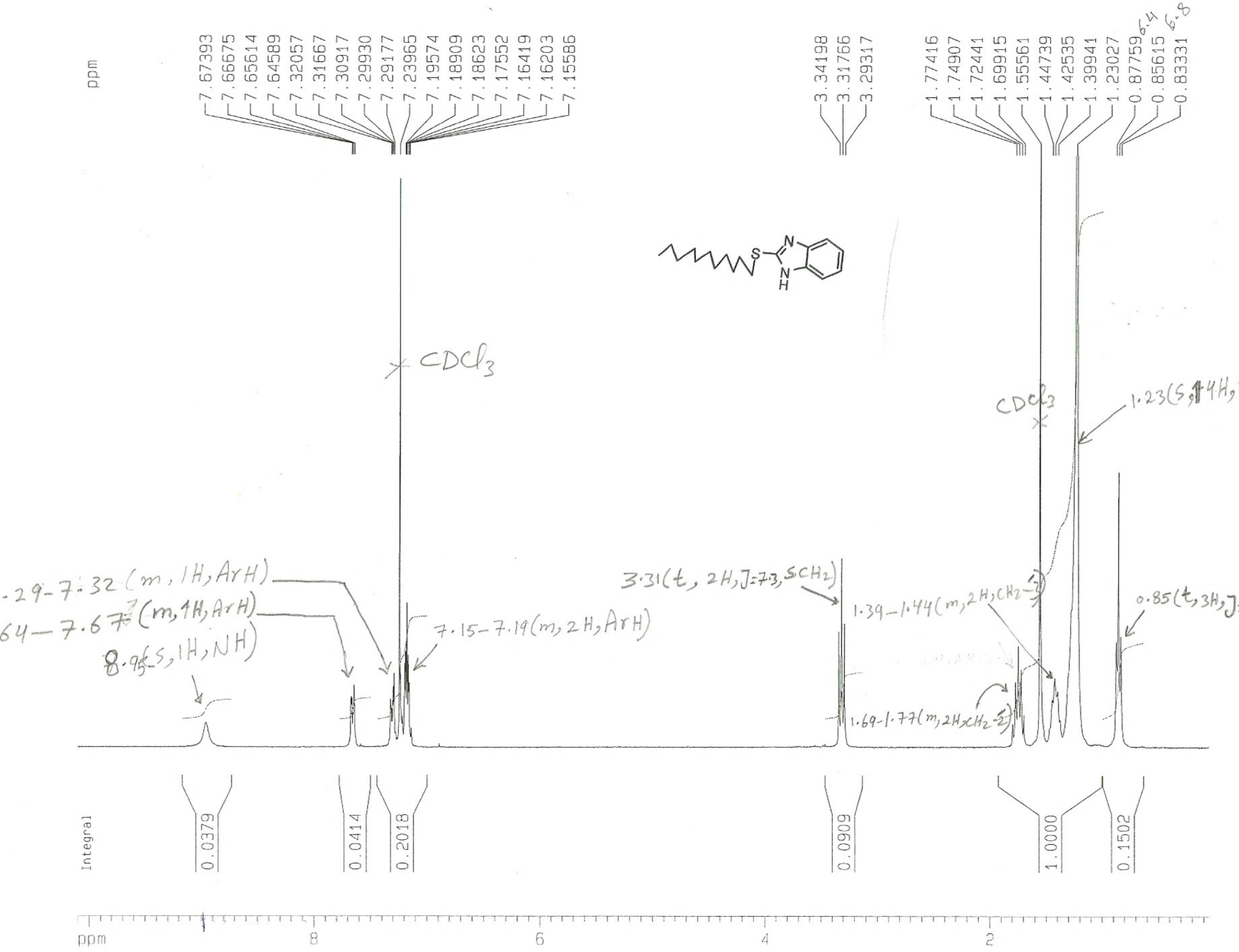

Current Data Parameters	
NAME	aug30
EXPNO	5
PROCNO	1
F2 - Acquisition Parameters	
Date_	20070830
Time	11.29
INSTRUM	spect
PROBHD	5 mm DUL 13C-1
PULPROG	zg30
TD	32768
SOLVENT	CDCl3
NS	128
DS	0
SWH	5995.204 Hz
FIDRES	0.182959 Hz
AQ	2.7329011 sec
RG	1625.5
DW	83.400 usec
DE	10.00 usec
TE	0.0 K
D1	1.00000000 sec
MCREST	0.00000000 sec
MCWRK	0.01500000 sec
======== CHANNEL f1 ========	
NUC1	1H
P1	10.20 usec
PL1	-3.00 dB
SFO1	300.1324010 MHz
F2 - Processing parameters	
SI	16384
SF	300.1300123 MHz
WDW	EM
SSB	0
LB	0.30 Hz
GB	0
PC	1.00
1D NMR plot parameters	
CX	20.00 cm
CY	20.00 cm
F1P	10.101 ppm
F1	3031.49 Hz
F2P	0.053 ppm
F2	15.87 Hz
PPMCM	0.50239 ppm/cm
HZCM	150.78117 Hz/cm

File Name : d:\mswin\data\mra-29.mss
Creation Date/Time : 30/08/07 at 9:36:36
File Type : Lo-Res Mass Data (Centroid)
File Source : Acquired on MASPEC system [msw/A091]
File Title : EI
Operator : Barkat Ali
Instrument : MAT312

SCAN GRAPH. Flagging=M/z. Filter=[Int:1%.].
Scan 7-4:45. Entries=48. 100% Int.=29890.

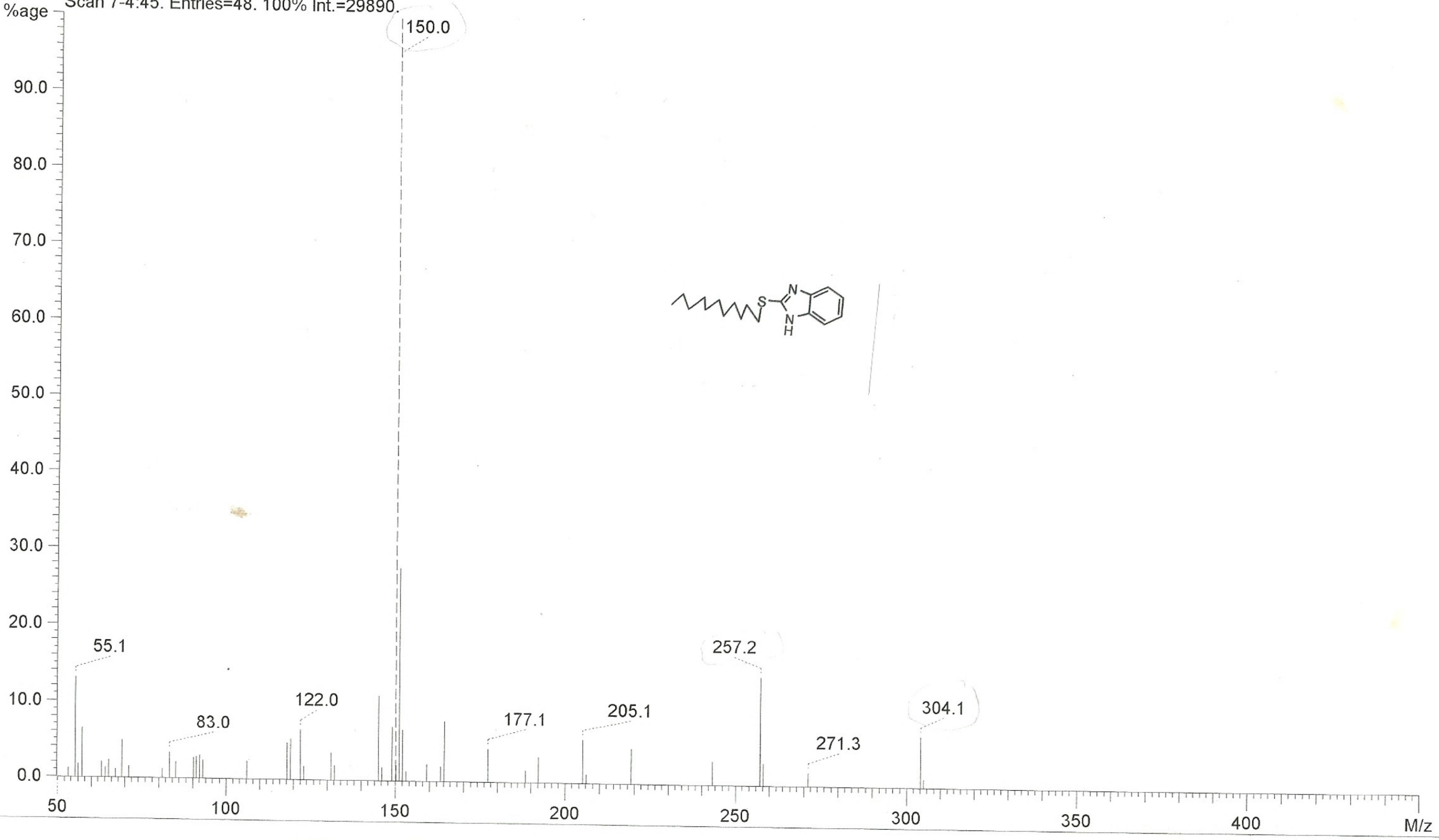

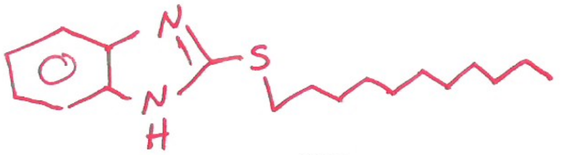

Mass	Relative Intensity	Theoretical Mass	Delta [ppm]	Delta [mmu]	Composition
		280.9808	3.9	1.1	$C_{10} H_3 O_9 N_1$
		280.9835	-5.6	-1.6	$C_{13} H_1 O_6 N_2$
292.9819	2.0460	292.9815	1.4	0.4	$C_5 H_9 O_{12} S_1$
		292.9810	3.2	0.9	$C_{18} H_1 O_1 N_2 S_1$
		292.9808	3.8	1.1	$C_{11} H_3 O_9 N_1$
303.1909	2.7597	303.1920	-3.7	-1.1	$C_{14} H_{27} O_5 N_2$
		303.1895	4.6	1.4	$C_{18} H_{27} N_2 S_1$
		303.1947	-12.5	-3.8	$C_{17} H_{25} O_2 N_3$
304.1980	33.5973	304.1973	2.3	0.7	$C_{18} H_{28} N_2 S_1$
		304.1998	-5.9	-1.8	$C_{14} H_{28} O_5 N_2$
		304.1946	11.1	3.4	$C_{15} H_{30} O_3 N_1 S_1$
305.2030	6.8713	305.2025	1.8	0.5	$C_{15} H_{31} O_3 N_1 S_1$
		305.2018	4.0	1.2	$C_{21} H_{25} N_2$
		305.2051	-7.0	-2.1	$C_{18} H_{29} N_2 S_1$
306.1949	2.1411	306.1943	1.7	0.5	$C_{17} H_{26} O_3 N_2$
		306.1970	-7.1	-2.2	$C_{20} H_{24} N_3$
		306.1977	-9.3	-2.9	$C_{14} H_{30} O_3 N_2 S_1$
330.9787	2.3783	330.9787	0.1	0.0	$C_{14} H_5 O_7 N_1 S_1$
		330.9785	0.6	0.2	$C_7 H_7 O_{15}$
		330.9772	4.7	1.5	$C_5 H_5 O_{14} N_3$
342.9787	1.5577	342.9787	0.1	0.0	$C_{15} H_5 O_7 N_1 S_1$
		342.9785	0.6	0.2	$C_8 H_7 O_{15}$
		342.9772	4.5	1.5	$C_6 H_5 O_{14} N_3$
380.9755	1.7489	380.9757	-0.5	-0.2	$C_{17} H_3 O_{10} N_1$
		380.9759	-1.0	-0.4	$C_{24} H_1 O_2 N_2 S_1$
		380.9750	1.2	0.5	$C_9 H_7 O_{12} N_3 S_1$
392.9755	1.0128	392.9757	-0.5	-0.2	$C_{18} H_3 O_{10} N_1$
		392.9759	-0.9	-0.4	$C_{25} H_1 O_2 N_2 S_1$
		392.9750	1.2	0.5	$C_{10} H_7 O_{12} N_3 S_1$
430.9723	1.2439	430.9721	0.5	0.2	$C_{12} H_5 O_{15} N_3$
		430.9728	-1.1	-0.5	$C_6 H_{11} O_{18} N_2 S_1$
		430.9734	-2.6	-1.1	$C_{14} H_7 O_{16}$

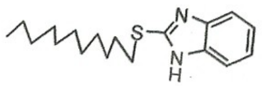

Found = 304.1980

Calc. = 304.1973

$C_{18}H_{28}N_2S$

RAMDAN/DR.EL ASHRY/MRA40
C13 {BB}

400
LAB. No. 117

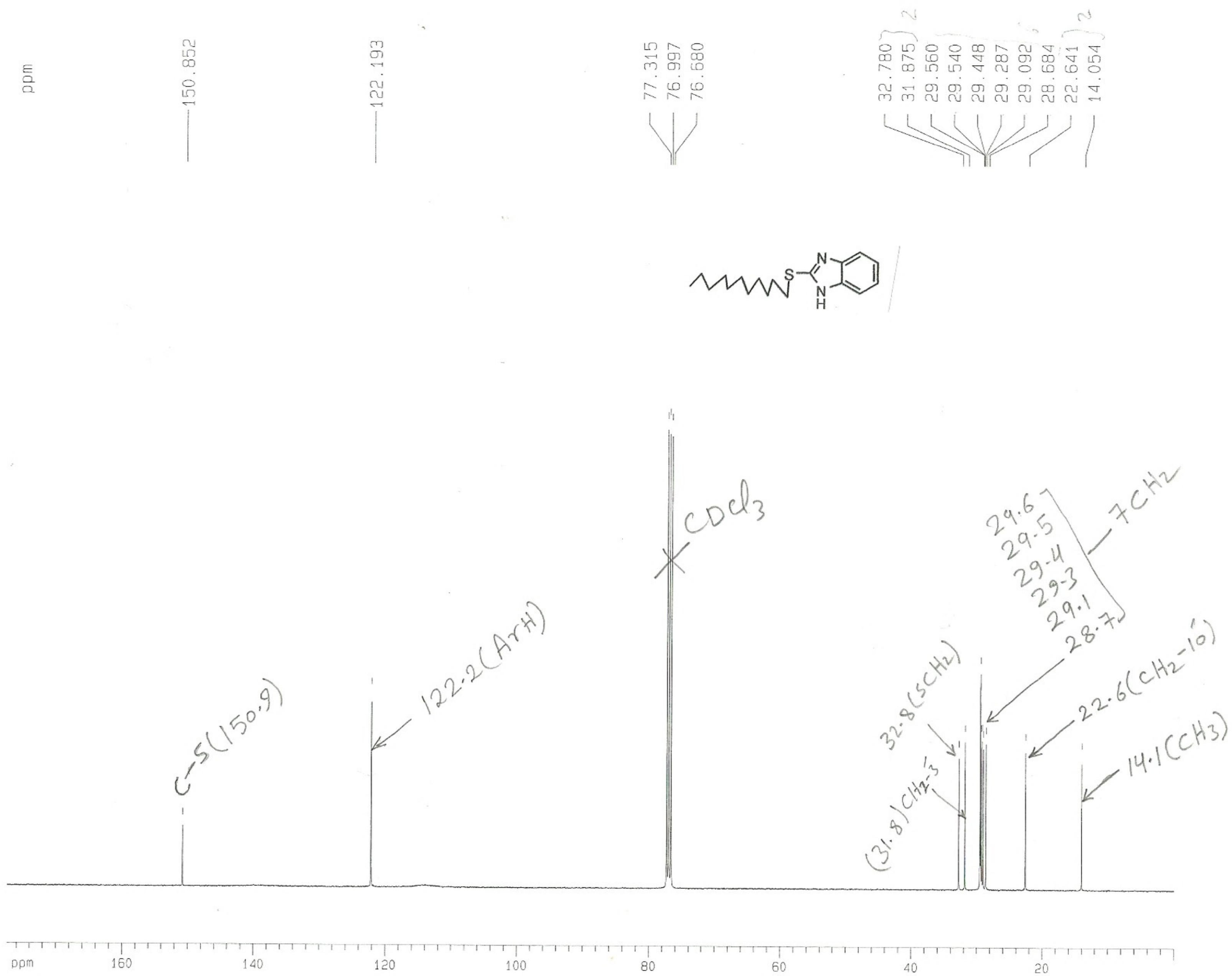

Current Data Parameters
NAME oct06
EXPNO 1
PROCNO 1

F2 - Acquisition Parameters
Date_ 20071006
Time 20.21
INSTRUM spect
PROBHD 5 mm DUL 13C-1
PULPROG zgpg30
TD 32768
SOLVENT CDCl3
NS 15000
DS 2
SWH 23640.662 Hz
FIDRES 0.721456 Hz
AQ 0.6930932 sec
RG 16384
DW 21.150 usec
DE 20.00 usec
TE 300.0 K
D1 1.50000000 sec
d11 0.03000000 sec
d12 0.00002000 sec

======== CHANNEL f1 ========
NUC1 13C
P1 5.50 usec
PL1 0.00 dB
SFO1 100.6242389 MHz

======== CHANNEL f2 ========
CPDPRG2 waltz16
NUC2 1H
PCPD2 100.00 usec
PL2 -3.00 dB
PL12 16.50 dB
PL13 16.50 dB
SFO2 400.1320007 MHz

F2 - Processing parameters
SI 32768
SF 100.6127704 MHz
WDW EM
SSB 0
LB 1.50 Hz
GB 0
PC 1.40

1D NMR plot parameters
CX 20.00 cm
CY 8.00 cm
F1P 177.486 ppm
F1 17857.35 Hz
F2P -0.037 ppm
F2 -3.71 Hz
PPMCM 8.87614 ppm/cm
HZCM 893.05292 Hz/cm

M.RAMADAN/DR.ELO ASHRY/MRA.39/CDCL3+CD3OD

J = 6.3 Hz

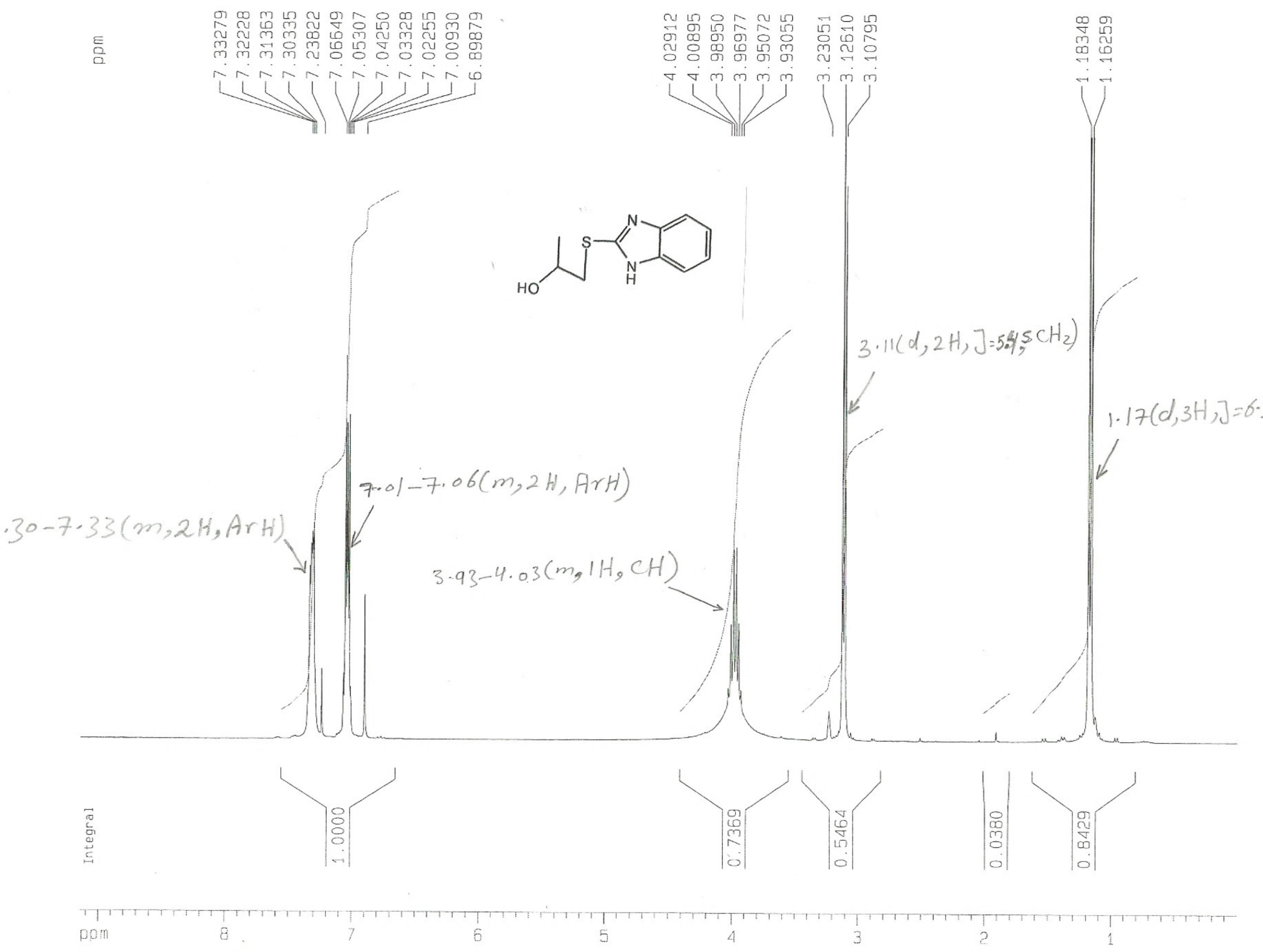

Current Data Parameters

NAME	nov27
EXPNO	8
PROCNO	1

F2 - Acquisition Parameters

Date_	20071127
Time	15.26
INSTRUM	spect
PROBHD	5 mm DUL 13C-1
PULPROG	zg30
TD	32768
SOLVENT	cdcl3
NS	128
DS	0
SWH	5995.204 Hz
FIDRES	0.182959 Hz
AQ	2.7329011 sec
RG	362
DW	83.400 usec
DE	10.00 usec
TE	0.0 K
D1	1.00000000 sec
MCREST	0.00000000 sec
MCWRK	0.01500000 sec

======== CHANNEL f1 ========

NUC1	1H
P1	10.20 usec
PL1	-3.00 dB
SFO1	300.1324010 MHz

F2 - Processing parameters

SI	16384
SF	300.1300123 MHz
WDW	EM
SSB	0
LB	0.30 Hz
GB	0
PC	1.00

1D NMR plot parameters

CX	20.00 cm
CY	16.00 cm
F1P	9.143 ppm
F1	2744.04 Hz
F2P	0.002 ppm
F2	0.71 Hz
PPMCM	0.45702 ppm/cm
HZCM	137.16653 Hz/cm

M.Ramadan/Dr.EL Ashry
Sample: MRA-39

AVANCE AV-300

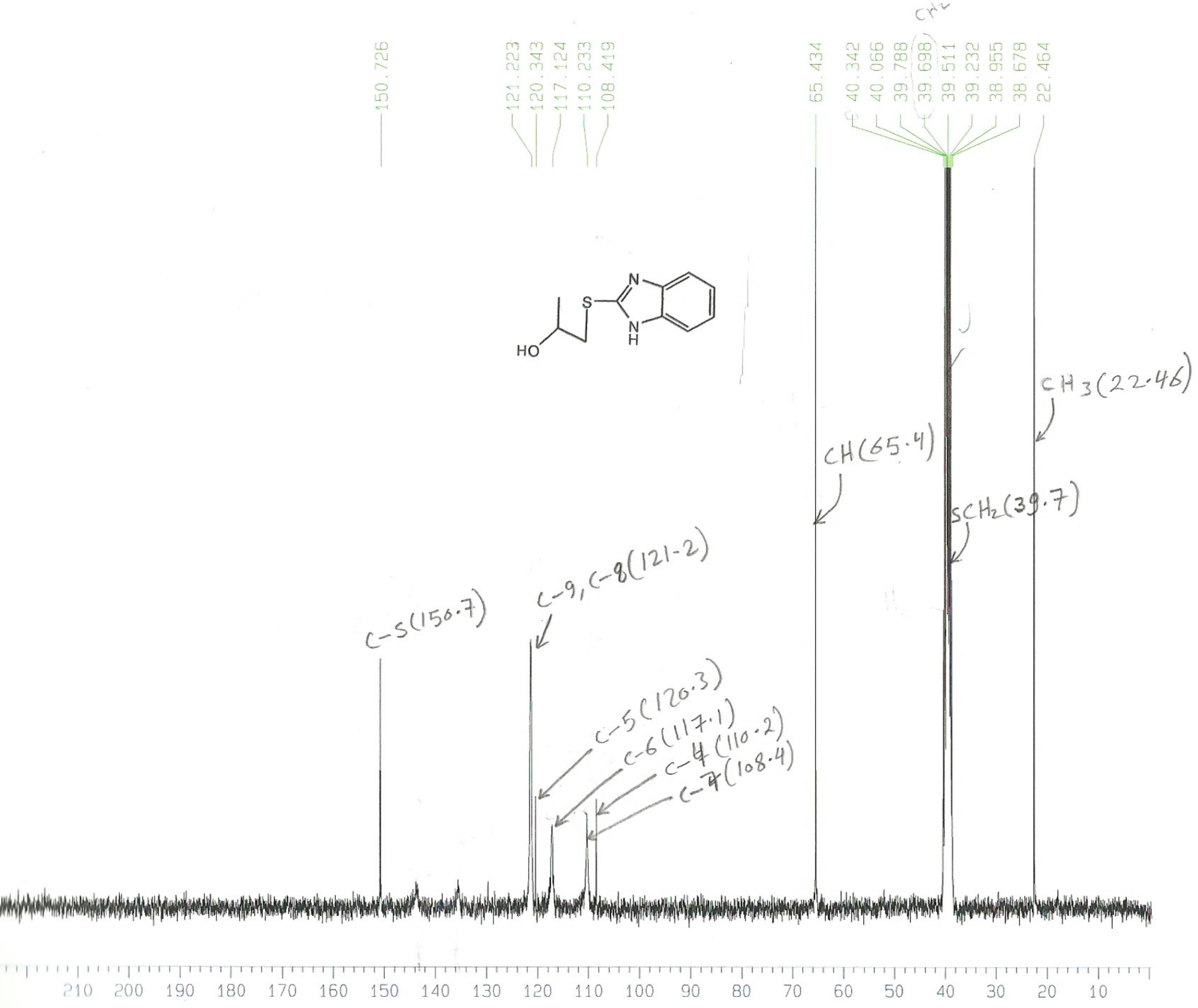

Current Data Parameters

NAME	feb02
EXPNO	1
PROCNO	1

F2 - Acquisition Parameters

Date_	20080202
Time	6.03
INSTRUM	spect
PROBHD	5 mm DUL 13C-1
PULPROG	zgpg
TD	66560
SOLVENT	DMSO
NS	3841
DS	4
SWH	17985.611 Hz
FIDRES	0.270217 Hz
AQ	1.8504180 sec
RG	32768
DW	27.800 usec
DE	6.00 usec
TE	300.1 K
D1	1.50000000 sec
d11	0.03000000 sec
DELTA	1.39999998 sec
MCREST	0.00000000 sec
MCWRK	0.01500000 sec

======== CHANNEL f1 ========

NUC1	13C
P1	6.50 usec
PL1	0.00 dB
SFO1	75.4764278 MHz

======== CHANNEL f2 ========

CPDPRG2	waltz16
NUC2	1H
PCPD2	85.00 usec
PL2	0.00 dB
PL12	19.00 dB
PL13	19.00 dB
SFO2	300.1315007 MHz

F2 - Processing parameters

SI	32768
SF	75.4677867 MHz
WDW	EM
SSB	0
LB	1.50 Hz
GB	0
PC	1.20

1D NMR plot parameters

CX	20.00 cm
CY	35.00 cm
F1P	232.640 ppm
F1	17556.84 Hz
F2P	-0.578 ppm
F2	-43.65 Hz
PPMCM	11.66092 ppm/cm
HZCM	880.02411 Hz/cm

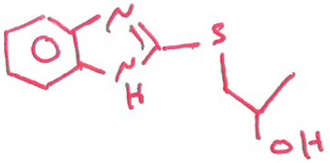

Mass	Relative Intensity	Theoretical Mass	Delta [ppm]	Delta [mmu]	Composition
		165.0426	-16.9	-2.8	$C_{8} H_{7} O_{3} N_{1}$
166.0351	2.9296	166.0352	-0.4	-0.1	$C_{4} H_{8} O_{6} N_{1}$
		166.0327	14.6	2.4	$C_{8} H_{8} O_{1} N_{1} S_{1}$
		166.0378	-16.6	-2.8	$C_{7} H_{6} O_{3} N_{2}$
168.9883	9.9992	168.9885	-1.6	-0.3	$C_{5} H_{1} O_{5} N_{2}$
		168.9860	13.2	2.2	$C_{9} H_{1} N_{2} S_{1}$
		168.9859	14.3	2.4	$C_{2} H_{3} O_{8} N_{1}$
175.0310	13.2998	175.0303	4.2	0.7	$C_{6} H_{9} O_{3} N_{1} S_{1}$
		175.0296	8.1	1.4	$C_{12} H_{3} N_{2}$
		175.0330	-11.2	-2.0	$C_{9} H_{7} N_{2} S_{1}$
176.0358	1.3652	176.0348	5.9	1.0	$C_{9} H_{6} O_{3} N_{1}$
		176.0374	-9.4	-1.6	$C_{12} H_{4} N_{2}$
		176.0381	-13.3	-2.3	$C_{6} H_{10} O_{3} N_{1} S_{1}$
180.9883	8.9156	180.9885	-1.5	-0.3	$C_{6} H_{1} O_{5} N_{2}$
		180.9860	12.3	2.2	$C_{10} H_{1} N_{2} S_{1}$
		180.9859	13.3	2.4	$C_{3} H_{3} O_{8} N_{1}$
189.0482	1.3990	189.0486	-2.2	-0.4	$C_{10} H_{9} N_{2} S_{1}$
		189.0460	12.0	2.3	$C_{7} H_{11} O_{3} N_{1} S_{1}$
		189.0511	-15.4	-2.9	$C_{6} H_{9} O_{5} N_{2}$
190.0575	8.0239	190.0565	5.5	1.1	$C_{10} H_{10} N_{2} S_{1}$
		190.0590	-7.6	-1.4	$C_{6} H_{10} O_{5} N_{2}$
		190.0538	19.6	3.7	$C_{7} H_{12} O_{3} N_{1} S_{1}$
191.0646	10.8718	191.0643	1.7	0.3	$C_{10} H_{11} N_{2} S_{1}$
		191.0668	-11.4	-2.2	$C_{6} H_{11} O_{5} N_{2}$
		191.0616	15.7	3.0	$C_{7} H_{13} O_{3} N_{1} S_{1}$
192.0670	1.4324	192.0661	5.0	1.0	$C_{10} H_{10} O_{3} N_{1}$
		192.0687	-8.9	-1.7	$C_{13} H_{8} N_{2}$
		192.0694	-12.5	-2.4	$C_{7} H_{14} O_{3} N_{1} S_{1}$
192.9883	1.6587	192.9885	-1.4	-0.3	$C_{7} H_{1} O_{5} N_{2}$
		192.9860	11.6	2.2	$C_{11} H_{1} N_{2} S_{1}$
		192.9859	12.5	2.4	$C_{4} H_{3} O_{8} N_{1}$
193.0425	15.0936	193.0436	-5.4	-1.0	$C_{9} H_{9} O_{1} N_{2} S_{1}$
		193.0409	8.5	1.6	$C_{6} H_{11} O_{4} N_{1} S_{1}$
		193.0402	12.1	2.3	$C_{12} H_{5} O_{1} N_{2}$
194.0450	1.7111	194.0453	-1.7	-0.3	$C_{9} H_{8} O_{4} N_{1}$
		194.0427	12.1	2.4	$C_{6} H_{10} O_{7}$
		194.0480	-15.5	-3.0	$C_{12} H_{6} O_{1} N_{2}$
207.0597	1.8176	207.0592	2.6	0.5	$C_{10} H_{11} O_{1} N_{2} S_{1}$
		207.0617	-9.5	-2.0	$C_{6} H_{11} O_{6} N_{2}$
		207.0565	15.5	3.2	$C_{7} H_{13} O_{4} N_{1} S_{1}$
208.0677	18.9620	208.0670	3.1	0.6	$C_{10} H_{12} O_{1} N_{2} S_{1}$
		208.0695	-8.9	-1.9	$C_{6} H_{12} O_{6} N_{2}$
		208.0644	16.0	3.3	$C_{7} H_{14} O_{4} N_{1} S_{1}$
209.0703	2.7959	209.0715	-5.5	-1.2	$C_{13} H_{9} O_{1} N_{2}$

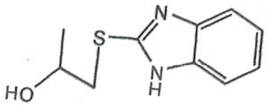

$C_{10} H_{12} O N_2 S$

found = 208.0677

calc = 208.0670

File Name	:	d:\mswin\data\mra39.mss
Creation Date/Time	:	25/10/07 at 11:37:23
File Type	:	Lo-Res Mass Data (Centroid)
File Source	:	Acquired on MASPEC system [msw/A091]
File Title	:	EI
Operator	:	Barkat Ali
Instrument	:	MAT312

SCAN GRAPH. Flagging=M/z. Filter=[Int:1%.].
Scan 18-12:17. Entries=39. 100% Int.=32224.

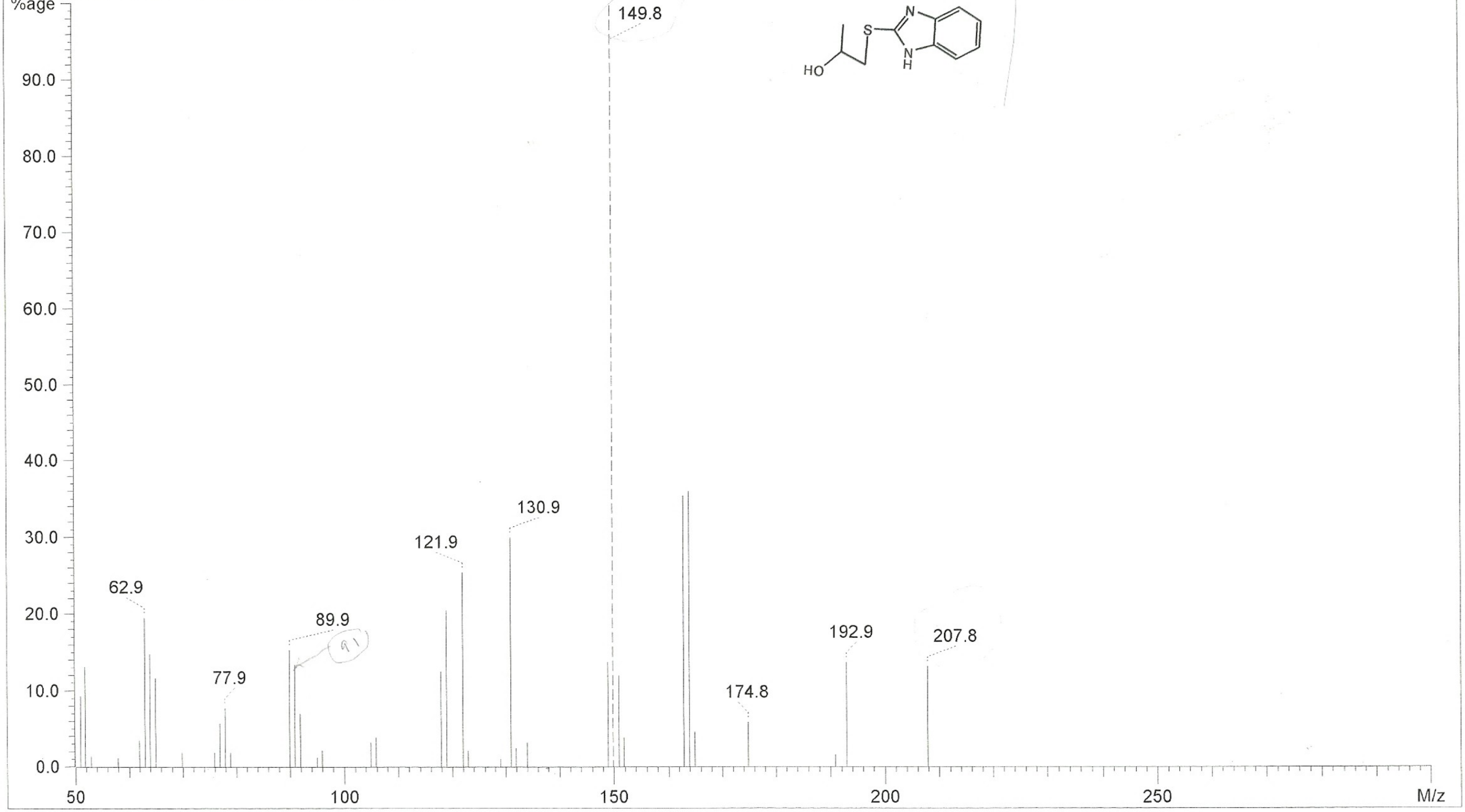

RAMADAN/DR.EL ASHRY/MRA.42/DMSO

OK

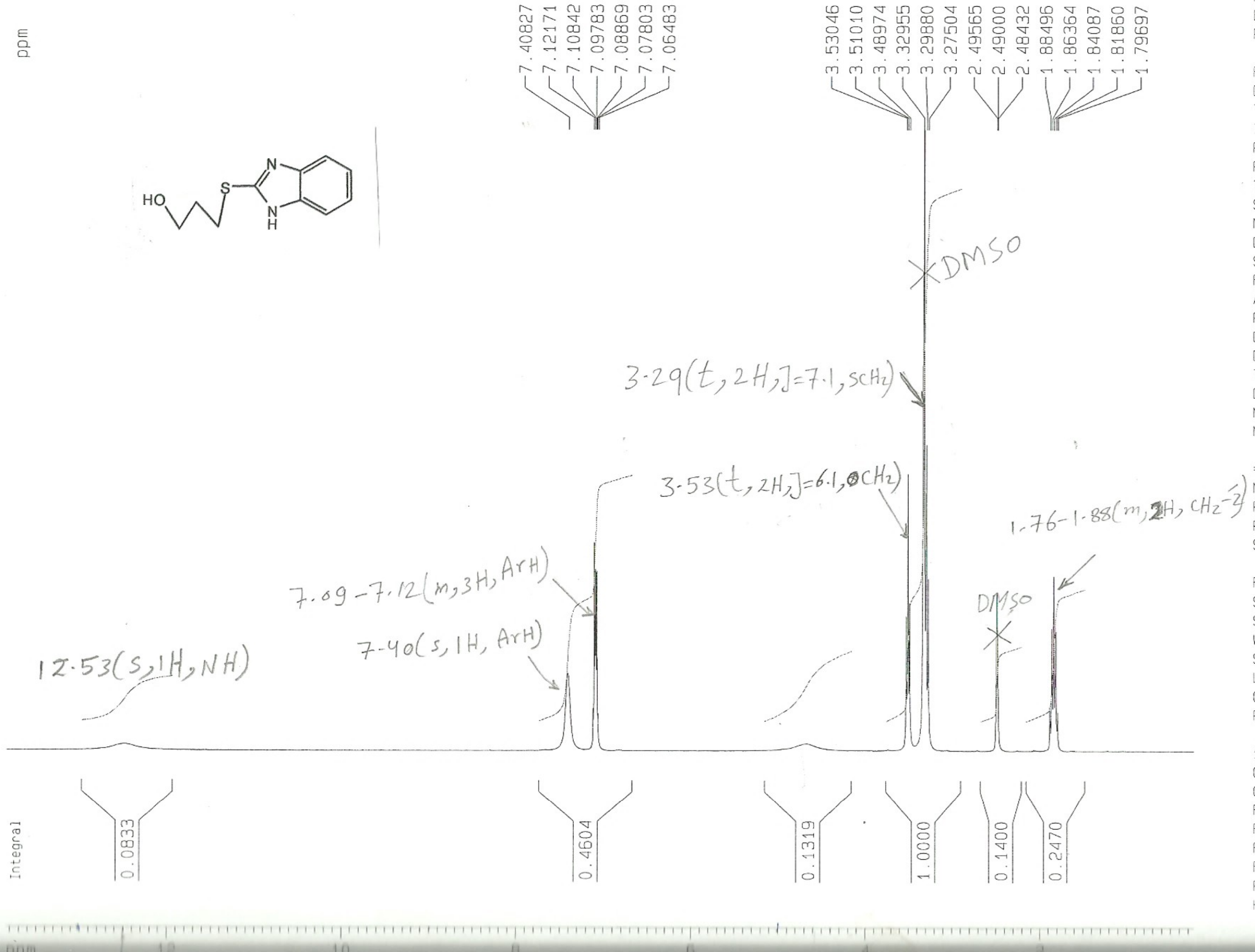

Current Data Parameters

NAME	oct12
EXPNO	8
PROCNO	1

F2 - Acquisition Parameters

Date_	20071012
Time	10.54
INSTRUM	spect
PROBHD	5 mm DUL 13C-1
PULPROG	zg30
TD	32768
SOLVENT	DMSO
NS	128
DS	0
SWH	5995.204 Hz
FIDRES	0.182959 Hz
AQ	2.7329011 sec
RG	724.1
DW	83.400 usec
DE	10.00 usec
TE	0.0 K
D1	1.00000000 sec
MCREST	0.00000000 sec
MCWRK	0.01500000 sec

======== CHANNEL f1 ========

NUC1	1H
P1	10.20 usec
PL1	-3.00 dB
SFO1	300.1324010 MHz

F2 - Processing parameters

SI	16384
SF	300.1300042 MHz
WDW	EM
SSB	0
LB	0.30 Hz
GB	0
PC	1.00

1D NMR plot parameters

CX	20.00 cm
CY	16.00 cm
F1P	13.842 ppm
F1	4154.30 Hz
F2P	0.236 ppm
F2	70.79 Hz
PPMCM	0.68029 ppm/cm
HZCM	204.17572 Hz/cm

M.Ramadan/Dr.EL Ashry
Sample: MRA-42

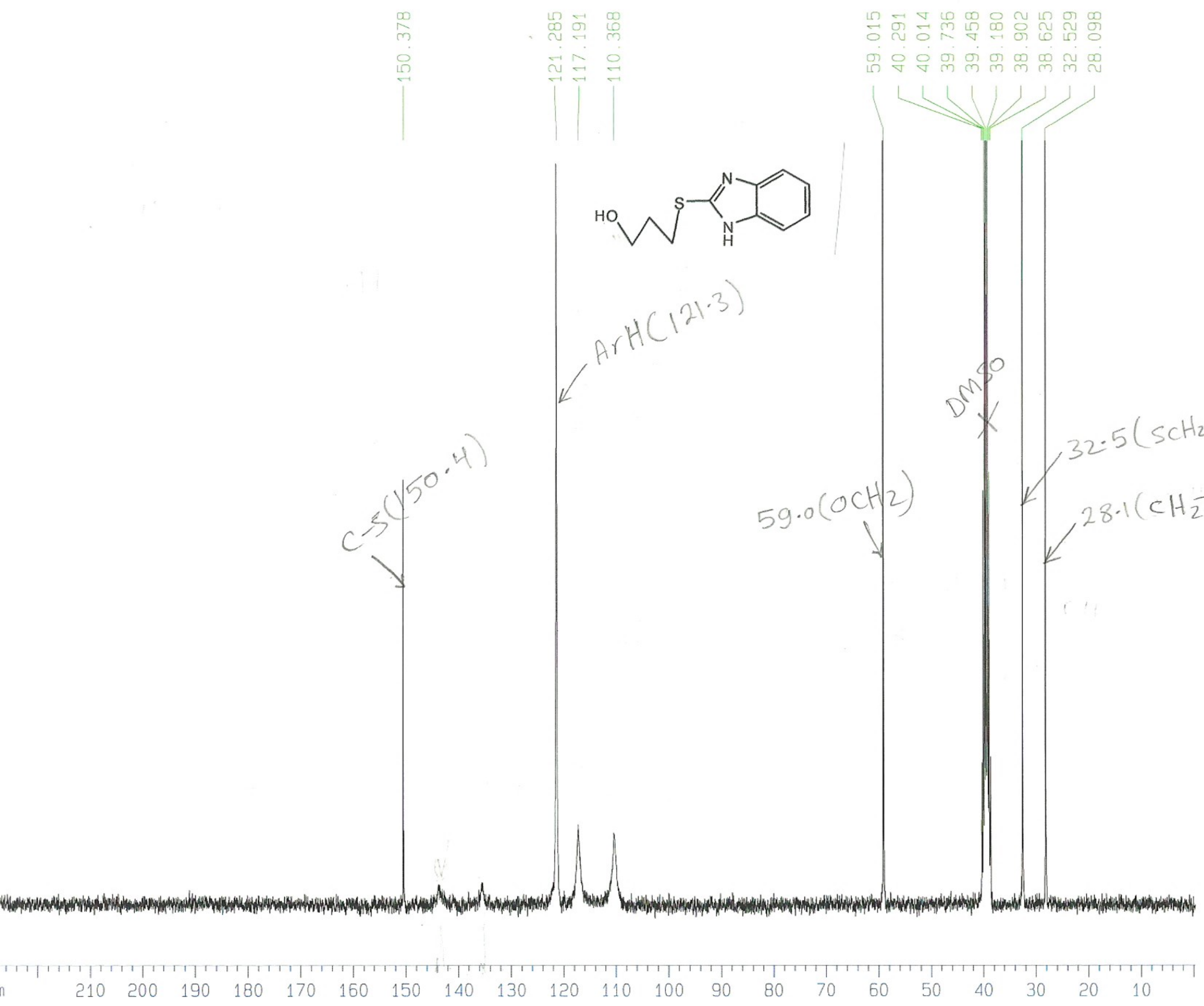

Current Data Parameters
NAME feb01
EXPNO 1
PROCNO 1

F2 - Acquisition Parameters
Date_ 20080201
Time 5.37
INSTRUM spect
PROBHD 5 mm DUL 13C-1
PULPROG zgpg
TD 66560
SOLVENT DMSO
NS 1703
DS 4
SWH 17985.611 Hz
FIDRES 0.270217 Hz
AQ 1.8504180 sec
RG 32768
DW 27.800 usec
DE 6.00 usec
TE 300.0 K
D1 1.50000000 sec
d11 0.03000000 sec
DELTA 1.39999998 sec
MCREST 0.00000000 sec
MCWRK 0.01500000 sec

======== CHANNEL f1 ========
NUC1 13C
P1 6.50 usec
PL1 0.00 dB
SFO1 75.4764278 MHz

======== CHANNEL f2 ========
CPDPRG2 waltz16
NUC2 1H
PCPD2 85.00 usec
PL2 0.00 dB
PL12 19.00 dB
PL13 19.00 dB
SFO2 300.1315007 MHz

F2 - Processing parameters
SI 32768
SF 75.4677867 MHz
WDW EM
SSB 0
LB 1.50 Hz
GB 0
PC 1.20

1D NMR plot parameters
CX 20.00 cm
CY 35.00 cm
F1P 232.130 ppm
F1 17518.32 Hz
F2P -0.323 ppm
F2 -24.39 Hz
PPMCM 11.62265 ppm/cm
HZCM 877.13550 Hz/cm

Mass	Relative Intensity	Theoretical Mass	Delta [ppm]	Delta [mmu]	Composition
		167.0279	21.0	3.5	$C_7 H_7 O_1 N_2 S_1$
168.9877	10.8575	168.9885	-5.2	-0.9	$C_5 H_1 O_5 N_2$
		168.9860	9.6	1.6	$C_9 H_1 N_2 S_1$
		168.9859	10.7	1.8	$C_2 H_3 O_8 N_1$
175.0323	5.0897	175.0330	-4.0	-0.7	$C_9 H_7 N_2 S_1$
		175.0303	11.4	2.0	$C_6 H_9 O_3 N_1 S_1$
		175.0296	15.3	2.7	$C_{12} H_3 N_2$
177.0472	24.6104	177.0460	7.1	1.3	$C_6 H_{11} O_3 N_1 S_1$
		177.0486	-8.0	-1.4	$C_9 H_9 N_2 S_1$
		177.0453	11.0	2.0	$C_{12} H_5 N_2$
178.0499	2.4079	178.0504	-2.7	-0.5	$C_9 H_8 O_3 N_1$
		178.0477	12.3	2.2	$C_6 H_{10} O_6$
		178.0531	-17.8	-3.2	$C_{12} H_6 N_2$
179.0459	1.2564	179.0457	1.5	0.3	$C_8 H_7 O_3 N_2$
		179.0430	16.4	2.9	$C_5 H_9 O_6 N_1$
		179.0490	-17.4	-3.1	$C_5 H_{11} O_3 N_2 S_1$
180.9883	8.9569	180.9885	-1.5	-0.3	$C_6 H_1 O_5 N_2$
		180.9860	12.3	2.2	$C_{10} H_1 N_2 S_1$
		180.9859	13.3	2.4	$C_3 H_3 O_8 N_1$
189.0517	3.9066	189.0511	2.9	0.6	$C_6 H_9 O_5 N_2$
		189.0486	16.2	3.1	$C_{10} H_9 N_2 S_1$
		189.0552	-18.4	-3.5	$C_{11} H_9 O_3$
190.0590	75.1682	190.0590	0.2	0.0	$C_6 H_{10} O_5 N_2$
		190.0565	13.4	2.5	$C_{10} H_{10} N_2 S_1$
		190.0630	-20.9	-4.0	$C_{11} H_{10} O_3$
191.0609	10.5608	191.0609	-0.2	-0.0	$C_{13} H_7 N_2$
		191.0616	-3.8	-0.7	$C_7 H_{13} O_3 N_1 S_1$
		191.0582	13.9	2.6	$C_{10} H_9 O_3 N_1$
192.0550	4.0965	192.0535	7.9	1.5	$C_9 H_8 O_3 N_2$
		192.0569	-9.7	-1.9	$C_6 H_{12} O_3 N_2 S_1$
		192.0575	-13.0	-2.5	$C_{14} H_8 O_1$
192.9883	1.5055	192.9885	-1.4	-0.3	$C_7 H_1 O_5 N_2$
		192.9860	11.6	2.2	$C_{11} H_1 N_2 S_1$
		192.9859	12.5	2.4	$C_4 H_3 O_8 N_1$
193.0469	1.3855	193.0461	4.4	0.8	$C_5 H_9 O_6 N_2$
		193.0501	-16.4	-3.2	$C_{10} H_9 O_4$
		193.0436	17.4	3.3	$C_9 H_9 O_1 N_2 S_1$
207.0598	1.2227	207.0592	2.8	0.6	$C_{10} H_{11} O_1 N_2 S_1$
		207.0617	-9.3	-1.9	$C_6 H_{11} O_6 N_2$
		207.0565	15.7	3.3	$C_7 H_{13} O_4 N_1 S_1$
208.0679	41.9321	208.0670	4.1	0.8	$C_{10} H_{12} O_1 N_2 S_1$
		208.0695	-8.0	-1.7	$C_6 H_{12} O_6 N_2$
		208.0644	16.9	3.5	$C_7 H_{14} O_4 N_1 S_1$
209.0715	4.8150	209.0715	0.3	0.1	$C_{13} H_9 O_1 N_2$

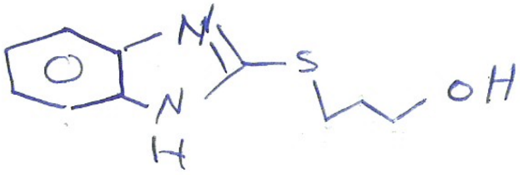

$C_{10}H_{12}N_2OS$

found: 208.0679

calc.: 208.0670

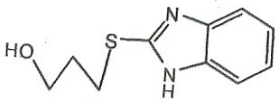

File Name	:	d:\mswin\data\mra-42.mss
Creation Date/Time	:	25/10/07 at 12:02:10
File Type	:	Lo-Res Mass Data (Centroid)
File Source	:	Acquired on MASPEC system [msw/A091]
File Title	:	EI
Operator	:	Barkat Ali
Instrument	:	MAT312

SCAN GRAPH. Flagging=M/z. Filter=[Int:1%.].
Scan 12-8:11. Entries=41. 100% Int.=19327.

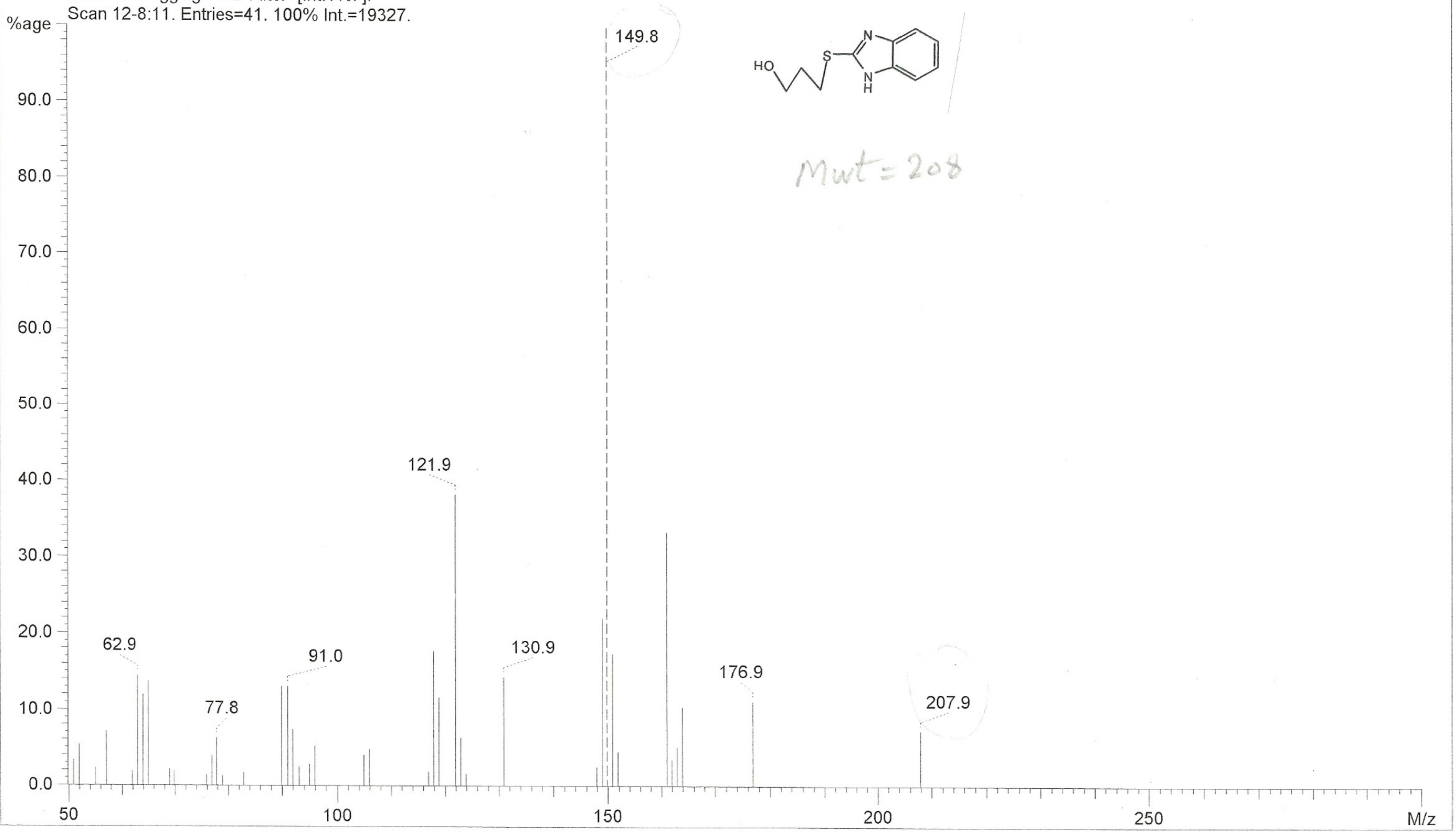

inting Time: 10:55:06 AM
inting Date: Saturday, April 04, 2009

*Installation
*QStar XL Ser. no. Y3200502

	Formula	Calculated m/z (amu)	mDa Error	PPM Error	DBE
1	C8 H13 N4 S2	229.0576	-1.6165	-7.0575	4.5
2	C11 H9 N4 S	229.0542	1.7553	7.6635	9.5

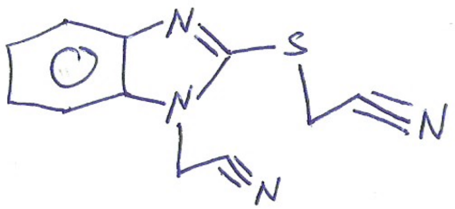

Found = 229.0542

Calc = 228.0470 + 1.0078 = 229.0548

File Name	:	d:\mswin\data\mra-54.mss
Creation Date/Time	:	30/08/07 at 9:21:58
File Type	:	Lo-Res Mass Data (Centroid)
File Source	:	Acquired on MASPEC system [msw/A091]
File Title	:	EI
Operator	:	Barkat Ali
Instrument	:	MAT312

SCAN GRAPH. Flagging=M/z. Filter=[Int:1%.].
Scan 9-6:07. Entries=106. 100% Int.=95948.

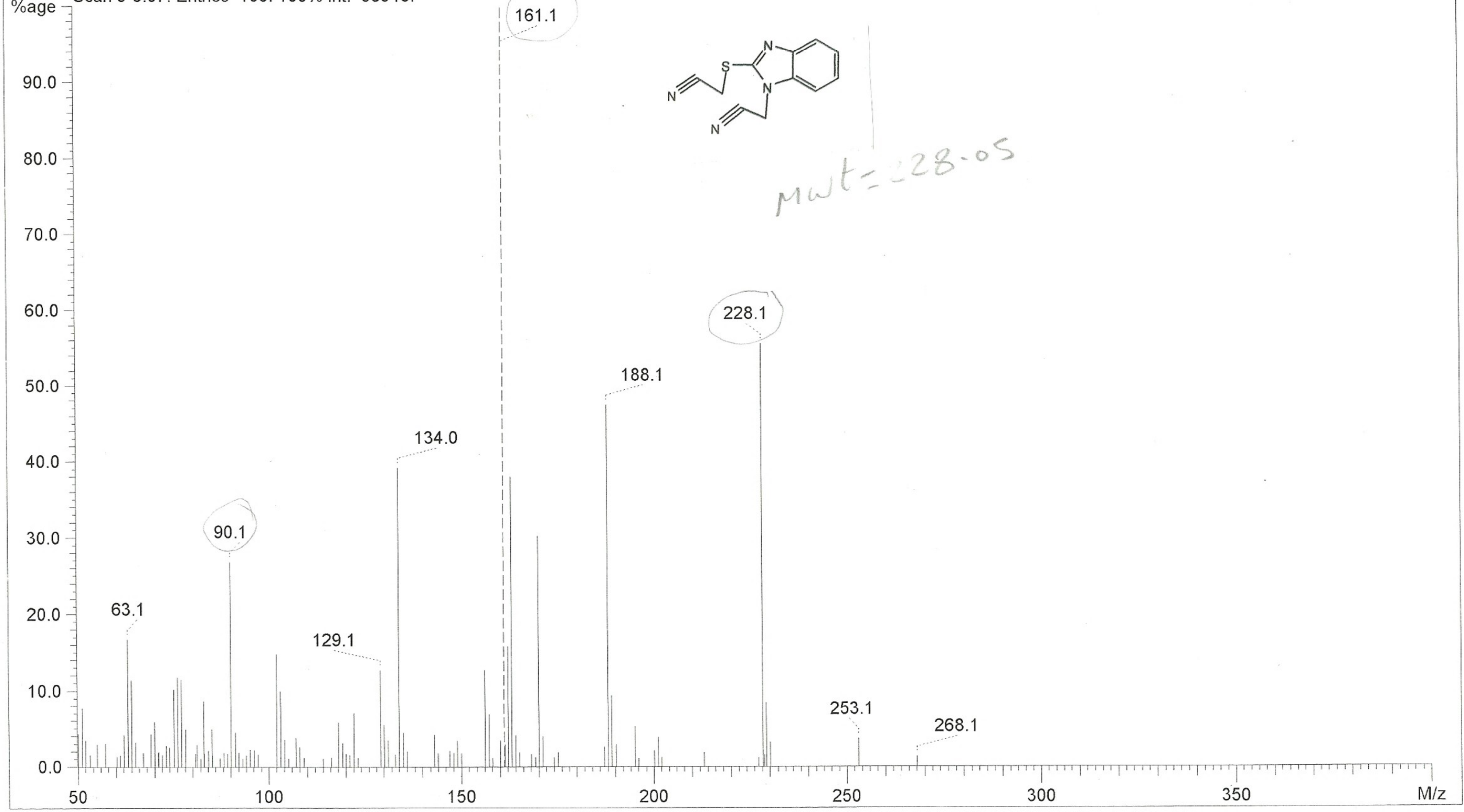

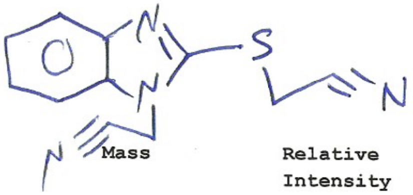

Mass	Relative Intensity	Theoretical Mass	Delta [ppm]	Delta [mmu]	Composition
		165.0201	30.9	5.1	$C_9 H_1 N_4$
167.0347	5.9155	167.0358	-6.6	-1.1	$C_9 H_3 N_4$
		167.0391	-26.8	-4.5	$C_6 H_7 N_4 S_1$
167.1849	0.6070	167.1800	29.5	4.9	$C_{12} H_{23}$
168.0351	0.3359				
168.0539	0.4649	168.0562	-13.3	-2.2	$C_{10} H_6 N_3$
		168.0595	-33.4	-5.6	$C_7 H_{10} N_3 S_1$
168.9874	22.2824	168.9860	7.8	1.3	$C_9 H_1 N_2 S_1$
169.1923	0.5474				
169.1972	0.7056				
169.2000	0.6125				
169.9900	0.9499	169.9939	-23.0	-3.9	$C_9 H_2 N_2 S_1$
170.0717	8.0047	170.0718	-0.9	-0.2	$C_{10} H_8 N_3$
		170.0752	-20.7	-3.5	$C_7 H_{12} N_3 S_1$
171.0769	1.2387	171.0796	-16.3	-2.8	$C_{10} H_9 N_3$
175.0342	0.2389	175.0330	6.9	1.2	$C_9 H_7 N_2 S_1$
		175.0296	26.1	4.6	$C_{12} H_3 N_2$
180.9883	19.5915	180.9860	12.3	2.2	$C_{10} H_1 N_2 S_1$
181.9903	0.6412	181.9939	-19.6	-3.6	$C_{10} H_2 N_2 S_1$
185.9864	0.4896				
187.0172	0.7018	187.0170	0.9	0.2	$C_{12} H_1 N_3$
		187.0204	-17.2	-3.2	$C_9 H_5 N_3 S_1$
188.0275	15.6202	188.0282	-3.7	-0.7	$C_9 H_6 N_3 S_1$
		188.0249	14.2	2.7	$C_{12} H_2 N_3$
189.0327	2.8764	189.0327	0.0	0.0	$C_{12} H_3 N_3$
		189.0361	-17.8	-3.4	$C_9 H_7 N_3 S_1$
192.9883	4.0500	192.9860	11.6	2.2	$C_{11} H_1 N_2 S_1$
195.0702	2.1684	195.0704	-1.4	-0.3	$C_8 H_{11} N_4 S_1$
		195.0671	15.9	3.1	$C_{11} H_7 N_4$
200.0316	0.5588	200.0282	16.5	3.3	$C_{10} H_6 N_3 S_1$
		200.0374	-29.5	-5.9	$C_{14} H_4 N_2$
200.9921	0.8675				
201.0383	1.3276	201.0361	11.1	2.2	$C_{10} H_7 N_3 S_1$
		201.0327	27.9	5.6	$C_{13} H_3 N_3$
204.9883	2.5056	204.9860	10.9	2.2	$C_{12} H_1 N_2 S_1$
211.2381	0.5112				
211.9746	0.5724	211.9721	11.9	2.5	$C_{15} S_1$
212.9907	0.2267				
216.9898	1.3374	216.9860	17.5	3.8	$C_{13} H_1 N_2 S_1$
218.9871	10.5598	218.9891	-9.3	-2.0	$C_{12} H_1 N_3 S_1$
228.0498	21.3361	228.0470	12.5	2.9	$C_{11} H_8 N_4 S_1$
228.9909	0.7058	228.9860	21.3	4.9	$C_{14} H_1 N_2 S_1$
229.0573	4.4182	229.0548	10.8	2.5	$C_{11} H_9 N_4 S_1$
		229.0514	25.6	5.9	$C_{14} H_5 N_4$

found

Calc.

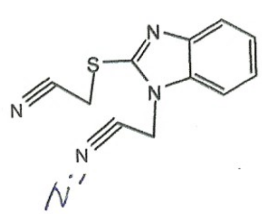

Found = 228.0498

calc. = 228.0470

$C_{11}H_8N_4S$

RAMADAN/DR.EL ASHRY/MRA.54/DMSO

c13 {BB}

AVANCE AV 300
LAB. No. 115

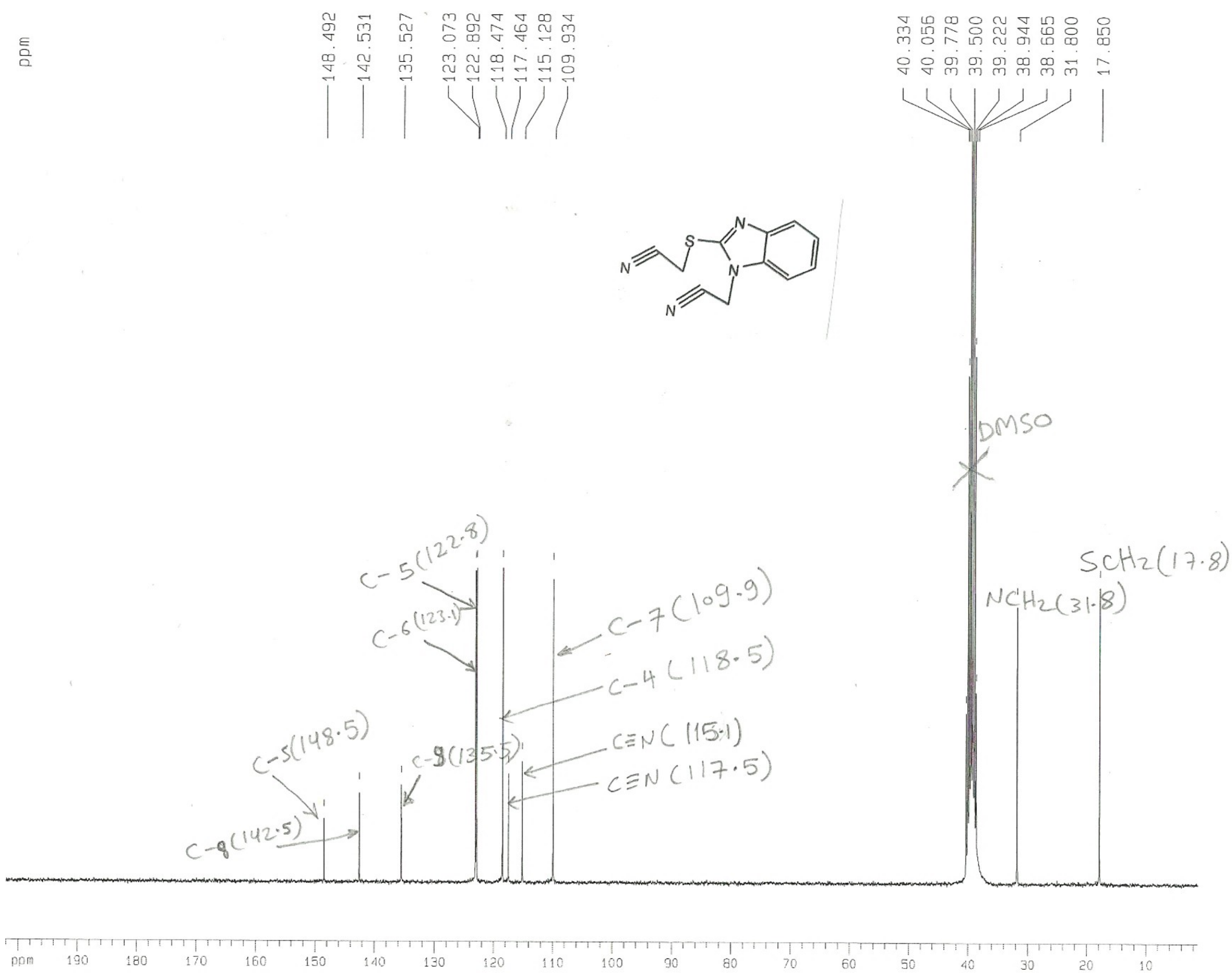

Current Data Parameters

NAME	oct06
EXPNO	10
PROCNO	1

F2 - Acquisition Parameters

Date_	20071007
Time	10.50
INSTRUM	spect
PROBHD	5 mm DUL 13C-1
PULPROG	zgpg30
TD	65536
SOLVENT	DMSO
NS	24948
DS	2
SWH	18518.518 Hz
FIDRES	0.282570 Hz
AQ	1.7695220 sec
RG	16384
DW	27.000 usec
DE	10.00 usec
TE	0.0 K
D1	1.50000000 sec
d11	0.03000000 sec
DELTA	1.39999998 sec
MCREST	0.00000000 sec
MCWRK	0.01500000 sec

======== CHANNEL f1 ========

NUC1	13C
P1	14.00 usec
PL1	-6.00 dB
SFO1	75.4764278 MHz

======== CHANNEL f2 ========

CPDPRG2	waltz16
NUC2	1H
PCPD2	100.00 usec
PL2	-3.00 dB
PL12	20.00 dB
PL13	20.00 dB
SFO2	300.1312005 MHz

F2 - Processing parameters

SI	32768
SF	75.4677869 MHz
WDW	EM
SSB	0
LB	1.50 Hz
GB	0
PC	1.40

1D NMR plot parameters

CX	20.00 cm
CY	20.00 cm
F1P	202.028 ppm
F1	15246.61 Hz
F2P	1.215 ppm
F2	91.69 Hz
PPMCM	10.04065 ppm/cm
HZCM	757.74573 Hz/cm

RAMADAN/DR.EL ASHRY/MRA.54/DMSO

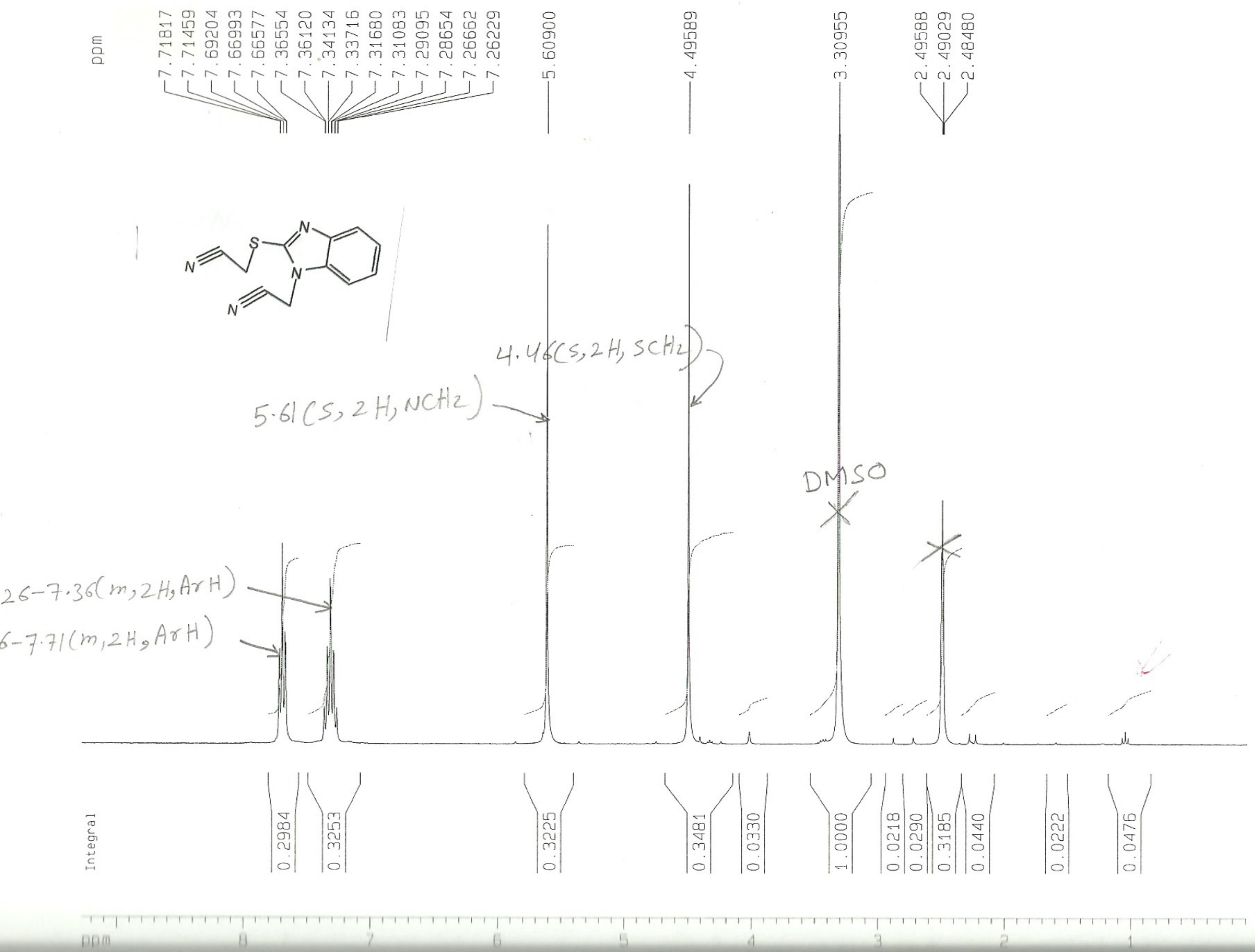

Current Data Parameters
NAME aug30
EXPNO 8
PROCNO 1

F2 - Acquisition Parameters
Date_ 20070830
Time 12.20
INSTRUM spect
PROBHD 5 mm DUL 13C-1
PULPROG zg30
TD 32768
SOLVENT DMSO
NS 128
DS 0
SWH 5995.204 Hz
FIDRES 0.182959 Hz
AQ 2.7329011 sec
RG 1024
DW 83.400 usec
DE 10.00 usec
TE 0.0 K
D1 1.00000000 sec
MCREST 0.00000000 sec
MCWRK 0.01500000 sec

======== CHANNEL f1 ========
NUC1 1H
P1 10.20 usec
PL1 -3.00 dB
SFO1 300.1324010 MHz

F2 - Processing parameters
SI 16384
SF 300.1300042 MHz
WDW EM
SSB 0
LB 0.30 Hz
GB 0
PC 1.00

1D NMR plot parameters
CX 20.00 cm
CY 35.00 cm
F1P 9.275 ppm
F1 2783.71 Hz
F2P 0.088 ppm
F2 26.40 Hz
PPMCM 0.45935 ppm/cm
HZCM 137.86566 Hz/cm

AVANCE AV 300
LAB. No. 116

M.RAMADAN/DR.EL ASHRY/MRA.37/DMSO

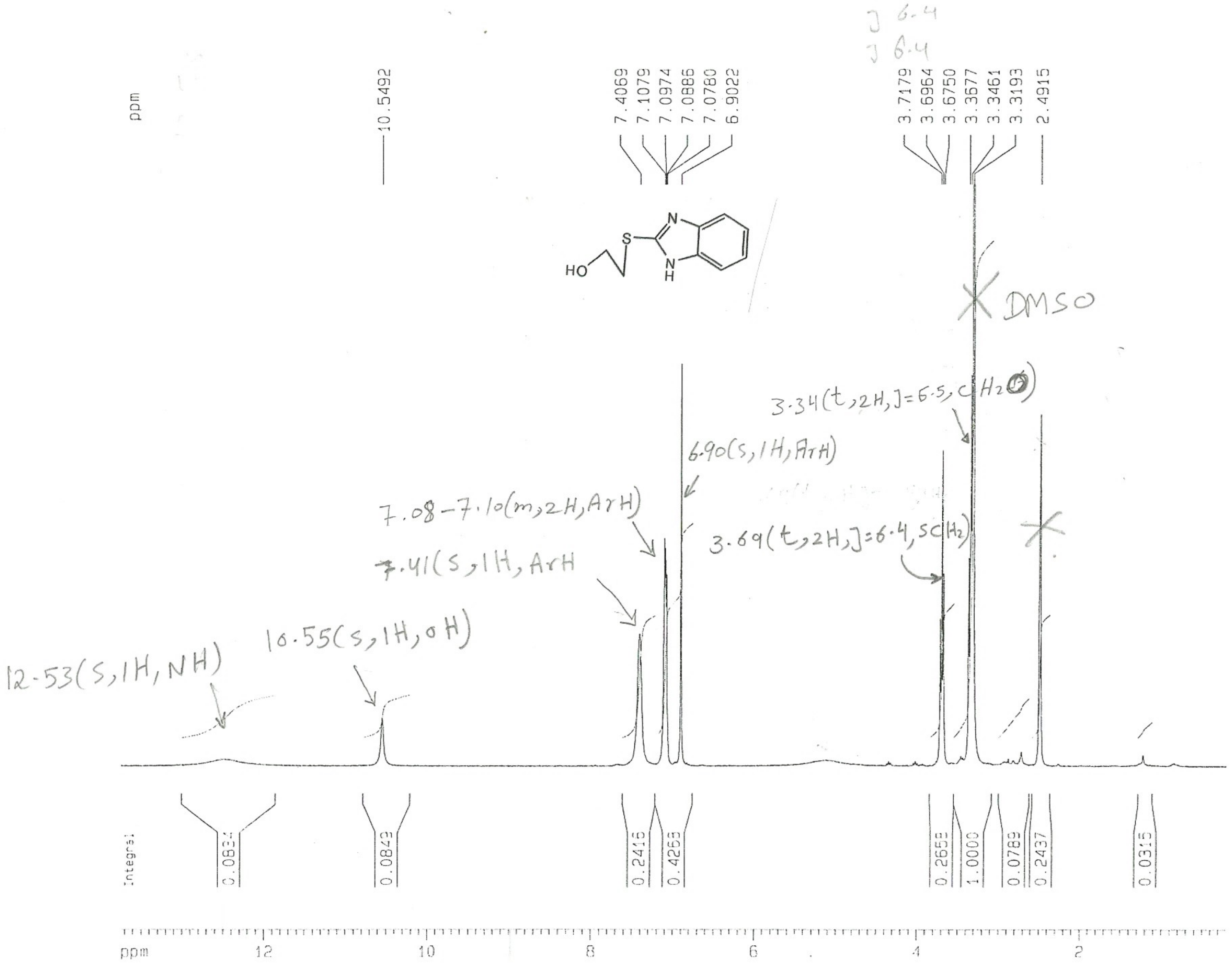

Current Data Parameters
NAME july30
EXPNO 4
PROCNO 1

F2 - Acquisition Parameters
Date_ 20070730
Time 10.05
INSTRUM spect
PROBHD 5 mm DUL 13C-1
PULPROG zg30
TD 32768
SOLVENT DMSO
NS 128
DS 0
SWH 5995.204 Hz
FIDRES 0.182959 Hz
AQ 2.7329011 sec
RG 1149.4
DW 83.400 usec
DE 10.00 usec
TE 0.0 K
D1 1.00000000 sec
MCREST 0.00000000 sec
MCWRK 0.01500000 sec

======== CHANNEL f1 ========
NUC1 1H
P1 10.20 usec
PL1 -3.00 dB
SFO1 300.1324010 MHz

F2 - Processing parameters
SI 16384
SF 300.1300042 MHz
WDW EM
SSB 0
LB 0.30 Hz
GB 0
PC 1.00

1D NMR plot parameters
CX 20.00 cm
CY 30.00 cm
F1P 13.751 ppm
F1 4126.96 Hz
F2P 0.200 ppm
F2 60.07 Hz
PPMCM 0.67752 ppm/cm
HZCM 203.34436 Hz/cm

HEJ

10/4/2007

File: MRA-37
Sample: MOHAMED RAMADAN AMER
Instrument: JEOL MSRoute
Inlet: My Inlet

Date Run: 10-04-2007 (Time Run: 09:04:47)

Ionization mode: EI-

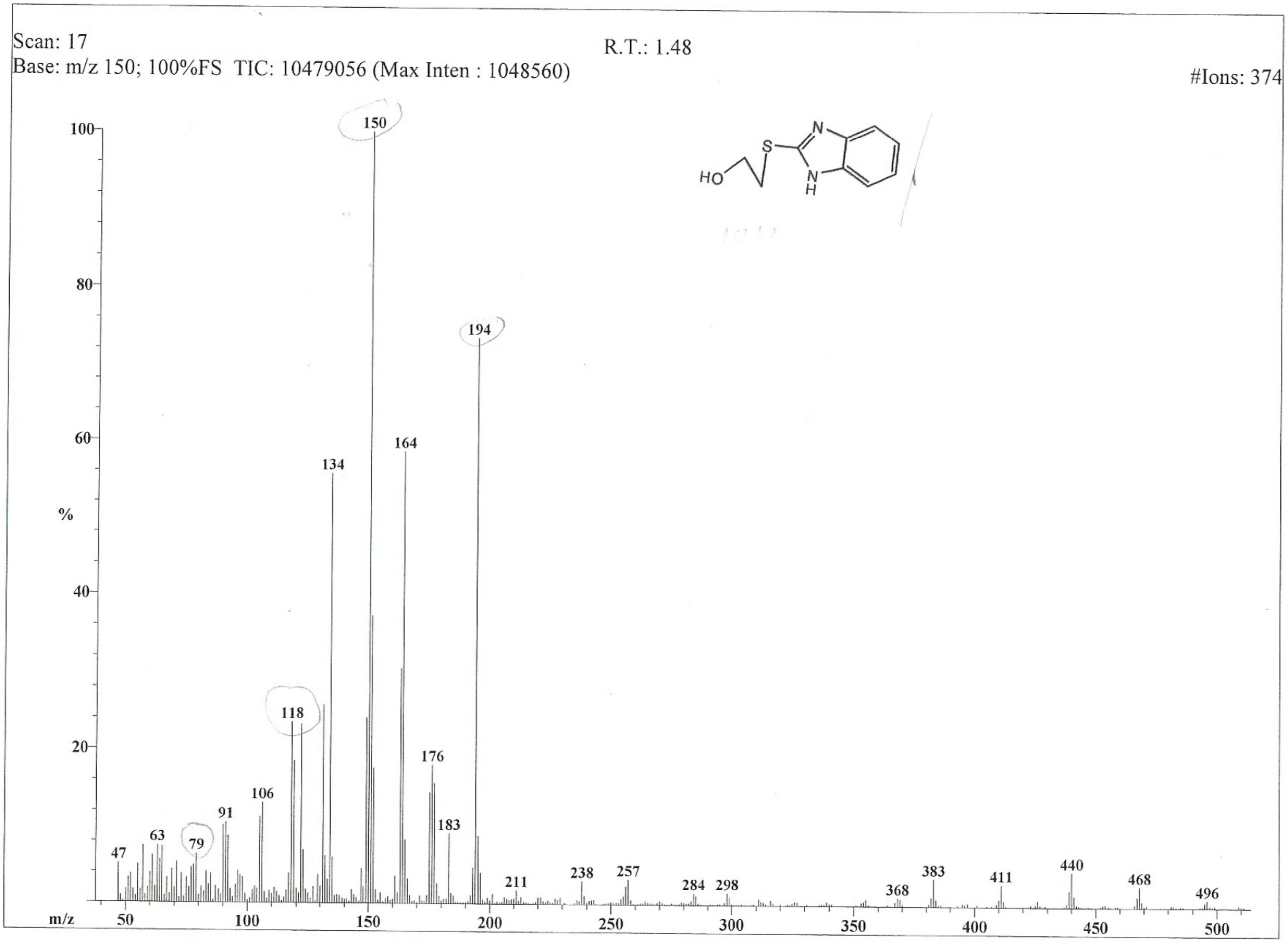

RAMADAN/DR.EL ASHRY/MRA.37/DMSO

c13 {BB}

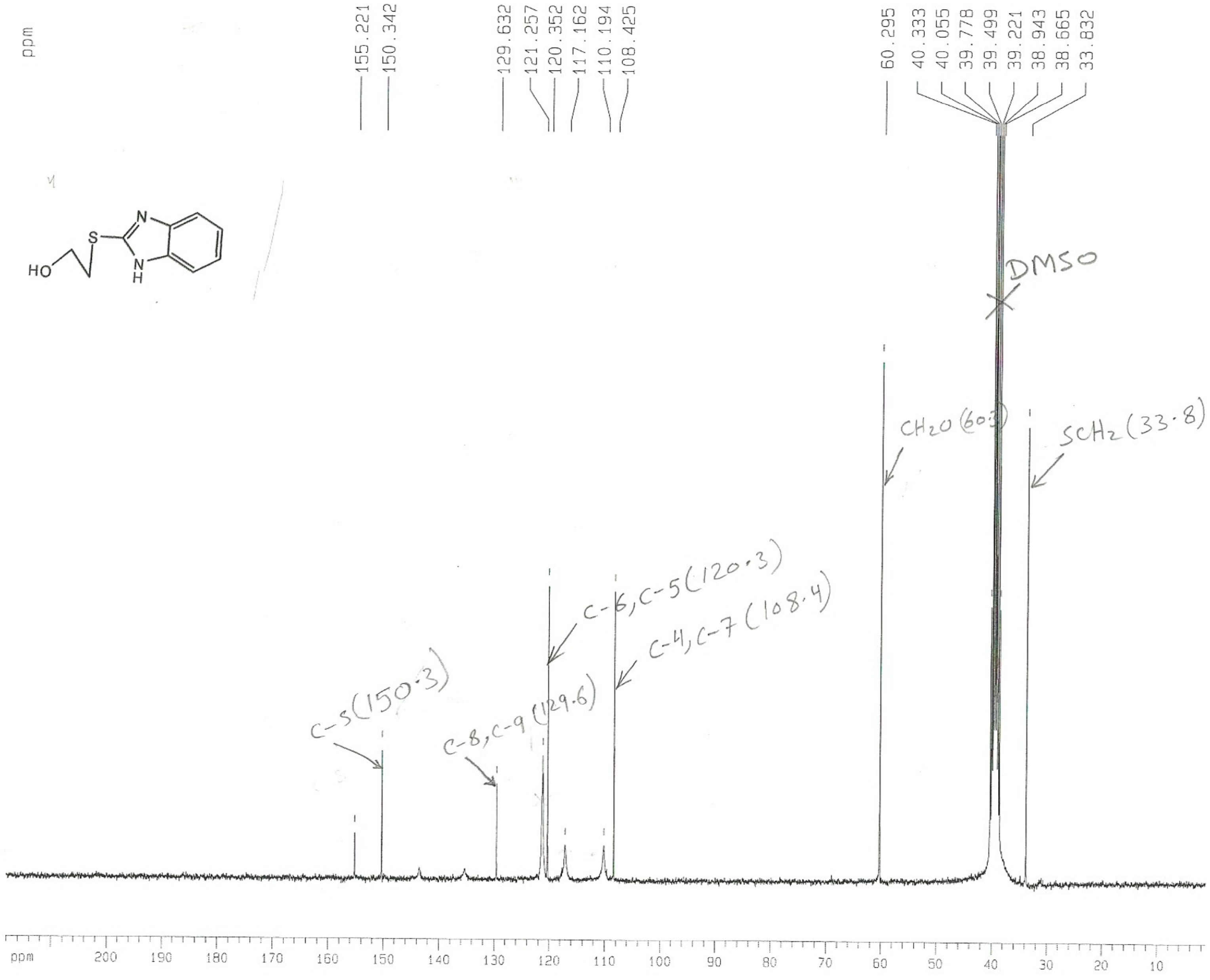

Current Data Parameters

NAME	oct09
EXPNO	10
PROCNO	1

F2 - Acquisition Parameters

Date_	20071010
Time	10.35
INSTRUM	spect
PROBHD	5 mm DUL 13C-1
PULPROG	zgpg30
TD	65536
SOLVENT	DMSO
NS	24581
DS	2
SWH	18518.518 Hz
FIDRES	0.282570 Hz
AQ	1.7695220 sec
RG	16384
DW	27.000 usec
DE	10.00 usec
TE	0.0 K
D1	1.50000000 sec
d11	0.03000000 sec
DELTA	1.39999998 sec
MCREST	0.00000000 sec
MCWRK	0.01500000 sec

======== CHANNEL f1 ========

NUC1	13C
P1	14.00 usec
PL1	-6.00 dB
SFO1	75.4764278 MHz

======== CHANNEL f2 ========

CPDPRG2	waltz16
NUC2	1H
PCPD2	100.00 usec
PL2	-3.00 dB
PL12	20.00 dB
PL13	20.00 dB
SFO2	300.1312005 MHz

F2 - Processing parameters

SI	32768
SF	75.4677864 MHz
WDW	EM
SSB	0
LB	1.50 Hz
GB	0
PC	1.40

1D NMR plot parameters

CX	20.00 cm
CY	30.00 cm
F1P	218.130 ppm
F1	16461.81 Hz
F2P	1.222 ppm
F2	92.26 Hz
PPMCM	10.84539 ppm/cm
HZCM	818.47754 Hz/cm

g Time: 11:34:17 AM
cing Date: Friday, April 03, 2009

*Installation
*QStar XL Ser. no. Y3200502

	Formula	Calculated m/z (amu)	mDa Error	PPM Error	DBE
1	C3 H9 N5 O5	195.0598	0.1799	0.9226	2.0
2	C4 H13 N5 S2	195.0606	-0.6905	-3.5403	1.0
3	C9 H11 N2 O S	195.0586	1.3386	6.8629	5.5
4	C6 H15 N2 O S2	195.0620	-2.0332	-10.4238	0.5
5	C7 H9 N5 S	195.0573	2.6813	13.7463	6.0
6	C8 H9 N3 O3	195.0638	-3.8427	-19.7001	6.0

Found = 195.0586
Calc. = 195.0592
$C_9H_{10}N_2OS$

found = 195.0586
Calc. = 195.0592

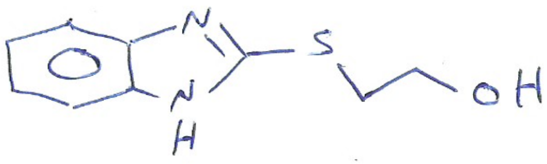

$\frac{195.060 - 195.0592}{195.0592} \times 10^6$

~~195.05138~~ + 1.0078
~~195.0592~~

194.0514
1.0078
195.0592

~~194.2535~~
~~1.0078~~
~~195.2612~~

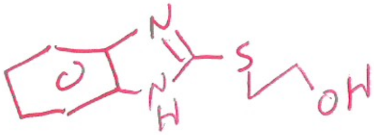

Mass	Relative Intensity	Theoretical Mass	Delta [ppm]	Delta [mmu]	Composition
192.9883	2.4190	181.9851	10.4	1.9	$C_7 H_2 O_6$
		192.9885	-1.4	-0.3	$C_7 H_1 O_5 N_2$
		192.9860	11.6	2.2	$C_{11} H_1 N_2 S_1$
		192.9859	12.5	2.4	$C_4 H_3 O_8 N_1$
193.0455	0.6639	193.0461	-2.9	-0.6	$C_5 H_9 O_6 N_2$
		193.0436	10.0	1.9	$C_9 H_9 O_1 N_2 S_1$
		193.0501	-23.8	-4.6	$C_{10} H_9 O_4$
194.0492	13.2899	194.0487	2.4	0.5	$C_6 H_{12} O_4 N_1 S_1$
		194.0480	5.9	1.1	$C_{12} H_6 O_1 N_2$
		194.0514	-11.5	-2.2	$C_9 H_{10} O_1 N_2 S_1$ ←
195.0501	1.2221	195.0505	-2.1	-0.4	$C_6 H_{11} O_7$
		195.0480	10.7	2.1	$C_{10} H_{11} O_2 S_1$
		195.0532	-15.9	-3.1	$C_9 H_9 O_4 N_1$
196.0450	0.5982	196.0457	-3.6	-0.7	$C_5 H_{10} O_7 N_1$
		196.0432	9.1	1.8	$C_9 H_{10} O_2 N_1 S_1$
		196.0484	-17.3	-3.4	$C_8 H_8 O_4 N_2$
204.9883	1.9593	204.9885	-1.3	-0.3	$C_8 H_1 O_5 N_2$
		204.9860	10.9	2.2	$C_{12} H_1 N_2 S_1$
		204.9859	11.7	2.4	$C_5 H_3 O_8 N_1$
211.9870	1.0355	211.9865	2.2	0.5	$C_4 H_6 O_7 N_1 S_1$
		211.9858	5.5	1.2	$C_{10} O_4 N_2$
		211.9892	-10.4	-2.2	$C_7 H_4 O_4 N_2 S_1$
216.9873	0.8001	216.9860	5.7	1.2	$C_{13} H_1 N_2 S_1$
		216.9885	-5.8	-1.3	$C_9 H_1 O_5 N_2$
		216.9859	6.5	1.4	$C_6 H_3 O_8 N_1$
218.9851	8.2335	218.9863	-5.4	-1.2	$C_2 H_5 O_{11} N_1$
		218.9838	6.0	1.3	$C_6 H_5 O_6 N_1 S_1$
		218.9864	-6.2	-1.4	$C_9 H_3 O_3 N_2 S_1$
230.9851	8.4968	230.9863	-5.1	-1.2	$C_3 H_5 O_{11} N_1$
		230.9838	5.7	1.3	$C_7 H_5 O_6 N_1 S_1$
		230.9864	-5.9	-1.4	$C_{10} H_3 O_3 N_2 S_1$
231.9888	0.6371	231.9889	-0.3	-0.1	$C_4 H_8 O_9 S_1$
		231.9882	2.7	0.6	$C_{10} H_2 O_6 N_1$
		231.9909	-8.9	-2.1	$C_{13} O_3 N_2$
235.9877	0.5520	235.9865	5.0	1.2	$C_6 H_6 O_7 N_1 S_1$
		235.9892	-6.4	-1.5	$C_9 H_4 O_4 N_2 S_1$
		235.9858	7.9	1.9	$C_{12} O_4 N_2$
242.9851	5.2079	242.9863	-4.9	-1.2	$C_4 H_5 O_{11} N_1$
		242.9838	5.4	1.3	$C_8 H_5 O_6 N_1 S_1$
		242.9864	-5.6	-1.4	$C_{11} H_3 O_3 N_2 S_1$
254.9851	1.6537	254.9863	-4.6	-1.2	$C_5 H_5 O_{11} N_1$
		254.9838	5.2	1.3	$C_9 H_5 O_6 N_1 S_1$
		254.9864	-5.3	-1.4	$C_{12} H_3 O_3 N_2 S_1$
266.9823	1.3256	266.9811	4.6	1.2	$C_7 H_7 O_9 S_1$

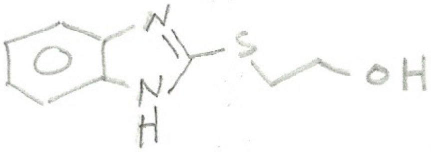

Found = 194.0492

Calc. = 194.0514

$C_9 H_{10} O_1 N_2 S_1$

AAMER/DR.EL ASHRY/MRA.139A

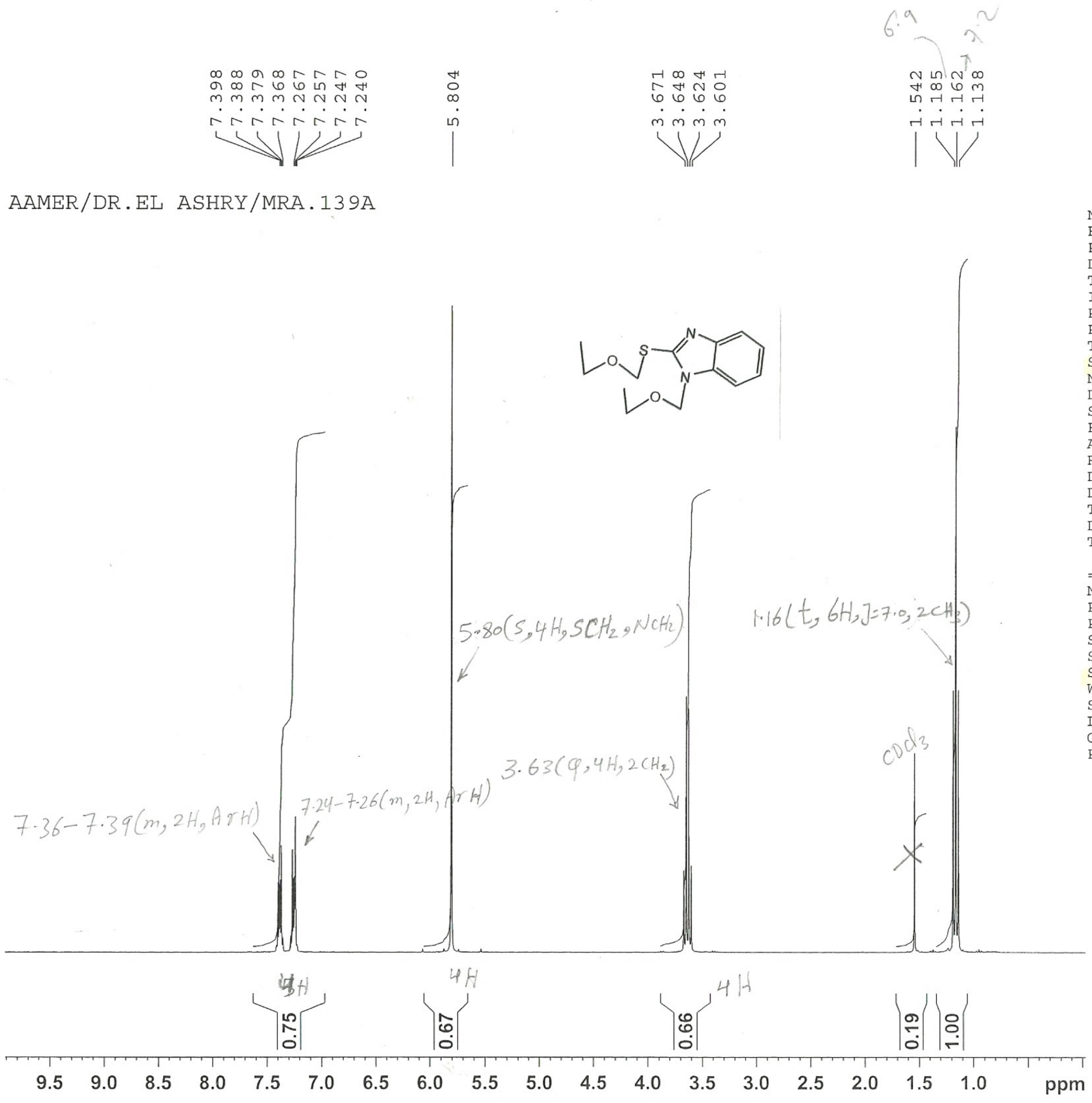

NAME	april21
EXPNO	5
PROCNO	1
Date_	20090421
Time	11.46
INSTRUM	spect
PROBHD	5 mm DUL 13C-1
PULPROG	zg30
TD	32768
SOLVENT	CDCl3
NS	128
DS	0
SWH	6172.839 Hz
FIDRES	0.188380 Hz
AQ	2.6542580 sec
RG	812.7
DW	81.000 usec
DE	6.50 usec
TE	300.0 K
D1	1.50000000 sec
TD0	1

======== CHANNEL f1 ========

NUC1	1H
P1	10.00 usec
PL1	-3.00 dB
SFO1	300.1324010 MHz
SI	16384
SF	300.1300119 MHz
WDW	EM
SSB	0
LB	0.30 Hz
GB	0
PC	1.00

AVANCE AV 300
LAB. NO. 115

File Name	:	d:\mswin\data\mra-280.mss
Creation Date/Time	:	20/04/09 at 14:37:08
File Type	:	Lo-Res Mass Data (Centroid)
File Source	:	Acquired on MASPEC system [msw/A091]
File Title	:	EI
Operator	:	Barkat Ali
Instrument	:	MAT312

SCAN GRAPH. Flagging=M/z. Filter=[Int:1%.].
Scan 3-1:30. Entries=66. 100% Int.=63659.

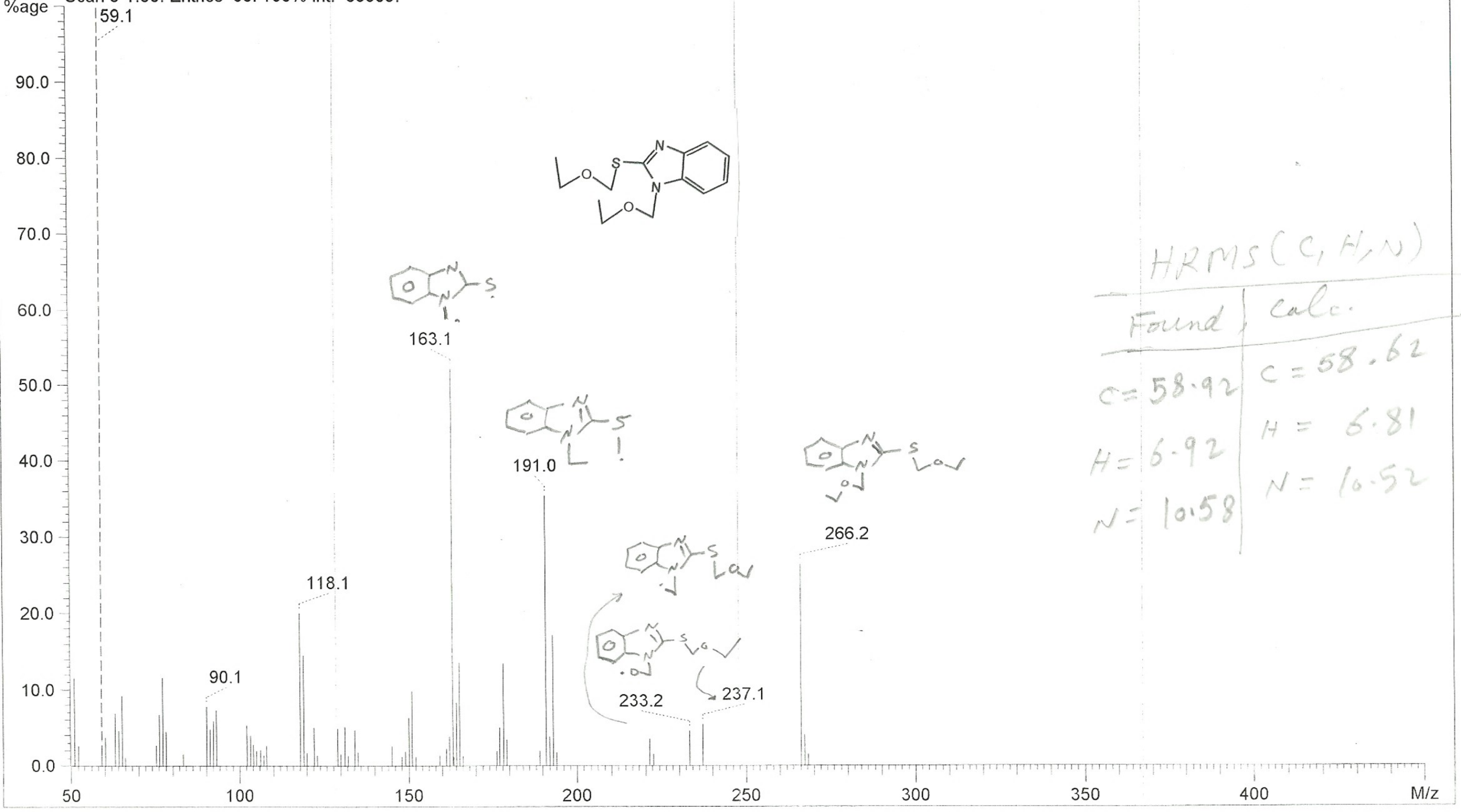

HRMS (C, H, N)

Found	Calc.
C = 58.92	C = 58.62
H = 6.92	H = 6.81
N = 10.58	N = 10.52

AVANCE AV 300
LAB. No. 115

M.AMER/DR.EL ASHRY/MRA-139

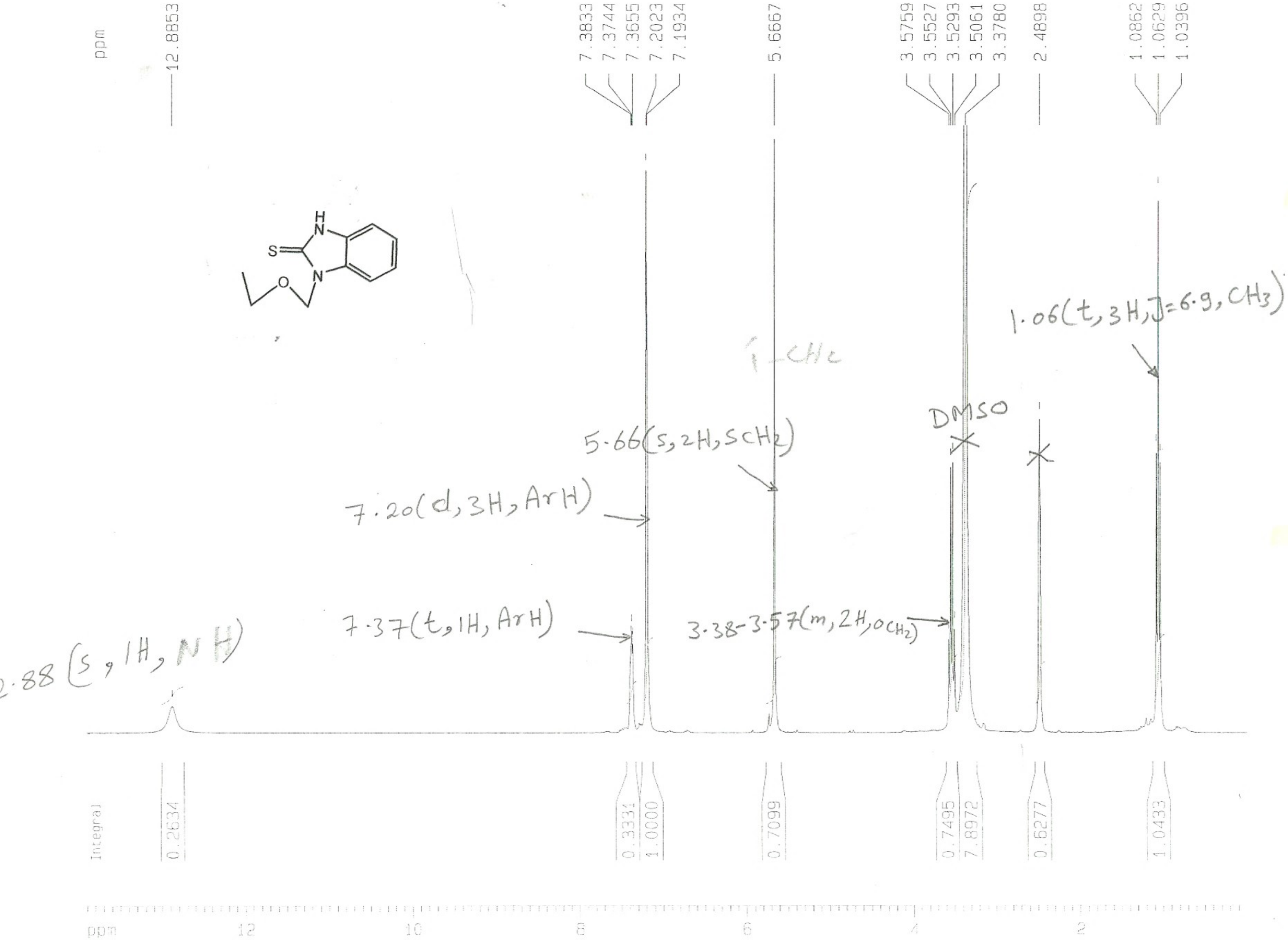

Current Data Parameters
NAME april01
EXPNO 2
PROCNO 1

F2 - Acquisition Parameters
Date_ 20080401
Time 12.16
INSTRUM spect
PROBHD 5 mm DUL 13C-1
PULPROG zg30
TD 32768
SOLVENT DMSO
NS 64
DS 0
SWH 5995.204 Hz
FIDRES 0.182959 Hz
AQ 2.7329011 sec
RG 456.1
DW 83.400 usec
DE 10.00 usec
TE 0.0 K
D1 1.00000000 sec
MCREST 0.00000000 sec
MCWRK 0.01500000 sec

======== CHANNEL f1 ========
NUC1 1H
P1 10.20 usec
PL1 -3.00 dB
SFO1 300.1324010 MHz

F2 - Processing parameters
SI 16384
SF 300.1300042 MHz
WDW EM
SSB 0
LB 0.30 Hz
GB 0
PC 1.00

1D NMR plot parameters
CX 20.00 cm
CY 0.00 cm
F1P 13.911 ppm
F1 4175.02 Hz
F2P 0.000 ppm
F2 0.00 Hz
PPMCM 0.69553 ppm/cm
HZCM 208.75054 Hz/cm

M/Amer/Dr.EL Ashry
Sample: MRA=139

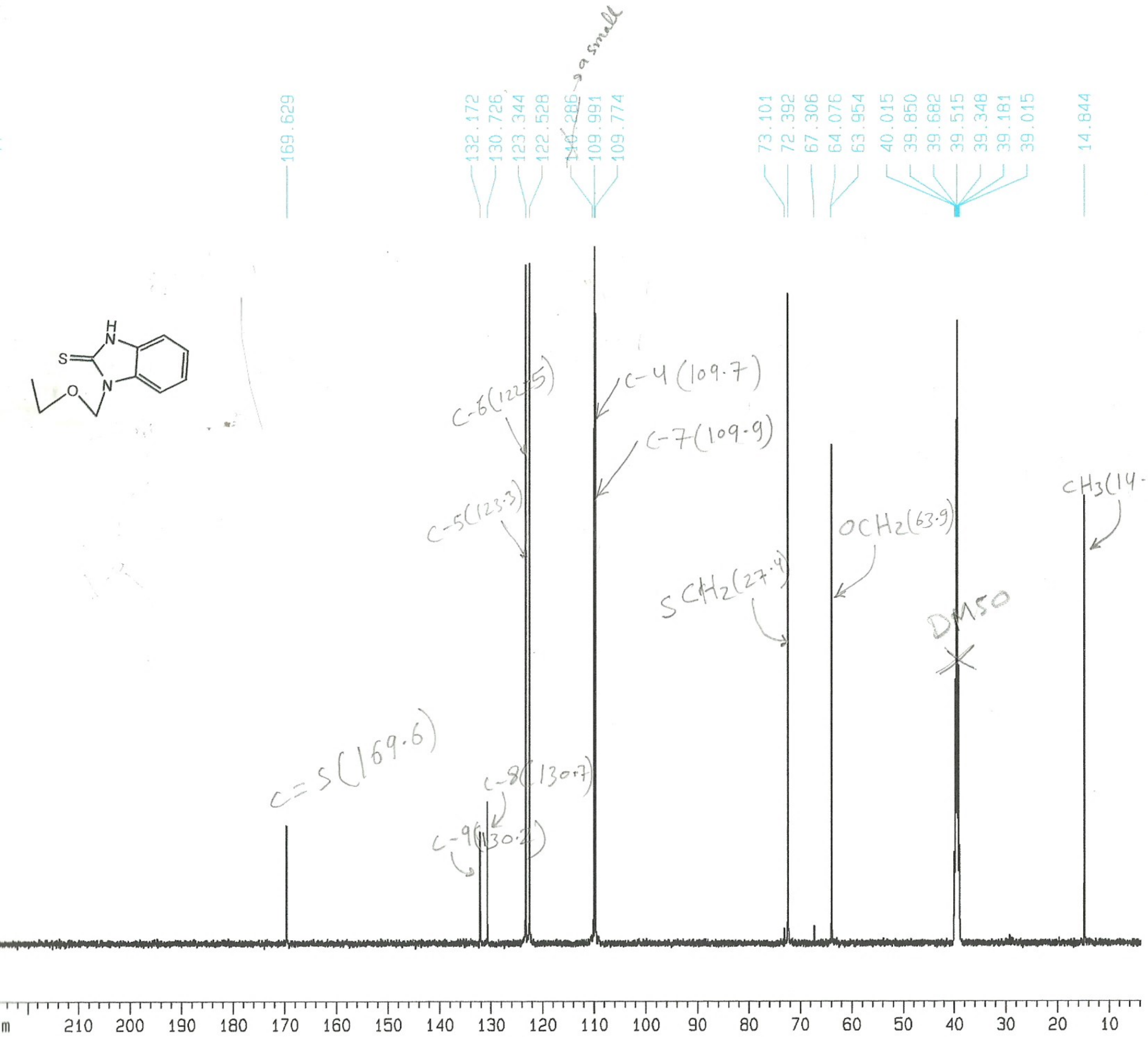

Current Data Parameters
NAME April01
EXPNO 5
PROCNO 1

F2 - Acquisition Parameters
Date_ 20080401
Time 14.05
INSTRUM spect
PROBHD 5 mm SEI 1H-13
PULPROG zgpg
TD 32768
SOLVENT DMSO
NS 6144
DS 2
SWH 30303.031 Hz
FIDRES 0.924775 Hz
AQ 0.5407385 sec
RG 32768
DW 16.500 usec
DE 50.00 usec
TE 300.0 K
D1 1.50000000 sec
d11 0.03000000 sec
d12 0.00002000 sec

======== CHANNEL f1 ========
NUC1 13C
P1 14.50 usec
PL1 0.00 dB
SFO1 125.7719204 MHz

======== CHANNEL f2 ========
CPDPRG2 waltz16
NUC2 1H
PCPD2 100.00 usec
PL2 5.00 dB
PL12 28.61 dB
PL13 23.00 dB
SFO2 500.1325006 MHz

F2 - Processing parameters
SI 32768
SF 125.7578496 MHz
WDW EM
SSB 0
LB 1.50 Hz
GB 0
PC 1.40

1D NMR plot parameters
CX 20.00 cm
CY 12.00 cm
F1P 229.074 ppm
F1 28807.81 Hz
F2P 3.857 ppm
F2 485.07 Hz
PPMCM 11.26082 ppm/cm
HZCM 1416.13696 Hz/cm

AVANCE 50[illegible]
LAB. No. 109-[illegible]

1.Amer/Dr.EL Ashry
/Sample: MRA=139
Dept 135

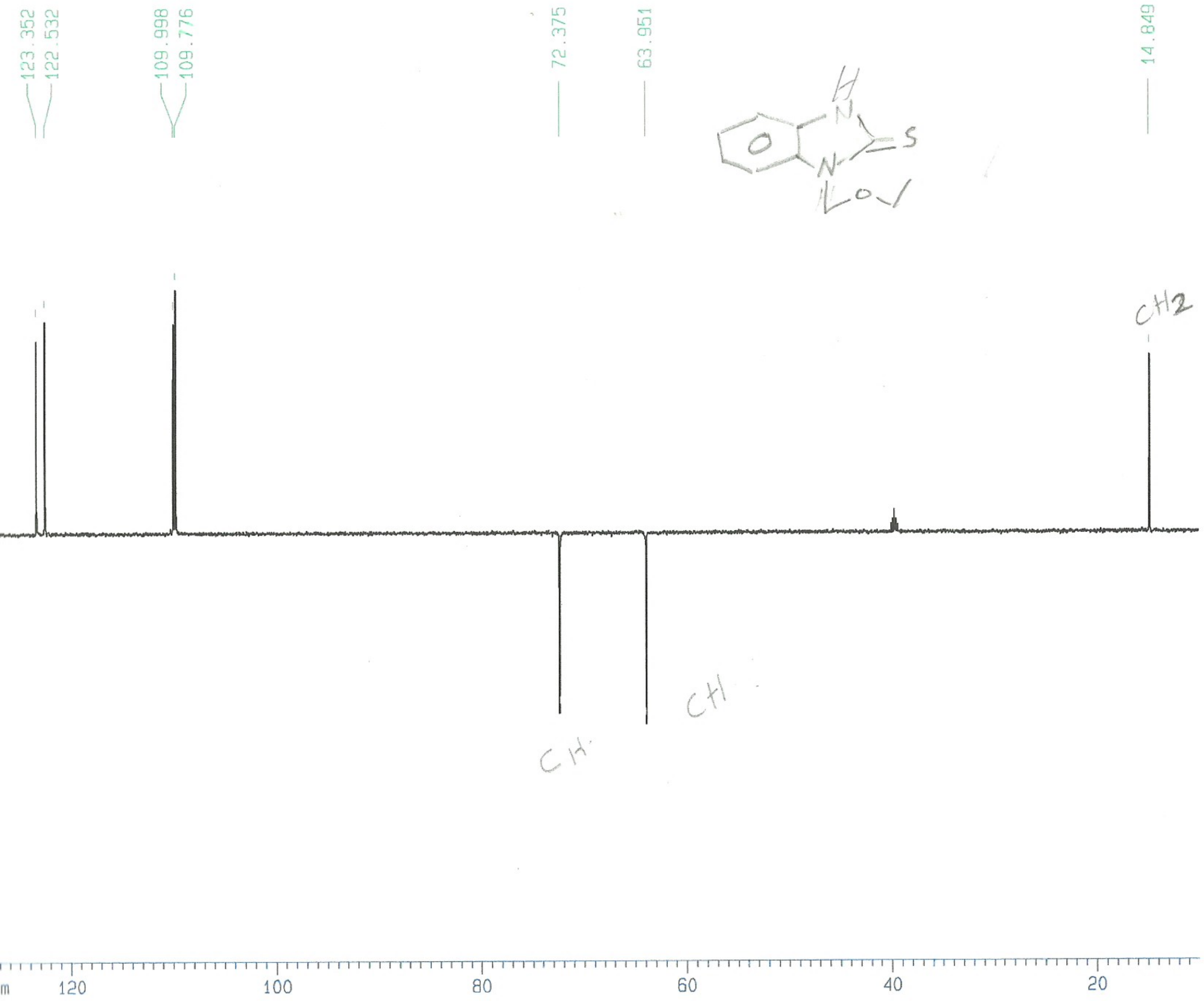

Current Data Parameters
NAME April01
EXPNO 6
PROCNO 1

F2 - Acquisition Parameters
Date_ 20080401
Time 19.54
INSTRUM spect
PROBHD 5 mm SEI 1H-13
PULPROG deptsp135
TD 32768
SOLVENT DMSO
NS 4096
DS 2
SWH 30030.029 Hz
FIDRES 0.916444 Hz
AQ 0.5456539 sec
RG 32768
DW 16.650 usec
DE 50.00 usec
TE 300.0 K
CNST2 145.0000000
D1 1.50000000 sec
d2 0.00344828 sec
d12 0.00002000 sec
DELTA 0.00001846 sec

======== CHANNEL f1 ========
NUC1 13C
P1 14.50 usec
P12 2000.00 usec
PL0 120.00 dB
PL1 0.00 dB
SFO1 125.7715724 MHz
SP2 1.99 dB
SPNAM2 Crp60comp.4
SPOFF2 0.00 Hz

======== CHANNEL f2 ========
CPDPRG2 waltz16
NUC2 1H
P3 6.60 usec
p4 13.20 usec
PCPD2 100.00 usec
PL2 5.00 dB
PL12 23.00 dB
SFO2 500.1325006 MHz

F2 - Processing parameters
SI 16384
SF 125.7578496 MHz
WDW EM
SSB 0
LB 1.50 Hz
GB 0
PC 1.40

1D NMR plot parameters
CX 20.00 cm
CY 4.00 cm
F1P 129.080 ppm
F1 16232.86 Hz
F2P 10.047 ppm
F2 1263.49 Hz
PPMCM 5.95167 ppm/cm
HZCM 748.46875 Hz/cm

AVANCE 500

File: MRA-139 **Date Run: 03-26-2008 (Time Run: 11:09:39)**
Sample: MOHAMED RAMADAN AMER
Instrument: JEOL MSRoute
Inlet: My Inlet **Ionization mode: EI-**

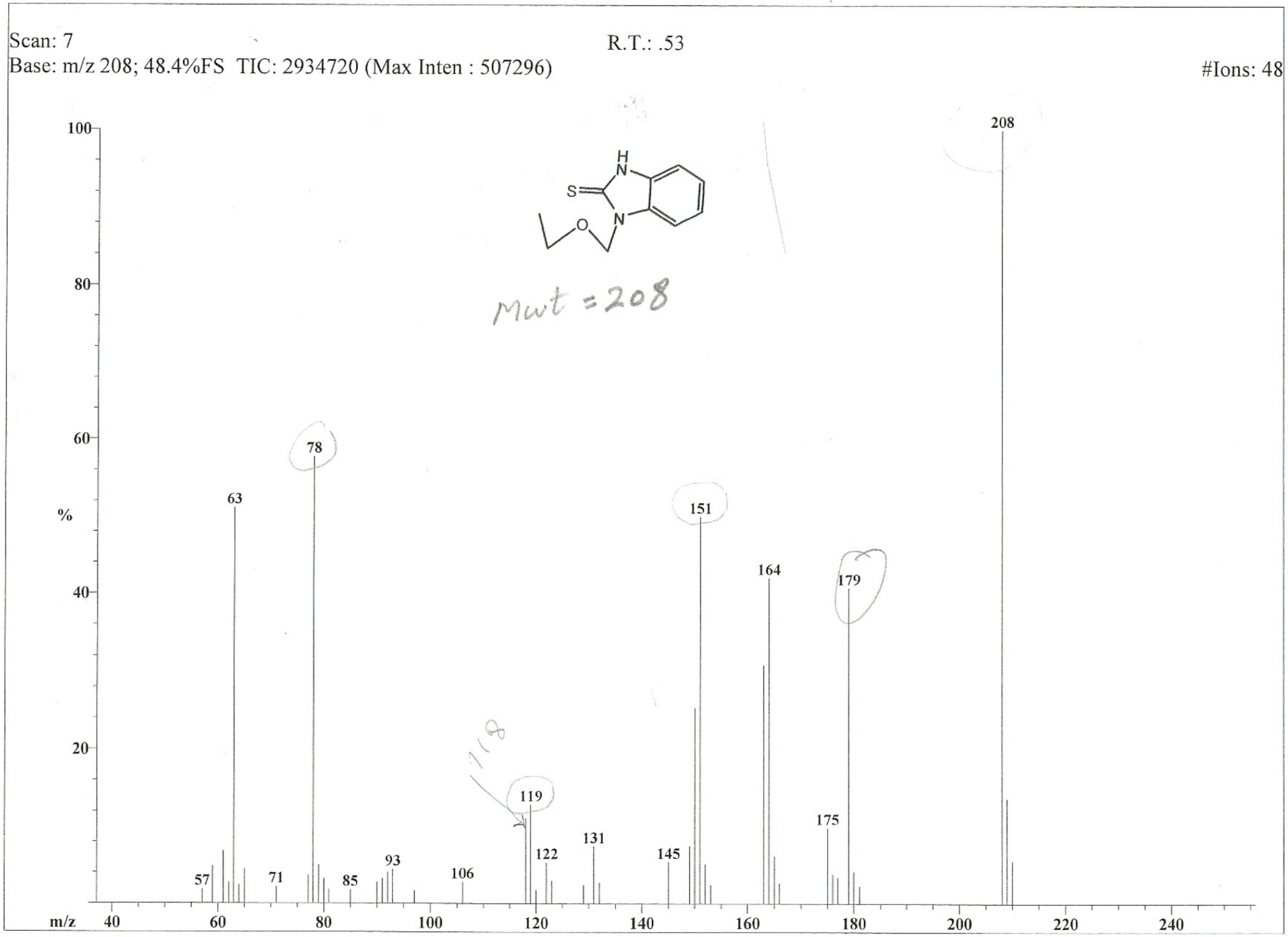

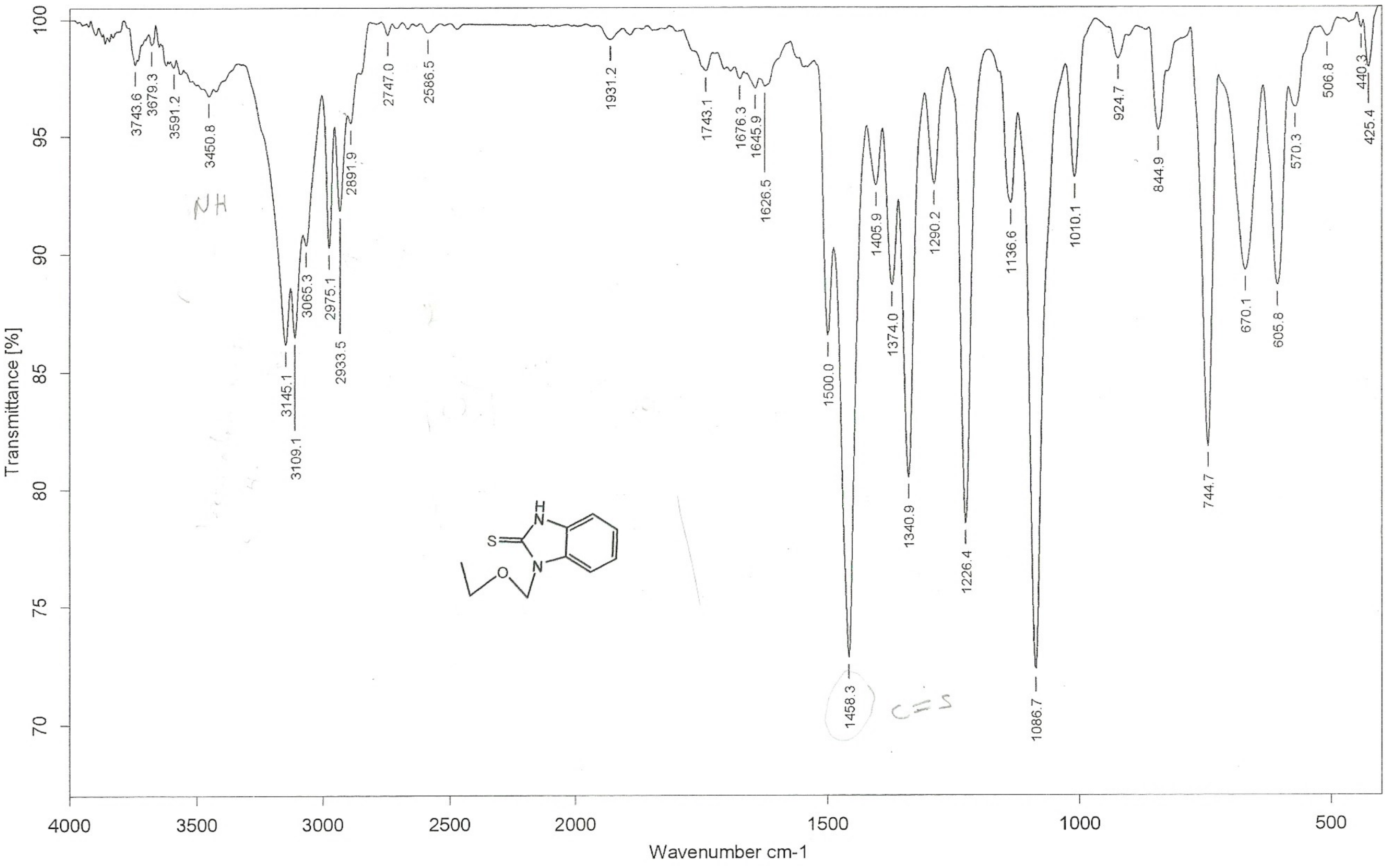

Sample : mra-139/amer/el ashrry

Measured : 24/01/2009 on VECTOR22

Resolution : 4 cm-1 (20 scans)

Spectrum : mra-139.0 (in D:\IRSTUDENT)

Technic : solid

Analyst : mohammed amer

	Formula	Calculated m/z (amu)	mDa Error	PPM Error	DBE
1	C10 H13 N2 O S	209.0743	1.9886	9.5113	5.5
2	C11 H15 N O S	209.0868	-10.5874	-50.6391	5.0
3	C11 H13 O2 S	209.0630	13.2219	63.2398	5.5
4	C7 H15 N O2 S2	209.0538	22.4260	107.2624	1.0
5	C10 H11 N O2 S	209.0505	25.7980	123.3902	6.0
6	C11 H13 S2	209.0453	30.9794	148.1725	5.5
7	C6 H13 N2 O2 S2	209.0412	35.0021	167.4129	1.5
8	C9 H9 N2 O2 S	209.0379	38.3741	183.5407	6.5
9	C12 H5 N2 O2	209.0345	41.7460	199.6686	11.5
10	C10 H11 N S2	209.0327	43.5555	208.3230	6.0

Found = 209.0743
calc. = 209.0748

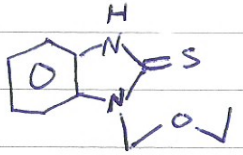

208.0670 + 1.0078

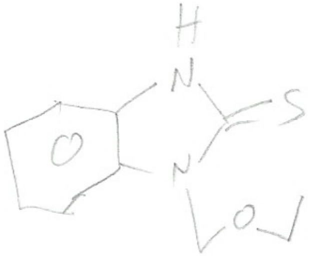

$C_{10}H_{13}N_2OS$
Found = 209.0743

208.0670
1.0078
209.0748

RAMADAN/DR.EL ASHRY/MRA.10/DMSO

AVANCE AV 300
LAB. No. 115

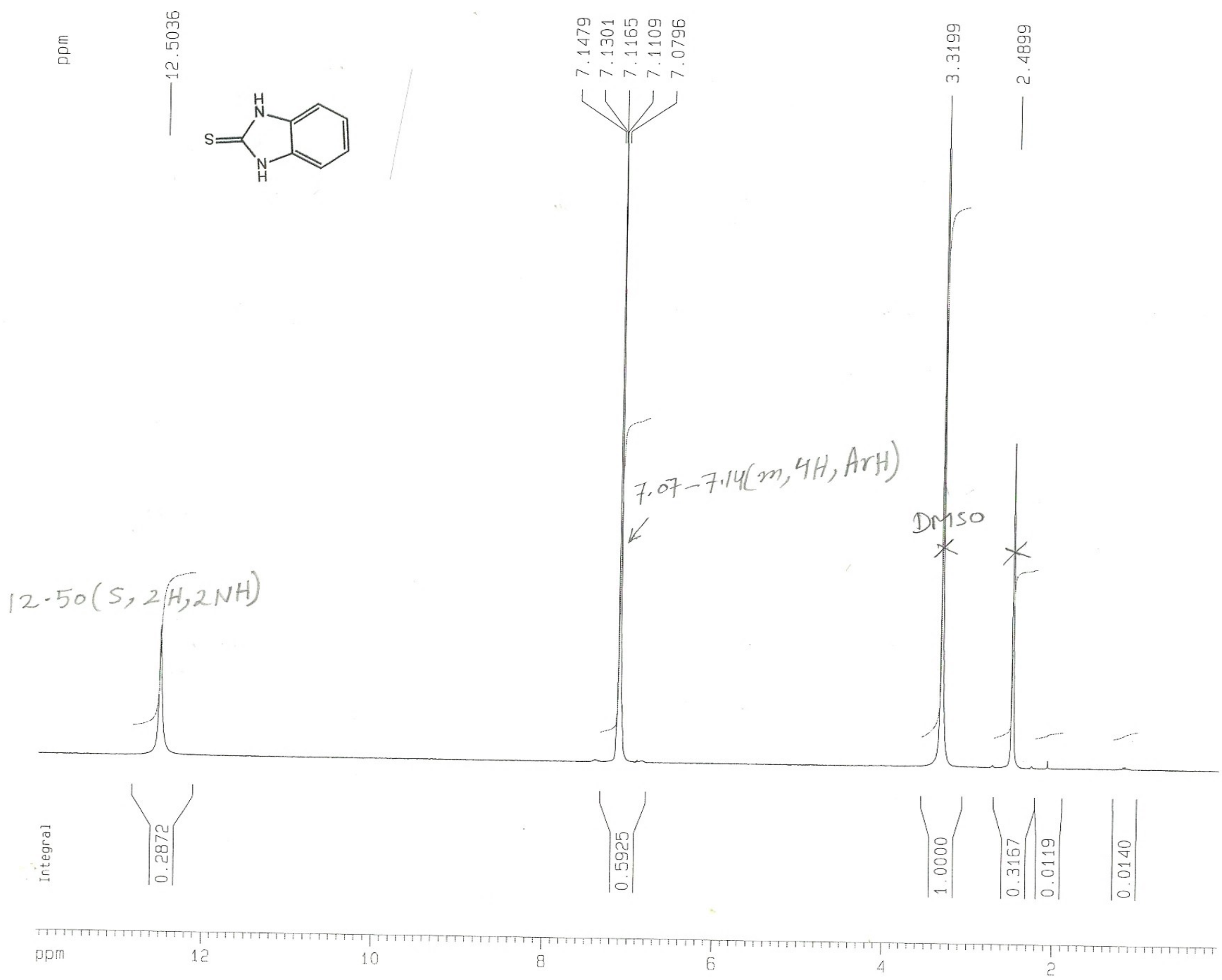

Current Data Parameters

NAME	oct09
EXPNO	6
PROCNO	1

F2 - Acquisition Parameters

Date_	20071009
Time	11.09
INSTRUM	spect
PROBHD	5 mm DUL 13C-1
PULPROG	zg30
TD	32768
SOLVENT	DMSO
NS	128
DS	0
SWH	5995.204 Hz
FIDRES	0.182959 Hz
AQ	2.7329011 sec
RG	1625.5
DW	83.400 usec
DE	10.00 usec
TE	0.0 K
D1	1.00000000 sec
MCREST	0.00000000 sec
MCWRK	0.01500000 sec

======== CHANNEL f1 ========

NUC1	1H
P1	10.20 usec
PL1	-3.00 dB
SFO1	300.1324010 MHz

F2 - Processing parameters

SI	16384
SF	300.1300042 MHz
WDW	EM
SSB	0
LB	0.30 Hz
GB	0
PC	1.00

1D NMR plot parameters

CX	20.00 cm
CY	30.00 cm
F1P	13.942 ppm
F1	4184.55 Hz
F2P	0.054 ppm
F2	16.34 Hz
PPMCM	0.69440 ppm/cm
HZCM	208.41048 Hz/cm

AVANCE 400
LAB. No. 117

M.RAMADAN/DR.EL.ASHRY/MRA-14
C13 {BB}

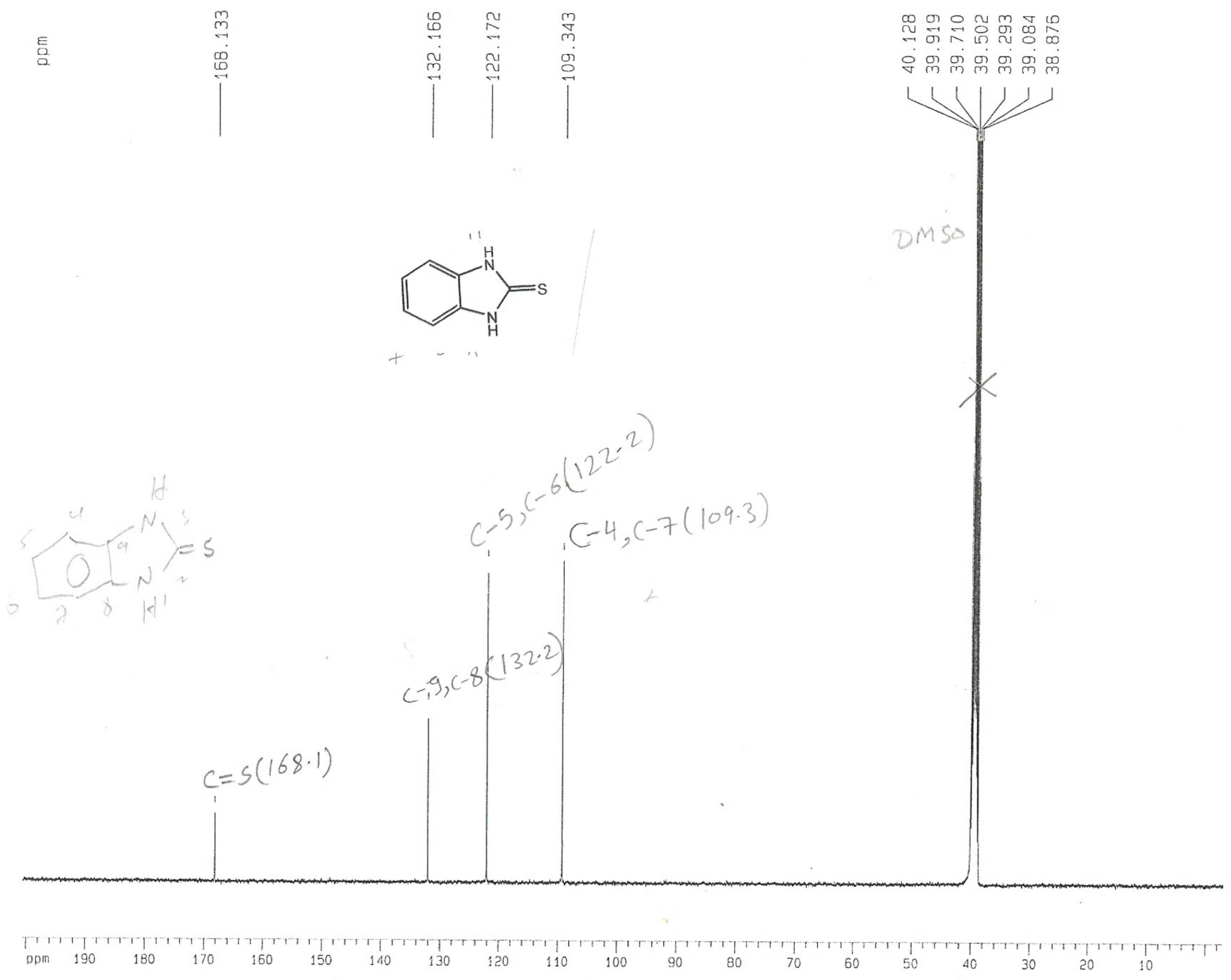

Current Data Parameters
NAME july28
EXPNO 1
PROCNO 1

F2 - Acquisition Parameters
Date_ 20070728
Time 14.00
INSTRUM spect
PROBHD 5 mm DUL 13C-1
PULPROG zgpg30
TD 32768
SOLVENT DMSO
NS 25000
DS 2
SWH 26178.010 Hz
FIDRES 0.798889 Hz
AQ 0.6259188 sec
RG 16384
DW 19.100 usec
DE 20.00 usec
TE 300.0 K
D1 1.50000000 sec
d11 0.03000000 sec
d12 0.00002000 sec

======== CHANNEL f1 ========
NUC1 13C
P1 5.50 usec
PL1 0.00 dB
SFO1 100.6243395 MHz

======== CHANNEL f2 ========
CPDPRG2 waltz16
NUC2 1H
PCPD2 100.00 usec
PL2 -3.00 dB
PL12 16.50 dB
PL13 16.50 dB
SFO2 400.1320007 MHz

F2 - Processing parameters
SI 32768
SF 100.6128269 MHz
WDW EM
SSB 0
LB 1.50 Hz
GB 0
PC 1.40

1D NMR plot parameters
CX 20.00 cm
CY 50.00 cm
F1P 200.653 ppm
F1 20188.24 Hz
F2P -2.922 ppm
F2 -293.98 Hz
PPMCM 10.17873 ppm/cm
HZCM 1024.11084 Hz/cm

File: MRA10 **Date Run:** 10-04-2007 (Time Run: 09:20:54)
Sample: MOHAMED RAMADAN AMER
Instrument: JEOL MSRoute
Inlet: My Inlet **Ionization mode:** EI-

Scan: 39 R.T.: 3.5
Base: m/z 150; 100%FS TIC: 4321072 (Max Inten : 1048560) #Ions: 109

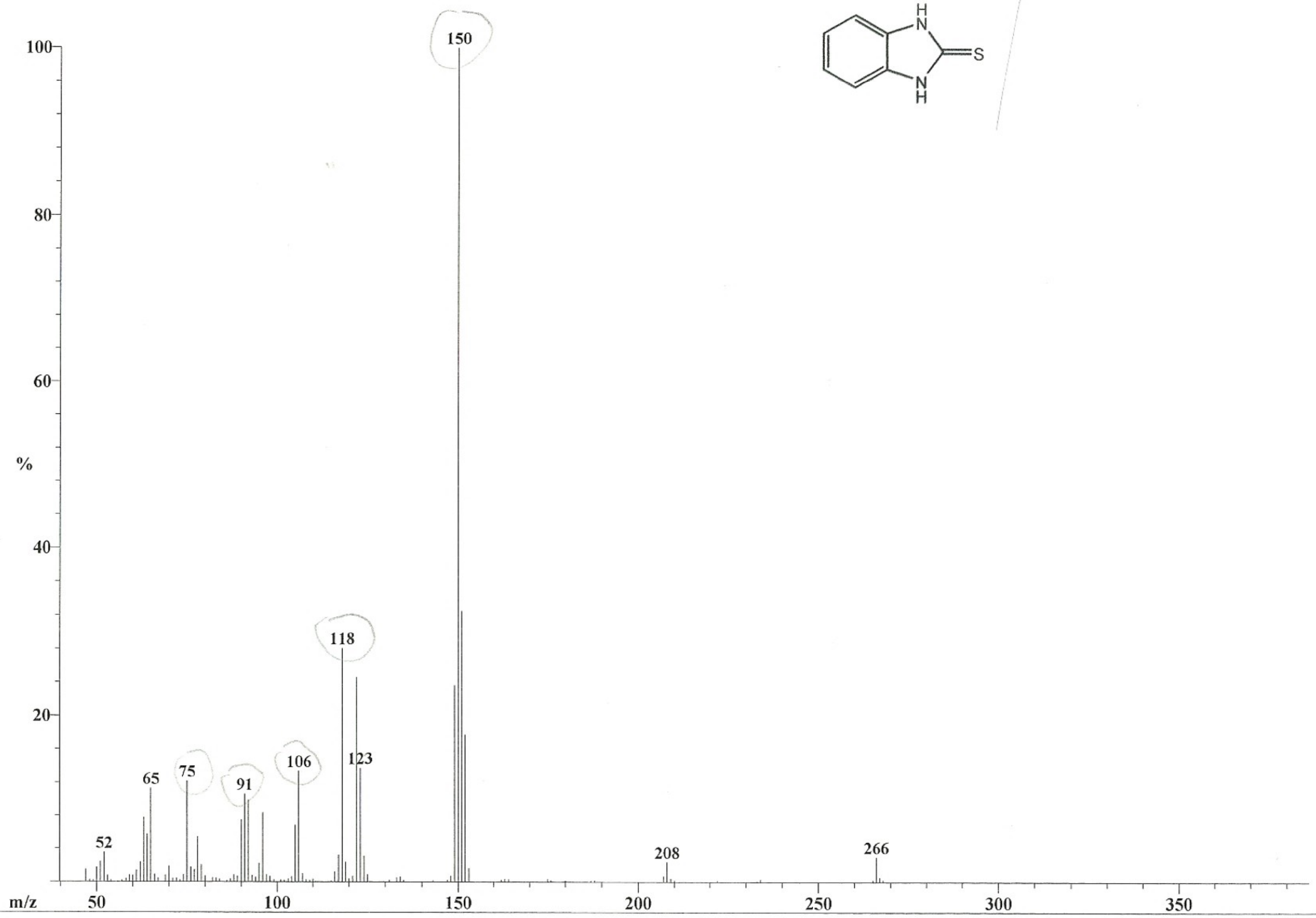

Mass	Relative Intensity	Theoretical Mass	Delta [ppm]	Delta [mmu]	Composition
124.0043	4.6868	124.0035	6.6	0.8	$C_5 H_2 O_3 N_1$
		124.0061	-15.0	-1.9	$C_8 N_2$
		124.0008	28.2	3.5	$C_2 H_4 O_6$
124.0146	4.6718	124.0160	-11.5	-1.4	$C_6 H_4 O_3$
		124.0120	20.9	2.6	$C_1 H_4 O_5 N_2$
		124.0187	-33.1	-4.1	$C_9 H_2 N_1$
125.0072	2.1854	125.0061	8.9	1.1	$C_6 H_5 O_1 S_1$
		125.0113	-32.6	-4.1	$C_5 H_3 O_3 N_1$
		125.0027	35.8	4.5	$C_9 H_1 O_1$
130.9915	4.0281	130.9915	-0.4	-0.1	$C_3 H_3 O_2 N_2 S_1$
		130.9955	-31.1	-4.1	$C_8 H_3 S_1$
		130.9855	45.8	6.0	$C_3 H_1 O_5 N_1$
132.9985	1.0274	132.9986	-0.6	-0.1	$C_7 H_3 N_1 S_1$
		133.0011	-19.4	-2.6	$C_3 H_3 O_5 N_1$
		132.9959	19.6	2.6	$C_4 H_5 O_3 S_1$
134.0469	1.7001	134.0480	-8.4	-1.1	$C_7 H_6 O_1 N_2$
		134.0453	11.6	1.6	$C_4 H_8 O_4 N_1$
		134.0514	-33.5	-4.5	$C_4 H_{10} O_1 N_2 S_1$
143.0749	1.4168	143.0735	9.5	1.4	$C_{10} H_9 N_1$
		143.0769	-14.1	-2.0	$C_7 H_{13} N_1 S_1$
		143.0708	28.2	4.0	$C_7 H_{11} O_3$
147.0008	0.8588	147.0017	-6.2	-0.9	$C_7 H_3 N_2 S_1$
		146.9990	12.1	1.8	$C_4 H_5 O_3 N_1 S_1$
		147.0042	-23.2	-3.4	$C_3 H_3 O_5 N_2$
148.0105	1.0313	148.0095	6.8	1.0	$C_7 H_4 N_2 S_1$
		148.0120	-10.1	-1.5	$C_3 H_4 O_5 N_2$
		148.0068	24.9	3.7	$C_4 H_6 O_3 N_1 S_1$
149.0179	79.1020	149.0173	3.6	0.5	$C_7 H_5 N_2 S_1$
		149.0198	-13.2	-2.0	$C_3 H_5 O_5 N_2$
		149.0147	21.6	3.2	$C_4 H_7 O_3 N_1 S_1$
150.0236	100.0000	150.0225	7.3	1.1	$C_4 H_8 O_3 N_1 S_1$
		150.0252	-10.6	-1.6	$C_7 H_6 N_2 S_1$
		150.0218	11.9	1.8	$C_{10} H_2 N_2$
151.0289	92.5912	151.0296	-5.0	-0.8	$C_{10} H_3 N_2$
		151.0303	-9.6	-1.5	$C_4 H_9 O_3 N_1 S_1$
		151.0269	12.7	1.9	$C_7 H_5 O_3 N_1$
152.0221	59.4125	152.0222	-0.3	-0.0	$C_6 H_4 O_3 N_2$
		152.0195	17.3	2.6	$C_3 H_6 O_6 N_1$
		152.0256	-22.5	-3.4	$C_3 H_8 O_3 N_2 S_1$
153.0244	4.1370	153.0248	-3.1	-0.5	$C_7 H_7 O_1 N_1 S_1$
		153.0215	18.9	2.9	$C_{10} H_3 O_1 N_1$
		153.0273	-19.5	-3.0	$C_3 H_7 O_6 N_1$
163.0336	0.8190	163.0330	3.5	0.6	$C_8 H_7 N_2 S_1$
		163.0355	-11.8	-1.9	$C_4 H_7 O_5 N_2$

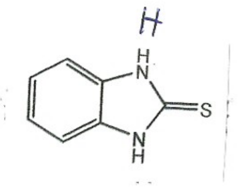

Found = 150.0236

Calc. = 150.0252

$C_7 H_6 N_6 S_1$

68K

AVANCE 500
LAB No. 109-B

AMER/DR.EL ASHRY/MRA-85C

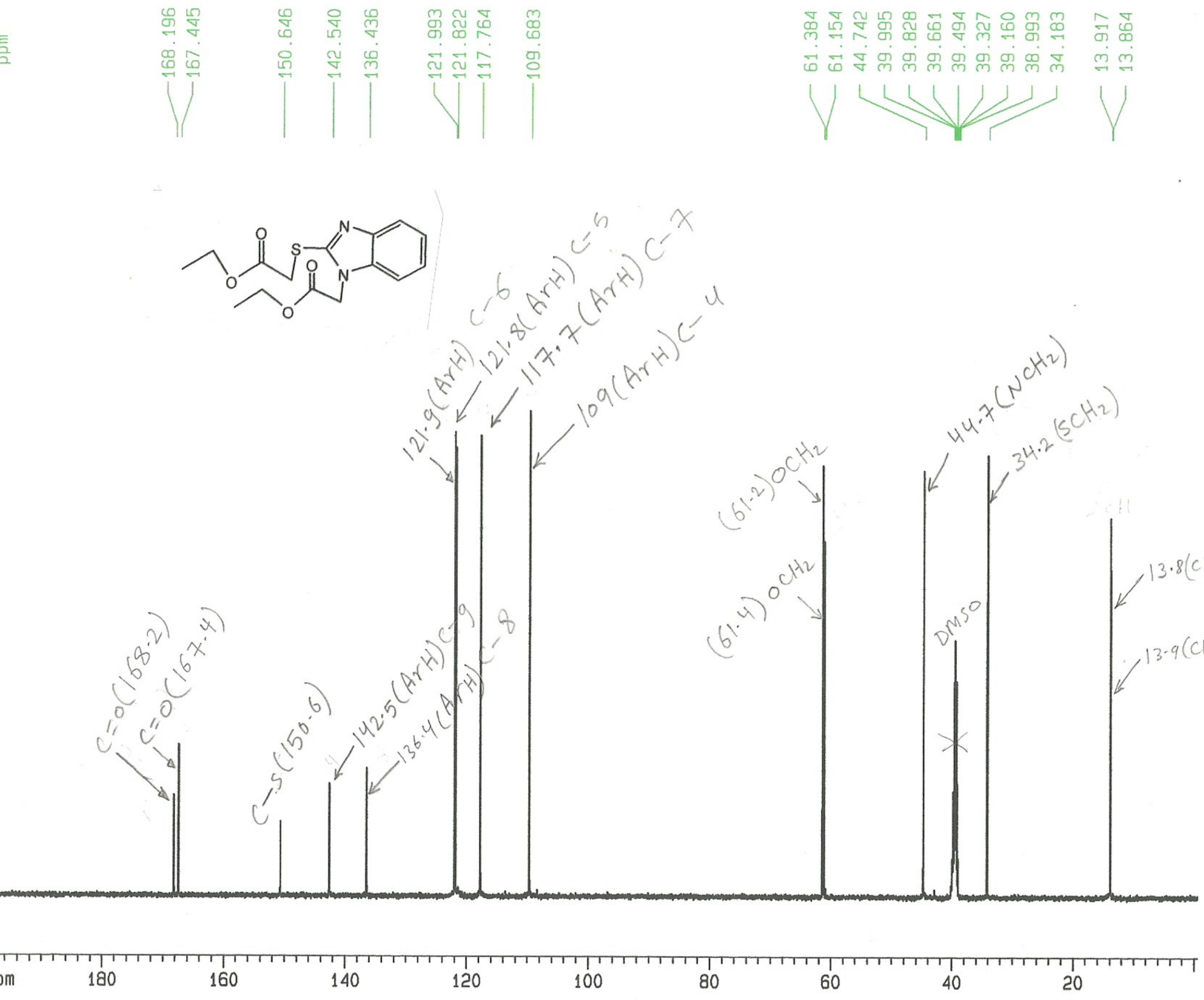

Current Data Parameters
NAME sep09
EXPNO 6
PROCNO 1

F2 - Acquisition Parameters
Date_ 20080909
Time 10.57
INSTRUM spect
PROBHD 5 mm SEI 1H-13
PULPROG zgpg
TD 32768
SOLVENT DMSO
NS 1913
DS 2
SWH 30303.031 Hz
FIDRES 0.924775 Hz
AQ 0.5407385 sec
RG 32768
DW 16.500 usec
DE 50.00 usec
TE 300.0 K
D1 1.50000000 sec
d11 0.03000000 sec
d12 0.00002000 sec

======== CHANNEL f1 ========
NUC1 13C
P1 14.50 usec
PL1 0.00 dB
SFO1 125.7719204 MHz

======== CHANNEL f2 ========
CPDPRG2 waltz16
NUC2 1H
PCPD2 100.00 usec
PL2 5.00 dB
PL12 28.61 dB
PL13 23.00 dB
SFO2 500.1325006 MHz

F2 - Processing parameters
SI 32768
SF 125.7578568 MHz
WDW EM
SSB 0
LB 1.00 Hz
GB 0
PC 1.40

1D NMR plot parameters
CX 20.00 cm
CY 8.00 cm
F1P 199.353 ppm
F1 25070.26 Hz
F2P -0.595 ppm
F2 -74.81 Hz
PPMCM 9.99741 ppm/cm
HZCM 1257.25330 Hz/cm

AMER/DR.EL ASHRY/MRA-85C

Dept 135

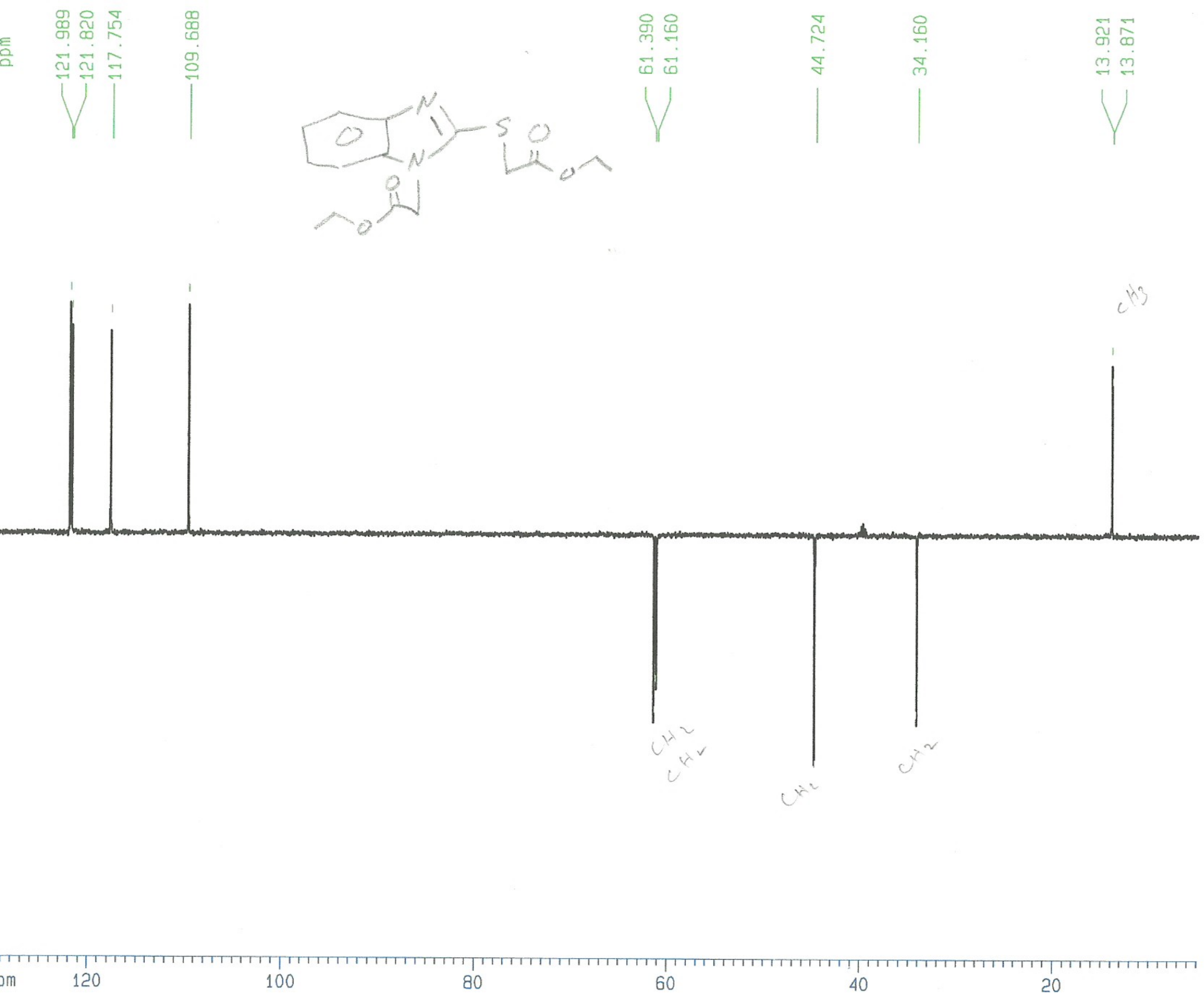

Current Data Parameters

NAME	sep09
EXPNO	7
PROCNO	1

F2 - Acquisition Parameters

Date_	20080909
Time	11.50
INSTRUM	spect
PROBHD	5 mm SEI 1H-13
PULPROG	deptsp135
TD	32768
SOLVENT	DMSO
NS	680
DS	2
SWH	30030.029 Hz
FIDRES	0.916444 Hz
AQ	0.5456539 sec
RG	32768
DW	16.650 usec
DE	50.00 usec
TE	300.0 K
CNST2	145.0000000
D1	1.50000000 sec
d2	0.00344828 sec
d12	0.00002000 sec
DELTA	0.00001846 sec

======== CHANNEL f1 ========

NUC1	13C
P1	14.50 usec
P12	2000.00 usec
PL0	120.00 dB
PL1	0.00 dB
SFO1	125.7715724 MHz
SP2	1.99 dB
SPNAM2	Crp60comp.4
SPOFF2	0.00 Hz

======== CHANNEL f2 ========

CPDPRG2	waltz16
NUC2	1H
P3	6.60 usec
p4	13.20 usec
PCPD2	100.00 usec
PL2	5.00 dB
PL12	23.00 dB
SFO2	500.1325006 MHz

F2 - Processing parameters

SI	16384
SF	125.7578568 MHz
WDW	EM
SSB	0
LB	1.50 Hz
GB	0
PC	1.40

1D NMR plot parameters

CX	20.00 cm
CY	4.00 cm
F1P	130.474 ppm
F1	16408.18 Hz
F2P	4.909 ppm
F2	617.31 Hz
PPMCM	6.27828 ppm/cm
HZCM	789.54327 Hz/cm

5

AVANCE AV 300
LAB. No. 115

AAMER/DR.EL ASHRY/MRA.85/DMSO

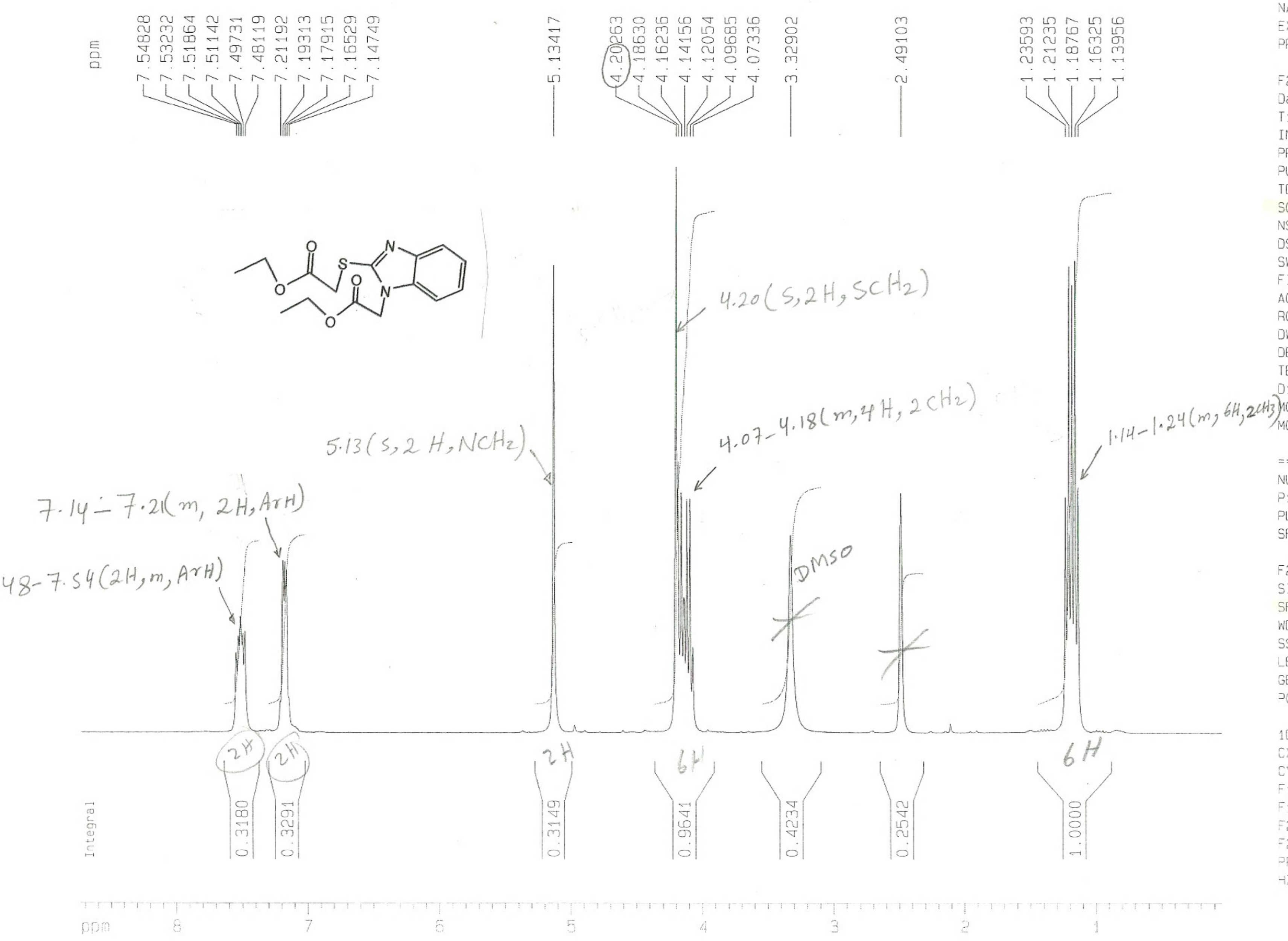

Current Data Parameters
NAME sep02
EXPNO 4
PROCNO 1

F2 - Acquisition Parameters
Date_ 20080902
Time 9.45
INSTRUM spect
PROBHD 5 mm DUL 13C-1
PULPROG zg30
TD 32768
SOLVENT DMSO
NS 128
DS 0
SWH 5995.204 Hz
FIDRES 0.182959 Hz
AQ 2.7329011 sec
RG 456.1
DW 83.400 usec
DE 10.00 usec
TE 0.0 K
D1 1.00000000 sec
MCREST 0.00000000 sec
MCWRK 0.01500000 sec

======== CHANNEL f1 ========
NUC1 1H
P1 10.20 usec
PL1 -3.00 dB
SFO1 300.1324010 MHz

F2 - Processing parameters
SI 16384
SF 300.1300042 MHz
WDW EM
SSB 0
LB 0.30 Hz
GB 0
PC 1.00

1D NMR plot parameters
CX 20.00 cm
CY 10.00 cm
F1P 8.730 ppm
F1 2620.28 Hz
F2P 0.043 ppm
F2 12.80 Hz
PPMCM 0.43439 ppm/cm
HZCM 130.37401 Hz/cm

File: MRA-85 **Date Run:** 09-01-2008 (Time Run: 10:15:15)
Sample: M RAMADAN AMER
Instrument: JEOL MSRoute
Inlet: Direct Probe **Ionization mode:** EI+

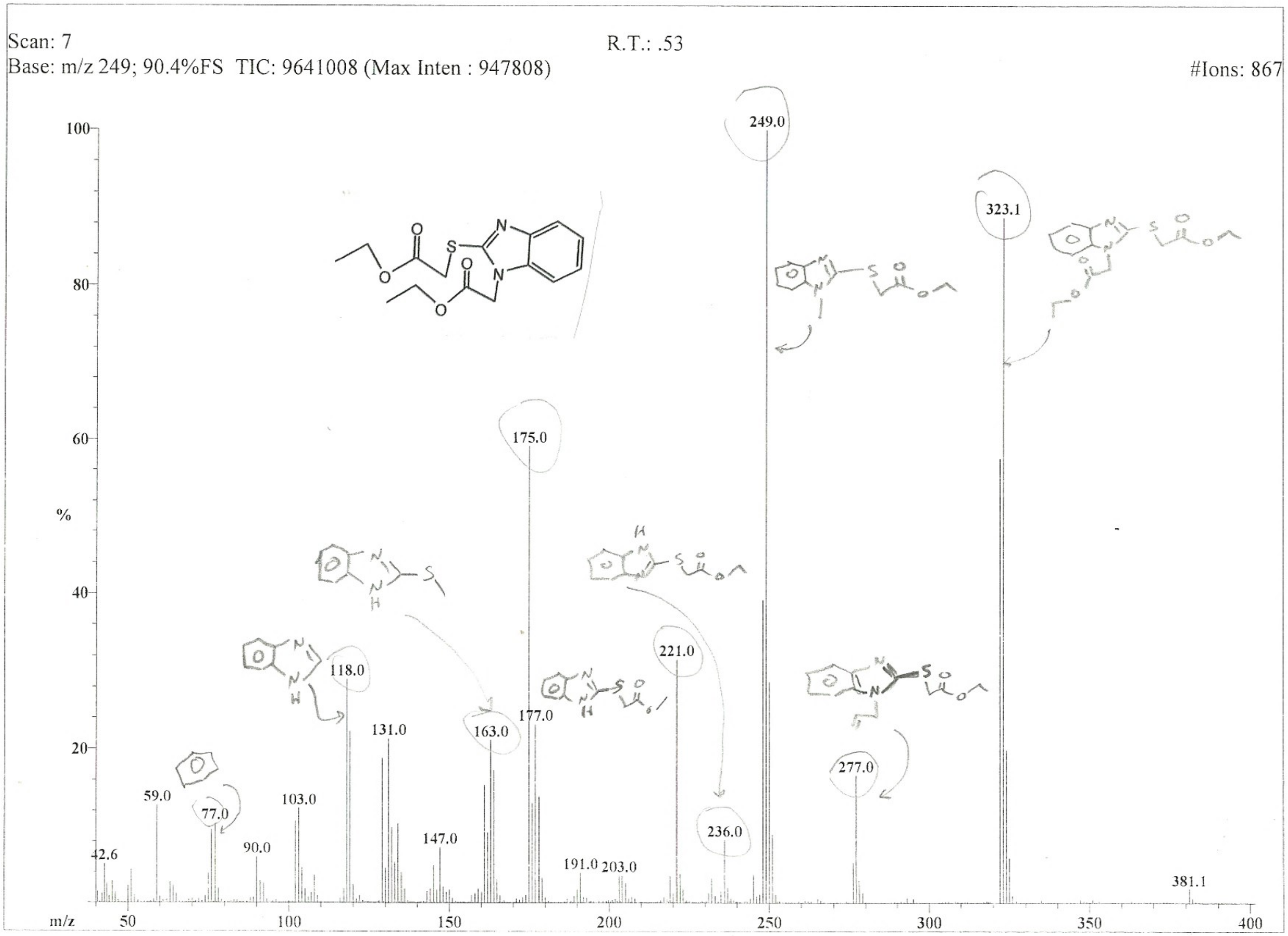

Mass	Relative Intensity	Theoretical Mass	Delta [ppm]	Delta [mmu]	RDB	Composition
		280.9810	3.3	0.9	18.5	$C_{17} H_{1} O_{1} N_{2} S_{1}$
		280.9808	3.9	1.1	10.0	$C_{10} H_{3} O_{9} N_{1}$
285.9833	1.0095	285.9835	-0.7	-0.2	8.5	$C_{9} H_{4} O_{10} N_{1}$
		285.9837	-1.3	-0.4	17.0	$C_{16} H_{2} O_{2} N_{2} S_{1}$
		285.9810	8.1	2.3	12.5	$C_{13} H_{4} O_{5} N_{1} S_{1}$
292.9819	12.0423	292.9815	1.4	0.4	1.5	$C_{5} H_{9} O_{12} S_{1}$
		292.9810	3.2	0.9	19.5	$C_{18} H_{1} O_{1} N_{2} S_{1}$
		292.9808	3.8	1.1	11.0	$C_{11} H_{3} O_{9} N_{1}$
304.9819	2.4054	304.9815	1.4	0.4	2.5	$C_{6} H_{9} O_{12} S_{1}$
		304.9810	3.0	0.9	20.5	$C_{19} H_{1} O_{1} N_{2} S_{1}$
		304.9808	3.6	1.1	12.0	$C_{12} H_{3} O_{9} N_{1}$
316.9808	1.8077	316.9808	0.0	0.0	13.0	$C_{13} H_{3} O_{9} N_{1}$
		316.9810	-0.5	-0.2	21.5	$C_{20} H_{1} O_{1} N_{2} S_{1}$
		316.9815	-2.1	-0.7	3.5	$C_{7} H_{9} O_{12} S_{1}$
318.9787	1.7064	318.9787	0.1	0.0	12.0	$C_{13} H_{5} O_{7} N_{1} S_{1}$
		318.9785	0.6	0.2	3.5	$C_{6} H_{7} O_{15}$
		318.9812	-7.8	-2.5	8.0	$C_{9} H_{5} O_{12} N_{1}$
322.0965	23.8791	322.0960	1.5	0.5	3.5	$C_{12} H_{20} O_{7} N_{1} S_{1}$
		322.0954	3.6	1.2	13.0	$C_{18} H_{14} O_{4} N_{2}$
		322.0987	-6.8	-2.2	8.0	$C_{15} H_{18} O_{4} N_{2} S_{1}$
323.1006	3.9683	323.1005	0.4	0.1	8.0	$C_{15} H_{17} O_{7} N_{1}$
		323.1032	-7.9	-2.6	12.5	$C_{18} H_{15} O_{4} N_{2}$
		323.0980	8.1	2.6	12.0	$C_{19} H_{17} O_{2} N_{1} S_{1}$
324.0976	1.3753	324.0973	1.1	0.3	16.0	$C_{23} H_{16} S_{1}$
		324.0991	-4.6	-1.5	3.0	$C_{11} H_{20} O_{7} N_{2} S_{1}$
		324.0958	5.8	1.9	8.0	$C_{14} H_{16} O_{7} N_{2}$
330.9787	6.7055	330.9787	0.1	0.0	13.0	$C_{14} H_{5} O_{7} N_{1} S_{1}$
		330.9785	0.6	0.2	4.5	$C_{7} H_{7} O_{15}$
		330.9812	-7.5	-2.5	9.0	$C_{10} H_{5} O_{12} N_{1}$
342.9787	5.2576	342.9787	0.1	0.0	14.0	$C_{15} H_{5} O_{7} N_{1} S_{1}$
		342.9785	0.6	0.2	5.5	$C_{8} H_{7} O_{15}$
		342.9812	-7.2	-2.5	10.0	$C_{11} H_{5} O_{12} N_{1}$
354.9787	2.4553	354.9787	0.1	0.0	15.0	$C_{16} H_{5} O_{7} N_{1} S_{1}$
		354.9785	0.6	0.2	6.5	$C_{9} H_{7} O_{15}$
		354.9812	-7.0	-2.5	11.0	$C_{12} H_{5} O_{12} N_{1}$
366.9780	1.3061	366.9785	-1.4	-0.5	7.5	$C_{10} H_{7} O_{15}$
		366.9787	-1.8	-0.7	16.0	$C_{17} H_{5} O_{7} N_{1} S_{1}$
		366.9760	5.5	2.0	11.5	$C_{14} H_{7} O_{10} S_{1}$
380.9755	4.3426	380.9757	-0.5	-0.2	17.0	$C_{17} H_{3} O_{10} N_{1}$
		380.9759	-1.0	-0.4	25.5	$C_{24} H_{1} O_{2} N_{2} S_{1}$
		380.9764	-2.3	-0.9	7.5	$C_{11} H_{9} O_{13} S_{1}$
392.9755	3.0940	392.9757	-0.5	-0.2	18.0	$C_{18} H_{3} O_{10} N_{1}$
		392.9759	-0.9	-0.4	26.5	$C_{25} H_{1} O_{2} N_{2} S_{1}$
		392.9749	1.6	0.6	0.5	$C_{3} H_{9} O_{20} N_{2}$

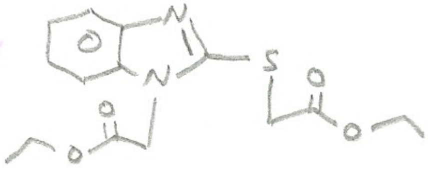

$C_{15}H_{18}N_2O_4S_1 = 32$

found = 322.0965

Calc. = 322.0987

Error = -6.8 ppm

$C_{15}H_{18}N_2O_4S_1$

	Formula	Calculated m/z (amu)	mDa Error	PPM Error	DBE
1	C14 H13 N2 S	241.0793	-0.3967	-1.6457	9.5
2	C17 H9 N2	241.0760	2.9752	12.3411	14.5
3	C11 H17 N2 S2	241.0827	-3.7687	-15.6326	4.5

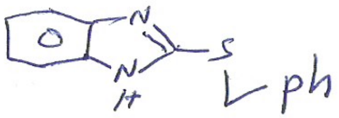

Calc. = 241.079[illegible]

Found = 241.0793

$C_{14}H_{13}N_2S$

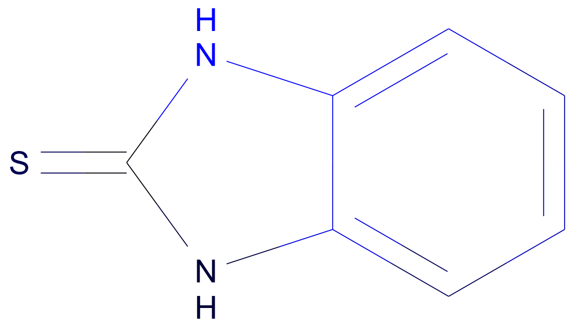

$C_7H_6N_2S$
Molecular Weight: 150.20094
ALogP: 2.536
Rotatable Bonds: 0
Acceptors: 1
Donors: 2

Model Prediction

Prediction: Non-Degradable
Probability: 0.147
Enrichment: 0.337
Bayesian Score: -9.17
Mahalanobis Distance: 9.74
Mahalanobis Distance p-value: 0.278

Prediction: Positive if the Bayesian score is above the estimated best cutoff value from minimizing the false positive and false negative rate.
Probability: The esimated probability that the sample is in the positive category. This assumes that the Bayesian score follows a normal distribution and is different from the prediction using a cutoff.
Enrichment: An estimate of enrichment, that is, the increased likelihood (versus random) of this sample being in the category.
Bayesian Score: The standard Laplacian-modified Bayesian score.
Mahalanobis Distance: The Mahalanobis distance (MD) is the distance to the center of the training data. The larger the MD, the less trustworthy the prediction.
Mahalanobis Distance p-value: The p-value gives the fraction of training data with an MD greater than or equal to the one for the given sample, assuming normally distributed data. The smaller the p-value, the less trustworthy the prediciton. For highly non-normal X properties (e.g., fingerprints), the MD p-value is wildly inaccurate.

Structural Similar Compounds

Name	1,2-Benzenediamine	Phenol,_2-amino-	Phenol,_3-amino-
Structure	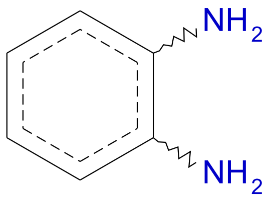	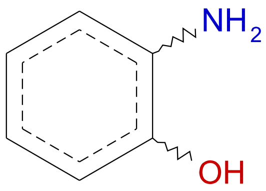	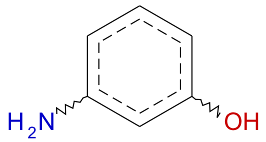
Actual Endpoint	Non-Degradable	Non-Degradable	Non-Degradable
Predicted Endpoint	Non-Degradable	Non-Degradable	Non-Degradable
Distance	0.555	0.562	0.586
Reference	Environmental Toxicology & Chemistry 18(9), 1763-1768, 1999.	Environmental Toxicology & Chemistry 18(9), 1763-1768, 1999.	Environmental Toxicology & Chemistry 18(9), 1763-1768, 1999.

Model Applicability

Unknown features are fingerprint features in the query molecule, but not found or appearing too infrequently in the training set.

1. All properties and OPS components are within expected ranges.

Feature Contribution

Top features for positive contribution

Fingerprint	Bit/Smiles	Feature Structure	Score	Degradable in training set
SCFP_12	1	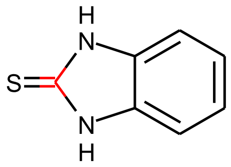 [*]C(=[*])[*]	0.359	194 out of 333

Top Features for negative contribution

Fingerprint	Bit/Smiles	Feature Structure	Score	Degradable in training set

SCFP_12	1334669481	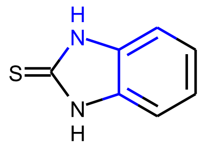<br>[*]:[cH]:[c]1N[*][*][c]:1:[*]	-1.63	10 out of 136
SCFP_12	18	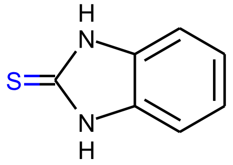<br>[*]=S	-1.62	0 out of 10
SCFP_12	10	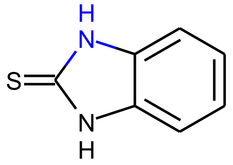<br>[*]N[*]	-1.61	11 out of 145

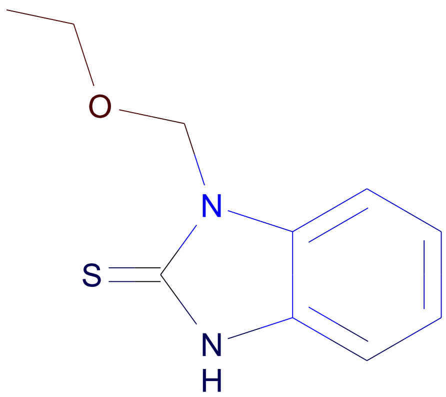

$C_{10}H_{12}N_2OS$
Molecular Weight: 208.28008
ALogP: 3.09
Rotatable Bonds: 3
Acceptors: 2
Donors: 1

Structural Similar Compounds

Name	4-chloro-1-nitro-2(trifluoromethyl)benzene	1-Naphthaleneacetic_acid	Benzoic_acid,_4-(1-methylethyl)-
Structure	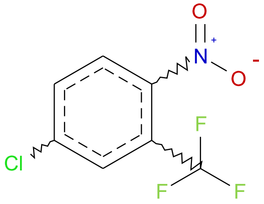	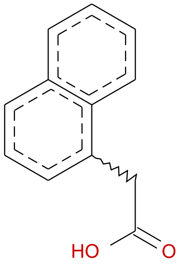	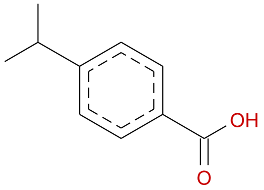
Actual Endpoint	Non-Degradable	Non-Degradable	Degradable
Predicted Endpoint	Non-Degradable	Non-Degradable	Degradable
Distance	0.546	0.558	0.564
Reference	Environmental Toxicology & Chemistry 18(9), 1763-1768, 1999.	Environmental Toxicology & Chemistry 18(9), 1763-1768, 1999.	Environmental Toxicology & Chemistry 18(9), 1763-1768, 1999.

Model Prediction

Prediction: Non-Degradable
Probability: 0.205
Enrichment: 0.47
Bayesian Score: -6.99
Mahalanobis Distance: 12.2
Mahalanobis Distance p-value: 0.000136

Prediction: Positive if the Bayesian score is above the estimated best cutoff value from minimizing the false positive and false negative rate.
Probability: The esimated probability that the sample is in the positive category. This assumes that the Bayesian score follows a normal distribution and is different from the prediction using a cutoff.
Enrichment: An estimate of enrichment, that is, the increased likelihood (versus random) of this sample being in the category.
Bayesian Score: The standard Laplacian-modified Bayesian score.
Mahalanobis Distance: The Mahalanobis distance (MD) is the distance to the center of the training data. The larger the MD, the less trustworthy the prediction.
Mahalanobis Distance p-value: The p-value gives the fraction of training data with an MD greater than or equal to the one for the given sample, assuming normally distributed data. The smaller the p-value, the less trustworthy the prediciton. For highly non-normal X properties (e.g., fingerprints), the MD p-value is wildly inaccurate.

Model Applicability

Unknown features are fingerprint features in the query molecule, but not found or appearing too infrequently in the training set.

1. All properties and OPS components are within expected ranges.

Feature Contribution

Top features for positive contribution

Fingerprint	Bit/Smiles	Feature Structure	Score	Degradable in training set
SCFP_12	-711686199	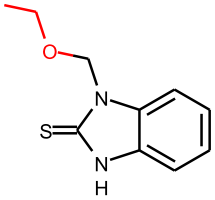 [*]OCC	0.49	106 out of 159

SCFP_12	-414063523	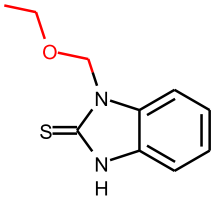 [*]COCC	0.422	3 out of 4
SCFP_12	136597326	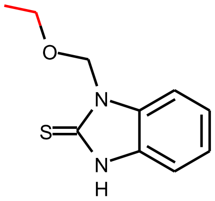 [*]CC	0.36	179 out of 307
**Top Features for negative contribution**				
**Fingerprint**	**Bit/Smiles**	**Feature Structure**	**Score**	**Degradable in training set**
SCFP_12	1334669481	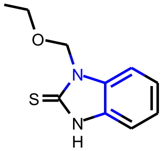 [*]:[cH]:[c]1N[*][*][c]:1:[*]	-1.63	10 out of 136
SCFP_12	18	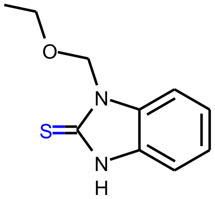 [*]=S	-1.62	0 out of 10
SCFP_12	10	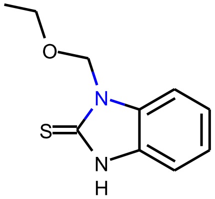 [*]N[*]	-1.61	11 out of 145

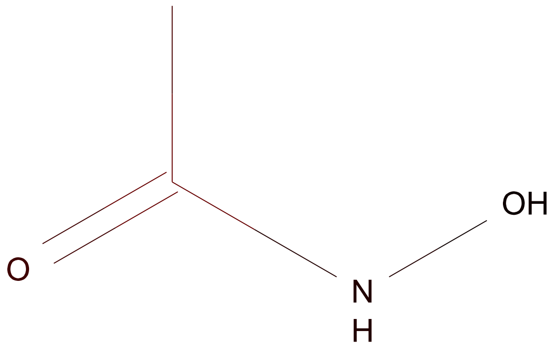

$C_2H_5NO_2$
Molecular Weight: 75.0666
ALogP: -0.82
Rotatable Bonds: 0
Acceptors: 2
Donors: 2

Model Prediction

Prediction: Degradable
Probability: 0.633
Enrichment: 1.45
Bayesian Score: 3.03
Mahalanobis Distance: 8.48
Mahalanobis Distance p-value: 0.867

Prediction: Positive if the Bayesian score is above the estimated best cutoff value from minimizing the false positive and false negative rate.
Probability: The esimated probability that the sample is in the positive category. This assumes that the Bayesian score follows a normal distribution and is different from the prediction using a cutoff.
Enrichment: An estimate of enrichment, that is, the increased likelihood (versus random) of this sample being in the category.
Bayesian Score: The standard Laplacian-modified Bayesian score.
Mahalanobis Distance: The Mahalanobis distance (MD) is the distance to the center of the training data. The larger the MD, the less trustworthy the prediction.
Mahalanobis Distance p-value: The p-value gives the fraction of training data with an MD greater than or equal to the one for the given sample, assuming normally distributed data. The smaller the p-value, the less trustworthy the prediciton. For highly non-normal X properties (e.g., fingerprints), the MD p-value is wildly inaccurate.

Structural Similar Compounds

Name	Ethanol,_2-amino-	1,2-Ethanediol	Hydrazine,_methyl-
Structure	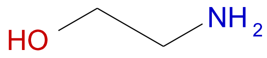	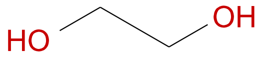	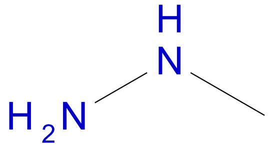
Actual Endpoint	Degradable	Degradable	Non-Degradable
Predicted Endpoint	Degradable	Degradable	Non-Degradable
Distance	0.492	0.507	0.527
Reference	Environmental Toxicology & Chemistry 18(9), 1763-1768, 1999.	Environmental Toxicology & Chemistry 18(9), 1763-1768, 1999.	Environmental Toxicology & Chemistry 18(9), 1763-1768, 1999.

Model Applicability

Unknown features are fingerprint features in the query molecule, but not found or appearing too infrequently in the training set.

1. All properties and OPS components are within expected ranges.

Feature Contribution

Top features for positive contribution

Fingerprint	Bit/Smiles	Feature Structure	Score	Degradable in training set
SCFP_12	136627117	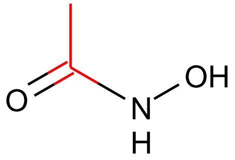 [*]C(=[*])C	0.662	56 out of 70

SCFP_12	1256995004	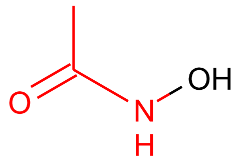 [*]NC(=O)C	0.54	23 out of 32
SCFP_12	1311071855	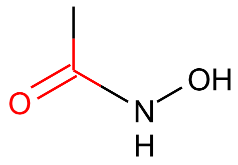 [*]C(=O)[*]	0.461	173 out of 268

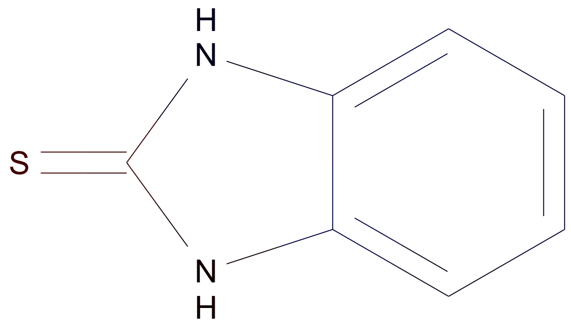

$C_7H_6N_2S$
Molecular Weight: 150.20094
ALogP: 2.536
Rotatable Bonds: 0
Acceptors: 1
Donors: 2

Structural Similar Compounds

Name	m-Aminophenol	o-Chloro-p-phenylenediamine	p-Phenylenediamine
Structure	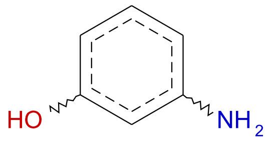	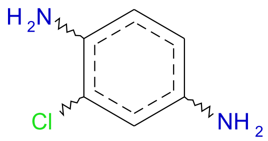	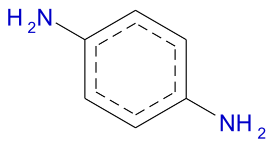
Actual Endpoint	Non-Toxic	Non-Toxic	Non-Toxic
Predicted Endpoint	Non-Toxic	Non-Toxic	Non-Toxic
Distance	0.578	0.586	0.611
Reference	Fundam Appl Toxicol 4:98-104; 1984	Food Chem Toxicol 22(2):147-9; 1984	Fundam Appl Toxicol 1:421-425; 1981

Model Prediction

Prediction: Non-Toxic
Probability: 0.53
Enrichment: 1.01
Bayesian Score: -0.46
Mahalanobis Distance: 8.09
Mahalanobis Distance p-value: 0.564

Prediction: Positive if the Bayesian score is above the estimated best cutoff value from minimizing the false positive and false negative rate.
Probability: The esimated probability that the sample is in the positive category. This assumes that the Bayesian score follows a normal distribution and is different from the prediction using a cutoff.
Enrichment: An estimate of enrichment, that is, the increased likelihood (versus random) of this sample being in the category.
Bayesian Score: The standard Laplacian-modified Bayesian score.
Mahalanobis Distance: The Mahalanobis distance (MD) is the distance to the center of the training data. The larger the MD, the less trustworthy the prediction.
Mahalanobis Distance p-value: The p-value gives the fraction of training data with an MD greater than or equal to the one for the given sample, assuming normally distributed data. The smaller the p-value, the less trustworthy the prediciton. For highly non-normal X properties (e.g., fingerprints), the MD p-value is wildly inaccurate.

Model Applicability

Unknown features are fingerprint features in the query molecule, but not found or appearing too infrequently in the training set.

1. All properties and OPS components are within expected ranges.

Feature Contribution

Top features for positive contribution

Fingerprint	Bit/Smiles	Feature Structure	Score	Toxic in training set
SCFP_6	1435188938	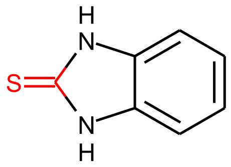 [*]C(=S)[*]	0.478	4 out of 4

SCFP_6	382734644	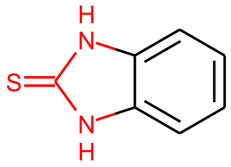<br>S=C1N[*]:[*]N1	0.478	4 out of 4
SCFP_6	18	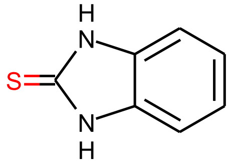<br>[*]=S	0.298	6 out of 8

Top Features for negative contribution

Fingerprint	Bit/Smiles	Feature Structure	Score	Toxic in training set
SCFP_6	-1380909229	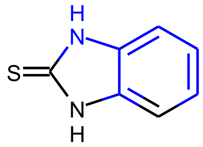<br>[*]1[*][c]2:[*]:[cH]:[cH]:[cH]:[c]:2N1	-0.449	6 out of 19
SCFP_6	1334669481	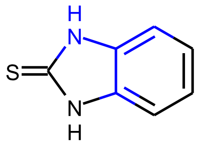<br>[*]:[cH]:[c]1N[*][*][c]:1:[*]	-0.355	10 out of 28
SCFP_6	-1379591900	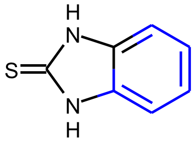<br>[*][c]1:[*]:[cH]:[cH]:[cH]:[cH]:1	-0.282	33 out of 84

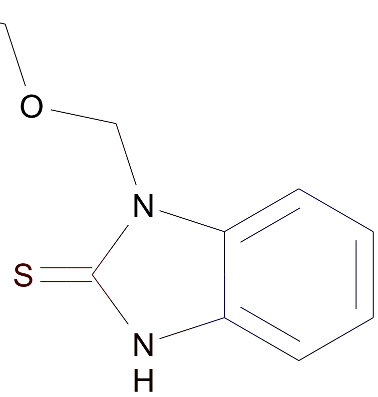

$C_{10}H_{12}N_2OS$
Molecular Weight: 208.28008
ALogP: 3.09
Rotatable Bonds: 3
Acceptors: 2
Donors: 1

Structural Similar Compounds

Name	2;4;5-Trichlorophenoxyacetic Acid	Carbaryl	Miroprofen
Structure	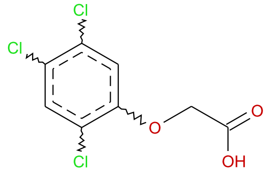	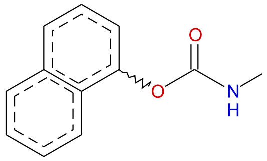	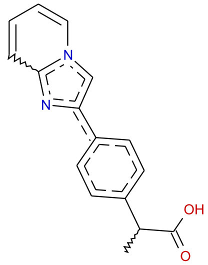
Actual Endpoint	Non-Toxic	Non-Toxic	Non-Toxic
Predicted Endpoint	Non-Toxic	Non-Toxic	Non-Toxic
Distance	0.574	0.582	0.588
Reference	Toxicol Appl Pharmacol 20(3):396-403; 1971	Toxicol Appl Pharmacol 26(4):621-38; 1973	Iyakuhin Kenkyu 12:808-826; 1981

Model Prediction

Prediction: Toxic
Probability: 0.55
Enrichment: 1.05
Bayesian Score: 0.0732
Mahalanobis Distance: 9.61
Mahalanobis Distance p-value: 0.0538

Prediction: Positive if the Bayesian score is above the estimated best cutoff value from minimizing the false positive and false negative rate.
Probability: The esimated probability that the sample is in the positive category. This assumes that the Bayesian score follows a normal distribution and is different from the prediction using a cutoff.
Enrichment: An estimate of enrichment, that is, the increased likelihood (versus random) of this sample being in the category.
Bayesian Score: The standard Laplacian-modified Bayesian score.
Mahalanobis Distance: The Mahalanobis distance (MD) is the distance to the center of the training data. The larger the MD, the less trustworthy the prediction.
Mahalanobis Distance p-value: The p-value gives the fraction of training data with an MD greater than or equal to the one for the given sample, assuming normally distributed data. The smaller the p-value, the less trustworthy the prediciton. For highly non-normal X properties (e.g., fingerprints), the MD p-value is wildly inaccurate.

Model Applicability

Unknown features are fingerprint features in the query molecule, but not found or appearing too infrequently in the training set.

1. All properties and OPS components are within expected ranges.

Feature Contribution

Top features for positive contribution				
**Fingerprint**	**Bit/Smiles**	**Feature Structure**	**Score**	**Toxic in training set**
SCFP_6	382734644	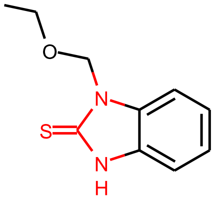 S=C1N[*]:[*]N1	0.478	4 out of 4

SCFP_6	1435188938	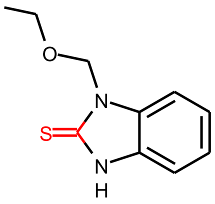 [*]C(=S)[*]	0.478	4 out of 4
SCFP_6	-110814626	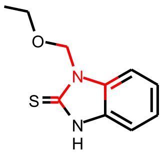 [*]CN1C(=[*])[*][*]:[c]1:[*]	0.381	2 out of 2
**Top Features for negative contribution**				
**Fingerprint**	**Bit/Smiles**	**Feature Structure**	**Score**	**Toxic in training set**
SCFP_6	-1380909229	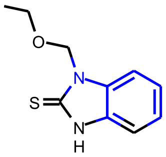 [*]1[*][c]2:[*]:[cH]: [cH]:[cH]:[c]:2N1	-0.449	6 out of 19
SCFP_6	1334669481	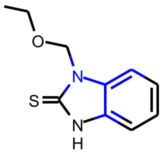 [*]:[cH]:[c]1N[*][*][c]:1:[*]	-0.355	10 out of 28
SCFP_6	-1379591900	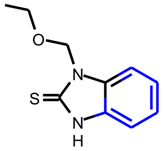 [*][c]1:[*]:[cH]:[cH] :[cH]:[cH]:1	-0.282	33 out of 84

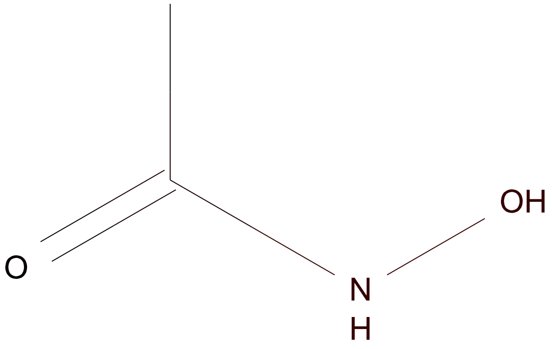

$C_2H_5NO_2$
Molecular Weight: 75.0666
ALogP: -0.82
Rotatable Bonds: 0
Acceptors: 2
Donors: 2

Model Prediction

Prediction: Toxic
Probability: 0.601
Enrichment: 1.14
Bayesian Score: 1.38
Mahalanobis Distance: 7.88
Mahalanobis Distance p-value: 0.663

Prediction: Positive if the Bayesian score is above the estimated best cutoff value from minimizing the false positive and false negative rate.
Probability: The esimated probability that the sample is in the positive category. This assumes that the Bayesian score follows a normal distribution and is different from the prediction using a cutoff.
Enrichment: An estimate of enrichment, that is, the increased likelihood (versus random) of this sample being in the category.
Bayesian Score: The standard Laplacian-modified Bayesian score.
Mahalanobis Distance: The Mahalanobis distance (MD) is the distance to the center of the training data. The larger the MD, the less trustworthy the prediction.
Mahalanobis Distance p-value: The p-value gives the fraction of training data with an MD greater than or equal to the one for the given sample, assuming normally distributed data. The smaller the p-value, the less trustworthy the prediciton. For highly non-normal X properties (e.g., fingerprints), the MD p-value is wildly inaccurate.

Structural Similar Compounds

Name	Methylhydrazine	Flucytosine	6-Aminonicotinamide
Structure	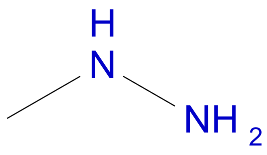	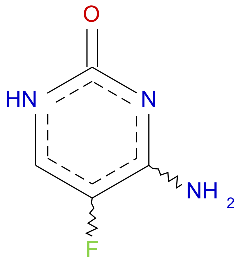	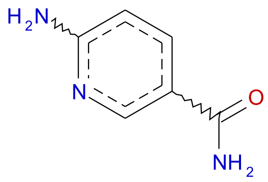
Actual Endpoint	Toxic	Toxic	Toxic
Predicted Endpoint	Toxic	Toxic	Toxic
Distance	0.520	0.541	0.563
Reference	J Toxicol Environ Health 13(1):125-31; 1984	Cancer Res 29(3):554-7; 1969	Teratology 1(1):103-8; 1968

Model Applicability

Unknown features are fingerprint features in the query molecule, but not found or appearing too infrequently in the training set.

1. All properties and OPS components are within expected ranges.

Feature Contribution

Top features for positive contribution

Fingerprint	Bit/Smiles	Feature Structure	Score	Toxic in training set
SCFP_6	450088063	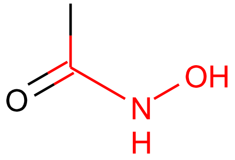 [*]C(=[*])NO	0.381	2 out of 2

SCFP_6	-424723671	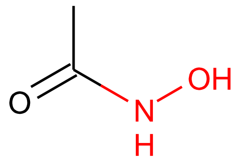 [*]NO	0.381	2 out of 2
SCFP_6	2094970867	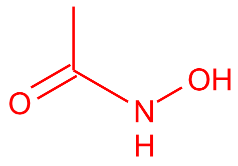 CC(=O)NO	0.271	1 out of 1

Top Features for negative contribution

Fingerprint	Bit/Smiles	Feature Structure	Score	Toxic in training set
SCFP_6	1256995004	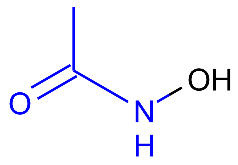 [*]NC(=O)C	-0.0998	17 out of 36
SCFP_6	136627117	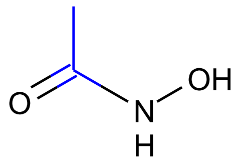 [*]C(=[*])C	-0.0885	10 out of 21
SCFP_6	1	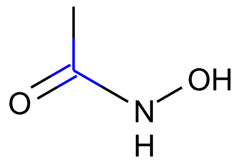 [*]C(=[*])[*]	0	90 out of 173

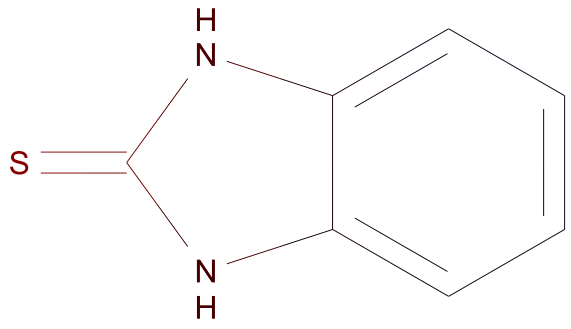

$C_7H_6N_2S$
Molecular Weight: 150.20094
ALogP: 2.536
Rotatable Bonds: 0
Acceptors: 1
Donors: 2

Structural Similar Compounds

Name	Propylthiouracil	Resorcinol	Hydroquinone
Structure	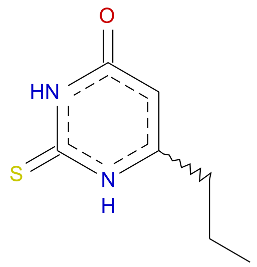	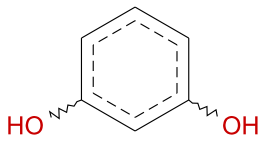	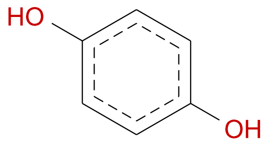
Actual Endpoint	Carcinogen	Non-Carcinogen	Carcinogen
Predicted Endpoint	Carcinogen	Non-Carcinogen	Carcinogen
Distance	0.583	0.600	0.611
Reference	US FDA (Centre for Drug Eval.& Res./Off. Testing & Res.) Sept. 1997	US FDA (Centre for Drug Eval.& Res./Off. Testing & Res.) Sept. 1997	US FDA (Centre for Drug Eval.& Res./Off. Testing & Res.) Sept. 1997

Model Prediction

Prediction: Carcinogen
Probability: 0.368
Enrichment: 1.15
Bayesian Score: 3.67
Mahalanobis Distance: 6.32
Mahalanobis Distance p-value: 1

Prediction: Positive if the Bayesian score is above the estimated best cutoff value from minimizing the false positive and false negative rate.
Probability: The esimated probability that the sample is in the positive category. This assumes that the Bayesian score follows a normal distribution and is different from the prediction using a cutoff.
Enrichment: An estimate of enrichment, that is, the increased likelihood (versus random) of this sample being in the category.
Bayesian Score: The standard Laplacian-modified Bayesian score.
Mahalanobis Distance: The Mahalanobis distance (MD) is the distance to the center of the training data. The larger the MD, the less trustworthy the prediction.
Mahalanobis Distance p-value: The p-value gives the fraction of training data with an MD greater than or equal to the one for the given sample, assuming normally distributed data. The smaller the p-value, the less trustworthy the prediciton. For highly non-normal X properties (e.g., fingerprints), the MD p-value is wildly inaccurate.

Model Applicability

Unknown features are fingerprint features in the query molecule, but not found or appearing too infrequently in the training set.

1. All properties and OPS components are within expected ranges.

Feature Contribution

Top features for positive contribution

Fingerprint	Bit/Smiles	Feature Structure	Score	Carcinogen in training set
ECFP_6	-829433624	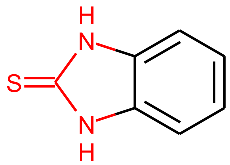 S=C1N[*]:[*]N1	0.73	3 out of 3

ECFP_6	-845108448	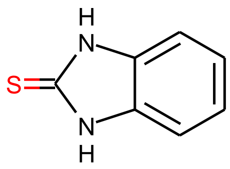<br><br>[*]=S	0.675	4 out of 5
ECFP_6	-1843135095	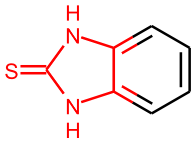<br><br>[*]:[c]1NC(=S)N[c]:1:[*]	0.617	2 out of 2

Top Features for negative contribution

Fingerprint	Bit/Smiles	Feature Structure	Score	Carcinogen in training set
ECFP_6	1997021792	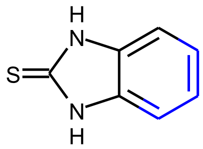<br><br>[*]:[cH]:[cH]:[cH]:[*]	-0.159	36 out of 137
ECFP_6	1335833675	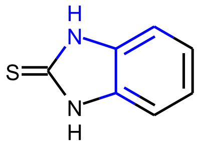<br><br>[*]:[cH]:[c]1N[*][*][c]:1:[*]	-0.147	2 out of 8
ECFP_6	1640720160	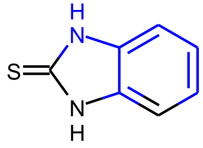<br><br>[*]1[*][c]2:[*]:[cH]:[cH]:[cH]:[c]:2N1	-0.112	1 out of 4

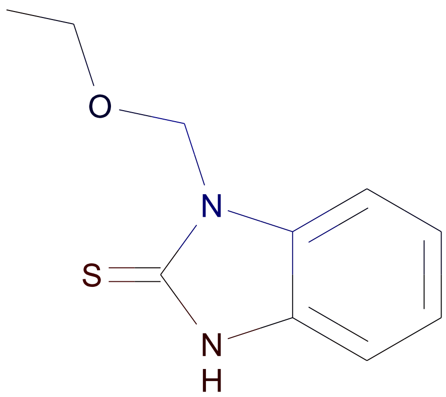

$C_{10}H_{12}N_2OS$
Molecular Weight: 208.28008
ALogP: 3.09
Rotatable Bonds: 3
Acceptors: 2
Donors: 1

Structural Similar Compounds

Name	Ketorolac	Mexiletine	Ketoprofen
Structure	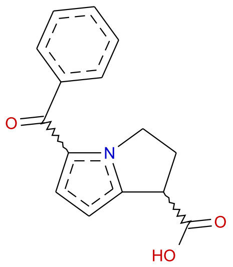	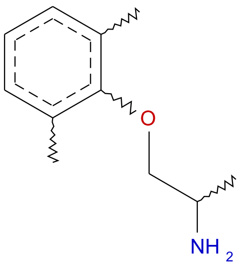	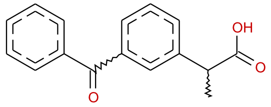
Actual Endpoint	Non-Carcinogen	Non-Carcinogen	Non-Carcinogen
Predicted Endpoint	Non-Carcinogen	Non-Carcinogen	Non-Carcinogen
Distance	0.593	0.601	0.611
Reference	US FDA (Centre for Drug Eval.& Res./Off. Testing & Res.) Sept. 1997	US FDA (Centre for Drug Eval.& Res./Off. Testing & Res.) Sept. 1997	US FDA (Centre for Drug Eval.& Res./Off. Testing & Res.) Sept. 1997

Model Prediction

Prediction: Non-Carcinogen
Probability: 0.236
Enrichment: 0.738
Bayesian Score: -1.56
Mahalanobis Distance: 10.4
Mahalanobis Distance p-value: 0.266

Prediction: Positive if the Bayesian score is above the estimated best cutoff value from minimizing the false positive and false negative rate.
Probability: The esimated probability that the sample is in the positive category. This assumes that the Bayesian score follows a normal distribution and is different from the prediction using a cutoff.
Enrichment: An estimate of enrichment, that is, the increased likelihood (versus random) of this sample being in the category.
Bayesian Score: The standard Laplacian-modified Bayesian score.
Mahalanobis Distance: The Mahalanobis distance (MD) is the distance to the center of the training data. The larger the MD, the less trustworthy the prediction.
Mahalanobis Distance p-value: The p-value gives the fraction of training data with an MD greater than or equal to the one for the given sample, assuming normally distributed data. The smaller the p-value, the less trustworthy the prediciton. For highly non-normal X properties (e.g., fingerprints), the MD p-value is wildly inaccurate.

Model Applicability

Unknown features are fingerprint features in the query molecule, but not found or appearing too infrequently in the training set.

1. All properties and OPS components are within expected ranges.
2. Unknown ECFP_2 feature: 434461827: [*]N1[*]:[*]NC1=S

Feature Contribution

Top features for positive contribution

Fingerprint	Bit/Smiles	Feature Structure	Score	Carcinogen in training set
ECFP_6	-845108448	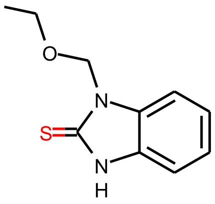 [*]=S	0.675	4 out of 5

ECFP_6	1985868180	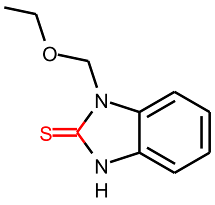<br>[*]C(=S)[*]	0.581	3 out of 4
ECFP_6	1639037056	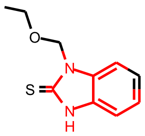<br>[*]N1C(=[*])N[c]2:[cH<br>]:[cH]:[*]:[cH]:[c]1<br>:2	0.424	1 out of 1

Top Features for negative contribution

**Fingerprint**	**Bit/Smiles**	**Feature Structure**	**Score**	**Carcinogen in training set**
ECFP_6	-661097313	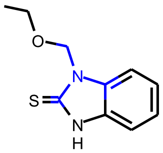<br>[*]CN1C(=[*])[*][*]:[<br>c]1:[*]	-1.55	0 out of 12
ECFP_6	-1252314528	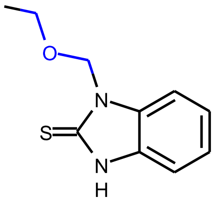<br>[*]COC[*]	-0.638	1 out of 9
ECFP_6	-1236953626	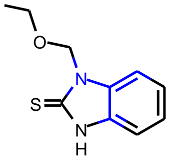<br>[*]N1[*][*][c](:[*]):<br>[c]1:[cH]:[*]	-0.448	3 out of 17

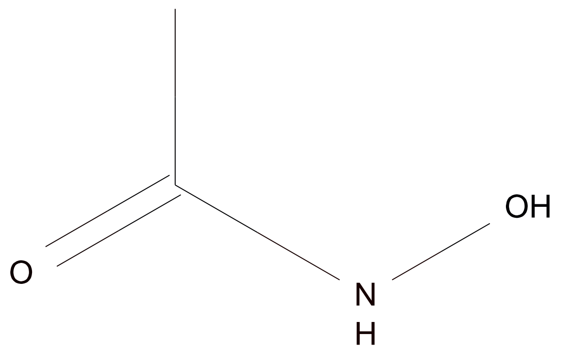

$C_2H_5NO_2$
Molecular Weight: 75.0666
ALogP: -0.82
Rotatable Bonds: 0
Acceptors: 2
Donors: 2

Model Prediction

Prediction: Carcinogen
Probability: 0.276
Enrichment: 0.86
Bayesian Score: 0.589
Mahalanobis Distance: 8.41
Mahalanobis Distance p-value: 0.966

Prediction: Positive if the Bayesian score is above the estimated best cutoff value from minimizing the false positive and false negative rate.
Probability: The esimated probability that the sample is in the positive category. This assumes that the Bayesian score follows a normal distribution and is different from the prediction using a cutoff.
Enrichment: An estimate of enrichment, that is, the increased likelihood (versus random) of this sample being in the category.
Bayesian Score: The standard Laplacian-modified Bayesian score.
Mahalanobis Distance: The Mahalanobis distance (MD) is the distance to the center of the training data. The larger the MD, the less trustworthy the prediction.
Mahalanobis Distance p-value: The p-value gives the fraction of training data with an MD greater than or equal to the one for the given sample, assuming normally distributed data. The smaller the p-value, the less trustworthy the prediciton. For highly non-normal X properties (e.g., fingerprints), the MD p-value is wildly inaccurate.

Structural Similar Compounds

Name	Acetamide	Thiouracil	Isoniazid
Structure	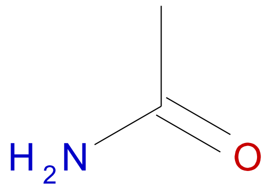	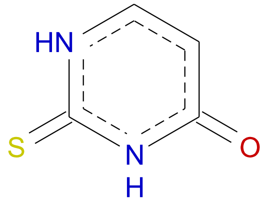	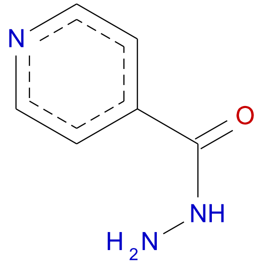
Actual Endpoint	Non-Carcinogen	Carcinogen	Carcinogen
Predicted Endpoint	Carcinogen	Carcinogen	Carcinogen
Distance	0.491	0.620	0.632
Reference	US FDA (Centre for Drug Eval.& Res./Off. Testing & Res.) Sept. 1997	US FDA (Centre for Drug Eval.& Res./Off. Testing & Res.) Sept. 1997	US FDA (Centre for Drug Eval.& Res./Off. Testing & Res.) Sept. 1997

Model Applicability

Unknown features are fingerprint features in the query molecule, but not found or appearing too infrequently in the training set.

1. All properties and OPS components are within expected ranges.
2. Unknown ECFP_2 feature: 379232550: [*]C(=[*])NO
3. Unknown ECFP_2 feature: 2023553742: [*]NO

Feature Contribution

Top features for positive contribution

Fingerprint	Bit/Smiles	Feature Structure	Score	Carcinogen in training set
ECFP_6	-474544785	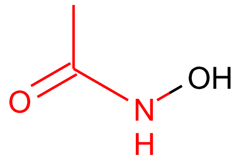 [*]NC(=O)C	0.442	2 out of 3

Top Features for negative contribution

Fingerprint	Bit/Smiles	Feature Structure	Score	Carcinogen in training set
ECFP_6	866218936	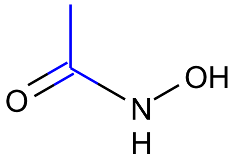 [*]C(=[*])C	-0.256	6 out of 26
ECFP_6	2099970318	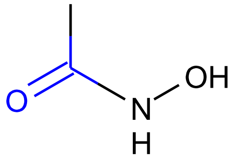 [*]C(=O)[*]	-0.166	36 out of 138
ECFP_6	-1100000244	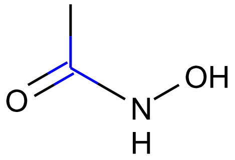 [*]C(=[*])[*]	-0.0874	43 out of 152

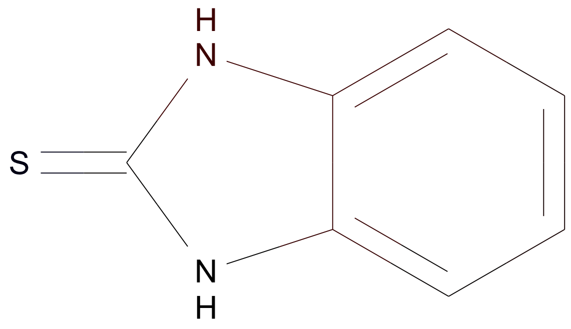

$C_7H_6N_2S$
Molecular Weight: 150.20094
ALogP: 2.536
Rotatable Bonds: 0
Acceptors: 1
Donors: 2

Model Prediction

Prediction: Multiple-Carcinogen
Probability: 0.394
Enrichment: 0.961
Bayesian Score: -0.186
Mahalanobis Distance: 6.54
Mahalanobis Distance p-value: 0.796

Prediction: Positive if the Bayesian score is above the estimated best cutoff value from minimizing the false positive and false negative rate.
Probability: The esimated probability that the sample is in the positive category. This assumes that the Bayesian score follows a normal distribution and is different from the prediction using a cutoff.
Enrichment: An estimate of enrichment, that is, the increased likelihood (versus random) of this sample being in the category.
Bayesian Score: The standard Laplacian-modified Bayesian score.
Mahalanobis Distance: The Mahalanobis distance (MD) is the distance to the center of the training data. The larger the MD, the less trustworthy the prediction.
Mahalanobis Distance p-value: The p-value gives the fraction of training data with an MD greater than or equal to the one for the given sample, assuming normally distributed data. The smaller the p-value, the less trustworthy the prediciton. For highly non-normal X properties (e.g., fingerprints), the MD p-value is wildly inaccurate.

Structural Similar Compounds

Name	Propylthiouracil	Hydroquinone	Methylthiouracil
Structure	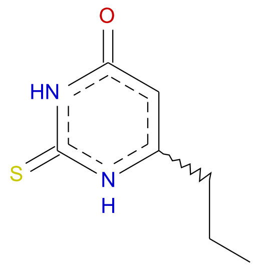	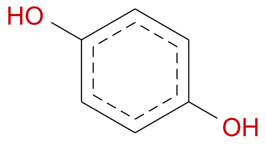	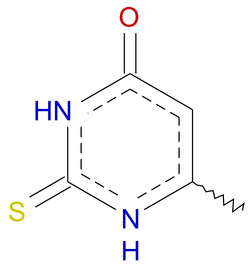
Actual Endpoint	Single-Carcinogen	Single-Carcinogen	Multiple-Carcinogen
Predicted Endpoint	Single-Carcinogen	Multiple-Carcinogen	Multiple-Carcinogen
Distance	0.553	0.596	0.598
Reference	US FDA (Centre for Drug Eval.& Res./Off. Testing & Res.) Sept. 1997	US FDA (Centre for Drug Eval.& Res./Off. Testing & Res.) Sept. 1997	US FDA (Centre for Drug Eval.& Res./Off. Testing & Res.) Sept. 1997

Model Applicability

Unknown features are fingerprint features in the query molecule, but not found or appearing too infrequently in the training set.

1. All properties and OPS components are within expected ranges.

Feature Contribution

Top features for positive contribution

Fingerprint	Bit/Smiles	Feature Structure	Score	Multiple-Carcinogen in training set
ECFP_4	1335833675	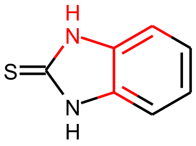 [*]:[cH]:[c]1N[*][*][c]:1:[*]	0.501	2 out of 2

ECFP_4	1640720160	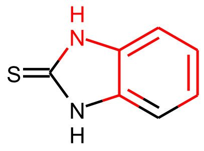 [*]1[*][c]2:[*]:[cH]:[cH]:[cH]:[c]:2N1	0.351	1 out of 1
ECFP_4	-154530762	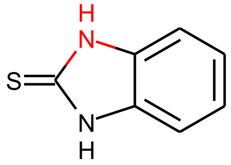 [*]N[*]	0.175	7 out of 14
**Top Features for negative contribution**				
**Fingerprint**	**Bit/Smiles**	**Feature Structure**	**Score**	**Multiple-Carcinogen in training set**
ECFP_4	-845108448	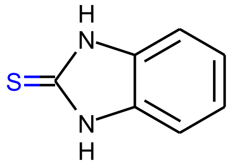 [*]=S	-0.275	1 out of 4
ECFP_4	1985868180	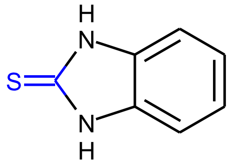 [*]C(=S)[*]	-0.107	1 out of 3
ECFP_4	-829433624	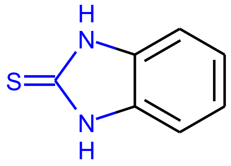 S=C1N[*]:[*]N1	-0.107	1 out of 3

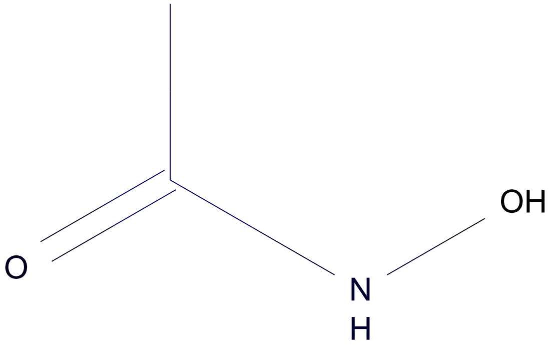

$C_2H_5NO_2$
Molecular Weight: 75.0666
ALogP: -0.82
Rotatable Bonds: 0
Acceptors: 2
Donors: 2

Model Prediction

Prediction: Multiple-Carcinogen
Probability: 0.343
Enrichment: 0.836
Bayesian Score: -1.64
Mahalanobis Distance: 8.95
Mahalanobis Distance p-value: 0.0845

Prediction: Positive if the Bayesian score is above the estimated best cutoff value from minimizing the false positive and false negative rate.
Probability: The esimated probability that the sample is in the positive category. This assumes that the Bayesian score follows a normal distribution and is different from the prediction using a cutoff.
Enrichment: An estimate of enrichment, that is, the increased likelihood (versus random) of this sample being in the category.
Bayesian Score: The standard Laplacian-modified Bayesian score.
Mahalanobis Distance: The Mahalanobis distance (MD) is the distance to the center of the training data. The larger the MD, the less trustworthy the prediction.
Mahalanobis Distance p-value: The p-value gives the fraction of training data with an MD greater than or equal to the one for the given sample, assuming normally distributed data. The smaller the p-value, the less trustworthy the prediciton. For highly non-normal X properties (e.g., fingerprints), the MD p-value is wildly inaccurate.

Structural Similar Compounds

Name	Thiouracil	Isoniazid	Methylthiouracil
Structure	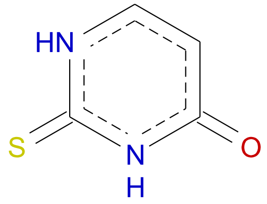	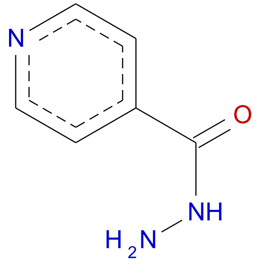	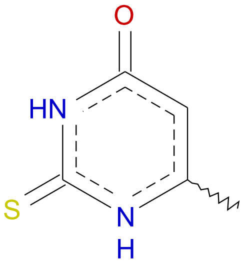
Actual Endpoint	Single-Carcinogen	Multiple-Carcinogen	Multiple-Carcinogen
Predicted Endpoint	Single-Carcinogen	Multiple-Carcinogen	Multiple-Carcinogen
Distance	0.616	0.626	0.644
Reference	US FDA (Centre for Drug Eval.& Res./Off. Testing & Res.) Sept. 1997	US FDA (Centre for Drug Eval.& Res./Off. Testing & Res.) Sept. 1997	US FDA (Centre for Drug Eval.& Res./Off. Testing & Res.) Sept. 1997

Model Applicability

Unknown features are fingerprint features in the query molecule, but not found or appearing too infrequently in the training set.

1. Molecular_Weight out of range. Value: 75.067. Training min, max, mean, SD: 107.15, 822.94, 288.14, 123.7.
2. Unknown ECFP_2 feature: 379232550: [*]C(=[*])NO
3. Unknown ECFP_2 feature: 2023553742: [*]NO

Feature Contribution

Top features for positive contribution

Fingerprint	Bit/Smiles	Feature Structure	Score	Multiple-Carcinogen in training set
ECFP_4	-1884411803	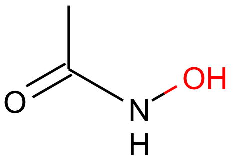 [*]O	0.0528	19 out of 44

Top Features for negative contribution				
**Fingerprint**	**Bit/Smiles**	**Feature Structure**	**Score**	**Multiple-Carcinogen in training set**
ECFP_4	866218936	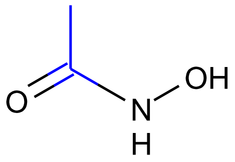 [*]C(=[*])C	-1.24	0 out of 6
ECFP_4	-474544785	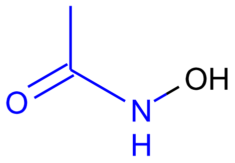 [*]NC(=O)C	-0.597	0 out of 2
ECFP_4	-1897341097	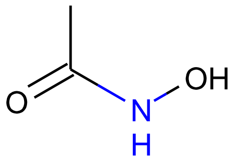 [*]N[*]	-0.356	8 out of 29

TOPKAT_Mouse_Male_FDA_None_vs_Carcinogen

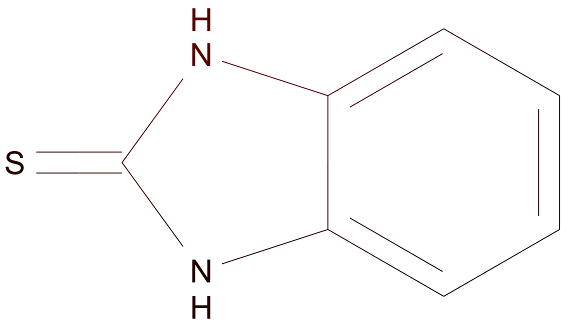

$C_7H_6N_2S$
Molecular Weight: 150.20094
ALogP: 2.536
Rotatable Bonds: 0
Acceptors: 1
Donors: 2

Structural Similar Compounds

Name	Phenicarbazide	Acetaminophen	Resorcinol
Structure	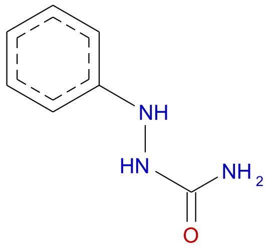	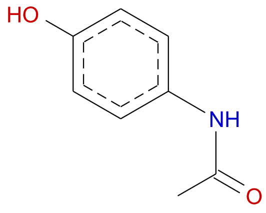	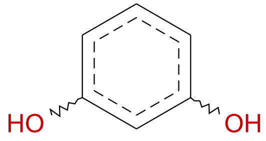
Actual Endpoint	Carcinogen	Carcinogen	Non-Carcinogen
Predicted Endpoint	Carcinogen	Carcinogen	Carcinogen
Distance	0.586	0.605	0.612
Reference	US FDA (Centre for Drug Eval.& Res./Off. Testing & Res.) Sept. 1997	US FDA (Centre for Drug Eval.& Res./Off. Testing & Res.) Sept. 1997	US FDA (Centre for Drug Eval.& Res./Off. Testing & Res.) Sept. 1997

Model Prediction

Prediction: Carcinogen
Probability: 0.42
Enrichment: 1.43
Bayesian Score: 3.56
Mahalanobis Distance: 8.67
Mahalanobis Distance p-value: 0.905

Prediction: Positive if the Bayesian score is above the estimated best cutoff value from minimizing the false positive and false negative rate.
Probability: The esimated probability that the sample is in the positive category. This assumes that the Bayesian score follows a normal distribution and is different from the prediction using a cutoff.
Enrichment: An estimate of enrichment, that is, the increased likelihood (versus random) of this sample being in the category.
Bayesian Score: The standard Laplacian-modified Bayesian score.
Mahalanobis Distance: The Mahalanobis distance (MD) is the distance to the center of the training data. The larger the MD, the less trustworthy the prediction.
Mahalanobis Distance p-value: The p-value gives the fraction of training data with an MD greater than or equal to the one for the given sample, assuming normally distributed data. The smaller the p-value, the less trustworthy the prediciton. For highly non-normal X properties (e.g., fingerprints), the MD p-value is wildly inaccurate.

Model Applicability

Unknown features are fingerprint features in the query molecule, but not found or appearing too infrequently in the training set.

1. All properties and OPS components are within expected ranges.

Feature Contribution

Top features for positive contribution

Fingerprint	Bit/Smiles	Feature Structure	Score	Carcinogen in training set
FCFP_6	1294255210	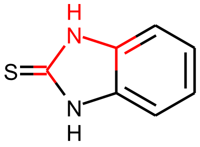 [*]=C1[*][*]:[c](:[*])N1	0.441	12 out of 28

FCFP_6	-773983804	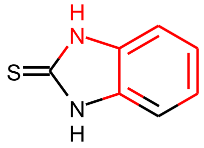 [*]1[*][c]2:[*]:[cH]:[cH]:[cH]:[c]:2N1	0.409	10 out of 24
FCFP_6	1499521844	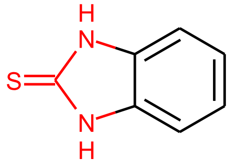 S=C1N[*]:[*]N1	0.388	9 out of 22

Top Features for negative contribution

Fingerprint	Bit/Smiles	Feature Structure	Score	Carcinogen in training set
FCFP_6	991735244	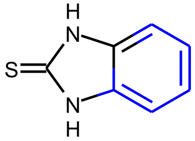 [*][c]1:[*]:[cH]:[cH]:[cH]:[cH]:1	-0.201	28 out of 131
FCFP_6	16	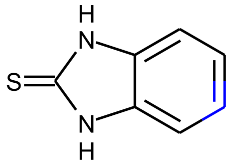 [*]:[cH]:[*]	0	65 out of 256
FCFP_6	1618154665	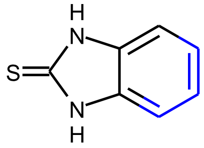 [*]:[cH]:[cH]:[cH]:[*]	0	59 out of 232

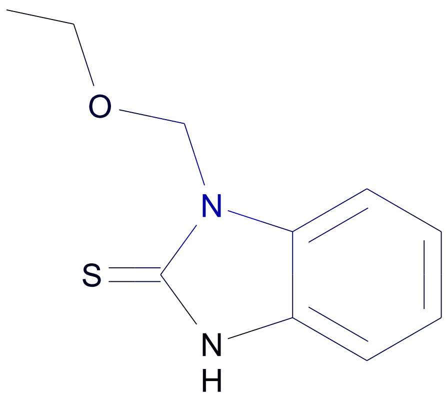

$C_{10}H_{12}N_2OS$
Molecular Weight: 208.28008
ALogP: 3.09
Rotatable Bonds: 3
Acceptors: 2
Donors: 1

Model Prediction

Prediction: Non-Carcinogen
Probability: 0.247
Enrichment: 0.841
Bayesian Score: -2.41
Mahalanobis Distance: 9.51
Mahalanobis Distance p-value: 0.601

Prediction: Positive if the Bayesian score is above the estimated best cutoff value from minimizing the false positive and false negative rate.
Probability: The esimated probability that the sample is in the positive category. This assumes that the Bayesian score follows a normal distribution and is different from the prediction using a cutoff.
Enrichment: An estimate of enrichment, that is, the increased likelihood (versus random) of this sample being in the category.
Bayesian Score: The standard Laplacian-modified Bayesian score.
Mahalanobis Distance: The Mahalanobis distance (MD) is the distance to the center of the training data. The larger the MD, the less trustworthy the prediction.
Mahalanobis Distance p-value: The p-value gives the fraction of training data with an MD greater than or equal to the one for the given sample, assuming normally distributed data. The smaller the p-value, the less trustworthy the prediciton. For highly non-normal X properties (e.g., fingerprints), the MD p-value is wildly inaccurate.

Structural Similar Compounds

Name	Phenacetin	Mexiletine	Ketorolac
Structure	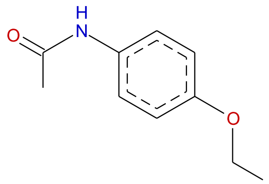	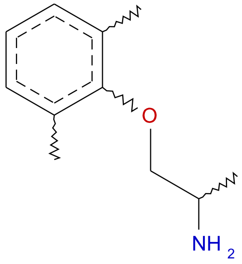	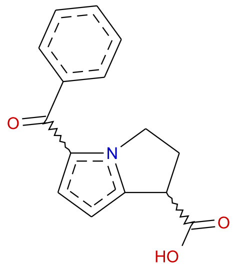
Actual Endpoint	Carcinogen	Non-Carcinogen	Non-Carcinogen
Predicted Endpoint	Carcinogen	Non-Carcinogen	Non-Carcinogen
Distance	0.571	0.572	0.581
Reference	US FDA (Centre for Drug Eval.& Res./Off. Testing & Res.) Sept. 1997	US FDA (Centre for Drug Eval.& Res./Off. Testing & Res.) Sept. 1997	US FDA (Centre for Drug Eval.& Res./Off. Testing & Res.) Sept. 1997

Model Applicability

Unknown features are fingerprint features in the query molecule, but not found or appearing too infrequently in the training set.

1. All properties and OPS components are within expected ranges.

Feature Contribution

Top features for positive contribution				
**Fingerprint**	**Bit/Smiles**	**Feature Structure**	**Score**	**Carcinogen in training set**
FCFP_6	1294255210	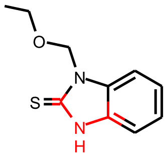 [*]=C1[*][*]:[c](:[*])N1	0.441	12 out of 28

FCFP_6	-773983804	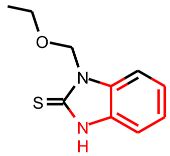 [*]1[*][c]2:[*]:[cH]:[cH]:[cH]:[c]:2N1	0.409	10 out of 24
FCFP_6	590925877	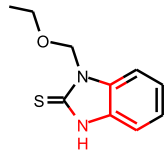 [*]:[cH]:[c]1N[*][*][c]:1:[*]	0.369	13 out of 33

Top Features for negative contribution

Fingerprint	Bit/Smiles	Feature Structure	Score	Carcinogen in training set
FCFP_6	470041467	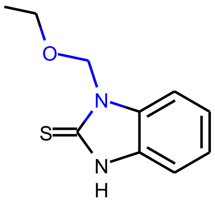 [*]OCN([*])[*]	-0.582	0 out of 3
FCFP_6	1176629869	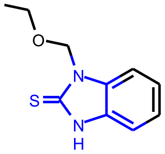 [*]N1C(=S)N[c](:[cH]:[*]):[c]1:[*]	-0.423	0 out of 2
FCFP_6	-2090462286	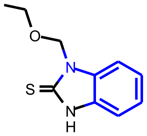 [*]N1[*][*][c]2:[cH]:[cH]:[cH]:[cH]:[c]1:2	-0.343	4 out of 23

HAE

TOPKAT_Mouse_Male_FDA_None_vs_Carcinogen

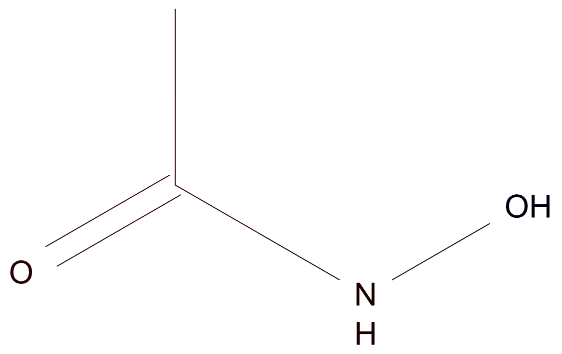

$C_2H_5NO_2$
Molecular Weight: 75.0666
ALogP: -0.82
Rotatable Bonds: 0
Acceptors: 2
Donors: 2

Model Prediction

Prediction: Carcinogen
Probability: 0.369
Enrichment: 1.25
Bayesian Score: 2.09
Mahalanobis Distance: 13.8
Mahalanobis Distance p-value: 1.22e-005

Prediction: Positive if the Bayesian score is above the estimated best cutoff value from minimizing the false positive and false negative rate.
Probability: The esimated probability that the sample is in the positive category. This assumes that the Bayesian score follows a normal distribution and is different from the prediction using a cutoff.
Enrichment: An estimate of enrichment, that is, the increased likelihood (versus random) of this sample being in the category.
Bayesian Score: The standard Laplacian-modified Bayesian score.
Mahalanobis Distance: The Mahalanobis distance (MD) is the distance to the center of the training data. The larger the MD, the less trustworthy the prediction.
Mahalanobis Distance p-value: The p-value gives the fraction of training data with an MD greater than or equal to the one for the given sample, assuming normally distributed data. The smaller the p-value, the less trustworthy the prediciton. For highly non-normal X properties (e.g., fingerprints), the MD p-value is wildly inaccurate.

Structural Similar Compounds

Name	Acetamide	Thiouracil	Methylthiouracil
Structure	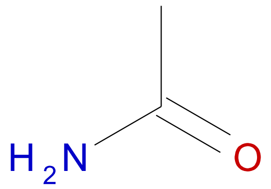	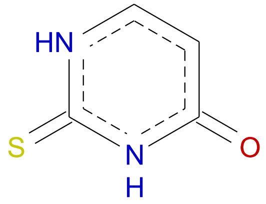	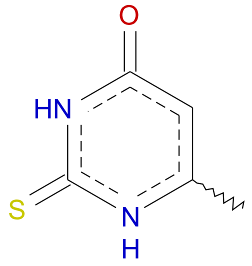
Actual Endpoint	Carcinogen	Carcinogen	Carcinogen
Predicted Endpoint	Carcinogen	Carcinogen	Carcinogen
Distance	0.398	0.527	0.558
Reference	US FDA (Centre for Drug Eval.& Res./Off. Testing & Res.) Sept. 1997	US FDA (Centre for Drug Eval.& Res./Off. Testing & Res.) Sept. 1997	US FDA (Centre for Drug Eval.& Res./Off. Testing & Res.) Sept. 1997

Model Applicability

Unknown features are fingerprint features in the query molecule, but not found or appearing too infrequently in the training set.

1. All properties and OPS components are within expected ranges.
2. Unknown FCFP_2 feature: 1666606459: [*]C(=[*])NO
3. Unknown FCFP_2 feature: -548542844: [*]NO

Feature Contribution

Top features for positive contribution

Fingerprint	Bit/Smiles	Feature Structure	Score	Carcinogen in training set
FCFP_6	566058135	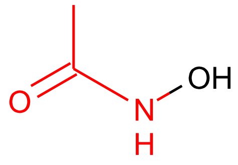 [*]NC(=O)C	0.447	17 out of 40

FCFP_6	1872154524	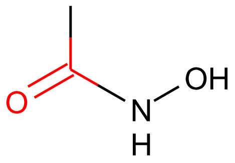 [*]C(=S)[*]	0.205	69 out of 213
FCFP_6	136597326	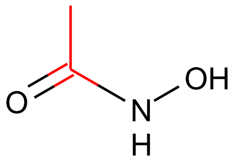 [*]CC	0.155	49 out of 159
**Top Features for negative contribution**				
**Fingerprint**	**Bit/Smiles**	**Feature Structure**	**Score**	**Carcinogen in training set**
FCFP_6	7	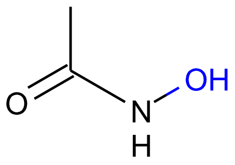 [*]O	-0.308	15 out of 79

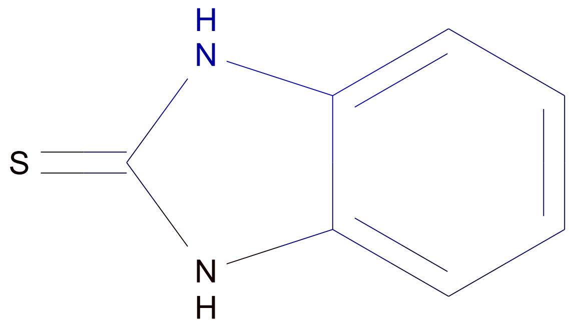

$C_7H_6N_2S$
Molecular Weight: 150.20094
ALogP: 2.536
Rotatable Bonds: 0
Acceptors: 1
Donors: 2

Model Prediction

Prediction: Single-Carcinogen
Probability: 0.163
Enrichment: 0.542
Bayesian Score: -5.36
Mahalanobis Distance: 9.77
Mahalanobis Distance p-value: 0.0263

Prediction: Positive if the Bayesian score is above the estimated best cutoff value from minimizing the false positive and false negative rate.
Probability: The esimated probability that the sample is in the positive category. This assumes that the Bayesian score follows a normal distribution and is different from the prediction using a cutoff.
Enrichment: An estimate of enrichment, that is, the increased likelihood (versus random) of this sample being in the category.
Bayesian Score: The standard Laplacian-modified Bayesian score.
Mahalanobis Distance: The Mahalanobis distance (MD) is the distance to the center of the training data. The larger the MD, the less trustworthy the prediction.
Mahalanobis Distance p-value: The p-value gives the fraction of training data with an MD greater than or equal to the one for the given sample, assuming normally distributed data. The smaller the p-value, the less trustworthy the prediciton. For highly non-normal X properties (e.g., fingerprints), the MD p-value is wildly inaccurate.

Structural Similar Compounds

Name	Acetaminophen	Phenicarbazide	Propylthiouracil
Structure	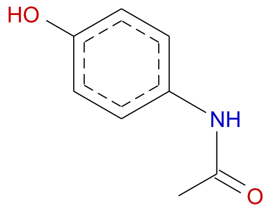	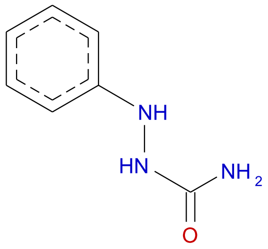	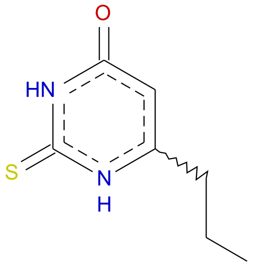
Actual Endpoint	Single-Carcinogen	Multiple-Carcinogen	Multiple-Carcinogen
Predicted Endpoint	Single-Carcinogen	Multiple-Carcinogen	Multiple-Carcinogen
Distance	0.606	0.626	0.628
Reference	US FDA (Centre for Drug Eval.& Res./Off. Testing & Res.) Sept. 1997	US FDA (Centre for Drug Eval.& Res./Off. Testing & Res.) Sept. 1997	US FDA (Centre for Drug Eval.& Res./Off. Testing & Res.) Sept. 1997

Model Applicability

Unknown features are fingerprint features in the query molecule, but not found or appearing too infrequently in the training set.

1. All properties and OPS components are within expected ranges.

Feature Contribution

Top features for positive contribution

Fingerprint	Bit/Smiles	Feature Structure	Score	Multiple-Carcinogen in training set
FCFP_12	1499521844	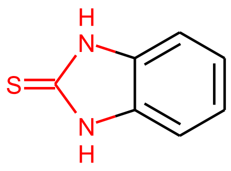 S=C1N[*]:[*]N1	0.39	5 out of 9

Top Features for negative contribution

Fingerprint	Bit/Smiles	Feature Structure	Score	Multiple-Carcinogen in training set
FCFP_12	1294255210	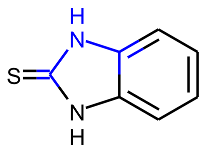 [*]=C1[*][*]:[c](:[*])N1	-1.63	0 out of 12
FCFP_12	590925877	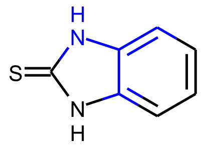 [*]:[cH]:[c]1N[*][*][c]:1:[*]	-0.998	1 out of 13
FCFP_12	-773983804	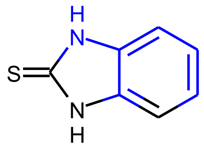 [*]1[*][c]2:[*]:[cH]:[cH]:[cH]:[c]:2N1	-0.789	1 out of 10

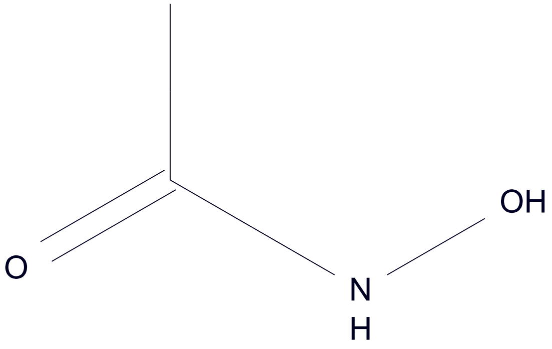

$C_2H_5NO_2$
Molecular Weight: 75.0666
ALogP: -0.82
Rotatable Bonds: 0
Acceptors: 2
Donors: 2

Model Prediction

Prediction: Multiple-Carcinogen
Probability: 0.221
Enrichment: 0.733
Bayesian Score: -1.53
Mahalanobis Distance: 10.6
Mahalanobis Distance p-value: 0.00655

Prediction: Positive if the Bayesian score is above the estimated best cutoff value from minimizing the false positive and false negative rate.
Probability: The esimated probability that the sample is in the positive category. This assumes that the Bayesian score follows a normal distribution and is different from the prediction using a cutoff.
Enrichment: An estimate of enrichment, that is, the increased likelihood (versus random) of this sample being in the category.
Bayesian Score: The standard Laplacian-modified Bayesian score.
Mahalanobis Distance: The Mahalanobis distance (MD) is the distance to the center of the training data. The larger the MD, the less trustworthy the prediction.
Mahalanobis Distance p-value: The p-value gives the fraction of training data with an MD greater than or equal to the one for the given sample, assuming normally distributed data. The smaller the p-value, the less trustworthy the prediciton. For highly non-normal X properties (e.g., fingerprints), the MD p-value is wildly inaccurate.

Structural Similar Compounds

Name	Acetamide	Thiouracil	Methylthiouracil
Structure	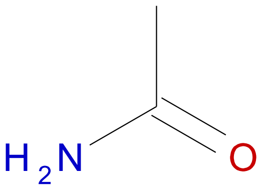	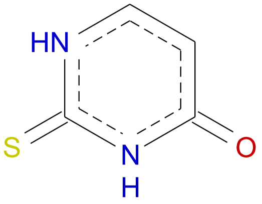	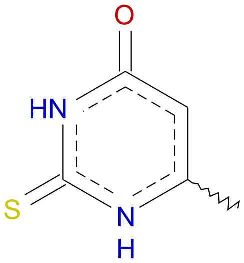
Actual Endpoint	Single-Carcinogen	Single-Carcinogen	Multiple-Carcinogen
Predicted Endpoint	Multiple-Carcinogen	Multiple-Carcinogen	Multiple-Carcinogen
Distance	0.445	0.524	0.552
Reference	US FDA (Centre for Drug Eval.& Res./Off. Testing & Res.) Sept. 1997	US FDA (Centre for Drug Eval.& Res./Off. Testing & Res.) Sept. 1997	US FDA (Centre for Drug Eval.& Res./Off. Testing & Res.) Sept. 1997

Model Applicability

Unknown features are fingerprint features in the query molecule, but not found or appearing too infrequently in the training set.

1. All properties and OPS components are within expected ranges.
2. Unknown FCFP_2 feature: 1666606459: [*]C(=[*])NO
3. Unknown FCFP_2 feature: -548542844: [*]NO

Feature Contribution

Top features for positive contribution

Fingerprint	Bit/Smiles	Feature Structure	Score	Multiple-Carcinogen in training set
FCFP_12	136597326	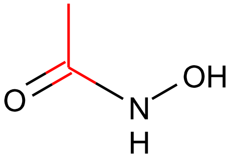 [*]C(=[*])C	0.0722	18 out of 49

Top Features for negative contribution

Fingerprint	Bit/Smiles	Feature Structure	Score	Multiple-Carcinogen in training set
FCFP_12	7	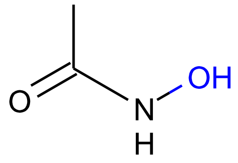 [*]O	-0.71	2 out of 15
FCFP_12	566058135	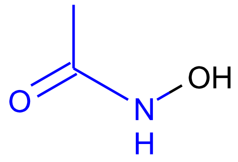 [*]NC(=O)C	-0.528	3 out of 17
FCFP_12	1	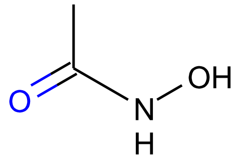 [*]=S	-0.113	23 out of 76

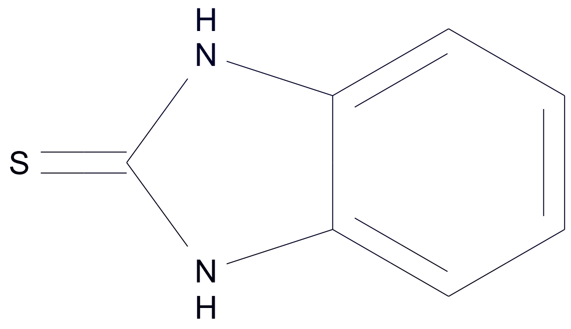

$C_7H_6N_2S$
Molecular Weight: 150.20094
ALogP: 2.536
Rotatable Bonds: 0
Acceptors: 1
Donors: 2

Structural Similar Compounds

Name	2-Benzimidazolethiol	ACETANILIDE; 2'-HYDROXY	Anthranilic acid; N-methyl-
Structure	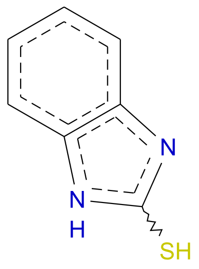	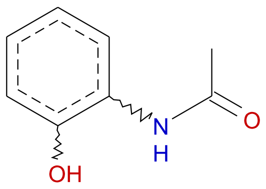	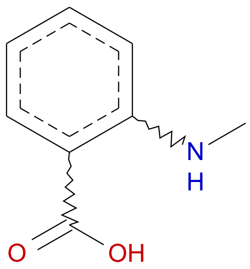
Actual Endpoint	Mild	Mild	Moderate_Severe
Predicted Endpoint	Mild	Mild	Mild
Distance	0.571	0.595	0.611
Reference	Prehled Prumyslove Toxikologie; Organicke Latky; Marhold; J. pp 990;86	28ZPAK-;106;72	Prehled Prumyslove Toxikologie; Organicke Latky; Marhold; J. -;741;86

Model Prediction

Prediction: Mild
Probability: 0.791
Enrichment: 1.15
Bayesian Score: -1.33
Mahalanobis Distance: 5.65
Mahalanobis Distance p-value: 1

Prediction: Positive if the Bayesian score is above the estimated best cutoff value from minimizing the false positive and false negative rate.
Probability: The esimated probability that the sample is in the positive category. This assumes that the Bayesian score follows a normal distribution and is different from the prediction using a cutoff.
Enrichment: An estimate of enrichment, that is, the increased likelihood (versus random) of this sample being in the category.
Bayesian Score: The standard Laplacian-modified Bayesian score.
Mahalanobis Distance: The Mahalanobis distance (MD) is the distance to the center of the training data. The larger the MD, the less trustworthy the prediction.
Mahalanobis Distance p-value: The p-value gives the fraction of training data with an MD greater than or equal to the one for the given sample, assuming normally distributed data. The smaller the p-value, the less trustworthy the prediciton. For highly non-normal X properties (e.g., fingerprints), the MD p-value is wildly inaccurate.

Model Applicability

Unknown features are fingerprint features in the query molecule, but not found or appearing too infrequently in the training set.

1. All properties and OPS components are within expected ranges.

Feature Contribution

Top features for positive contribution

Fingerprint	Bit/Smiles	Feature Structure	Score	Moderate_Severe in training set
FCFP_10	-402549409	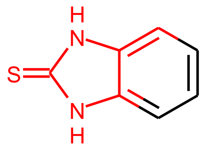 [*]:[cH]:[c]1NC(=S)N[c]:1:[*]	0.186	1 out of 1

FCFP_10	3	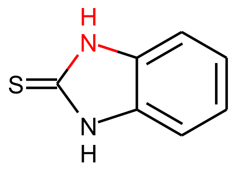 [*]N[*]	0.165	383 out of 491

Top Features for negative contribution

**Fingerprint**	**Bit/Smiles**	**Feature Structure**	**Score**	**Moderate_Severe in training set**
FCFP_10	1499521844	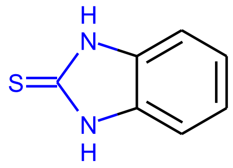 S=C1N[*]:[*]N1	-0.4	1 out of 3
FCFP_10	-773983804	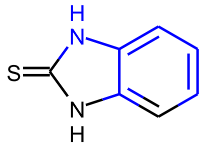 [*]1[*][c]2:[*]:[cH]:[cH]:[cH]:[c]:2N1	-0.294	50 out of 102
FCFP_10	1294255210	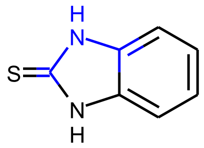 [*]=C1[*][*]:[c](:[*])N1	-0.218	20 out of 38

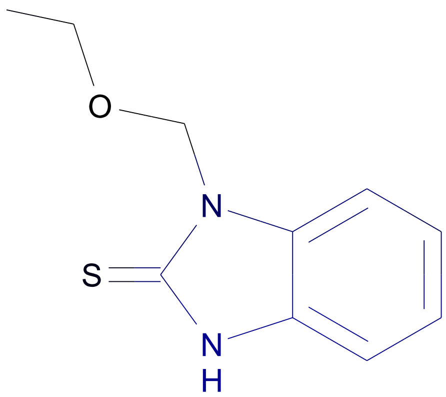

$C_{10}H_{12}N_2OS$
Molecular Weight: 208.28008
ALogP: 3.09
Rotatable Bonds: 3
Acceptors: 2
Donors: 1

Model Prediction

Prediction: Mild
Probability: 0.721
Enrichment: 1.05
Bayesian Score: -3.12
Mahalanobis Distance: 7.47
Mahalanobis Distance p-value: 0.986

Prediction: Positive if the Bayesian score is above the estimated best cutoff value from minimizing the false positive and false negative rate.
Probability: The esimated probability that the sample is in the positive category. This assumes that the Bayesian score follows a normal distribution and is different from the prediction using a cutoff.
Enrichment: An estimate of enrichment, that is, the increased likelihood (versus random) of this sample being in the category.
Bayesian Score: The standard Laplacian-modified Bayesian score.
Mahalanobis Distance: The Mahalanobis distance (MD) is the distance to the center of the training data. The larger the MD, the less trustworthy the prediction.
Mahalanobis Distance p-value: The p-value gives the fraction of training data with an MD greater than or equal to the one for the given sample, assuming normally distributed data. The smaller the p-value, the less trustworthy the prediciton. For highly non-normal X properties (e.g., fingerprints), the MD p-value is wildly inaccurate.

Structural Similar Compounds

Name	DIPHENYLAMINE; 4-NITRO	ANILINE;P-PHENOXY-	BENZOTHIAZOLE; 5-AMINO-2-PHENYL-
Structure	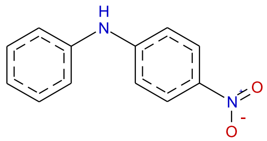	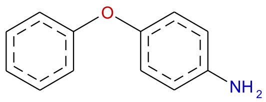	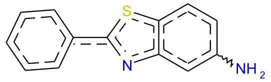
Actual Endpoint	Mild	Moderate_Severe	Moderate_Severe
Predicted Endpoint	Mild	Mild	Mild
Distance	0.517	0.540	0.546
Reference	28ZPAK-;134;72	28ZPAK-;119;72	28ZPAK -;203;72

Model Applicability

Unknown features are fingerprint features in the query molecule, but not found or appearing too infrequently in the training set.

1. All properties and OPS components are within expected ranges.

Feature Contribution

Top features for positive contribution

Fingerprint	Bit/Smiles	Feature Structure	Score	Moderate_Severe in training set
FCFP_10	1603272225	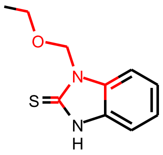 [*]COCN1C(=[*])[*][*]:[c]1:[*]	0.186	1 out of 1

FCFP_10	3	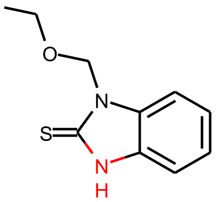 [*]N[*]	0.165	383 out of 491
FCFP_10	675769755	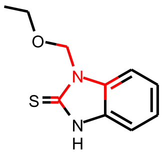 [*]CN1C(=[*])[*][*]:[c]1:[*]	0.116	12 out of 16
**Top Features for negative contribution**				
**Fingerprint**	**Bit/Smiles**	**Feature Structure**	**Score**	**Moderate_Severe in training set**
FCFP_10	-505031736	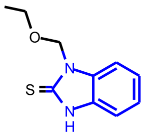 [*]N1C(=[*])N[c]2:[cH]:[cH]:[cH]:[cH]:[c] 1:2	-0.842	0 out of 2
FCFP_10	-1155471474	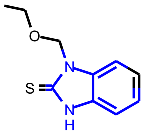 [*]N1C(=[*])N[c]2:[cH]:[cH]:[*]:[cH]:[c]1 :2	-0.842	0 out of 2
FCFP_10	-1986098826	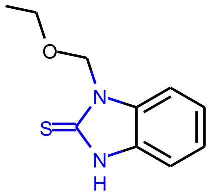 [*]N1[*]:[*]NC1=S	-0.361	2 out of 5

HAE   TOPKAT_Ocular_Irritancy_Mild_vs_Moderate_Severe

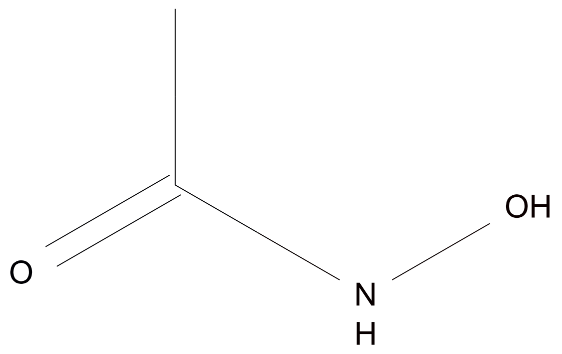

$C_2H_5NO_2$
Molecular Weight: 75.0666
ALogP: -0.82
Rotatable Bonds: 0
Acceptors: 2
Donors: 2

Model Prediction

Prediction: Moderate_Severe
Probability: 0.831
Enrichment: 1.21
Bayesian Score: 0.591
Mahalanobis Distance: 7.65
Mahalanobis Distance p-value: 0.974

Prediction: Positive if the Bayesian score is above the estimated best cutoff value from minimizing the false positive and false negative rate.
Probability: The esimated probability that the sample is in the positive category. This assumes that the Bayesian score follows a normal distribution and is different from the prediction using a cutoff.
Enrichment: An estimate of enrichment, that is, the increased likelihood (versus random) of this sample being in the category.
Bayesian Score: The standard Laplacian-modified Bayesian score.
Mahalanobis Distance: The Mahalanobis distance (MD) is the distance to the center of the training data. The larger the MD, the less trustworthy the prediction.
Mahalanobis Distance p-value: The p-value gives the fraction of training data with an MD greater than or equal to the one for the given sample, assuming normally distributed data. The smaller the p-value, the less trustworthy the prediciton. For highly non-normal X properties (e.g., fingerprints), the MD p-value is wildly inaccurate.

Structural Similar Compounds

Name	LACTIC ACID	ETHANOL; 2-AMINO-	1;2-ETHANEDIAMINE
Structure	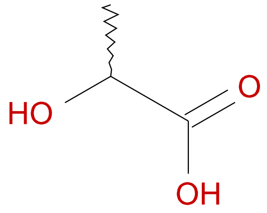	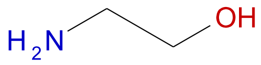	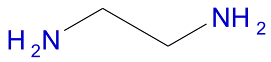
Actual Endpoint	Moderate_Severe	Moderate_Severe	Moderate_Severe
Predicted Endpoint	Moderate_Severe	Moderate_Severe	Moderate_Severe
Distance	0.410	0.481	0.505
Reference	AJOPAA 29;1363;46	AJOPAA 29;1363;46	AJOPAA 29;1363;46

Model Applicability

Unknown features are fingerprint features in the query molecule, but not found or appearing too infrequently in the training set.

1. All properties and OPS components are within expected ranges.
2. Unknown FCFP_2 feature: 1666606459: [*]C(=[*])NO
3. Unknown FCFP_2 feature: -548542844: [*]NO

Feature Contribution

Top features for positive contribution				
**Fingerprint**	**Bit/Smiles**	**Feature Structure**	**Score**	**Moderate_Severe in training set**
FCFP_10	7	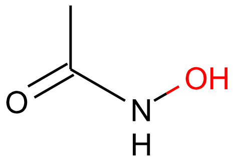 [*]O	0.219	117 out of 142

FCFP_10	3	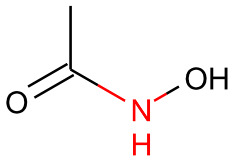 [*]N[*]	0.165	383 out of 491

Top Features for negative contribution

Fingerprint	Bit/Smiles	Feature Structure	Score	Moderate_Severe in training set
FCFP_10	1872154524	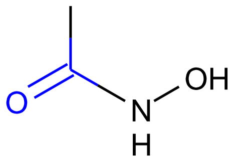 [*]C(=S)[*]	-0.0781	344 out of 563
FCFP_10	1	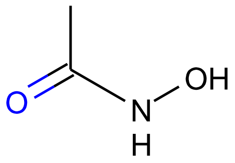 [*]=S	0	554 out of 872
FCFP_10	566058135	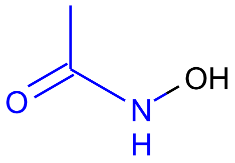 [*]NC(=O)C	0	16 out of 23

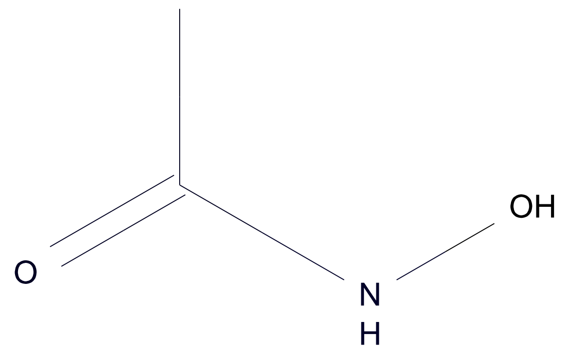

$C_2H_5NO_2$
Molecular Weight: 75.0666
ALogP: -0.82
Rotatable Bonds: 0
Acceptors: 2
Donors: 2

Model Prediction

Prediction: Moderate
Probability: 0.644
Enrichment: 1.04
Bayesian Score: -1.28
Mahalanobis Distance: 9.93
Mahalanobis Distance p-value: 0.0518

Prediction: Positive if the Bayesian score is above the estimated best cutoff value from minimizing the false positive and false negative rate.
Probability: The esimated probability that the sample is in the positive category. This assumes that the Bayesian score follows a normal distribution and is different from the prediction using a cutoff.
Enrichment: An estimate of enrichment, that is, the increased likelihood (versus random) of this sample being in the category.
Bayesian Score: The standard Laplacian-modified Bayesian score.
Mahalanobis Distance: The Mahalanobis distance (MD) is the distance to the center of the training data. The larger the MD, the less trustworthy the prediction.
Mahalanobis Distance p-value: The p-value gives the fraction of training data with an MD greater than or equal to the one for the given sample, assuming normally distributed data. The smaller the p-value, the less trustworthy the prediciton. For highly non-normal X properties (e.g., fingerprints), the MD p-value is wildly inaccurate.

Structural Similar Compounds

Name	LACTIC ACID	ETHANOL; 2-AMINO-	GLYCOLIC ACID
Structure	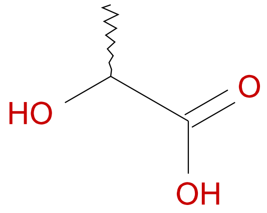	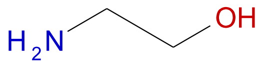	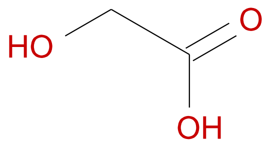
Actual Endpoint	Severe	Severe	Severe
Predicted Endpoint	Severe	Severe	Severe
Distance	0.462	0.499	0.529
Reference	AJOPAA 29;1363;46	AJOPAA 29;1363;46	AJOPAA 29;1363;46

Model Applicability

Unknown features are fingerprint features in the query molecule, but not found or appearing too infrequently in the training set.

1. All properties and OPS components are within expected ranges.

Feature Contribution

Top Features for negative contribution				
**Fingerprint**	**Bit/Smiles**	**Feature Structure**	**Score**	**Severe in training set**
SCFP_12	1256995004	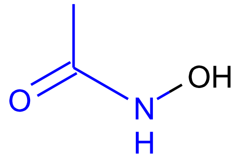 [*]NC(=O)C	-0.483	12 out of 33

SCFP_12	9	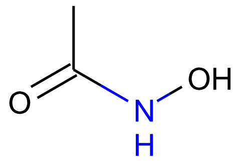 [*]N[*]	-0.315	29 out of 66
SCFP_12	13	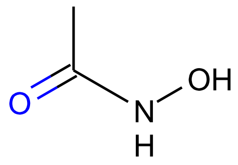 [*]=O	-0.105	185 out of 338

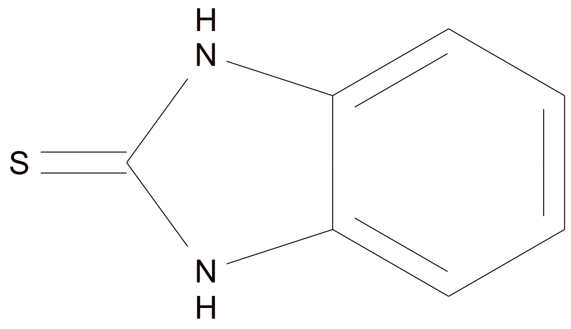

$C_7H_6N_2S$
Molecular Weight: 150.20094
ALogP: 2.536
Rotatable Bonds: 0
Acceptors: 1
Donors: 2

Structural Similar Compounds

Name	2-Benzimidazolethiol	ACETANILIDE; 2'-HYDROXY	PHENOL;M-AMINO-
Structure	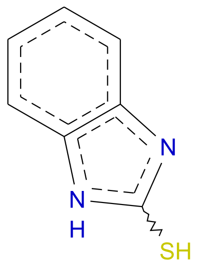	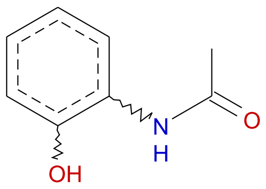	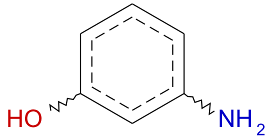
Actual Endpoint	Irritant	Irritant	Irritant
Predicted Endpoint	Irritant	Irritant	Irritant
Distance	0.567	0.577	0.592
Reference	Prehled Prumyslove Toxikologie; Organicke Latky; Marhold; J. pp 990;86	28ZPAK-;106;72	28ZPAK-;109;72

Model Prediction

Prediction: Irritant
Probability: 1
Enrichment: 1.18
Bayesian Score: 0.851
Mahalanobis Distance: 4.85
Mahalanobis Distance p-value: 1

Prediction: Positive if the Bayesian score is above the estimated best cutoff value from minimizing the false positive and false negative rate.
Probability: The esimated probability that the sample is in the positive category. This assumes that the Bayesian score follows a normal distribution and is different from the prediction using a cutoff.
Enrichment: An estimate of enrichment, that is, the increased likelihood (versus random) of this sample being in the category.
Bayesian Score: The standard Laplacian-modified Bayesian score.
Mahalanobis Distance: The Mahalanobis distance (MD) is the distance to the center of the training data. The larger the MD, the less trustworthy the prediction.
Mahalanobis Distance p-value: The p-value gives the fraction of training data with an MD greater than or equal to the one for the given sample, assuming normally distributed data. The smaller the p-value, the less trustworthy the prediciton. For highly non-normal X properties (e.g., fingerprints), the MD p-value is wildly inaccurate.

Model Applicability

Unknown features are fingerprint features in the query molecule, but not found or appearing too infrequently in the training set.

1. All properties and OPS components are within expected ranges.

Feature Contribution

Top features for positive contribution

Fingerprint	Bit/Smiles	Feature Structure	Score	Irritant in training set
FCFP_12	1499521844	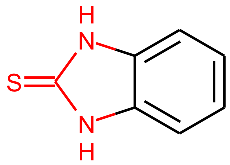 S=C1N[*]:[*]N1	0.156	3 out of 3

FCFP_12	3	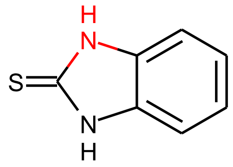 [*]N[*]	0.105	491 out of 547
FCFP_12	-1724769936	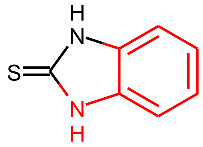 [*]1[*][c]2:[cH]:[cH]:[cH]:[cH]:[c]:2N1	0.102	18 out of 20

Top Features for negative contribution

Fingerprint	Bit/Smiles	Feature Structure	Score	Irritant in training set
FCFP_12	0	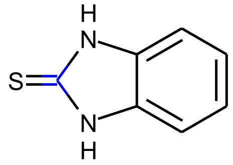 [*]C(=[*])[*]	0	1184 out of 1397
FCFP_12	1	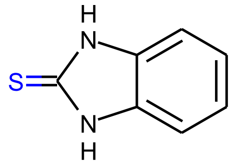 [*]=S	0	872 out of 1051
FCFP_12	1872154524	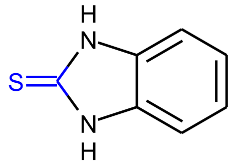 [*]C(=S)[*]	0	563 out of 690

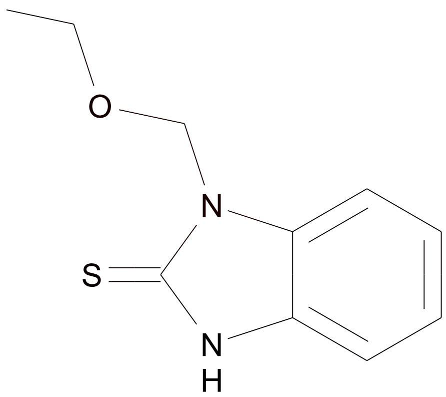

$C_{10}H_{12}N_2OS$
Molecular Weight: 208.28008
ALogP: 3.09
Rotatable Bonds: 3
Acceptors: 2
Donors: 1

Model Prediction

Prediction: Irritant
Probability: 1
Enrichment: 1.18
Bayesian Score: 0.967
Mahalanobis Distance: 6.85
Mahalanobis Distance p-value: 0.999

Prediction: Positive if the Bayesian score is above the estimated best cutoff value from minimizing the false positive and false negative rate.
Probability: The esimated probability that the sample is in the positive category. This assumes that the Bayesian score follows a normal distribution and is different from the prediction using a cutoff.
Enrichment: An estimate of enrichment, that is, the increased likelihood (versus random) of this sample being in the category.
Bayesian Score: The standard Laplacian-modified Bayesian score.
Mahalanobis Distance: The Mahalanobis distance (MD) is the distance to the center of the training data. The larger the MD, the less trustworthy the prediction.
Mahalanobis Distance p-value: The p-value gives the fraction of training data with an MD greater than or equal to the one for the given sample, assuming normally distributed data. The smaller the p-value, the less trustworthy the prediciton. For highly non-normal X properties (e.g., fingerprints), the MD p-value is wildly inaccurate.

Structural Similar Compounds

Name	DIPHENYLAMINE; 4-NITRO	ANILINE;P-PHENOXY-	BENZOTHIAZOLE; 5-AMINO-2-PHENYL-
Structure	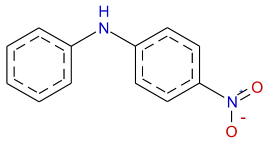	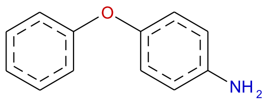	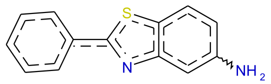
Actual Endpoint	Irritant	Irritant	Irritant
Predicted Endpoint	Irritant	Non-Irritant	Irritant
Distance	0.514	0.538	0.542
Reference	28ZPAK-;134;72	28ZPAK-;119;72	28ZPAK -;203;72

Model Applicability

Unknown features are fingerprint features in the query molecule, but not found or appearing too infrequently in the training set.

1. All properties and OPS components are within expected ranges.

Feature Contribution

Top features for positive contribution

Fingerprint	Bit/Smiles	Feature Structure	Score	Irritant in training set
FCFP_12	470041467	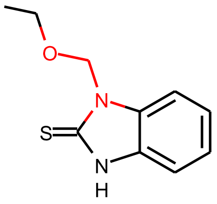 [*]OCN([*])[*]	0.187	8 out of 8

FCFP_12	1400413117	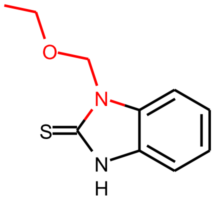 [*]N([*])COCC	0.175	5 out of 5
FCFP_12	-1986098826	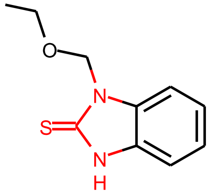 [*]N1[*]:[*]NC1=S	0.175	5 out of 5

Top Features for negative contribution

Fingerprint	Bit/Smiles	Feature Structure	Score	Irritant in training set
FCFP_12	-1155471474	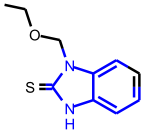 [*]N1C(=[*])N[c]2:[cH]:[cH]:[*]:[cH]:[c]1 :2	-0.344	2 out of 4
FCFP_12	-505031736	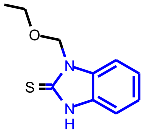 [*]N1C(=[*])N[c]2:[cH]:[cH]:[cH]:[cH]:[c] 1:2	-0.132	2 out of 3
FCFP_12	65948508	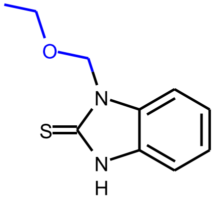 [*]COCC	-0.0658	68 out of 90

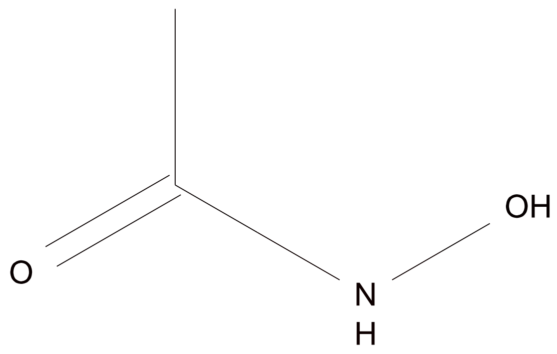

$C_2H_5NO_2$
Molecular Weight: 75.0666
ALogP: -0.82
Rotatable Bonds: 0
Acceptors: 2
Donors: 2

Model Prediction

Prediction: Irritant
Probability: 1
Enrichment: 1.18
Bayesian Score: 0.651
Mahalanobis Distance: 5.74
Mahalanobis Distance p-value: 1

Prediction: Positive if the Bayesian score is above the estimated best cutoff value from minimizing the false positive and false negative rate.
Probability: The esimated probability that the sample is in the positive category. This assumes that the Bayesian score follows a normal distribution and is different from the prediction using a cutoff.
Enrichment: An estimate of enrichment, that is, the increased likelihood (versus random) of this sample being in the category.
Bayesian Score: The standard Laplacian-modified Bayesian score.
Mahalanobis Distance: The Mahalanobis distance (MD) is the distance to the center of the training data. The larger the MD, the less trustworthy the prediction.
Mahalanobis Distance p-value: The p-value gives the fraction of training data with an MD greater than or equal to the one for the given sample, assuming normally distributed data. The smaller the p-value, the less trustworthy the prediciton. For highly non-normal X properties (e.g., fingerprints), the MD p-value is wildly inaccurate.

Structural Similar Compounds

Name	LACTIC ACID	ETHANOL; 2-AMINO-	1;2-ETHANEDIAMINE
Structure	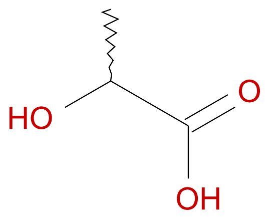	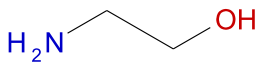	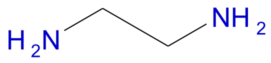
Actual Endpoint	Irritant	Irritant	Irritant
Predicted Endpoint	Irritant	Irritant	Irritant
Distance	0.404	0.476	0.496
Reference	AJOPAA 29;1363;46	AJOPAA 29;1363;46	AJOPAA 29;1363;46

Model Applicability

Unknown features are fingerprint features in the query molecule, but not found or appearing too infrequently in the training set.

1. All properties and OPS components are within expected ranges.
2. Unknown FCFP_2 feature: 1666606459: [*]C(=[*])NO
3. Unknown FCFP_2 feature: -548542844: [*]NO

Feature Contribution

Top features for positive contribution				
**Fingerprint**	**Bit/Smiles**	**Feature Structure**	**Score**	**Irritant in training set**
FCFP_12	566058135	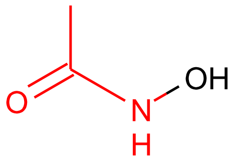 [*]NC(=O)C	0.163	23 out of 24

FCFP_12	7	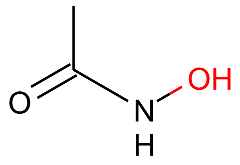 [*]O	0.119	142 out of 156
FCFP_12	3	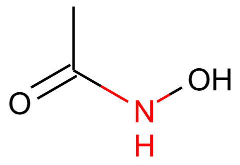 [*]N[*]	0.105	491 out of 547

Top Features for negative contribution

Fingerprint	Bit/Smiles	Feature Structure	Score	Irritant in training set
FCFP_12	1872154524	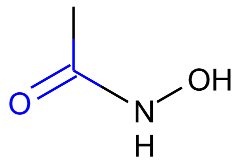 [*]C(=S)[*]	0	563 out of 690
FCFP_12	136597326	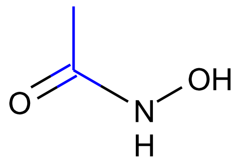 [*]CC	0	612 out of 753
FCFP_12	1	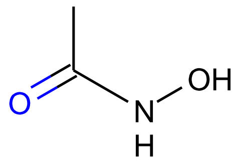 [*]=S	0	872 out of 1051

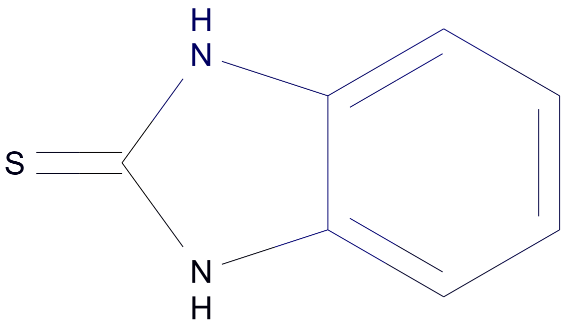

$C_7H_6N_2S$
Molecular Weight: 150.20094
ALogP: 2.536
Rotatable Bonds: 0
Acceptors: 1
Donors: 2

Model Prediction

Prediction: Non-Carcinogen
Probability: 0.253
Enrichment: 0.784
Bayesian Score: -2.61
Mahalanobis Distance: 5.94
Mahalanobis Distance p-value: 1

Prediction: Positive if the Bayesian score is above the estimated best cutoff value from minimizing the false positive and false negative rate.
Probability: The esimated probability that the sample is in the positive category. This assumes that the Bayesian score follows a normal distribution and is different from the prediction using a cutoff.
Enrichment: An estimate of enrichment, that is, the increased likelihood (versus random) of this sample being in the category.
Bayesian Score: The standard Laplacian-modified Bayesian score.
Mahalanobis Distance: The Mahalanobis distance (MD) is the distance to the center of the training data. The larger the MD, the less trustworthy the prediction.
Mahalanobis Distance p-value: The p-value gives the fraction of training data with an MD greater than or equal to the one for the given sample, assuming normally distributed data. The smaller the p-value, the less trustworthy the prediciton. For highly non-normal X properties (e.g., fingerprints), the MD p-value is wildly inaccurate.

Structural Similar Compounds

Name	Resorcinol	Hydroquinone	Thiabendazole
Structure	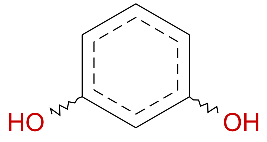	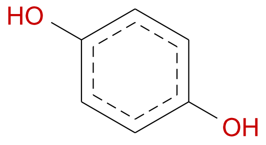	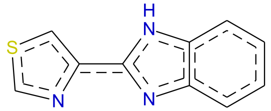
Actual Endpoint	Non-Carcinogen	Carcinogen	Non-Carcinogen
Predicted Endpoint	Non-Carcinogen	Carcinogen	Non-Carcinogen
Distance	0.607	0.618	0.669
Reference	US FDA (Centre for Drug Eval.& Res./Off. Testing & Res.) Sept. 1997	US FDA (Centre for Drug Eval.& Res./Off. Testing & Res.) Sept. 1997	US FDA (Centre for Drug Eval.& Res./Off. Testing & Res.) Sept. 1997

Model Applicability

Unknown features are fingerprint features in the query molecule, but not found or appearing too infrequently in the training set.

1. All properties and OPS components are within expected ranges.
2. Unknown ECFP_2 feature: -829433624: S=C1N[*]:[*]N1
3. Unknown ECFP_2 feature: 1985868180: [*]C(=S)[*]

Feature Contribution

Top features for positive contribution

Fingerprint	Bit/Smiles	Feature Structure	Score	Carcinogen in training set
ECFP_12	-1699286547	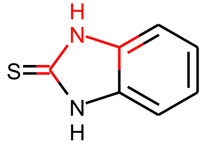 [*]=C1[*][*]:[c](:[*])N1	0.0535	5 out of 15

Top Features for negative contribution

Fingerprint	Bit/Smiles	Feature Structure	Score	Carcinogen in training set
ECFP_12	1335833675	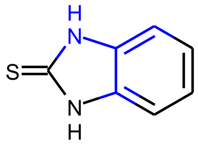 [*]:[cH]:[c]1N[*][*][c]:1:[*]	-1.25	0 out of 8
ECFP_12	1640720160	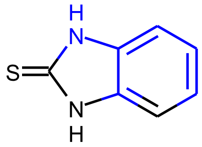 [*]1[*][c]2:[*]:[cH]:[cH]:[cH]:[c]:2N1	-0.485	0 out of 2
ECFP_12	-845108448	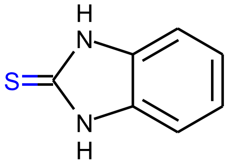 [*]=S	-0.485	0 out of 2

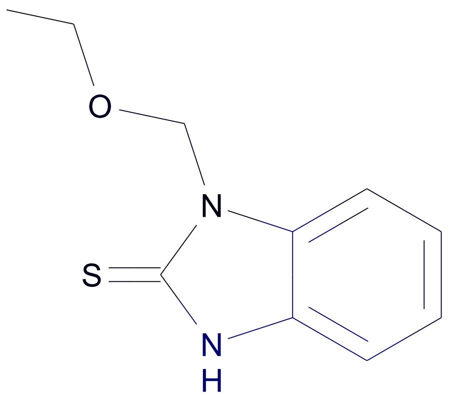

$C_{10}H_{12}N_2OS$
Molecular Weight: 208.28008
ALogP: 3.09
Rotatable Bonds: 3
Acceptors: 2
Donors: 1

Model Prediction

Prediction: Non-Carcinogen
Probability: 0.239
Enrichment: 0.742
Bayesian Score: -3.56
Mahalanobis Distance: 8.42
Mahalanobis Distance p-value: 0.953

Prediction: Positive if the Bayesian score is above the estimated best cutoff value from minimizing the false positive and false negative rate.
Probability: The esimated probability that the sample is in the positive category. This assumes that the Bayesian score follows a normal distribution and is different from the prediction using a cutoff.
Enrichment: An estimate of enrichment, that is, the increased likelihood (versus random) of this sample being in the category.
Bayesian Score: The standard Laplacian-modified Bayesian score.
Mahalanobis Distance: The Mahalanobis distance (MD) is the distance to the center of the training data. The larger the MD, the less trustworthy the prediction.
Mahalanobis Distance p-value: The p-value gives the fraction of training data with an MD greater than or equal to the one for the given sample, assuming normally distributed data. The smaller the p-value, the less trustworthy the prediciton. For highly non-normal X properties (e.g., fingerprints), the MD p-value is wildly inaccurate.

Structural Similar Compounds

Name	Ketorolac	Naproxen	Mexiletine
Structure	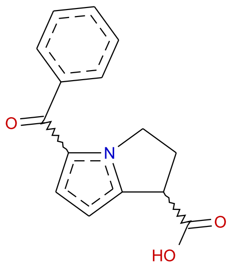	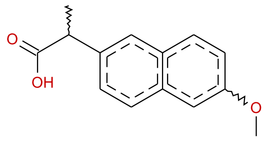	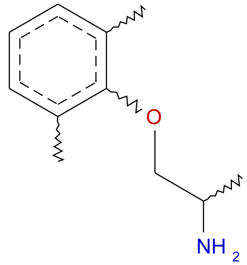
Actual Endpoint	Non-Carcinogen	Non-Carcinogen	Non-Carcinogen
Predicted Endpoint	Non-Carcinogen	Non-Carcinogen	Non-Carcinogen
Distance	0.601	0.606	0.607
Reference	US FDA (Centre for Drug Eval.& Res./Off. Testing & Res.) Sept. 1997	US FDA (Centre for Drug Eval.& Res./Off. Testing & Res.) Sept. 1997	US FDA (Centre for Drug Eval.& Res./Off. Testing & Res.) Sept. 1997

Model Applicability

Unknown features are fingerprint features in the query molecule, but not found or appearing too infrequently in the training set.

1. All properties and OPS components are within expected ranges.
2. Unknown ECFP_2 feature: 434461827: [*]N1[*]:[*]NC1=S
3. Unknown ECFP_2 feature: 1985868180: [*]C(=S)[*]

Feature Contribution

Top features for positive contribution

Fingerprint	Bit/Smiles	Feature Structure	Score	Carcinogen in training set
ECFP_12	-669219631	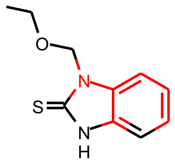 [*]N1[*][*][c]2:[*]:[cH]:[cH]:[cH]:[c]1:2	0.119	4 out of 11

ECFP_12	-749801696	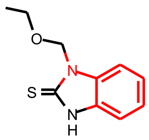 [*]N1[*][*][c]2:[cH]:[cH]:[cH]:[cH]:[c]1:2	0.119	4 out of 11
ECFP_12	734603939	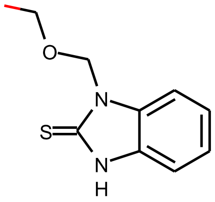 [*]C	0.0966	92 out of 267
**Top Features for negative contribution**				
**Fingerprint**	**Bit/Smiles**	**Feature Structure**	**Score**	**Carcinogen in training set**
ECFP_12	1335833675	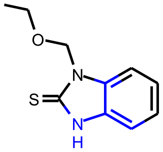 [*]:[cH]:[c]1N[*][*][c]:1:[*]	-1.25	0 out of 8
ECFP_12	1640720160	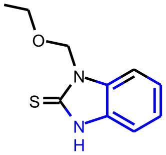 [*]1[*][c]2:[*]:[cH]:[cH]:[cH]:[c]:2N1	-0.485	0 out of 2
ECFP_12	-845108448	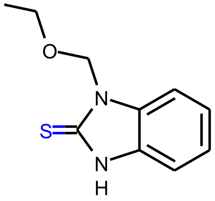 [*]=S	-0.485	0 out of 2

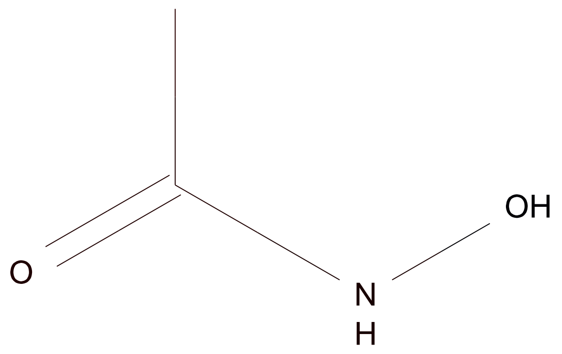

$C_2H_5NO_2$
Molecular Weight: 75.0666
ALogP: -0.82
Rotatable Bonds: 0
Acceptors: 2
Donors: 2

Model Prediction

Prediction: Carcinogen
Probability: 0.333
Enrichment: 1.04
Bayesian Score: 1.59
Mahalanobis Distance: 7.86
Mahalanobis Distance p-value: 0.993

Prediction: Positive if the Bayesian score is above the estimated best cutoff value from minimizing the false positive and false negative rate.
Probability: The esimated probability that the sample is in the positive category. This assumes that the Bayesian score follows a normal distribution and is different from the prediction using a cutoff.
Enrichment: An estimate of enrichment, that is, the increased likelihood (versus random) of this sample being in the category.
Bayesian Score: The standard Laplacian-modified Bayesian score.
Mahalanobis Distance: The Mahalanobis distance (MD) is the distance to the center of the training data. The larger the MD, the less trustworthy the prediction.
Mahalanobis Distance p-value: The p-value gives the fraction of training data with an MD greater than or equal to the one for the given sample, assuming normally distributed data. The smaller the p-value, the less trustworthy the prediciton. For highly non-normal X properties (e.g., fingerprints), the MD p-value is wildly inaccurate.

Structural Similar Compounds

Name	Acetamide	Isoniazid	Pyrazinamide
Structure	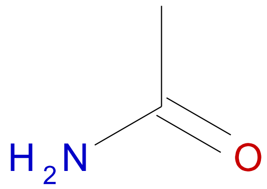	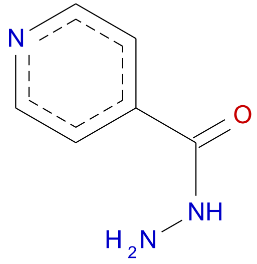	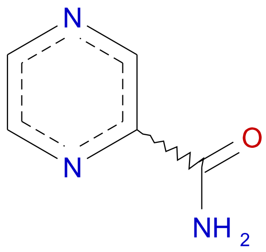
Actual Endpoint	Carcinogen	Carcinogen	Non-Carcinogen
Predicted Endpoint	Carcinogen	Carcinogen	Non-Carcinogen
Distance	0.495	0.635	0.661
Reference	US FDA (Centre for Drug Eval.& Res./Off. Testing & Res.) Sept. 1997	US FDA (Centre for Drug Eval.& Res./Off. Testing & Res.) Sept. 1997	US FDA (Centre for Drug Eval.& Res./Off. Testing & Res.) Sept. 1997

Model Applicability

Unknown features are fingerprint features in the query molecule, but not found or appearing too infrequently in the training set.

1. All properties and OPS components are within expected ranges.
2. Unknown ECFP_2 feature: 379232550: [*]C(=[*])NO
3. Unknown ECFP_2 feature: 2023553742: [*]NO

Feature Contribution

Top features for positive contribution

Fingerprint	Bit/Smiles	Feature Structure	Score	Carcinogen in training set
ECFP_12	-474544785	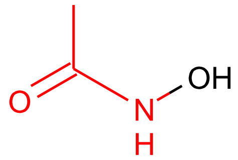 [*]NC(=O)C	0.575	3 out of 4

ECFP_12	866218936	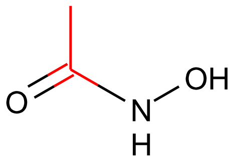 [*]C(=[*])C	0.196	12 out of 31
ECFP_12	-1074141656	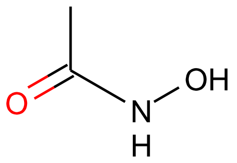 [*]=O	0.103	86 out of 248

Top Features for negative contribution

Fingerprint	Bit/Smiles	Feature Structure	Score	Carcinogen in training set
ECFP_12	-1884411803	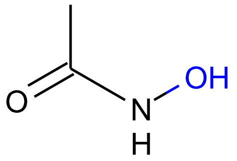 [*]O	-0.106	55 out of 196
ECFP_12	-1897341097	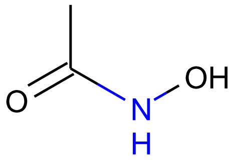 [*]N[*]	-0.096	30 out of 106
ECFP_12	2099970318	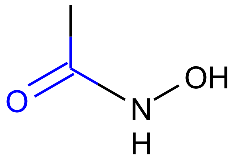 [*]C(=O)[*]	-0.0766	50 out of 173

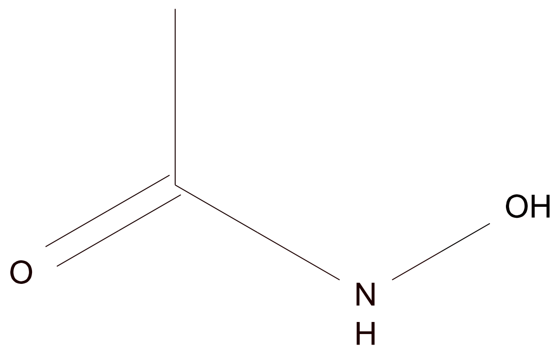

$C_2H_5NO_2$
Molecular Weight: 75.0666
ALogP: -0.82
Rotatable Bonds: 0
Acceptors: 2
Donors: 2

Model Prediction

Prediction: Multiple-Carcinogen
Probability: 0.577
Enrichment: 1.54
Bayesian Score: 2.72
Mahalanobis Distance: 9.32
Mahalanobis Distance p-value: 0.157

Prediction: Positive if the Bayesian score is above the estimated best cutoff value from minimizing the false positive and false negative rate.
Probability: The esimated probability that the sample is in the positive category. This assumes that the Bayesian score follows a normal distribution and is different from the prediction using a cutoff.
Enrichment: An estimate of enrichment, that is, the increased likelihood (versus random) of this sample being in the category.
Bayesian Score: The standard Laplacian-modified Bayesian score.
Mahalanobis Distance: The Mahalanobis distance (MD) is the distance to the center of the training data. The larger the MD, the less trustworthy the prediction.
Mahalanobis Distance p-value: The p-value gives the fraction of training data with an MD greater than or equal to the one for the given sample, assuming normally distributed data. The smaller the p-value, the less trustworthy the prediciton. For highly non-normal X properties (e.g., fingerprints), the MD p-value is wildly inaccurate.

Structural Similar Compounds

Name	Acetamide	Isoniazid	Acetaminophen
Structure	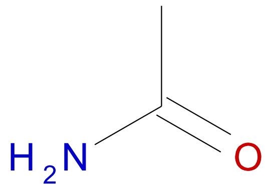	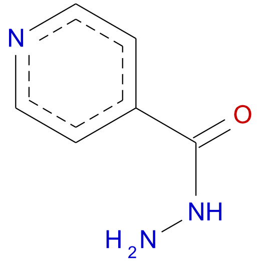	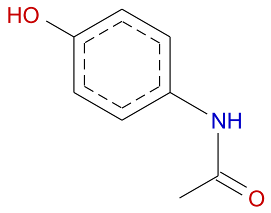
Actual Endpoint	Single-Carcinogen	Single-Carcinogen	Multiple-Carcinogen
Predicted Endpoint	Multiple-Carcinogen	Single-Carcinogen	Multiple-Carcinogen
Distance	0.419	0.621	0.691
Reference	US FDA (Centre for Drug Eval.& Res./Off. Testing & Res.) Sept. 1997	US FDA (Centre for Drug Eval.& Res./Off. Testing & Res.) Sept. 1997	US FDA (Centre for Drug Eval.& Res./Off. Testing & Res.) Sept. 1997

Model Applicability

Unknown features are fingerprint features in the query molecule, but not found or appearing too infrequently in the training set.

1. All properties and OPS components are within expected ranges.

Feature Contribution

Top features for positive contribution

Fingerprint	Bit/Smiles	Feature Structure	Score	Multiple-Carcinogen in training set
SCFP_4	1256995004	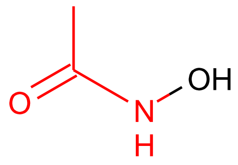 [*]NC(=O)C	0.327	10 out of 22

SCFP_4	9	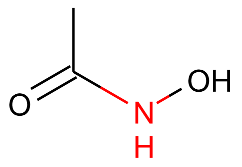 [*]N[*]	0.245	18 out of 44
SCFP_4	0	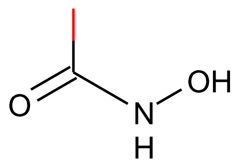 [*]C	0.155	41 out of 111
**Top Features for negative contribution**				
**Fingerprint**	**Bit/Smiles**	**Feature Structure**	**Score**	**Multiple-Carcinogen in training set**
SCFP_4	136627117	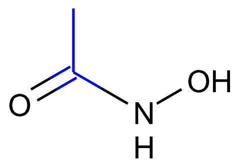 [*]C(=[*])C	0	6 out of 18

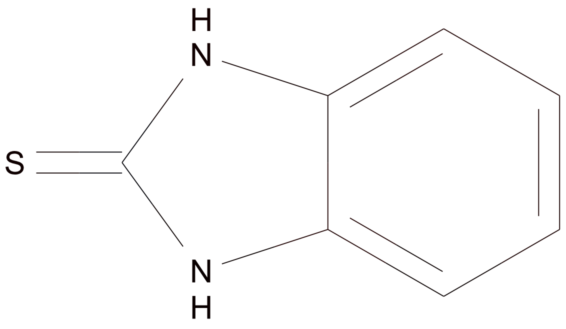

$C_7H_6N_2S$
Molecular Weight: 150.20094
ALogP: 2.536
Rotatable Bonds: 0
Acceptors: 1
Donors: 2

Model Prediction

Prediction: Carcinogen
Probability: 0.404
Enrichment: 1.21
Bayesian Score: 1.56
Mahalanobis Distance: 9.44
Mahalanobis Distance p-value: 0.862

Prediction: Positive if the Bayesian score is above the estimated best cutoff value from minimizing the false positive and false negative rate.
Probability: The esimated probability that the sample is in the positive category. This assumes that the Bayesian score follows a normal distribution and is different from the prediction using a cutoff.
Enrichment: An estimate of enrichment, that is, the increased likelihood (versus random) of this sample being in the category.
Bayesian Score: The standard Laplacian-modified Bayesian score.
Mahalanobis Distance: The Mahalanobis distance (MD) is the distance to the center of the training data. The larger the MD, the less trustworthy the prediction.
Mahalanobis Distance p-value: The p-value gives the fraction of training data with an MD greater than or equal to the one for the given sample, assuming normally distributed data. The smaller the p-value, the less trustworthy the prediciton. For highly non-normal X properties (e.g., fingerprints), the MD p-value is wildly inaccurate.

Structural Similar Compounds

Name	Resorcinol	Hydroquinone	Propylthiouracil
Structure	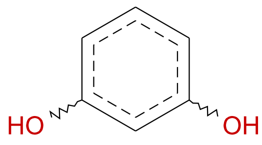	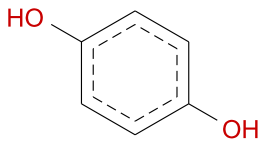	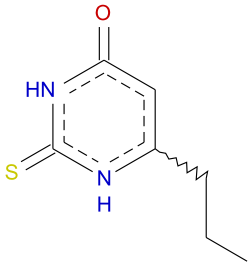
Actual Endpoint	Non-Carcinogen	Carcinogen	Carcinogen
Predicted Endpoint	Carcinogen	Carcinogen	Carcinogen
Distance	0.615	0.632	0.635
Reference	US FDA (Centre for Drug Eval.& Res./Off. Testing & Res.) Sept. 1997	US FDA (Centre for Drug Eval.& Res./Off. Testing & Res.) Sept. 1997	US FDA (Centre for Drug Eval.& Res./Off. Testing & Res.) Sept. 1997

Model Applicability

Unknown features are fingerprint features in the query molecule, but not found or appearing too infrequently in the training set.

1. All properties and OPS components are within expected ranges.

Feature Contribution

Top features for positive contribution

Fingerprint	Bit/Smiles	Feature Structure	Score	Carcinogen in training set
SCFP_6	-1380909229	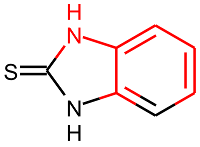 [*]1[*][c]2:[*]:[cH]:[cH]:[cH]:[c]:2N1	0.287	17 out of 39

SCFP_6	10	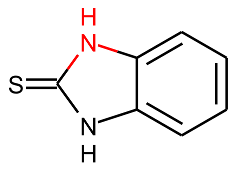 [*]N[*]	0.21	39 out of 98
SCFP_6	1654335448	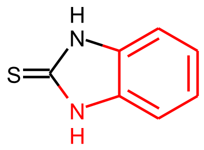 [*]1[*][c]2:[cH]:[cH] :[cH]:[cH]:[c]:2N1	0.186	6 out of 15

Top Features for negative contribution

**Fingerprint**	**Bit/Smiles**	**Feature Structure**	**Score**	**Carcinogen in training set**
SCFP_6	1760928239	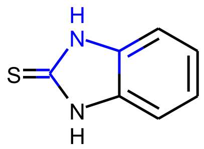 [*]=C1[*][*]:[c](:[*])N1	-0.278	0 out of 1
SCFP_6	-496409612	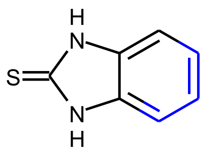 [*]:[cH]:[cH]:[cH]:[*]	0	86 out of 276
SCFP_6	1	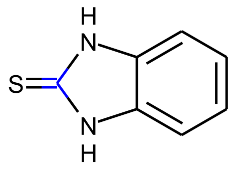 [*]C(=[*])[*]	0	90 out of 272

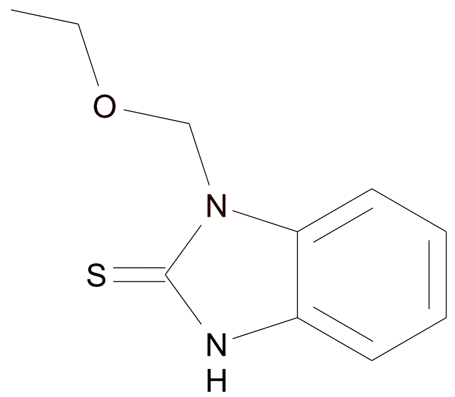

$C_{10}H_{12}N_2OS$
Molecular Weight: 208.28008
ALogP: 3.09
Rotatable Bonds: 3
Acceptors: 2
Donors: 1

Model Prediction

Prediction: Carcinogen
Probability: 0.401
Enrichment: 1.2
Bayesian Score: 1.48
Mahalanobis Distance: 10.6
Mahalanobis Distance p-value: 0.355

Prediction: Positive if the Bayesian score is above the estimated best cutoff value from minimizing the false positive and false negative rate.
Probability: The esimated probability that the sample is in the positive category. This assumes that the Bayesian score follows a normal distribution and is different from the prediction using a cutoff.
Enrichment: An estimate of enrichment, that is, the increased likelihood (versus random) of this sample being in the category.
Bayesian Score: The standard Laplacian-modified Bayesian score.
Mahalanobis Distance: The Mahalanobis distance (MD) is the distance to the center of the training data. The larger the MD, the less trustworthy the prediction.
Mahalanobis Distance p-value: The p-value gives the fraction of training data with an MD greater than or equal to the one for the given sample, assuming normally distributed data. The smaller the p-value, the less trustworthy the prediciton. For highly non-normal X properties (e.g., fingerprints), the MD p-value is wildly inaccurate.

Structural Similar Compounds

Name	Ketorolac	Naproxen	Mexiletine
Structure	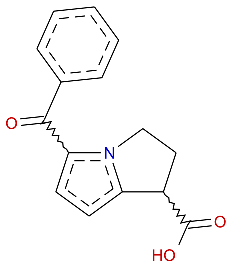	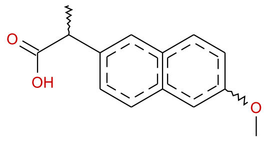	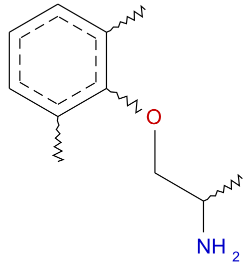
Actual Endpoint	Non-Carcinogen	Non-Carcinogen	Non-Carcinogen
Predicted Endpoint	Non-Carcinogen	Non-Carcinogen	Non-Carcinogen
Distance	0.585	0.588	0.592
Reference	US FDA (Centre for Drug Eval.& Res./Off. Testing & Res.) Sept. 1997	US FDA (Centre for Drug Eval.& Res./Off. Testing & Res.) Sept. 1997	US FDA (Centre for Drug Eval.& Res./Off. Testing & Res.) Sept. 1997

Model Applicability

Unknown features are fingerprint features in the query molecule, but not found or appearing too infrequently in the training set.

1. All properties and OPS components are within expected ranges.

Feature Contribution

Top features for positive contribution				
**Fingerprint**	**Bit/Smiles**	**Feature Structure**	**Score**	**Carcinogen in training set**
SCFP_6	-711864945	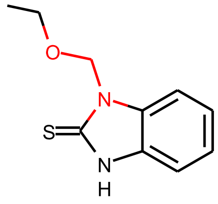 [*]OCN([*])[*]	0.415	1 out of 1

SCFP_6	-1380909229	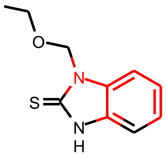 [*]1[*][c]2:[*]:[cH]:[cH]:[cH]:[c]:2N1	0.287	17 out of 39
SCFP_6	10	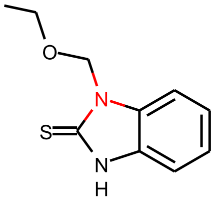 [*]N[*]	0.21	39 out of 98
**Top Features for negative contribution**				
**Fingerprint**	**Bit/Smiles**	**Feature Structure**	**Score**	**Carcinogen in training set**
SCFP_6	1760928239	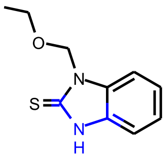 [*]=C1[*][*]:[c](:[*])N1	-0.278	0 out of 1
SCFP_6	-110814626	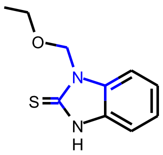 [*]CN1C(=[*])[*][*]:[c]1:[*]	-0.264	1 out of 5
SCFP_6	-711686199	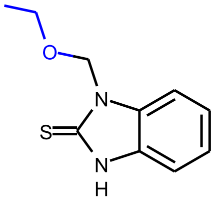 [*]OCC	0	26 out of 81

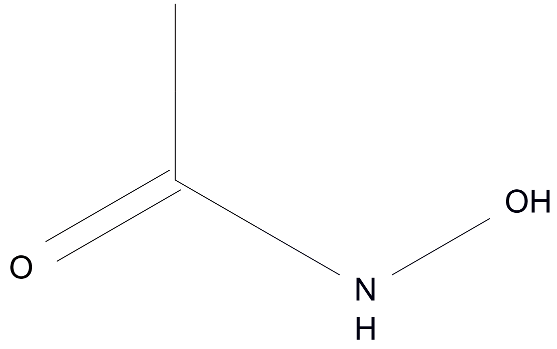

$C_2H_5NO_2$
Molecular Weight: 75.0666
ALogP: -0.82
Rotatable Bonds: 0
Acceptors: 2
Donors: 2

Model Prediction

Prediction: Carcinogen
Probability: 0.366
Enrichment: 1.09
Bayesian Score: 0.325
Mahalanobis Distance: 9.38
Mahalanobis Distance p-value: 0.879

Prediction: Positive if the Bayesian score is above the estimated best cutoff value from minimizing the false positive and false negative rate.
Probability: The esimated probability that the sample is in the positive category. This assumes that the Bayesian score follows a normal distribution and is different from the prediction using a cutoff.
Enrichment: An estimate of enrichment, that is, the increased likelihood (versus random) of this sample being in the category.
Bayesian Score: The standard Laplacian-modified Bayesian score.
Mahalanobis Distance: The Mahalanobis distance (MD) is the distance to the center of the training data. The larger the MD, the less trustworthy the prediction.
Mahalanobis Distance p-value: The p-value gives the fraction of training data with an MD greater than or equal to the one for the given sample, assuming normally distributed data. The smaller the p-value, the less trustworthy the prediciton. For highly non-normal X properties (e.g., fingerprints), the MD p-value is wildly inaccurate.

Structural Similar Compounds

Name	Acetamide	Barbituric acid	Fluorouracil; 5-
Structure	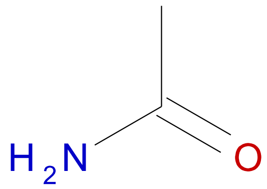	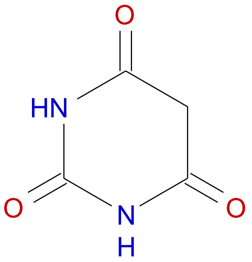	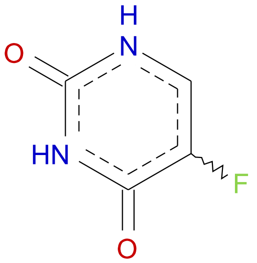
Actual Endpoint	Carcinogen	Non-Carcinogen	Non-Carcinogen
Predicted Endpoint	Carcinogen	Non-Carcinogen	Non-Carcinogen
Distance	0.379	0.517	0.561
Reference	US FDA (Centre for Drug Eval.& Res./Off. Testing & Res.) Sept. 1997	US FDA (Centre for Drug Eval.& Res./Off. Testing & Res.) Sept. 1997	US FDA (Centre for Drug Eval.& Res./Off. Testing & Res.) Sept. 1997

Model Applicability

Unknown features are fingerprint features in the query molecule, but not found or appearing too infrequently in the training set.

1. All properties and OPS components are within expected ranges.

Feature Contribution

Top features for positive contribution

Fingerprint	Bit/Smiles	Feature Structure	Score	Carcinogen in training set
SCFP_6	136627117	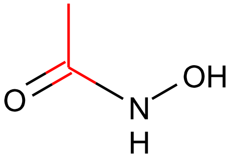 [*]C(=[*])C	0.167	18 out of 47

SCFP_6	13	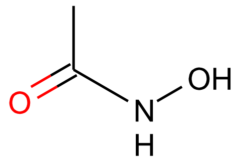 [*]=O	0.0717	90 out of 261
**Top Features for negative contribution**				
**Fingerprint**	**Bit/Smiles**	**Feature Structure**	**Score**	**Carcinogen in training set**
SCFP_6	450088063	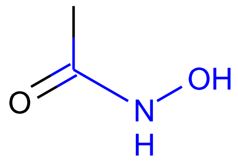 [*]C(=[*])NO	-0.278	0 out of 1
SCFP_6	9	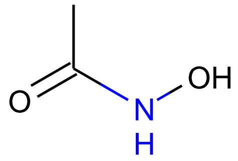 [*]N[*]	-0.0553	40 out of 132
SCFP_6	12	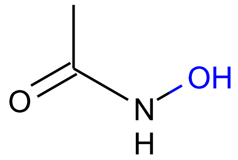 [*]O[*]	0	79 out of 255

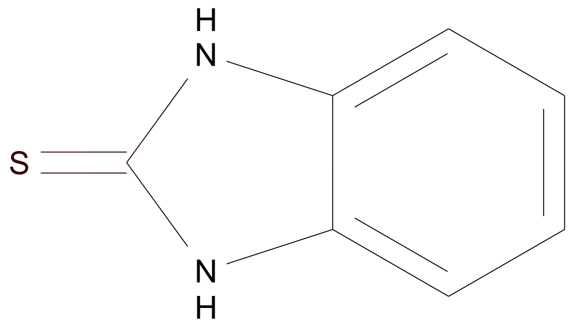

$C_7H_6N_2S$
Molecular Weight: 150.20094
ALogP: 2.536
Rotatable Bonds: 0
Acceptors: 1
Donors: 2

Structural Similar Compounds

Name	Hydroquinone	Propylthiouracil	Proflavine
Structure	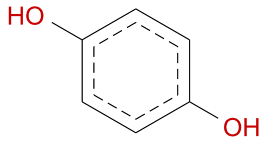	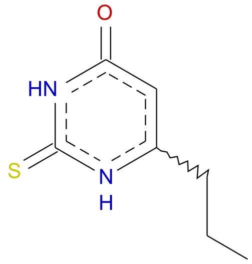	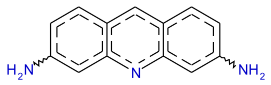
Actual Endpoint	Multiple-Carcinogen	Multiple-Carcinogen	Single-Carcinogen
Predicted Endpoint	Multiple-Carcinogen	Multiple-Carcinogen	Single-Carcinogen
Distance	0.643	0.651	0.698
Reference	US FDA (Centre for Drug Eval.& Res./Off. Testing & Res.) Sept. 1997	US FDA (Centre for Drug Eval.& Res./Off. Testing & Res.) Sept. 1997	US FDA (Centre for Drug Eval.& Res./Off. Testing & Res.) Sept. 1997

Model Prediction

Prediction: Multiple-Carcinogen
Probability: 0.569
Enrichment: 1.37
Bayesian Score: 1.82
Mahalanobis Distance: 11.2
Mahalanobis Distance p-value: 0.0167

Prediction: Positive if the Bayesian score is above the estimated best cutoff value from minimizing the false positive and false negative rate.
Probability: The esimated probability that the sample is in the positive category. This assumes that the Bayesian score follows a normal distribution and is different from the prediction using a cutoff.
Enrichment: An estimate of enrichment, that is, the increased likelihood (versus random) of this sample being in the category.
Bayesian Score: The standard Laplacian-modified Bayesian score.
Mahalanobis Distance: The Mahalanobis distance (MD) is the distance to the center of the training data. The larger the MD, the less trustworthy the prediction.
Mahalanobis Distance p-value: The p-value gives the fraction of training data with an MD greater than or equal to the one for the given sample, assuming normally distributed data. The smaller the p-value, the less trustworthy the prediciton. For highly non-normal X properties (e.g., fingerprints), the MD p-value is wildly inaccurate.

Model Applicability

Unknown features are fingerprint features in the query molecule, but not found or appearing too infrequently in the training set.

1. All properties and OPS components are within expected ranges.

Feature Contribution

Top features for positive contribution

Fingerprint	Bit/Smiles	Feature Structure	Score	Multiple-Carcinogen in training set
SCFP_8	1435188938	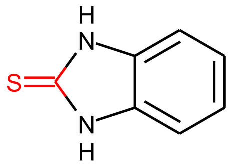 [*]C(=S)[*]	0.553	2 out of 2

SCFP_8	18	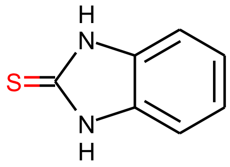 [*]=S	0.553	2 out of 2
SCFP_8	1654335448	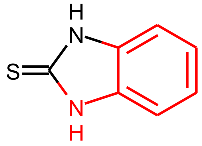 [*]1[*][c]2:[cH]:[cH]:[cH]:[cH]:[c]:2N1	0.23	3 out of 6
**Top Features for negative contribution**				
**Fingerprint**	**Bit/Smiles**	**Feature Structure**	**Score**	**Multiple-Carcinogen in training set**
SCFP_8	-1379591900	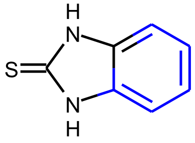 [*][c]1:[*]:[cH]:[cH]:[cH]:[cH]:1	-0.275	13 out of 48
SCFP_8	1334669481	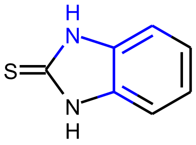 [*]:[cH]:[c]1N[*][*][c]:1:[*]	0	7 out of 19
SCFP_8	-496409612	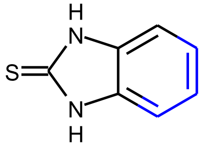 [*]:[cH]:[cH]:[cH]:[*]	0	31 out of 86

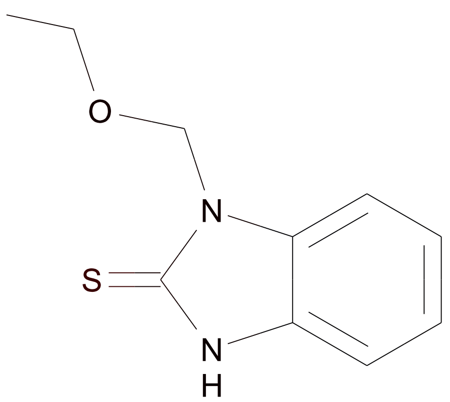

$C_{10}H_{12}N_2OS$
Molecular Weight: 208.28008
ALogP: 3.09
Rotatable Bonds: 3
Acceptors: 2
Donors: 1

Model Prediction

Prediction: Multiple-Carcinogen
Probability: 0.569
Enrichment: 1.37
Bayesian Score: 1.89
Mahalanobis Distance: 12.3
Mahalanobis Distance p-value: 0.00268

Prediction: Positive if the Bayesian score is above the estimated best cutoff value from minimizing the false positive and false negative rate.
Probability: The esimated probability that the sample is in the positive category. This assumes that the Bayesian score follows a normal distribution and is different from the prediction using a cutoff.
Enrichment: An estimate of enrichment, that is, the increased likelihood (versus random) of this sample being in the category.
Bayesian Score: The standard Laplacian-modified Bayesian score.
Mahalanobis Distance: The Mahalanobis distance (MD) is the distance to the center of the training data. The larger the MD, the less trustworthy the prediction.
Mahalanobis Distance p-value: The p-value gives the fraction of training data with an MD greater than or equal to the one for the given sample, assuming normally distributed data. The smaller the p-value, the less trustworthy the prediciton. For highly non-normal X properties (e.g., fingerprints), the MD p-value is wildly inaccurate.

Structural Similar Compounds

Name	Flutamide	Valproic acid	Phenacetin
Structure	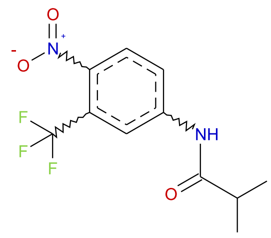	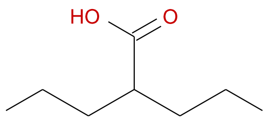	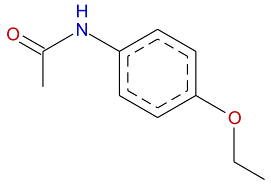
Actual Endpoint	Multiple-Carcinogen	Single-Carcinogen	Multiple-Carcinogen
Predicted Endpoint	Multiple-Carcinogen	Single-Carcinogen	Multiple-Carcinogen
Distance	0.618	0.654	0.659
Reference	US FDA (Centre for Drug Eval.& Res./Off. Testing & Res.) Sept. 1997	US FDA (Centre for Drug Eval.& Res./Off. Testing & Res.) Sept. 1997	US FDA (Centre for Drug Eval.& Res./Off. Testing & Res.) Sept. 1997

Model Applicability

Unknown features are fingerprint features in the query molecule, but not found or appearing too infrequently in the training set.

1. All properties and OPS components are within expected ranges.

Feature Contribution

Top features for positive contribution				
**Fingerprint**	**Bit/Smiles**	**Feature Structure**	**Score**	**Multiple-Carcinogen in training set**
SCFP_8	18	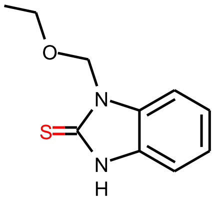 [*]=S	0.553	2 out of 2

SCFP_8	1435188938	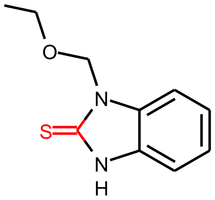<br>[*]C(=S)[*]	0.553	2 out of 2
SCFP_8	-711864945	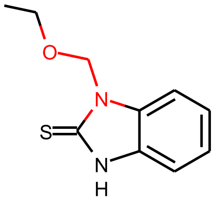<br>[*]OCN([*])[*]	0.383	1 out of 1
**Top Features for negative contribution**				
**Fingerprint**	**Bit/Smiles**	**Feature Structure**	**Score**	**Multiple-Carcinogen in training set**
SCFP_8	-110814626	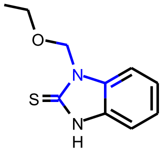<br>[*]CN1C(=[*])[*][*]:[c]1:[*]	-0.31	0 out of 1
SCFP_8	-1379591900	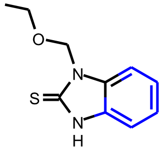<br>[*][c]1:[*]:[cH]:[cH]:[cH]:[cH]:1	-0.275	13 out of 48
SCFP_8	276193969	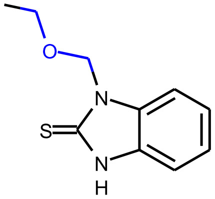<br>[*]COC[*]	-0.072	5 out of 15

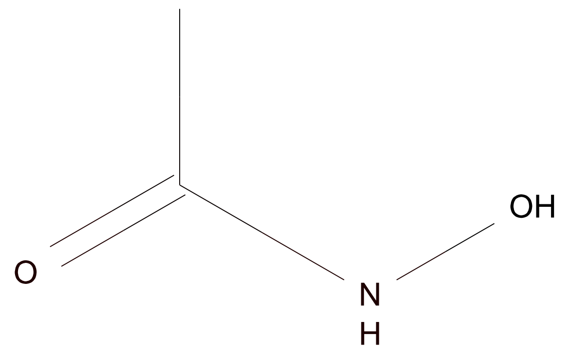

$C_2H_5NO_2$
Molecular Weight: 75.0666
ALogP: -0.82
Rotatable Bonds: 0
Acceptors: 2
Donors: 2

Model Prediction

Prediction: Multiple-Carcinogen
Probability: 0.567
Enrichment: 1.37
Bayesian Score: 2.3
Mahalanobis Distance: 10.4
Mahalanobis Distance p-value: 0.0608

Prediction: Positive if the Bayesian score is above the estimated best cutoff value from minimizing the false positive and false negative rate.
Probability: The esimated probability that the sample is in the positive category. This assumes that the Bayesian score follows a normal distribution and is different from the prediction using a cutoff.
Enrichment: An estimate of enrichment, that is, the increased likelihood (versus random) of this sample being in the category.
Bayesian Score: The standard Laplacian-modified Bayesian score.
Mahalanobis Distance: The Mahalanobis distance (MD) is the distance to the center of the training data. The larger the MD, the less trustworthy the prediction.
Mahalanobis Distance p-value: The p-value gives the fraction of training data with an MD greater than or equal to the one for the given sample, assuming normally distributed data. The smaller the p-value, the less trustworthy the prediciton. For highly non-normal X properties (e.g., fingerprints), the MD p-value is wildly inaccurate.

Structural Similar Compounds

Name	Acetamide	Thiouracil	Acetaminophen
Structure	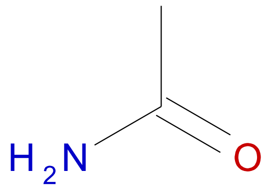	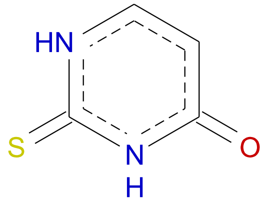	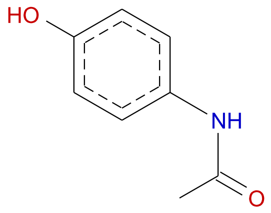
Actual Endpoint	Single-Carcinogen	Multiple-Carcinogen	Multiple-Carcinogen
Predicted Endpoint	Multiple-Carcinogen	Multiple-Carcinogen	Multiple-Carcinogen
Distance	0.419	0.575	0.739
Reference	US FDA (Centre for Drug Eval.& Res./Off. Testing & Res.) Sept. 1997	US FDA (Centre for Drug Eval.& Res./Off. Testing & Res.) Sept. 1997	US FDA (Centre for Drug Eval.& Res./Off. Testing & Res.) Sept. 1997

Model Applicability

Unknown features are fingerprint features in the query molecule, but not found or appearing too infrequently in the training set.

1. All properties and OPS components are within expected ranges.

Feature Contribution

Top features for positive contribution

Fingerprint	Bit/Smiles	Feature Structure	Score	Multiple-Carcinogen in training set
SCFP_8	1256995004	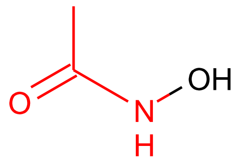 [*]NC(=O)C	0.331	10 out of 19

SCFP_8	9	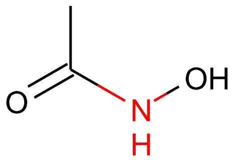 [*]N[*]	0.253	19 out of 40
SCFP_8	13	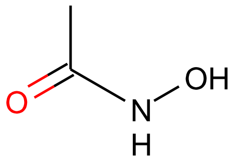 [*]=O	0.172	39 out of 90
**Top Features for negative contribution**				
**Fingerprint**	**Bit/Smiles**	**Feature Structure**	**Score**	**Multiple-Carcinogen in training set**
SCFP_8	136627117	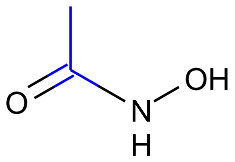 [*]C(=[*])C	-0.41	4 out of 18
SCFP_8	12	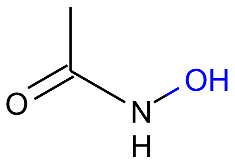 [*]O[*]	0	29 out of 79

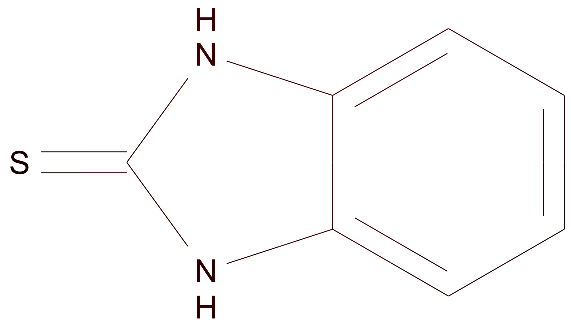

$C_7H_6N_2S$
Molecular Weight: 150.20094
ALogP: 2.536
Rotatable Bonds: 0
Acceptors: 1
Donors: 2

Model Prediction

Prediction: Sensitizer
Probability: 0.854
Enrichment: 1.24
Bayesian Score: 1.49
Mahalanobis Distance: 4.75
Mahalanobis Distance p-value: 0.996

Prediction: Positive if the Bayesian score is above the estimated best cutoff value from minimizing the false positive and false negative rate.
Probability: The esimated probability that the sample is in the positive category. This assumes that the Bayesian score follows a normal distribution and is different from the prediction using a cutoff.
Enrichment: An estimate of enrichment, that is, the increased likelihood (versus random) of this sample being in the category.
Bayesian Score: The standard Laplacian-modified Bayesian score.
Mahalanobis Distance: The Mahalanobis distance (MD) is the distance to the center of the training data. The larger the MD, the less trustworthy the prediction.
Mahalanobis Distance p-value: The p-value gives the fraction of training data with an MD greater than or equal to the one for the given sample, assuming normally distributed data. The smaller the p-value, the less trustworthy the prediciton. For highly non-normal X properties (e.g., fingerprints), the MD p-value is wildly inaccurate.

Structural Similar Compounds

Name	o-Aminophenol	o-Phenylenediamine	3-Aminophenol
Structure	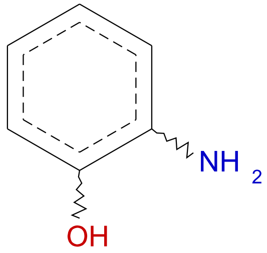	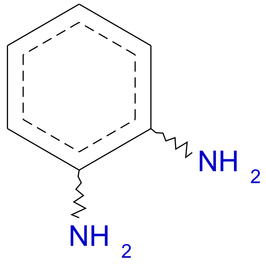	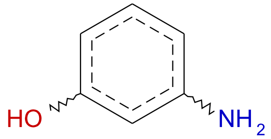
Actual Endpoint	Sensitizer	Sensitizer	Sensitizer
Predicted Endpoint	Sensitizer	Sensitizer	Sensitizer
Distance	0.572	0.575	0.596
Reference	Contact Dermatitis (1985) 13:226	Contact Dermatitis (1985) 13:226	SAR and QSAR in Env Res (1994) 2:159

Model Applicability

Unknown features are fingerprint features in the query molecule, but not found or appearing too infrequently in the training set.

1. All properties and OPS components are within expected ranges.

Feature Contribution

Top features for positive contribution

Fingerprint	Bit/Smiles	Feature Structure	Score	Sensitizer in training set
FCFP_12	-1724769936	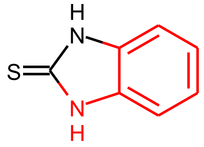 [*]1[*][c]2:[cH]:[cH]:[cH]:[cH]:[c]:2N1	0.274	6 out of 6

FCFP_12	-773983804	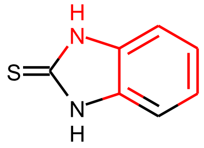 [*]1[*][c]2:[*]:[cH]:[cH]:[cH]:[c]:2N1	0.249	28 out of 30
FCFP_12	590925877	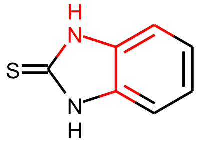 [*]:[cH]:[c]1N[*][*][c]:1:[*]	0.221	29 out of 32

Top Features for negative contribution

Fingerprint	Bit/Smiles	Feature Structure	Score	Sensitizer in training set
FCFP_12	3	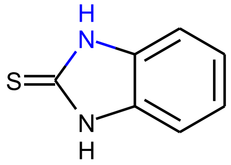 [*]N[*]	-0.0947	89 out of 136
FCFP_12	0	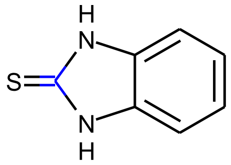 [*]C(=[*])[*]	0	255 out of 371
FCFP_12	1	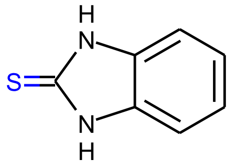 [*]=S	0	232 out of 338

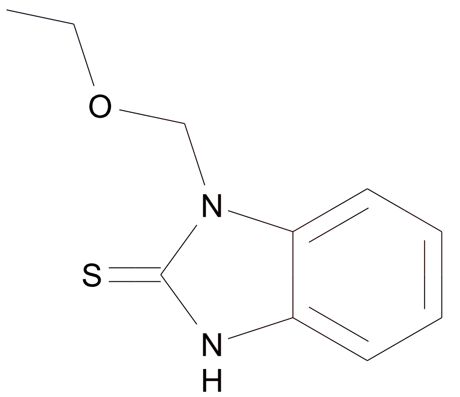

$C_{10}H_{12}N_2OS$
Molecular Weight: 208.28008
ALogP: 3.09
Rotatable Bonds: 3
Acceptors: 2
Donors: 1

Model Prediction

Prediction: Sensitizer
Probability: 0.806
Enrichment: 1.17
Bayesian Score: 0.361
Mahalanobis Distance: 5.48
Mahalanobis Distance p-value: 0.94

Prediction: Positive if the Bayesian score is above the estimated best cutoff value from minimizing the false positive and false negative rate.
Probability: The esimated probability that the sample is in the positive category. This assumes that the Bayesian score follows a normal distribution and is different from the prediction using a cutoff.
Enrichment: An estimate of enrichment, that is, the increased likelihood (versus random) of this sample being in the category.
Bayesian Score: The standard Laplacian-modified Bayesian score.
Mahalanobis Distance: The Mahalanobis distance (MD) is the distance to the center of the training data. The larger the MD, the less trustworthy the prediction.
Mahalanobis Distance p-value: The p-value gives the fraction of training data with an MD greater than or equal to the one for the given sample, assuming normally distributed data. The smaller the p-value, the less trustworthy the prediciton. For highly non-normal X properties (e.g., fingerprints), the MD p-value is wildly inaccurate.

Structural Similar Compounds

Name	Phenylsalicylate	Ethylene toluene sulfonamide	Propyl paraben
Structure	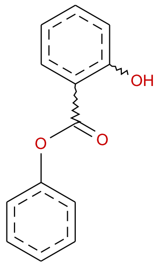	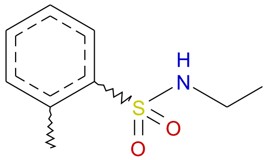	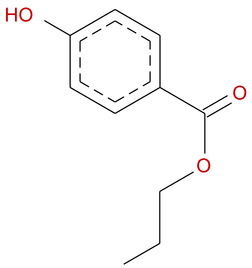
Actual Endpoint	Sensitizer	Non-Sensitizer	Non-Sensitizer
Predicted Endpoint	Sensitizer	Non-Sensitizer	Non-Sensitizer
Distance	0.549	0.563	0.580
Reference	Arch Derm Res (1982) 272:61	Howard I Maibach (priv comm)	SAR and QSAR in Env Res (1994) 2:159

Model Applicability

Unknown features are fingerprint features in the query molecule, but not found or appearing too infrequently in the training set.

1. All properties and OPS components are within expected ranges.

Feature Contribution

Top features for positive contribution

Fingerprint	Bit/Smiles	Feature Structure	Score	Sensitizer in training set
FCFP_12	-1724769936	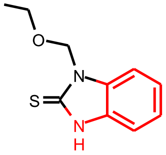 [*]1[*][c]2:[cH]:[cH]:[cH]:[cH]:[c]:2N1	0.274	6 out of 6

FCFP_12	675769755	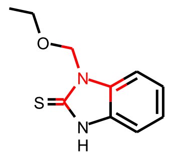<br>[*]CN1C(=[*])[*][*]:[c]1:[*]	0.253	4 out of 4
FCFP_12	-773983804	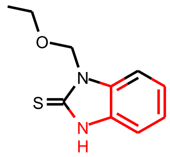<br>[*]1[*][c]2:[*]:[cH]:[cH]:[cH]:[c]:2N1	0.249	28 out of 30
**Top Features for negative contribution**				
**Fingerprint**	**Bit/Smiles**	**Feature Structure**	**Score**	**Sensitizer in training set**
FCFP_12	-1155471474	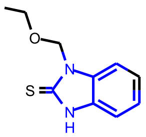<br>[*]N1C(=[*])N[c]2:[cH]:[cH]:[*]:[cH]:[c]1:2	-0.542	0 out of 1
FCFP_12	-1272768868	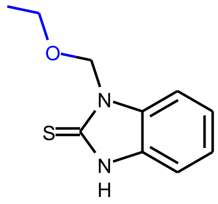<br>[*]OCC	-0.31	52 out of 99
FCFP_12	-1143715940	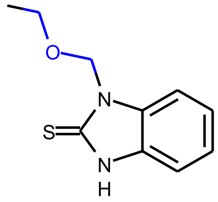<br>[*]COC[*]	-0.134	78 out of 124

HAE TOPKAT_Skin_Sensitization_None_vs_Sensitizer

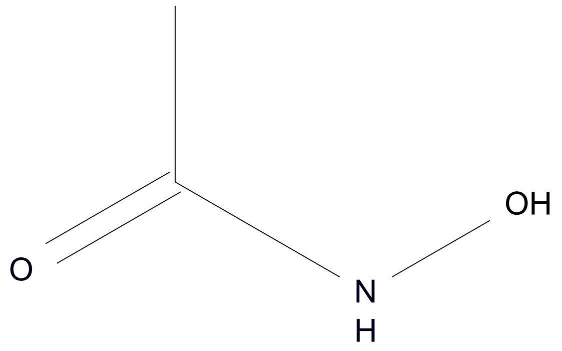

$C_2H_5NO_2$

Molecular Weight: 75.0666

ALogP: -0.82

Rotatable Bonds: 0

Acceptors: 2

Donors: 2

Model Prediction

Prediction: Non-Sensitizer

Probability: 0.719

Enrichment: 1.05

Bayesian Score: -1.45

Mahalanobis Distance: 5.73

Mahalanobis Distance p-value: 0.882

Prediction: Positive if the Bayesian score is above the estimated best cutoff value from minimizing the false positive and false negative rate.
Probability: The esimated probability that the sample is in the positive category. This assumes that the Bayesian score follows a normal distribution and is different from the prediction using a cutoff.
Enrichment: An estimate of enrichment, that is, the increased likelihood (versus random) of this sample being in the category.
Bayesian Score: The standard Laplacian-modified Bayesian score.
Mahalanobis Distance: The Mahalanobis distance (MD) is the distance to the center of the training data. The larger the MD, the less trustworthy the prediction.
Mahalanobis Distance p-value: The p-value gives the fraction of training data with an MD greater than or equal to the one for the given sample, assuming normally distributed data. The smaller the p-value, the less trustworthy the prediciton. For highly non-normal X properties (e.g., fingerprints), the MD p-value is wildly inaccurate.

Structural Similar Compounds

Name	Lactic Acid	Acetamide	Monoethanolamine
Structure	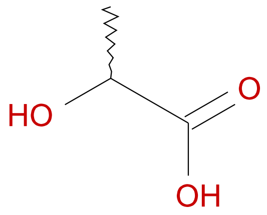	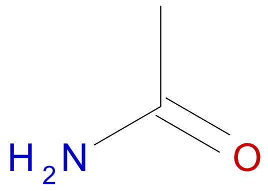	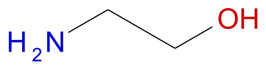
Actual Endpoint	Non-Sensitizer	Non-Sensitizer	Non-Sensitizer
Predicted Endpoint	Non-Sensitizer	Non-Sensitizer	Non-Sensitizer
Distance	0.415	0.424	0.485
Reference	SAR and QSAR in Env Res (1994) 2:159	SAR and QSAR in Env Res (1994) 2:159	Dermatosen (1996) 44:222

Model Applicability

Unknown features are fingerprint features in the query molecule, but not found or appearing too infrequently in the training set.

1. All properties and OPS components are within expected ranges.
2. Unknown FCFP_2 feature: 1666606459: [*]C(=[*])NO
3. Unknown FCFP_2 feature: -548542844: [*]NO

Feature Contribution

Top Features for negative contribution				
**Fingerprint**	**Bit/Smiles**	**Feature Structure**	**Score**	**Sensitizer in training set**
FCFP_12	566058135	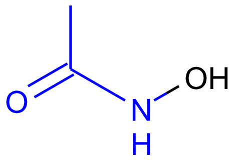 [*]NC(=O)C	-0.386	8 out of 17

FCFP_12	3	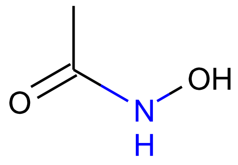<br>[*]N[*]	-0.0947	89 out of 136
FCFP_12	7	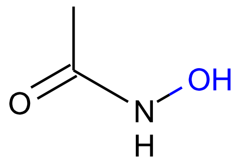<br>[*]O	-0.0926	80 out of 122

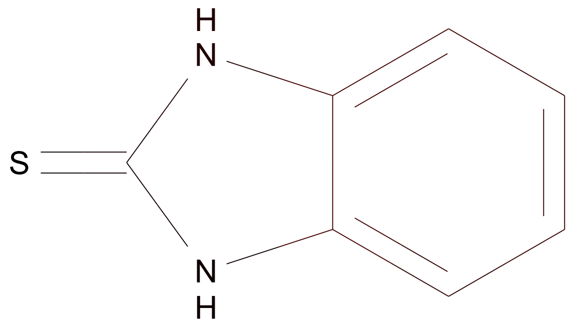

$C_7H_6N_2S$
Molecular Weight: 150.20094
ALogP: 2.536
Rotatable Bonds: 0
Acceptors: 1
Donors: 2

Model Prediction

Prediction: Strong-Sensitizer
Probability: 0.924
Enrichment: 1.19
Bayesian Score: 1.31
Mahalanobis Distance: 4.76
Mahalanobis Distance p-value: 0.951

Prediction: Positive if the Bayesian score is above the estimated best cutoff value from minimizing the false positive and false negative rate.
Probability: The esimated probability that the sample is in the positive category. This assumes that the Bayesian score follows a normal distribution and is different from the prediction using a cutoff.
Enrichment: An estimate of enrichment, that is, the increased likelihood (versus random) of this sample being in the category.
Bayesian Score: The standard Laplacian-modified Bayesian score.
Mahalanobis Distance: The Mahalanobis distance (MD) is the distance to the center of the training data. The larger the MD, the less trustworthy the prediction.
Mahalanobis Distance p-value: The p-value gives the fraction of training data with an MD greater than or equal to the one for the given sample, assuming normally distributed data. The smaller the p-value, the less trustworthy the prediciton. For highly non-normal X properties (e.g., fingerprints), the MD p-value is wildly inaccurate.

Structural Similar Compounds

Name	o-Aminophenol	o-Phenylenediamine	2-Chloro-p-phenylenediamine
Structure	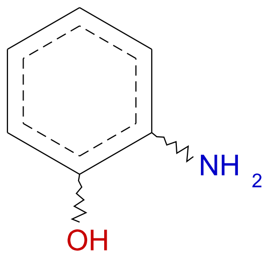	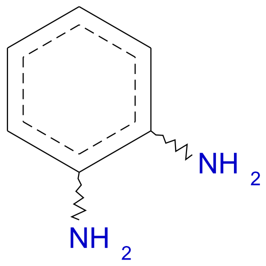	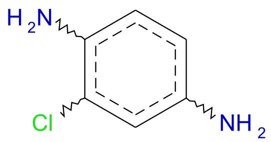
Actual Endpoint	Strong-Sensitizer	Strong-Sensitizer	Strong-Sensitizer
Predicted Endpoint	Strong-Sensitizer	Strong-Sensitizer	Strong-Sensitizer
Distance	0.599	0.615	0.620
Reference	Contact Dermatitis (1985) 13:226	Contact Dermatitis (1985) 13:226	J Am Coll Toxicol (1992) 11(4):521

Model Applicability

Unknown features are fingerprint features in the query molecule, but not found or appearing too infrequently in the training set.

1. All properties and OPS components are within expected ranges.

Feature Contribution

Top features for positive contribution

Fingerprint	Bit/Smiles	Feature Structure	Score	Strong-Sensitizer in training set
FCFP_12	16	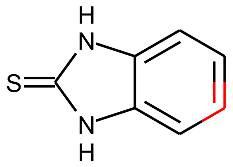 [*]:[cH]:[*]	0.232	165 out of 165

FCFP_12	1618154665	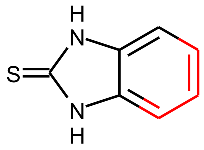 [*]:[cH]:[cH]:[cH]:[*]	0.232	164 out of 164
FCFP_12	991735244	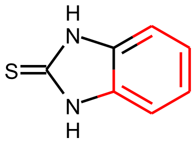 [*][c]1:[*]:[cH]:[cH] :[cH]:[cH]:1	0.23	76 out of 76

Top Features for negative contribution

Fingerprint	Bit/Smiles	Feature Structure	Score	Strong-Sensitizer in training set
FCFP_12	1499521844	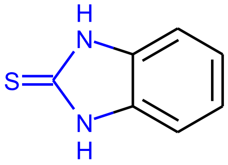 S=C1N[*]:[*]N1	-0.329	2 out of 4
FCFP_12	3	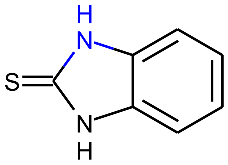 [*]N[*]	-0.131	61 out of 88
FCFP_12	1	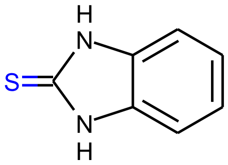 [*]=S	0	173 out of 222

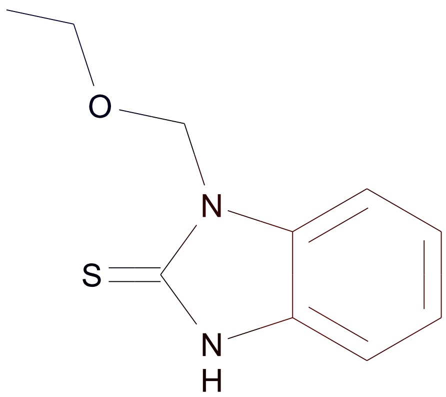

$C_{10}H_{12}N_2OS$
Molecular Weight: 208.28008
ALogP: 3.09
Rotatable Bonds: 3
Acceptors: 2
Donors: 1

Model Prediction

Prediction: Strong-Sensitizer
Probability: 0.971
Enrichment: 1.25
Bayesian Score: 1.7
Mahalanobis Distance: 5.36
Mahalanobis Distance p-value: 0.789

Prediction: Positive if the Bayesian score is above the estimated best cutoff value from minimizing the false positive and false negative rate.
Probability: The esimated probability that the sample is in the positive category. This assumes that the Bayesian score follows a normal distribution and is different from the prediction using a cutoff.
Enrichment: An estimate of enrichment, that is, the increased likelihood (versus random) of this sample being in the category.
Bayesian Score: The standard Laplacian-modified Bayesian score.
Mahalanobis Distance: The Mahalanobis distance (MD) is the distance to the center of the training data. The larger the MD, the less trustworthy the prediction.
Mahalanobis Distance p-value: The p-value gives the fraction of training data with an MD greater than or equal to the one for the given sample, assuming normally distributed data. The smaller the p-value, the less trustworthy the prediciton. For highly non-normal X properties (e.g., fingerprints), the MD p-value is wildly inaccurate.

Structural Similar Compounds

Name	Phenylsalicylate	Benzyl salicylate	Benzocaine
Structure	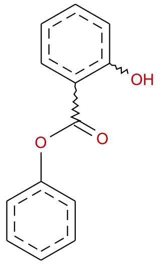	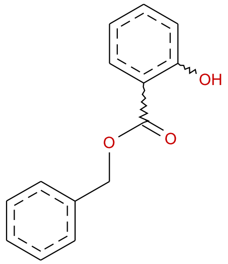	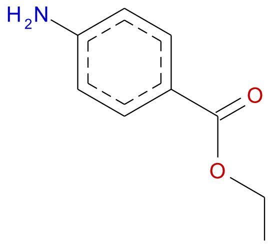
Actual Endpoint	Strong-Sensitizer	Strong-Sensitizer	Strong-Sensitizer
Predicted Endpoint	Strong-Sensitizer	Strong-Sensitizer	Strong-Sensitizer
Distance	0.542	0.595	0.629
Reference	Arch Derm Res (1982) 272:61	Contact Dermatitis (1993) 28:235	Contact Dermatitis (1993) 28:235

Model Applicability

Unknown features are fingerprint features in the query molecule, but not found or appearing too infrequently in the training set.

1. All properties and OPS components are within expected ranges.

Feature Contribution

Top features for positive contribution				
**Fingerprint**	**Bit/Smiles**	**Feature Structure**	**Score**	**Strong-Sensitizer in training set**
FCFP_12	16	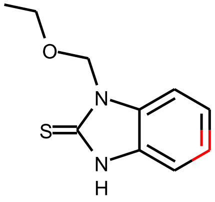 [*]:[cH]:[*]	0.232	165 out of 165

FCFP_12	1618154665	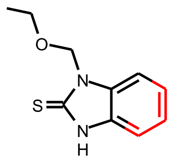 [*]:[cH]:[cH]:[cH]:[*]	0.232	164 out of 164
FCFP_12	332760439	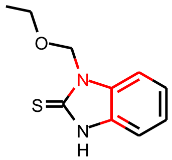 [*]N1[*][*][c](:[*]):[c]1:[cH]:[*]	0.23	80 out of 80

Top Features for negative contribution

Fingerprint	Bit/Smiles	Feature Structure	Score	Strong-Sensitizer in training set
FCFP_12	65948508	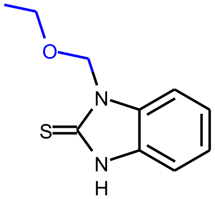 [*]COCC	-0.406	6 out of 12
FCFP_12	-1986098826	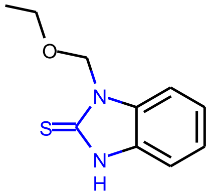 [*]N1[*]:[*]NC1=S	-0.256	1 out of 2
FCFP_12	136597326	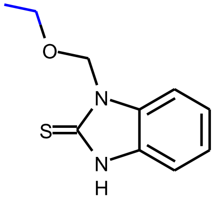 [*]CC	-0.239	74 out of 119

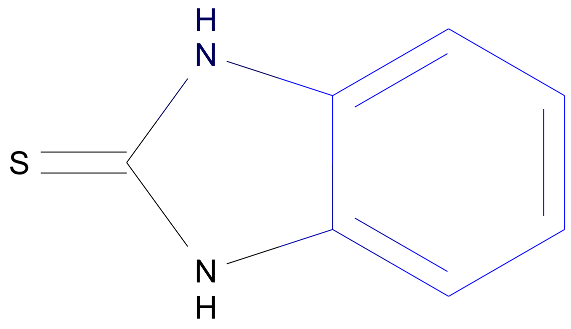

$C_7H_6N_2S$

Molecular Weight: 150.20094

ALogP: 2.536

Rotatable Bonds: 0

Acceptors: 1

Donors: 2

Model Prediction

Prediction: 97.3

Unit: mg/kg_body_weight/day

Mahalanobis Distance: 9.27

Mahalanobis Distance p-value: 0.184

Mahalanobis Distance: The Mahalanobis distance (MD) is a generalization of the Euclidean distance that accounts for correlations among the X properties. It is calculated as the distance to the center of the training data. The larger the MD, the less trustworthy the prediction.
Mahalanobis Distance p-value: The p-value gives the fraction of training data with an MD greater than or equal to the one for the given sample, assuming normally distributed data. The smaller the p-value, the less trustworthy the prediciton. For highly non-normal X properties (e.g., fingerprints), the MD p-value is wildly inaccurate.

Structural Similar Compounds

Name	Catechol	577	p-Tolylurea
Structure	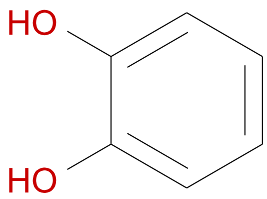	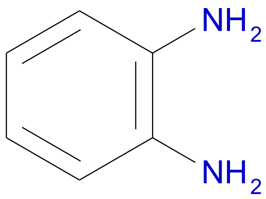	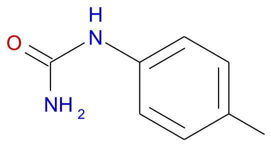
Actual Endpoint (-log C)	2.65444	2.1677	2.86274
Predicted Endpoint (-log C)	2.38537	2.56409	3.19105
Distance	0.583	0.617	0.617
Reference	CPDB	CPDB	CPDB

Model Applicability

Unknown features are fingerprint features in the query molecule, but not found or appearing too infrequently in the training set.

1. All properties and OPS components are within expected ranges.

Feature Contribution

Top Features for negative contribution			
**Fingerprint**	**Bit/Smiles**	**Feature Structure**	**Score**
ECFP_6	1996767644	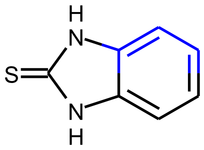 [*][c](:[*]):[cH]:[cH]:[*]	-0.251

ECFP_6	642810091	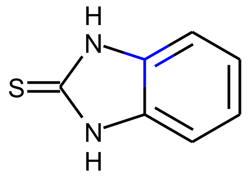 [*][c](:[*]):[*]	-0.247
ECFP_6	-182236392	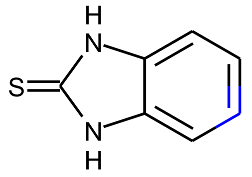 [*]:[cH]:[*]	-0.232

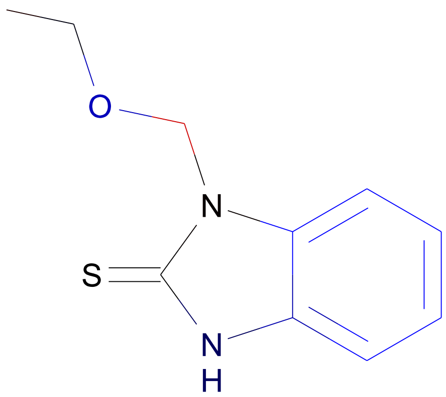

$C_{10}H_{12}N_2OS$
Molecular Weight: 208.28008
ALogP: 3.09
Rotatable Bonds: 3
Acceptors: 2
Donors: 1

Model Prediction

Prediction: 164
Unit: mg/kg_body_weight/day
Mahalanobis Distance: 12.9
Mahalanobis Distance p-value: 1.95e-007

Mahalanobis Distance: The Mahalanobis distance (MD) is a generalization of the Euclidean distance that accounts for correlations among the X properties. It is calculated as the distance to the center of the training data. The larger the MD, the less trustworthy the prediction.
Mahalanobis Distance p-value: The p-value gives the fraction of training data with an MD greater than or equal to the one for the given sample, assuming normally distributed data. The smaller the p-value, the less trustworthy the prediciton. For highly non-normal X properties (e.g., fingerprints), the MD p-value is wildly inaccurate.

Structural Similar Compounds

Name	666	Ciprofibrate	o-Ethoxybenzamide
Structure	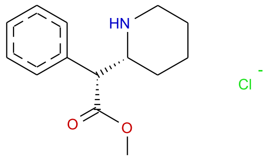	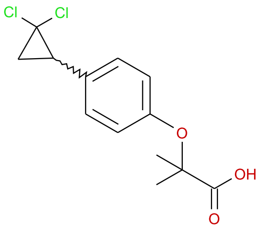	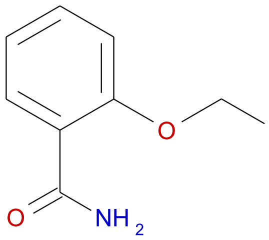
Actual Endpoint (-log C)	3.36793	4.66874	2.50786
Predicted Endpoint (-log C)	2.71924	3.1937	2.72346
Distance	0.588	0.600	0.606
Reference	CPDB	CPDB	CPDB

Model Applicability

Unknown features are fingerprint features in the query molecule, but not found or appearing too infrequently in the training set.

1. All properties and OPS components are within expected ranges.
2. Unknown ECFP_2 feature: -1236953626: [*]N1[*][*][c](:[*]):[c]1:[cH]:[*]
3. Unknown ECFP_2 feature: -661097313: [*]CN1C(=[*])[*][*]:[c]1:[*]
4. Unknown ECFP_2 feature: 434461827: [*]N1[*]:[*]NC1=S
5. Unknown ECFP_2 feature: -39008081: [*]OCN([*])[*]

Feature Contribution

Top features for positive contribution

Fingerprint	Bit/Smiles	Feature Structure	Score
ECFP_6	1559650422	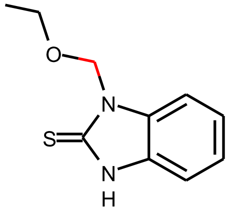 [*]C[*]	0.203

ECFP_6	734603939	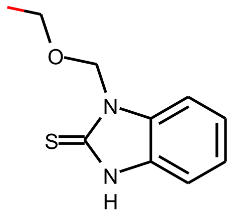 [*]C	0.0424
ECFP_6	670515721	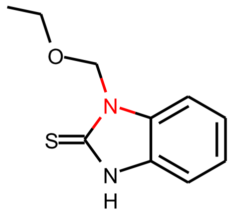 [*]N([*])[*]	0.00735
**Top Features for negative contribution**			
**Fingerprint**	**Bit/Smiles**	**Feature Structure**	**Score**
ECFP_6	1996767644	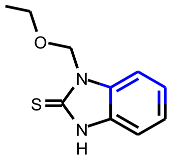 [*][c](:[*]):[cH]:[cH]:[*]	-0.251
ECFP_6	642810091	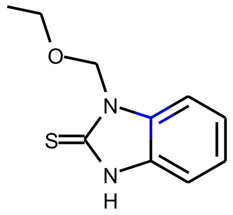 [*][c](:[*]):[*]	-0.247
ECFP_6	-182236392	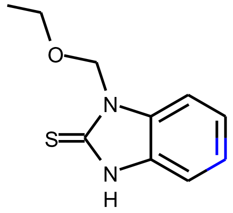 [*]:[cH]:[*]	-0.232

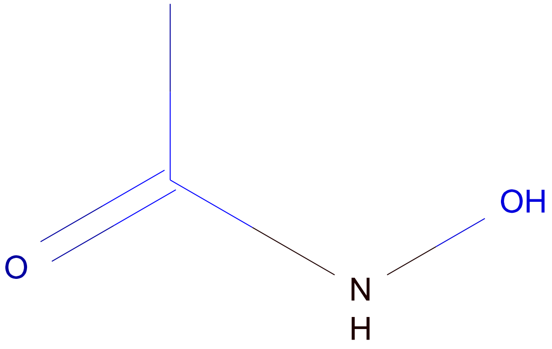

$C_2H_5NO_2$
Molecular Weight: 75.0666
ALogP: -0.82
Rotatable Bonds: 0
Acceptors: 2
Donors: 2

Model Prediction

Prediction: 107
Unit: mg/kg_body_weight/day
Mahalanobis Distance: 7.43
Mahalanobis Distance p-value: 0.94

Mahalanobis Distance: The Mahalanobis distance (MD) is a generalization of the Euclidean distance that accounts for correlations among the X properties. It is calculated as the distance to the center of the training data. The larger the MD, the less trustworthy the prediction.
Mahalanobis Distance p-value: The p-value gives the fraction of training data with an MD greater than or equal to the one for the given sample, assuming normally distributed data. The smaller the p-value, the less trustworthy the prediciton. For highly non-normal X properties (e.g., fingerprints), the MD p-value is wildly inaccurate.

Structural Similar Compounds

Name	Monoacetyl hydrazine	Acetamide	Methylhydrazine s
Structure	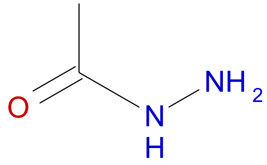	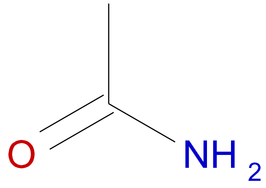	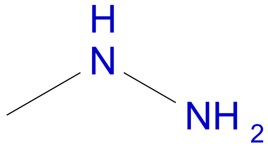
Actual Endpoint (-log C)	3.87628	1.29278	3.78549
Predicted Endpoint (-log C)	3.31537	3.09647	3.69975
Distance	0.321	0.463	0.489
Reference	CPDB	CPDB	CPDB

Model Applicability

Unknown features are fingerprint features in the query molecule, but not found or appearing too infrequently in the training set.

1. All properties and OPS components are within expected ranges.
2. Unknown ECFP_2 feature: 379232550: [*]C(=[*])NO
3. Unknown ECFP_2 feature: 2023553742: [*]NO

Feature Contribution

Top features for positive contribution

Fingerprint	Bit/Smiles	Feature Structure	Score
ECFP_6	734603939	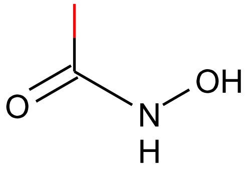 [*]C	0.0424

ECFP_6	-1897341097	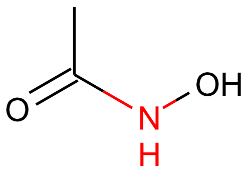 [*]N[*]	0.0284

Top Features for negative contribution			
**Fingerprint**	**Bit/Smiles**	**Feature Structure**	**Score**
ECFP_6	-1884411803	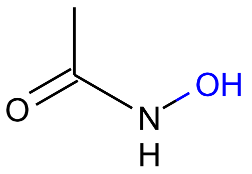 [*]O	-0.217
ECFP_6	866218936	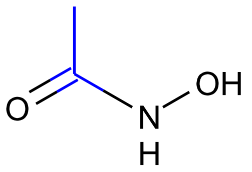 [*]C(=[*])C	-0.197
ECFP_6	2099970318	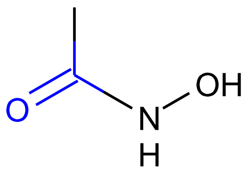 [*]C(=O)[*]	-0.118

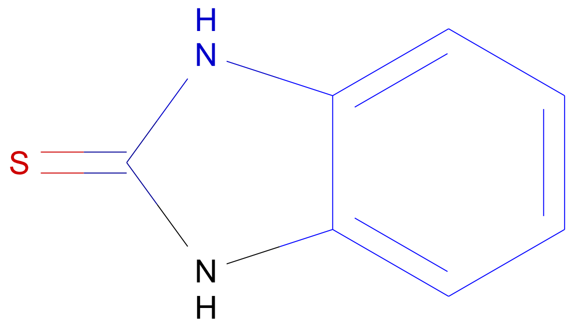

$C_7H_6N_2S$
Molecular Weight: 150.20094
ALogP: 2.536
Rotatable Bonds: 0
Acceptors: 1
Donors: 2

Model Prediction

Prediction: 74.8
Unit: mg/kg_body_weight/day
Mahalanobis Distance: 8.8
Mahalanobis Distance p-value: 0.769

Mahalanobis Distance: The Mahalanobis distance (MD) is a generalization of the Euclidean distance that accounts for correlations among the X properties. It is calculated as the distance to the center of the training data. The larger the MD, the less trustworthy the prediction.
Mahalanobis Distance p-value: The p-value gives the fraction of training data with an MD greater than or equal to the one for the given sample, assuming normally distributed data. The smaller the p-value, the less trustworthy the prediciton. For highly non-normal X properties (e.g., fingerprints), the MD p-value is wildly inaccurate.

Structural Similar Compounds

Name	2-Mercaptobenzothiazole	577	Acetaminophen
Structure	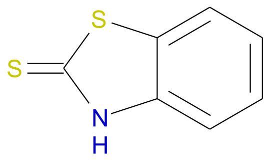	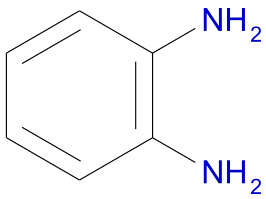	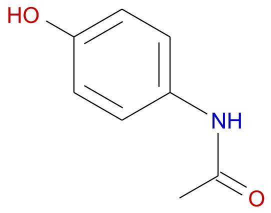
Actual Endpoint (-log C)	2.68681	2.63954	2.48484
Predicted Endpoint (-log C)	3.15302	2.76412	2.46116
Distance	0.494	0.532	0.546
Reference	CPDB	CPDB	CPDB

Model Applicability

Unknown features are fingerprint features in the query molecule, but not found or appearing too infrequently in the training set.

1. All properties and OPS components are within expected ranges.

Feature Contribution

Top features for positive contribution			
**Fingerprint**	**Bit/Smiles**	**Feature Structure**	**Score**
FCFP_6	1	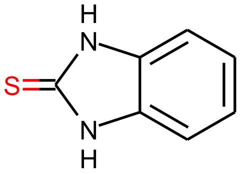 [*]=S	0.234

FCFP_6	3	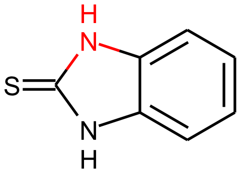 [*]N[*]	0.064
FCFP_6	-773983804	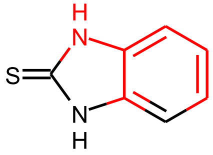 [*]1[*][c]2:[*]:[cH]:[cH]:[cH]:[c]:2N1	0.0614

Top Features for negative contribution

Fingerprint	Bit/Smiles	Feature Structure	Score
FCFP_6	991735244	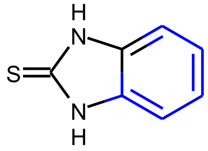 [*][c]1:[*]:[cH]:[cH]:[cH]:[cH]:1	-0.422
FCFP_6	16	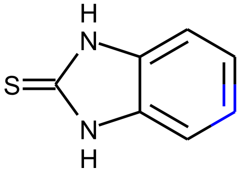 [*]:[cH]:[*]	-0.354
FCFP_6	590925877	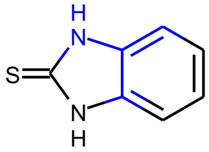 [*]:[cH]:[c]1N[*][*][c]:1:[*]	-0.323

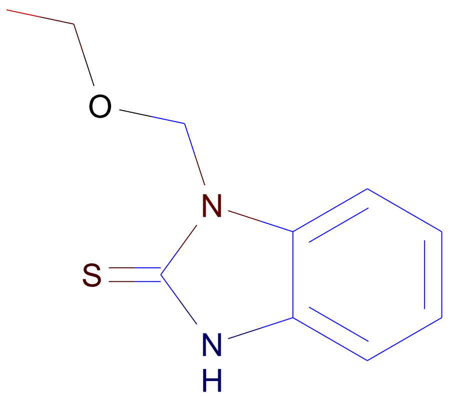

$C_{10}H_{12}N_2OS$
Molecular Weight: 208.28008
ALogP: 3.09
Rotatable Bonds: 3
Acceptors: 2
Donors: 1

Structural Similar Compounds

Name	2-Mercaptobenzothiazole	Phenacetin	p-Nitrosodiphenylamine
Structure	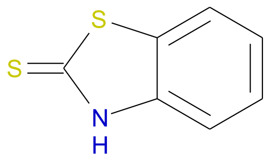	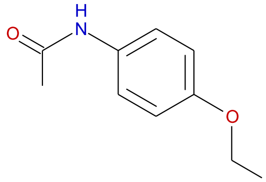	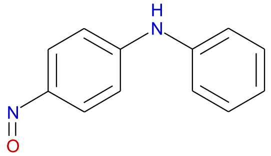
Actual Endpoint (-log C)	2.68681	2.15647	2.99395
Predicted Endpoint (-log C)	3.15302	2.91803	2.99566
Distance	0.487	0.545	0.557
Reference	CPDB	CPDB	CPDB

Model Prediction

Prediction: 128
Unit: mg/kg_body_weight/day
Mahalanobis Distance: 12.7
Mahalanobis Distance p-value: 1.88e-005

Mahalanobis Distance: The Mahalanobis distance (MD) is a generalization of the Euclidean distance that accounts for correlations among the X properties. It is calculated as the distance to the center of the training data. The larger the MD, the less trustworthy the prediction.
Mahalanobis Distance p-value: The p-value gives the fraction of training data with an MD greater than or equal to the one for the given sample, assuming normally distributed data. The smaller the p-value, the less trustworthy the prediciton. For highly non-normal X properties (e.g., fingerprints), the MD p-value is wildly inaccurate.

Model Applicability

Unknown features are fingerprint features in the query molecule, but not found or appearing too infrequently in the training set.

1. All properties and OPS components are within expected ranges.

Feature Contribution

Top features for positive contribution			
**Fingerprint**	**Bit/Smiles**	**Feature Structure**	**Score**
FCFP_6	-2090462286	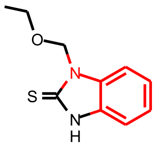 [*]N1[*][*][c]2:[cH]:[cH]:[cH]:[cH]:[c]1:2	0.245

FCFP_6	1	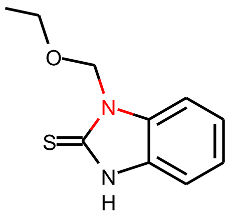 [*]=S	0.234
FCFP_6	-1272768868	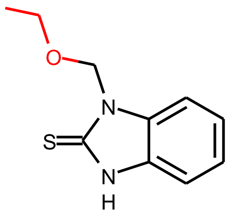 [*]OCC	0.127

Top Features for negative contribution

Fingerprint	Bit/Smiles	Feature Structure	Score
FCFP_6	991735244	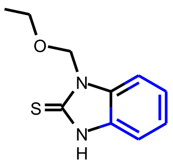 [*][c]1:[*]:[cH]:[cH]:[cH]:[cH]:1	-0.422
FCFP_6	16	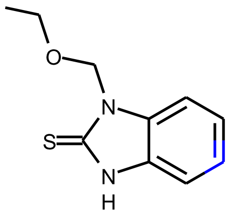 [*]:[cH]:[*]	-0.354
FCFP_6	590925877	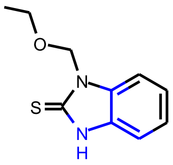 [*]:[cH]:[c]1N[*][*][c]:1:[*]	-0.323

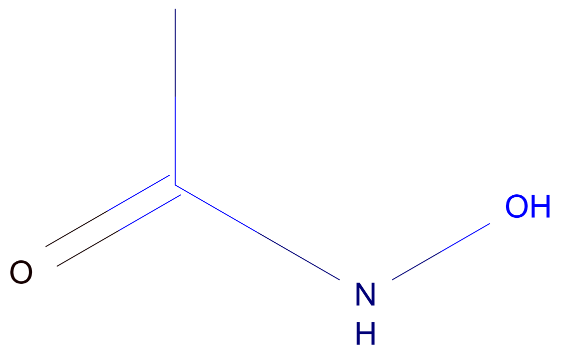

$C_2H_5NO_2$
Molecular Weight: 75.0666
ALogP: -0.82
Rotatable Bonds: 0
Acceptors: 2
Donors: 2

Model Prediction

Prediction: 82.2
Unit: mg/kg_body_weight/day
Mahalanobis Distance: 10.3
Mahalanobis Distance p-value: 0.105

Mahalanobis Distance: The Mahalanobis distance (MD) is a generalization of the Euclidean distance that accounts for correlations among the X properties. It is calculated as the distance to the center of the training data. The larger the MD, the less trustworthy the prediction.
Mahalanobis Distance p-value: The p-value gives the fraction of training data with an MD greater than or equal to the one for the given sample, assuming normally distributed data. The smaller the p-value, the less trustworthy the prediciton. For highly non-normal X properties (e.g., fingerprints), the MD p-value is wildly inaccurate.

Structural Similar Compounds

Name	Acetamide	Thioacetamide	Dimethylarsinic acid
Structure	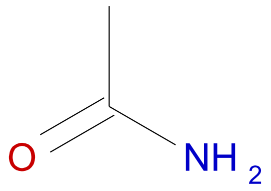	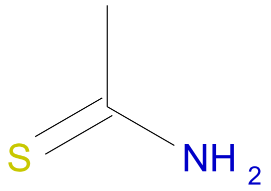	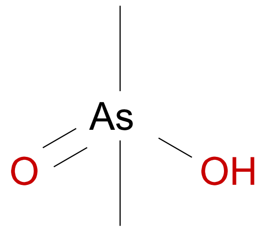
Actual Endpoint (-log C)	2.51607	3.81513	4.08297
Predicted Endpoint (-log C)	3.09872	3.027	3.61554
Distance	0.366	0.408	0.470
Reference	CPDB	CPDB	CPDB

Model Applicability

Unknown features are fingerprint features in the query molecule, but not found or appearing too infrequently in the training set.

1. All properties and OPS components are within expected ranges.
2. Unknown FCFP_2 feature: 1666606459: [*]C(=[*])NO
3. Unknown FCFP_2 feature: -548542844: [*]NO

Feature Contribution

Top features for positive contribution

Fingerprint	Bit/Smiles	Feature Structure	Score
FCFP_6	1	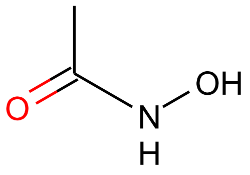 [*]=S	0.234

FCFP_6	136597326	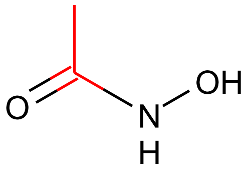 [*]CC	0.0695
FCFP_6	3	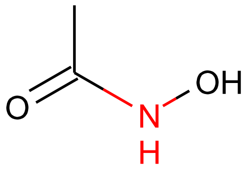 [*]N[*]	0.064
**Top Features for negative contribution**			
**Fingerprint**	**Bit/Smiles**	**Feature Structure**	**Score**
FCFP_6	7	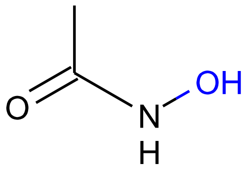 [*]O	-0.372
FCFP_6	566058135	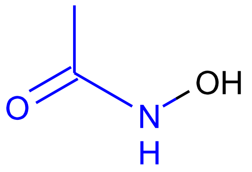 [*]NC(=O)C	-0.182
FCFP_6	0	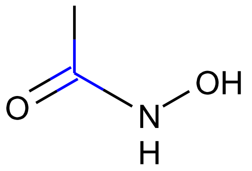 [*]C(=[*])[*]	-0.115

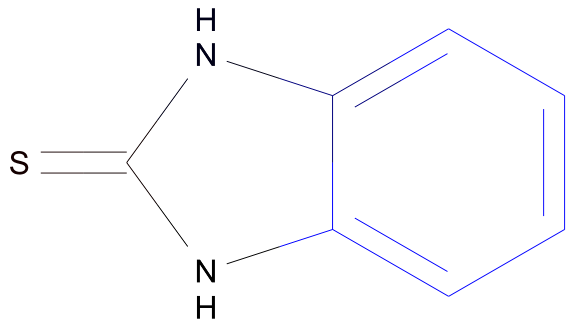

$C_7H_6N_2S$

Molecular Weight: 150.20094

ALogP: 2.536

Rotatable Bonds: 0

Acceptors: 1

Donors: 2

Model Prediction

Prediction: 0.0694

Unit: g/kg_body_weight

Mahalanobis Distance: 24.5

Mahalanobis Distance p-value: 1.16e-014

Mahalanobis Distance: The Mahalanobis distance (MD) is a generalization of the Euclidean distance that accounts for correlations among the X properties. It is calculated as the distance to the center of the training data. The larger the MD, the less trustworthy the prediction.
Mahalanobis Distance p-value: The p-value gives the fraction of training data with an MD greater than or equal to the one for the given sample, assuming normally distributed data. The smaller the p-value, the less trustworthy the prediciton. For highly non-normal X properties (e.g., fingerprints), the MD p-value is wildly inaccurate.

Structural Similar Compounds

Name	1-PHENYL-2-THIOUREA	2;6-DICHLORO-P-PHENYLENEDIAMIN	4-CHLORO-O-PHENYLENEDIAMINE
Structure	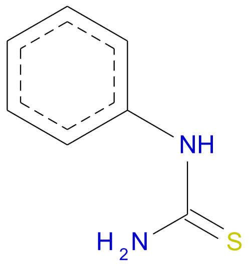	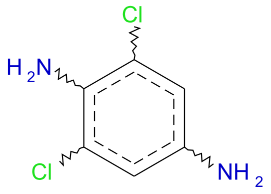	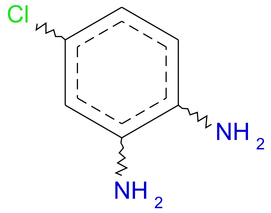
Actual Endpoint (-log C)	4.40431	3.54907	2.75614
Predicted Endpoint (-log C)	3.48637	3.72614	3.49591
Distance	0.430	0.520	0.541
Reference	NTP 148 C-8	NTP REPORT # 219	NTP 63 27

Model Applicability

Unknown features are fingerprint features in the query molecule, but not found or appearing too infrequently in the training set.

1. All properties and OPS components are within expected ranges.
2. Unknown ECFP_6 feature: -154530762: [*]N[*]
3. Unknown ECFP_6 feature: 1997021792: [*]:[cH]:[cH]:[cH]:[*]
4. Unknown ECFP_6 feature: 1335833675: [*]:[cH]:[c]1N[*][*][c]:1:[*]
5. Unknown ECFP_6 feature: -1699286547: [*]=C1[*][*]:[c](:[*])N1
6. Unknown ECFP_6 feature: -829433624: S=C1N[*]:[*]N1
7. Unknown ECFP_6 feature: 1985868180: [*]C(=S)[*]

Feature Contribution

Top features for positive contribution			
**Fingerprint**	**Bit/Smiles**	**Feature Structure**	**Score**

ECFP_6	-845108448	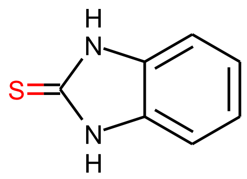<br>[*]=S	0.105
FCFP_6	3	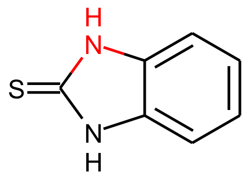<br>[*]N[*]	0.0924
ECFP_6	642810091	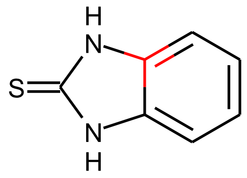<br>[*][c](:[*]):[*]	0.0424
**Top Features for negative contribution**			
**Fingerprint**	**Bit/Smiles**	**Feature Structure**	**Score**
FCFP_6	991735244	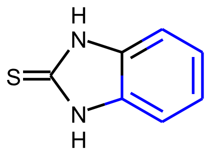<br>[*][c]1:[*]:[cH]:[cH]<br>:[cH]:[cH]:1	-0.134
ECFP_6	1564392544	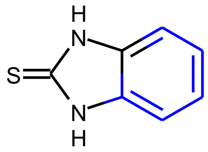<br>[*][c]1:[*]:[cH]:[cH]<br>:[cH]:[cH]:1	-0.133

FCFP_6	1	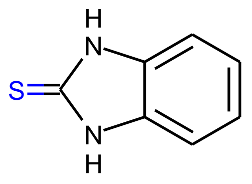 [*]=S	-0.102

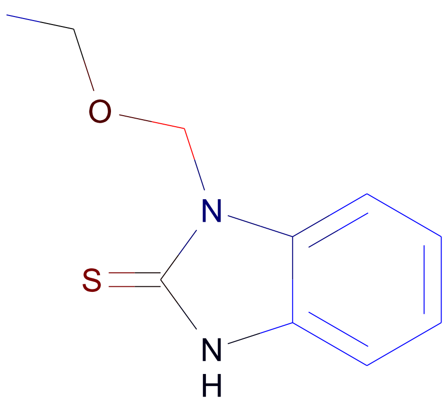

$C_{10}H_{12}N_2OS$
Molecular Weight: 208.28008
ALogP: 3.09
Rotatable Bonds: 3
Acceptors: 2
Donors: 1

Model Prediction

Prediction: 0.0341
Unit: g/kg_body_weight
Mahalanobis Distance: 38.4
Mahalanobis Distance p-value: 1.68e-039

Mahalanobis Distance: The Mahalanobis distance (MD) is a generalization of the Euclidean distance that accounts for correlations among the X properties. It is calculated as the distance to the center of the training data. The larger the MD, the less trustworthy the prediction.
Mahalanobis Distance p-value: The p-value gives the fraction of training data with an MD greater than or equal to the one for the given sample, assuming normally distributed data. The smaller the p-value, the less trustworthy the prediciton. For highly non-normal X properties (e.g., fingerprints), the MD p-value is wildly inaccurate.

Structural Similar Compounds

Name	CHLORPROPHAM	11	DICHLOROPROP
Structure	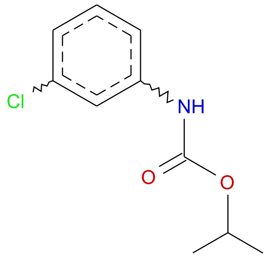	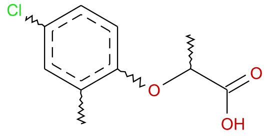	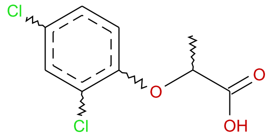
Actual Endpoint (-log C)	3.15363	3.91675	3.97324
Predicted Endpoint (-log C)	3.82083	3.52639	3.57411
Distance	0.490	0.520	0.524
Reference	COVER SHEET 0283;891101;(1)	EPA COVER SHEET 0067;890101;(1)	HEEP ECAO CIN P079;8408;(2)

Model Applicability

Unknown features are fingerprint features in the query molecule, but not found or appearing too infrequently in the training set.

1. All properties and OPS components are within expected ranges.
2. Unknown ECFP_6 feature: -154530762: [*]N[*]
3. Unknown ECFP_6 feature: 1997021792: [*]:[cH]:[cH]:[cH]:[*]
4. Unknown ECFP_6 feature: -1236953626: [*]N1[*][*][c](:[*]):[c]1:[cH]:[*]
5. Unknown ECFP_6 feature: 1335833675: [*]:[cH]:[c]1N[*][*][c]:1:[*]
6. Unknown ECFP_6 feature: -661097313: [*]CN1C(=[*])[*][*]:[c]1:[*]
7. Unknown ECFP_6 feature: 434461827: [*]N1[*]:[*]NC1=S
8. Unknown ECFP_6 feature: -1699286547: [*]=C1[*][*]:[c](:[*])N1
9. Unknown ECFP_6 feature: 1985868180: [*]C(=S)[*]
10. Unknown ECFP_6 feature: -39008081: [*]OCN([*])[*]
11. Unknown ECFP_6 feature: -1252314528: [*]COC[*]
12. Unknown ECFP_6 feature: -949601813: [*]OCC

Feature Contribution

Top features for positive contribution

Fingerprint	Bit/Smiles	Feature Structure	Score

FCFP_6	-1143715940	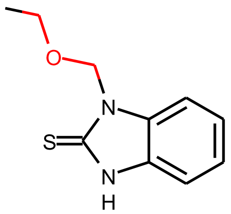 [*]COC[*]	0.13
ECFP_6	1559650422	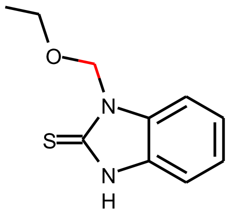 [*]C[*]	0.129
ECFP_6	-845108448	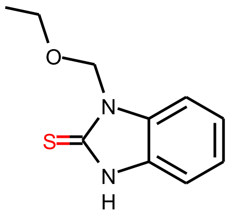 [*]=S	0.105
**Top Features for negative contribution**			
**Fingerprint**	**Bit/Smiles**	**Feature Structure**	**Score**
FCFP_6	991735244	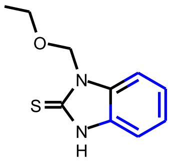 [*][c]1:[*]:[cH]:[cH]:[cH]:[cH]:1	-0.134
ECFP_6	1564392544	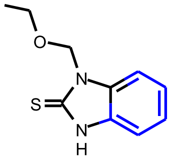 [*][c]1:[*]:[cH]:[cH]:[cH]:[cH]:1	-0.133

FCFP_6	1	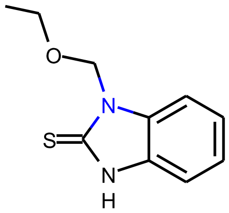 [*]=S	-0.102

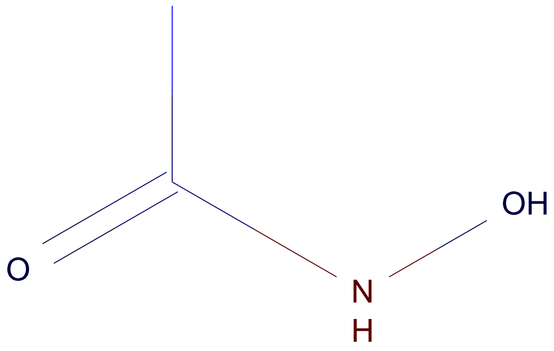

$C_2H_5NO_2$

Molecular Weight: 75.0666

ALogP: -0.82

Rotatable Bonds: 0

Acceptors: 2

Donors: 2

Model Prediction

Prediction: 0.424

Unit: g/kg_body_weight

Mahalanobis Distance: 21.9

Mahalanobis Distance p-value: 3.72e-010

Mahalanobis Distance: The Mahalanobis distance (MD) is a generalization of the Euclidean distance that accounts for correlations among the X properties. It is calculated as the distance to the center of the training data. The larger the MD, the less trustworthy the prediction.
Mahalanobis Distance p-value: The p-value gives the fraction of training data with an MD greater than or equal to the one for the given sample, assuming normally distributed data. The smaller the p-value, the less trustworthy the prediciton. For highly non-normal X properties (e.g., fingerprints), the MD p-value is wildly inaccurate.

Structural Similar Compounds

Name	MALEIC HYDRAZIDE	N-METHYLOLACRYLAMIDE	ETHYLENE GLYCOL
Structure	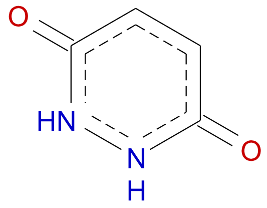	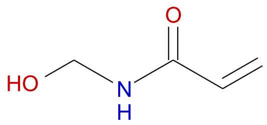	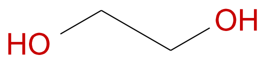
Actual Endpoint (-log C)	2.35059	4.3713	1.79287
Predicted Endpoint (-log C)	2.86351	3.60852	2.68139
Distance	0.461	0.486	0.503
Reference	HEED ECAO CIN G057;8908;(1)	NTP REPORT # 352	EPA COVER SHEET 0238;890901;(1)

Model Applicability

Unknown features are fingerprint features in the query molecule, but not found or appearing too infrequently in the training set.

1. All properties and OPS components are within expected ranges.
2. Unknown FCFP_2 feature: 1666606459: [*]C(=[*])NO
3. Unknown FCFP_2 feature: -548542844: [*]NO
4. Unknown ECFP_6 feature: -474544785: [*]NC(=O)C
5. Unknown ECFP_6 feature: 379232550: [*]C(=[*])NO
6. Unknown ECFP_6 feature: 2023553742: [*]NO

Feature Contribution

Top features for positive contribution			
**Fingerprint**	**Bit/Smiles**	**Feature Structure**	**Score**

FCFP_6	3	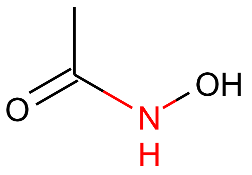 [*]N[*]	0.0924
ECFP_6	2099970318	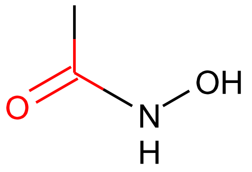 [*]C(=O)[*]	0.0766
ECFP_6	-1897341097	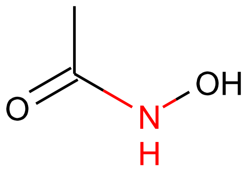 [*]N[*]	0.0537
**Top Features for negative contribution**			
**Fingerprint**	**Bit/Smiles**	**Feature Structure**	**Score**
FCFP_6	1	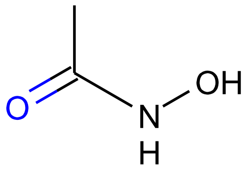 [*]=S	-0.102
FCFP_6	136597326	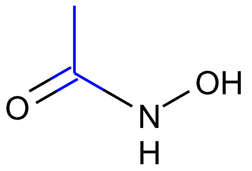 [*]CC	-0.0815

ECFP_6	866218936	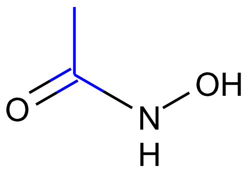<br>[*]C(=[*])C	-0.074

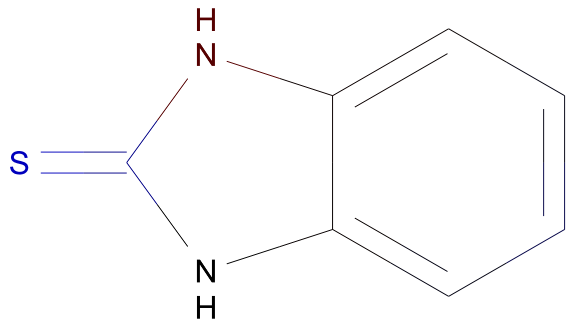

$C_7H_6N_2S$
Molecular Weight: 150.20094
ALogP: 2.536
Rotatable Bonds: 0
Acceptors: 1
Donors: 2

Model Prediction

Prediction: 0.116
Unit: g/kg_body_weight
Mahalanobis Distance: 7.86
Mahalanobis Distance p-value: 0.0344

Mahalanobis Distance: The Mahalanobis distance (MD) is a generalization of the Euclidean distance that accounts for correlations among the X properties. It is calculated as the distance to the center of the training data. The larger the MD, the less trustworthy the prediction.
Mahalanobis Distance p-value: The p-value gives the fraction of training data with an MD greater than or equal to the one for the given sample, assuming normally distributed data. The smaller the p-value, the less trustworthy the prediciton. For highly non-normal X properties (e.g., fingerprints), the MD p-value is wildly inaccurate.

Structural Similar Compounds

Name	1-PHENYL-2-THIOUREA	ACETAMINOPHEN (4-HYDROXYACETANILIDE)	2,6-DICHLORO-P-PHENYLENEDIAMINE
Structure	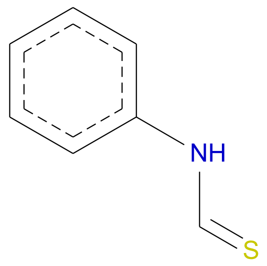	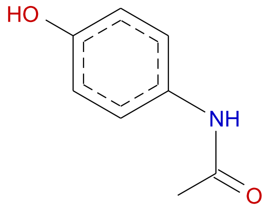	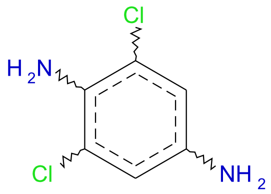
Actual Endpoint (-log C)	4.40496	2.74809	3.2938
Predicted Endpoint (-log C)	3.16907	2.67552	3.24284
Distance	0.298	0.411	0.473
Reference	NCI/NTP TR-148	NCI/NTP TR-394	NCI/NTP TR-219

Model Applicability

Unknown features are fingerprint features in the query molecule, but not found or appearing too infrequently in the training set.

1. All properties and OPS components are within expected ranges.

Feature Contribution

Top features for positive contribution			
**Fingerprint**	**Bit/Smiles**	**Feature Structure**	**Score**
FCFP_2	3	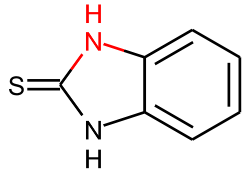 [*]N[*]	0.0737

FCFP_2	590925877	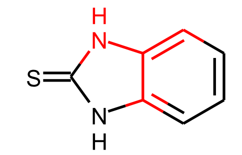 [*]:[cH]:[c]1N[*][*][c]:1:[*]	0.00762
FCFP_2	1294255210	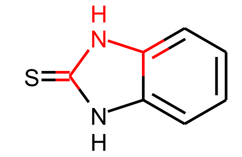 [*]=C1[*][*]:[c](:[*])N1	0.00319

Top Features for negative contribution

Fingerprint	Bit/Smiles	Feature Structure	Score
FCFP_2	1872154524	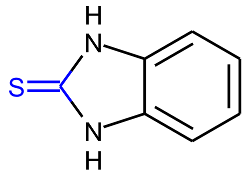 [*]C(=S)[*]	-0.105
FCFP_2	1	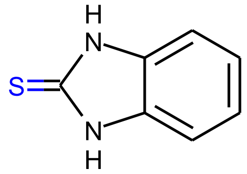 [*]=S	-0.0796
FCFP_2	16	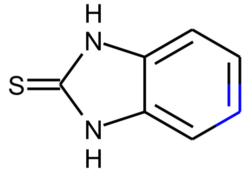 [*]:[cH]:[*]	-0.0512

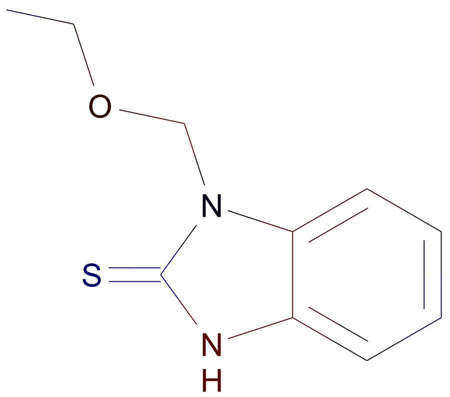

$C_{10}H_{12}N_2OS$
Molecular Weight: 208.28008
ALogP: 3.09
Rotatable Bonds: 3
Acceptors: 2
Donors: 1

Model Prediction

Prediction: 0.072
Unit: g/kg_body_weight
Mahalanobis Distance: 9.78
Mahalanobis Distance p-value: 9.98e-005

Mahalanobis Distance: The Mahalanobis distance (MD) is a generalization of the Euclidean distance that accounts for correlations among the X properties. It is calculated as the distance to the center of the training data. The larger the MD, the less trustworthy the prediction.
Mahalanobis Distance p-value: The p-value gives the fraction of training data with an MD greater than or equal to the one for the given sample, assuming normally distributed data. The smaller the p-value, the less trustworthy the prediciton. For highly non-normal X properties (e.g., fingerprints), the MD p-value is wildly inaccurate.

Structural Similar Compounds

Name	1-PHENYL-2-THIOUREA	MEXACARBATE	4-(CHLOROACETYL) ACETANILIDE
Structure	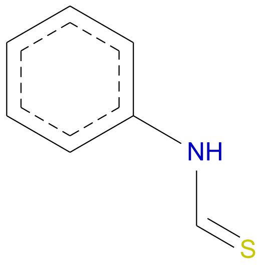	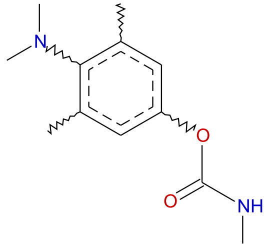	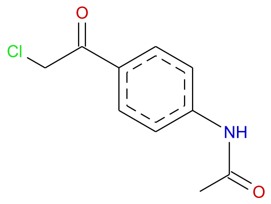
Actual Endpoint (-log C)	4.40496	4.07253	3.37137
Predicted Endpoint (-log C)	3.16907	3.7152	3.35582
Distance	0.430	0.432	0.461
Reference	NCI/NTP TR-148	NCI/NTP TR-147	NCI/NTP TR-177

Model Applicability

Unknown features are fingerprint features in the query molecule, but not found or appearing too infrequently in the training set.

1. All properties and OPS components are within expected ranges.

Feature Contribution

Top features for positive contribution			
**Fingerprint**	**Bit/Smiles**	**Feature Structure**	**Score**
FCFP_2	-1143715940	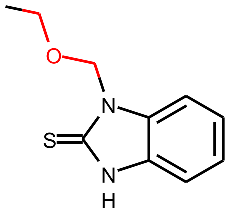 [*]COC[*]	0.095

FCFP_2	3	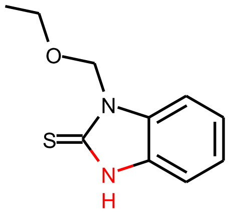 [*]N[*]	0.0737
FCFP_2	332760439	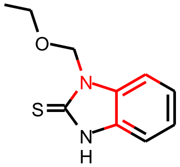 [*]N1[*][*][c](:[*]):[c]1:[cH]:[*]	0.0611

Top Features for negative contribution

Fingerprint	Bit/Smiles	Feature Structure	Score
FCFP_2	1872154524	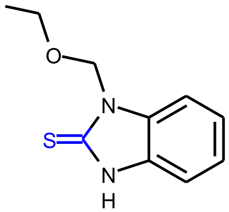 [*]C(=S)[*]	-0.105
FCFP_2	1	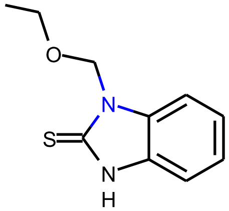 [*]=S	-0.0796
FCFP_2	16	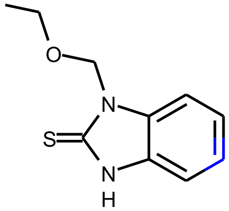 [*]:[cH]:[*]	-0.0512

HAE

TOPKAT_Rat_Maximum_Tolerated_Dose_Feed

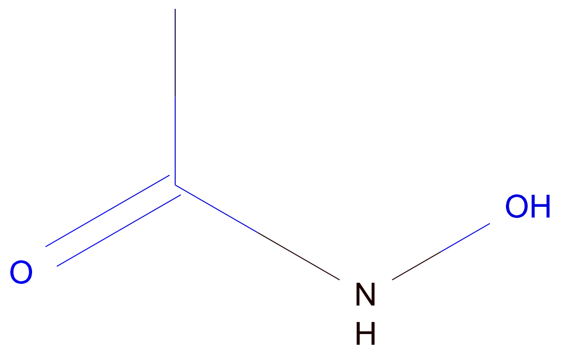

$C_2H_5NO_2$

Molecular Weight: 75.0666

ALogP: -0.82

Rotatable Bonds: 0

Acceptors: 2

Donors: 2

Model Prediction

Prediction: 0.135

Unit: g/kg_body_weight

Mahalanobis Distance: 11.2

Mahalanobis Distance p-value: 3.19e-007

Mahalanobis Distance: The Mahalanobis distance (MD) is a generalization of the Euclidean distance that accounts for correlations among the X properties. It is calculated as the distance to the center of the training data. The larger the MD, the less trustworthy the prediction.
Mahalanobis Distance p-value: The p-value gives the fraction of training data with an MD greater than or equal to the one for the given sample, assuming normally distributed data. The smaller the p-value, the less trustworthy the prediciton. For highly non-normal X properties (e.g., fingerprints), the MD p-value is wildly inaccurate.

Structural Similar Compounds

Name	ETHYLENE THIOUREA (ETU)	CAPROLACTAM	ACETAMINOPHEN (4-HYDROXYACETANILIDE)
Structure	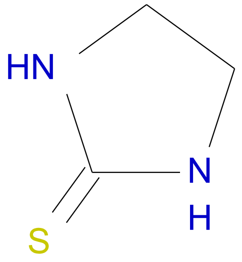	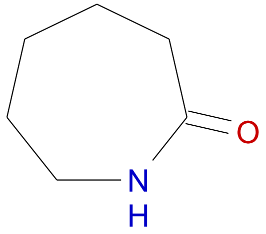	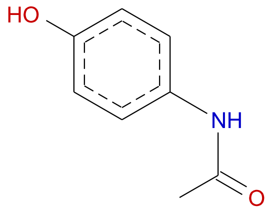
Actual Endpoint (-log C)	3.98807	2.52542	2.74809
Predicted Endpoint (-log C)	3.2588	3.29258	2.67552
Distance	0.490	0.544	0.552
Reference	NCI/NTP TR-388	NCI/NTP TR-214	NCI/NTP TR-394

Model Applicability

Unknown features are fingerprint features in the query molecule, but not found or appearing too infrequently in the training set.

1. All properties and OPS components are within expected ranges.
2. Unknown FCFP_2 feature: 1666606459: [*]C(=[*])NO
3. Unknown FCFP_2 feature: -548542844: [*]NO

Feature Contribution

Top features for positive contribution

Fingerprint	Bit/Smiles	Feature Structure	Score
FCFP_2	3	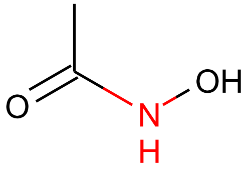 [*]N[*]	0.0737

Top Features for negative contribution

Fingerprint	Bit/Smiles	Feature Structure	Score

FCFP_2	7	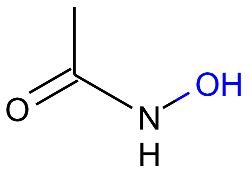 [*]O	-0.214
FCFP_2	1872154524	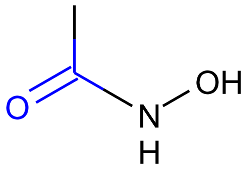 [*]C(=S)[*]	-0.105
FCFP_2	1	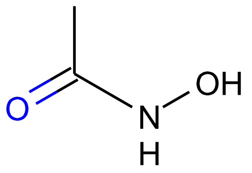 [*]=S	-0.0796

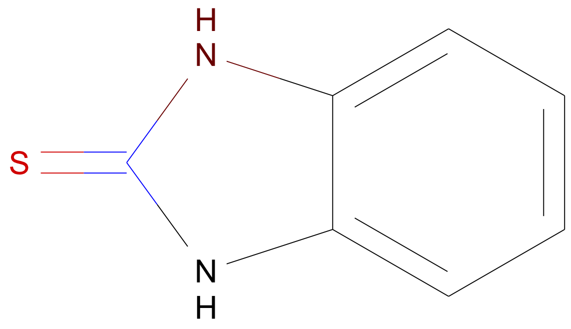

$C_7H_6N_2S$
Molecular Weight: 150.20094
ALogP: 2.536
Rotatable Bonds: 0
Acceptors: 1
Donors: 2

Model Prediction

Prediction: 0.185
Unit: g/kg_body_weight
Mahalanobis Distance: 7.3
Mahalanobis Distance p-value: 0.0121

Mahalanobis Distance: The Mahalanobis distance (MD) is a generalization of the Euclidean distance that accounts for correlations among the X properties. It is calculated as the distance to the center of the training data. The larger the MD, the less trustworthy the prediction.
Mahalanobis Distance p-value: The p-value gives the fraction of training data with an MD greater than or equal to the one for the given sample, assuming normally distributed data. The smaller the p-value, the less trustworthy the prediciton. For highly non-normal X properties (e.g., fingerprints), the MD p-value is wildly inaccurate.

Structural Similar Compounds

Name	MONURON	M-CRESIDINE	RESORCINOL
Structure	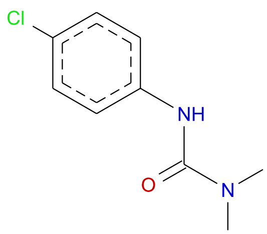	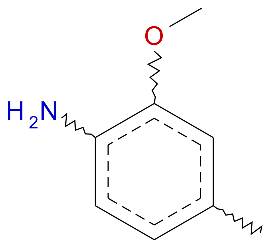	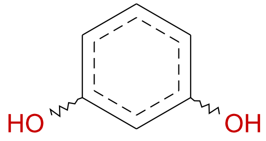
Actual Endpoint (-log C)	3.46878	5.93318	2.99262
Predicted Endpoint (-log C)	3.8732	3.87056	3.36172
Distance	0.572	0.614	0.635
Reference	NCI/NTP TR-266	NCI/NTP TR-105	NCI/NTP TR-403

Model Applicability

Unknown features are fingerprint features in the query molecule, but not found or appearing too infrequently in the training set.

1. All properties and OPS components are within expected ranges.
2. Unknown FCFP_2 feature: 1499521844: S=C1N[*]:[*]N1

Feature Contribution

Top features for positive contribution

Fingerprint	Bit/Smiles	Feature Structure	Score
FCFP_2	1	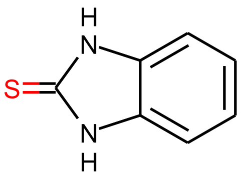 [*]=S	0.511

FCFP_2	3	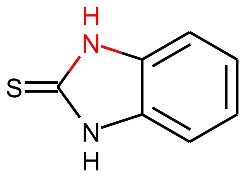 [*]N[*]	0.104

Top Features for negative contribution

Fingerprint	Bit/Smiles	Feature Structure	Score
FCFP_2	1872154524	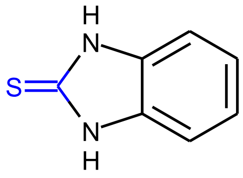 [*]C(=S)[*]	-0.307
FCFP_2	0	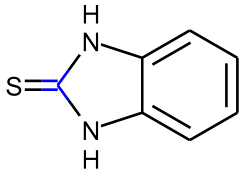 [*]C(=[*])[*]	-0.29

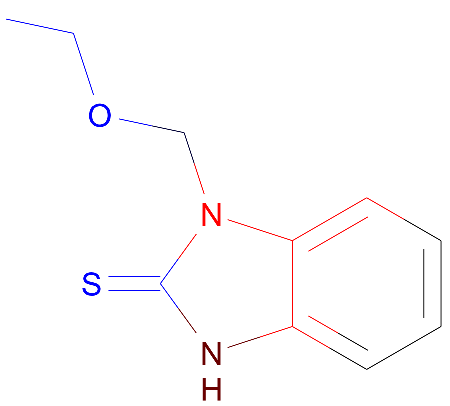

$C_{10}H_{12}N_2OS$
Molecular Weight: 208.28008
ALogP: 3.09
Rotatable Bonds: 3
Acceptors: 2
Donors: 1

Model Prediction

Prediction: 0.172
Unit: g/kg_body_weight
Mahalanobis Distance: 13.6
Mahalanobis Distance p-value: 4.63e-011

Mahalanobis Distance: The Mahalanobis distance (MD) is a generalization of the Euclidean distance that accounts for correlations among the X properties. It is calculated as the distance to the center of the training data. The larger the MD, the less trustworthy the prediction.
Mahalanobis Distance p-value: The p-value gives the fraction of training data with an MD greater than or equal to the one for the given sample, assuming normally distributed data. The smaller the p-value, the less trustworthy the prediciton. For highly non-normal X properties (e.g., fingerprints), the MD p-value is wildly inaccurate.

Structural Similar Compounds

Name	MONURON	M-CRESIDINE	BETA-NITROSTYRENE
Structure	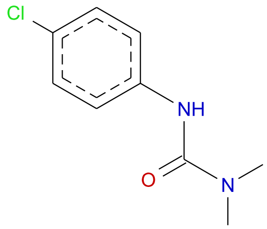	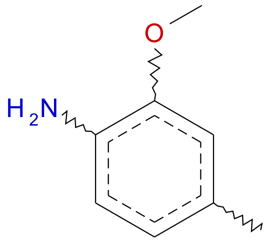	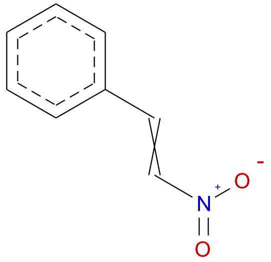
Actual Endpoint (-log C)	3.46878	5.93318	2.6965
Predicted Endpoint (-log C)	3.8732	3.87056	2.69255
Distance	0.563	0.615	0.665
Reference	NCI/NTP TR-266	NCI/NTP TR-105	NCI/NTP TR-170

Model Applicability

Unknown features are fingerprint features in the query molecule, but not found or appearing too infrequently in the training set.

1. All properties and OPS components are within expected ranges.
2. Unknown FCFP_2 feature: 470041467: [*]OCN([*])[*]

Feature Contribution

Top features for positive contribution			
**Fingerprint**	**Bit/Smiles**	**Feature Structure**	**Score**
FCFP_2	332760439	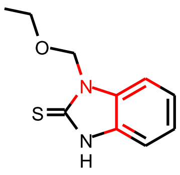 [*]N1[*][*][c](:[*]):[c]1:[cH]:[*]	0.672

FCFP_2	1	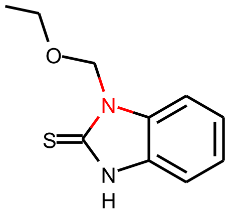 [*]=S	0.511
FCFP_2	3	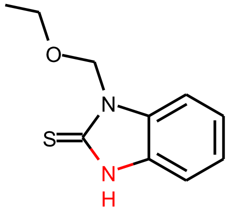 [*]N[*]	0.104

Top Features for negative contribution

Fingerprint	Bit/Smiles	Feature Structure	Score
FCFP_2	136597326	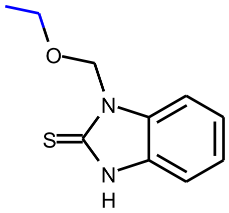 [*]CC	-0.489
FCFP_2	1872154524	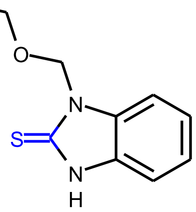 [*]C(=S)[*]	-0.307
FCFP_2	0	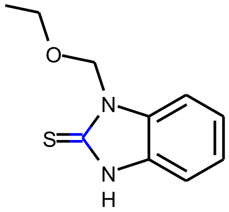 [*]C(=[*])[*]	-0.29

HAE

TOPKAT_Rat_Maximum_Tolerated_Dose_Gavage

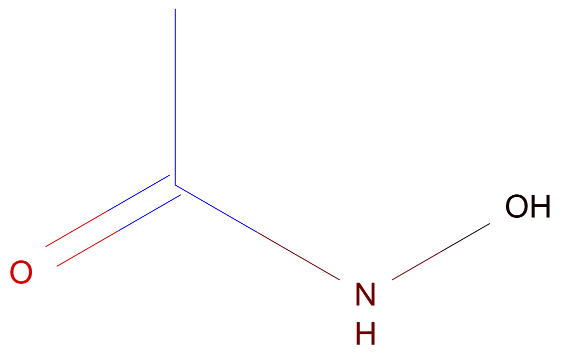

$C_2H_5NO_2$

Molecular Weight: 75.0666

ALogP: -0.82

Rotatable Bonds: 0

Acceptors: 2

Donors: 2

Model Prediction

Prediction: 0.434

Unit: g/kg_body_weight

Mahalanobis Distance: 7.33

Mahalanobis Distance p-value: 0.0114

Mahalanobis Distance: The Mahalanobis distance (MD) is a generalization of the Euclidean distance that accounts for correlations among the X properties. It is calculated as the distance to the center of the training data. The larger the MD, the less trustworthy the prediction.
Mahalanobis Distance p-value: The p-value gives the fraction of training data with an MD greater than or equal to the one for the given sample, assuming normally distributed data. The smaller the p-value, the less trustworthy the prediciton. For highly non-normal X properties (e.g., fingerprints), the MD p-value is wildly inaccurate.

Structural Similar Compounds

Name	N-METHYLOLACRYLAMIDE	METHYL CARBAMATE	MONOCHLOROACETIC ACID
Structure	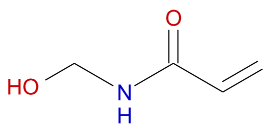	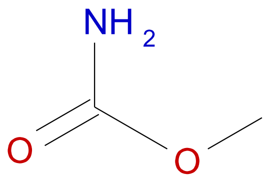	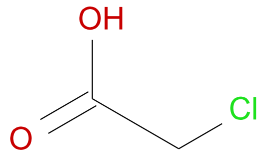
Actual Endpoint (-log C)	3.92559	2.57442	3.49828
Predicted Endpoint (-log C)	3.22367	2.53169	3.74848
Distance	0.464	0.582	0.629
Reference	NCI/NTP TR-352	NCI/NTP TR-328	NCI/NTP TR-396

Model Applicability

Unknown features are fingerprint features in the query molecule, but not found or appearing too infrequently in the training set.

1. All properties and OPS components are within expected ranges.
2. Unknown FCFP_2 feature: 1666606459: [*]C(=[*])NO
3. Unknown FCFP_2 feature: -548542844: [*]NO

Feature Contribution

Top features for positive contribution			
**Fingerprint**	**Bit/Smiles**	**Feature Structure**	**Score**
FCFP_2	1	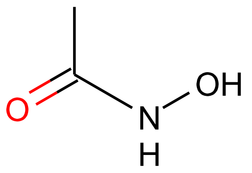 [*]=S	0.511

FCFP_2	3	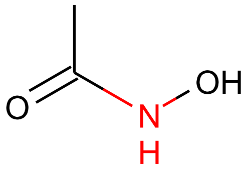 [*]N[*]	0.104
FCFP_2	7	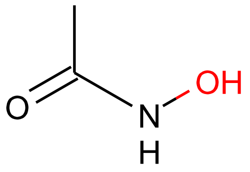 [*]O	0.0144

Top Features for negative contribution

Fingerprint	Bit/Smiles	Feature Structure	Score
FCFP_2	136597326	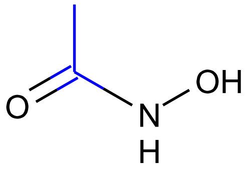 [*]CC	-0.489
FCFP_2	1872154524	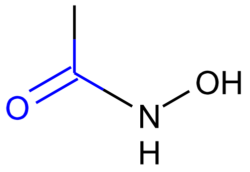 [*]C(=S)[*]	-0.307
FCFP_2	0	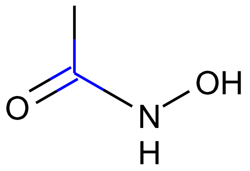 [*]C(=[*])[*]	-0.29

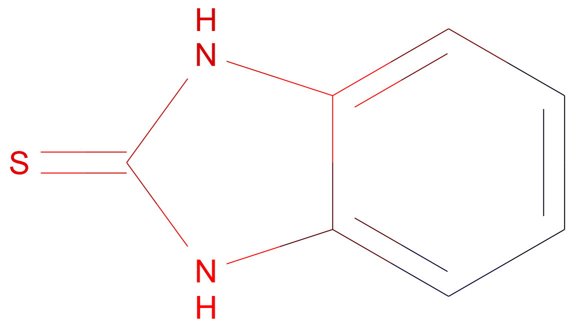

$C_7H_6N_2S$
Molecular Weight: 150.20094
ALogP: 2.536
Rotatable Bonds: 0
Acceptors: 1
Donors: 2

Model Prediction

Prediction: 0.271
Unit: g/kg_body_weight
Mahalanobis Distance: 19.8
Mahalanobis Distance p-value: 2.13e-008

Mahalanobis Distance: The Mahalanobis distance (MD) is a generalization of the Euclidean distance that accounts for correlations among the X properties. It is calculated as the distance to the center of the training data. The larger the MD, the less trustworthy the prediction.
Mahalanobis Distance p-value: The p-value gives the fraction of training data with an MD greater than or equal to the one for the given sample, assuming normally distributed data. The smaller the p-value, the less trustworthy the prediciton. For highly non-normal X properties (e.g., fingerprints), the MD p-value is wildly inaccurate.

Structural Similar Compounds

Name	1-PHENYL-2-THIOUREA	m-TOLYLUREA	PHENYLUREA
Structure	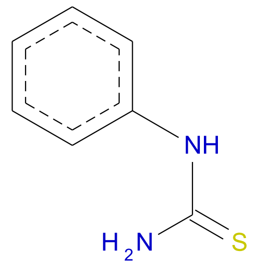	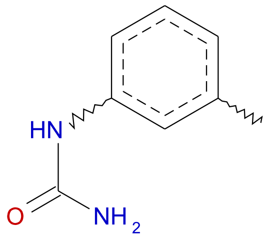	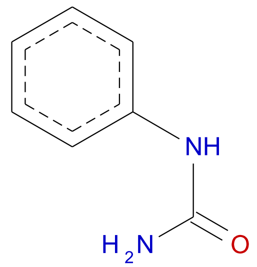
Actual Endpoint (-log C)	4.705	2.053	1.833
Predicted Endpoint (-log C)	2.82778	1.90667	2.30027
Distance	0.462	0.496	0.512
Reference	JMPCAS 4;109;61	IIFBA4 12;195;69	IIFBA4 12;195;69

Model Applicability

Unknown features are fingerprint features in the query molecule, but not found or appearing too infrequently in the training set.

1. All properties and OPS components are within expected ranges.
2. Unknown FCFP_6 feature: 16: [*]:[cH]:[*]
3. Unknown FCFP_6 feature: 1618154665: [*]:[cH]:[cH]:[cH]:[*]

Feature Contribution

Top features for positive contribution			
**Fingerprint**	**Bit/Smiles**	**Feature Structure**	**Score**
ECFP_6	642810091	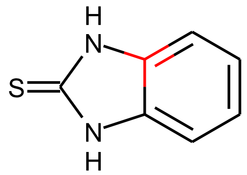 [*][c](:[*]):[*]	0.281

FCFP_6	1499521844	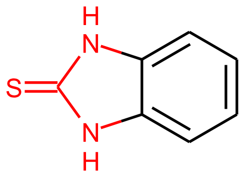 S=C1N[*]:[*]N1	0.258
ECFP_6	-154530762	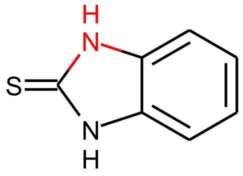 [*]N[*]	0.0652
**Top Features for negative contribution**			
**Fingerprint**	**Bit/Smiles**	**Feature Structure**	**Score**
FCFP_6	3	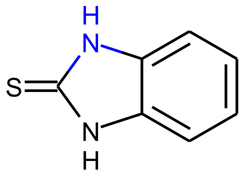 [*]N[*]	-0.107
ECFP_6	1997021792	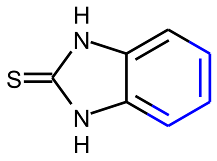 [*]:[cH]:[cH]:[cH]:[*]	-0.0912
FCFP_6	0	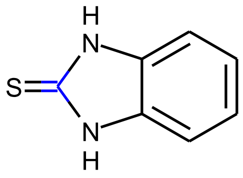 [*]C(=[*])[*]	-0.0791

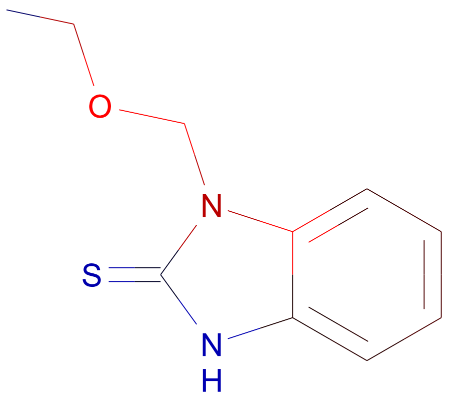

$C_{10}H_{12}N_2OS$

Molecular Weight: 208.28008

ALogP: 3.09

Rotatable Bonds: 3

Acceptors: 2

Donors: 1

Model Prediction

Prediction: 0.315

Unit: g/kg_body_weight

Mahalanobis Distance: 23.3

Mahalanobis Distance p-value: 5.58e-022

Mahalanobis Distance: The Mahalanobis distance (MD) is a generalization of the Euclidean distance that accounts for correlations among the X properties. It is calculated as the distance to the center of the training data. The larger the MD, the less trustworthy the prediction.
Mahalanobis Distance p-value: The p-value gives the fraction of training data with an MD greater than or equal to the one for the given sample, assuming normally distributed data. The smaller the p-value, the less trustworthy the prediciton. For highly non-normal X properties (e.g., fingerprints), the MD p-value is wildly inaccurate.

Structural Similar Compounds

Name	CARBAMIC ACID; O-CHLORO-.alpha.-ETHYLBENZYL ESTER	TIFEMOXONE	UREA; 1;1-DIMETHYL-3-(m-METHYLPHENYL)2-THIO-
Structure	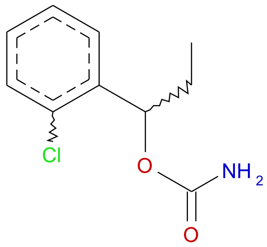	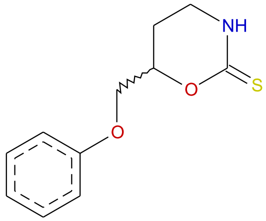	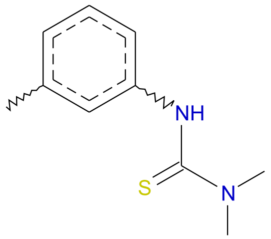
Actual Endpoint (-log C)	2.479	2.006	1.946
Predicted Endpoint (-log C)	2.14594	2.01841	2.30329
Distance	0.452	0.478	0.492
Reference	ARZNAD 13;856;63	ATSUDG 1;229;78	FMCHA2 -;C190;89

Model Applicability

Unknown features are fingerprint features in the query molecule, but not found or appearing too infrequently in the training set.

1. All properties and OPS components are within expected ranges.
2. Unknown ECFP_2 feature: 434461827: [*]N1[*]:[*]NC1=S
3. Unknown FCFP_6 feature: 16: [*]:[cH]:[*]
4. Unknown FCFP_6 feature: 1618154665: [*]:[cH]:[cH]:[cH]:[*]

Feature Contribution

Top features for positive contribution			
Fingerprint	Bit/Smiles	Feature Structure	Score
FCFP_6	470041467	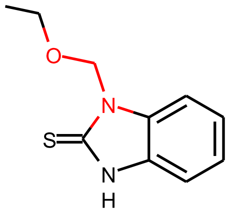 [*]OCN([*])[*]	0.322

ECFP_6	642810091	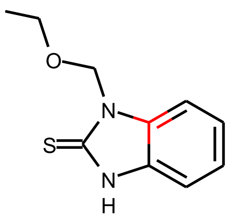 [*][c](:[*]):[*]	0.281
FCFP_6	675769755	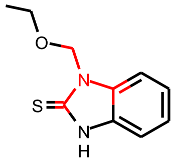 [*]CN1C(=[*])[*][*]:[c]1:[*]	0.155

Top Features for negative contribution

Fingerprint	Bit/Smiles	Feature Structure	Score
ECFP_6	734603939	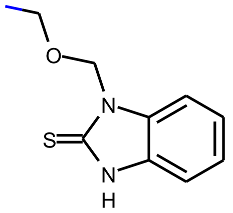 [*]C	-0.201
FCFP_6	-1986098826	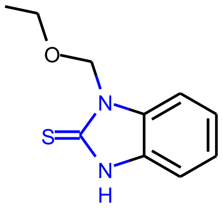 [*]N1[*]:[*]NC1=S	-0.189
ECFP_6	-1236953626	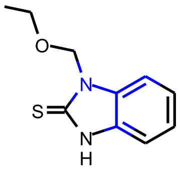 [*]N1[*][*][c](:[*]): [c]1:[cH]:[*]	-0.11

HAE — TOPKAT_Rat_Oral_LD50

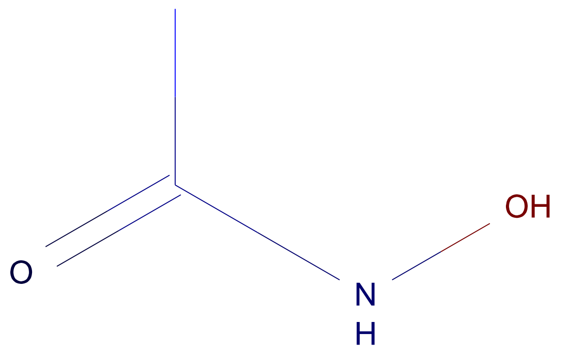

$C_2H_5NO_2$

Molecular Weight: 75.0666

ALogP: -0.82

Rotatable Bonds: 0

Acceptors: 2

Donors: 2

Model Prediction

Prediction: 1.09

Unit: g/kg_body_weight

Mahalanobis Distance: 16.7

Mahalanobis Distance p-value: 0.0714

Mahalanobis Distance: The Mahalanobis distance (MD) is a generalization of the Euclidean distance that accounts for correlations among the X properties. It is calculated as the distance to the center of the training data. The larger the MD, the less trustworthy the prediction.
Mahalanobis Distance p-value: The p-value gives the fraction of training data with an MD greater than or equal to the one for the given sample, assuming normally distributed data. The smaller the p-value, the less trustworthy the prediciton. For highly non-normal X properties (e.g., fingerprints), the MD p-value is wildly inaccurate.

Structural Similar Compounds

Name	MALEIC HYDRAZIDE	METHYLHYDRAZINE	1-AMINO-2-PROPANOL
Structure	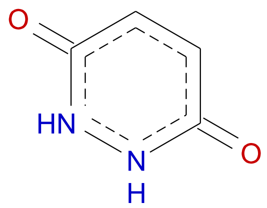	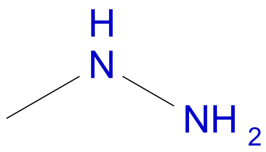	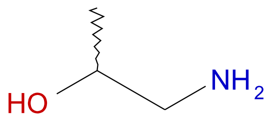
Actual Endpoint (-log C)	1.47	3.158	1.554
Predicted Endpoint (-log C)	1.89666	2.25156	2.12407
Distance	0.458	0.475	0.475
Reference	WRPCA2 9;119;70	XAWPA2 CWL	JACTDZ 6(1);53;87

Model Applicability

Unknown features are fingerprint features in the query molecule, but not found or appearing too infrequently in the training set.

1. All properties and OPS components are within expected ranges.
2. Unknown FCFP_6 feature: 1666606459: [*]C(=[*])NO
3. Unknown FCFP_6 feature: -548542844: [*]NO

Feature Contribution

Top features for positive contribution

Fingerprint	Bit/Smiles	Feature Structure	Score
ECFP_6	-1897341097	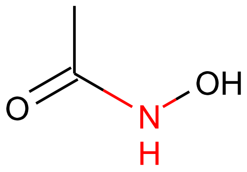 [*]N[*]	0.216

ECFP_6	-1074141656	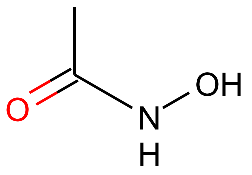<br>[*]=O	0.142
FCFP_6	136597326	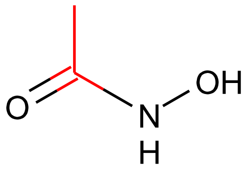<br>[*]CC	0.0977
**Top Features for negative contribution**			
**Fingerprint**	**Bit/Smiles**	**Feature Structure**	**Score**
FCFP_6	566058135	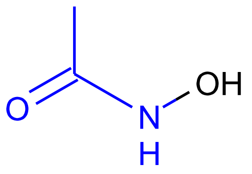<br>[*]NC(=O)C	-0.216
ECFP_6	734603939	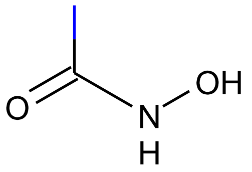<br>[*]C	-0.201
FCFP_6	3	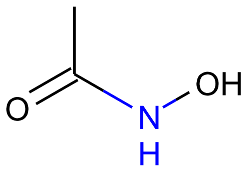<br>[*]N[*]	-0.107
